# Further correction: Reductive annulations of arylidene malonates with unsaturated electrophiles using photoredox/Lewis acid cooperative catalysis

**DOI:** 10.1039/d6sc90025a

**Published:** 2026-02-16

**Authors:** Rick C. Betori, Benjamin R. McDonald, Karl A. Scheidt

**Affiliations:** a Department of Chemistry, Center for Molecular Innovation and Drug Discovery, Northwestern University 2145 Sheridan Road Evanston Illinois 60208 USA Scheidt@northwestern.edu

## Abstract

Further correction for ‘Reductive annulations of arylidene malonates with unsaturated electrophiles using photoredox/Lewis acid cooperative catalysis’ by Rick C. Betori *et al.*, *Chem. Sci.*, 2019, **10**, 3353–3359, https://doi.org/10.1039/C9SC00302A.

The corresponding author regrets that following publication of the article he became aware that the Stern–Volmer experimental results in Fig. 6 had been fabricated. All other results and the supplementary information (SI) were correct. The relevant Stern–Volmer (SV) experiment has been conducted and corrected SV data has replaced those originally published in Fig. 6. The resulting data has indicated a modified description of the mechanistic pathway for the overall transformation.

A corrected version of Fig. 6 is provided below and the SI for the original manuscript has been updated and included with this correction.

**Fig. 6:**
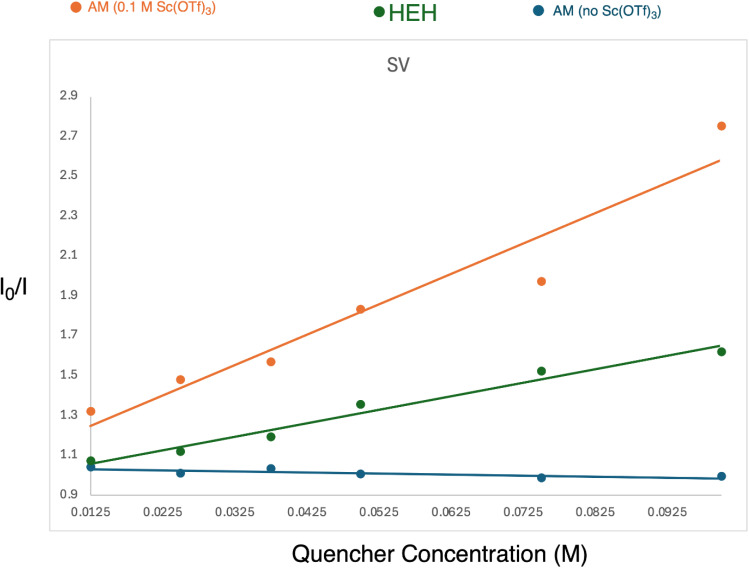
Stern–Volmer fluorescence quenching analysis.

The Royal Society of Chemistry apologises for these errors and any consequent inconvenience to authors and readers.

