# Supplementary material for: Further correction: Reductive annulations of arylidene malonates with unsaturated electrophiles using photoredox/Lewis acid cooperative catalysis
Source: Chem Sci. 2026 Feb 16;17(9):4825–6. doi: 10.1039/d6sc90025a (PMC12908705; doi:10.1039/d6sc90025a)
Supplement: SC-017-D6SC90025A-s001 [file SC-017-D6SC90025A-s001.pdf]

Supplementary Information (SI) for Chemical Science.
This journal is © The Royal Society of Chemistry 2026

This version of the SI published 17/02/2026 is updated in line with the correction article. The Stern-Volmer data have been replaced with a corrected version.

Supporting Information

Reductive Annulations of Arylidene Malonates With Unsaturated Electrophiles Using Photoredox/Lewis Acid Cooperative Catalysis

Rick C. Betori, Benjamin R. McDonald and Karl A. Scheidt*

Department of Chemistry, Center for Molecular Innovation and Drug Discovery,
Northwestern University, 2145 Sheridan Road, Evanston, Illinois 60208, United States

Table of Contents

General Information S2
Preparation of Salicylaldehyde Derived Arylidene Malonates S2
Preparation of Salicylaldehyde Derived Arylidene Malonates Requiring Metathesis Reactions S9
Preparation of Salicylaldehyde Derived Alkyne Electrophiles S11
Preparation of Salicylaldehyde Derived Alkyne-Aryl Arylidene Malonates S13
Preparation of Tetrahydroquinoline-Precursor Arylidene Malonate S14
Preparation of Tetrahydronapthalene-Precursor Arylidene Malonate S15
Preparation of Dihydrobenzofuran-Precursor Arylidene Malonate S17
Selected Optimization Data: S18
General Procedure for Reductive Cyclization S20
Procedure for Krapcho Decarboxylation and Dieckmann Condensation S30
Stern-Volmer Fluorescence Quenching Experiments S31
Procedure for Determination of Quantum Yield S32
Procedure for Light/Dark Experiment S33
Procedure for UV-Vis Experiments S33
ORTEPS of Crystallographic Structure S34
NMR Spectra for Cyclization Starting Materials S35
NMR Spectra for Cyclization Products S58
References: S84

General Information

All reactions were carried out under an argon or nitrogen atmosphere in flame-dried glassware with magnetic stirring. Solvents used in reactions were purified by passage through a bed of activated alumina. Unless stated otherwise, reagents were purified prior to use following the guidelines of Perrin and Armarego.[1] Purification of reaction products was carried out by flash chromatography on Biotage Isolera 4 systems with Ultra-grade silica cartridges. Analytical thin layer chromatography was performed on EM Reagent 0.25 mm silica gel 60-F plates. Visualization was accomplished with UV light. Infrared spectra were recorded on a Bruker Tensor 37 FT-IR spectrometer. ^{1}H NMR spectra were recorded on an AVANCE III 500 MHz spectrometer with direct cryoprobe (500 MHz) and Bruker Avance III 600 MHz (151 MHz) system. Spectra are reported in ppm using solvent as an internal standard ($CDCl_3$ at 7.26 ppm). Peak multiplicities are reported as (s = singlet, d = doublet, t = triplet, q = quartet, quint= quintet, m = multiplet, br= broad; coupling constant(s) in Hz; integration.) Proton-decoupled ^{13}C NMR spectra were recorded on an AVANCE III 500 MHz with direct cryoprobe (125 MHz) spectrometer and Bruker Avance III 600 MHz (151 MHz) system. These are reported in ppm using solvent as an internal standard ($CDCl_3$ at 77.16 ppm). Low-resolution mass spectra were obtained on WATERS Acquity-H UPLC-MS with a single quad detector (ESI) Varian1200 Quadrupole Mass Spectrometer. High-resolution mass spectra were obtained using an Agilent 6120A LC-time of flight mass spectrometer. Gas chromatography experiments were run on Agilent 7890A/5975C GC/MS System. Enantioselectivity measurements were made on an Agilent 1290 Infinity SFC, using a Chiralpak ID-3 column. Blue light was generated by 3 40 W Kessil H150 LED lights.

Iridium and Ruthenium photocatalysts were obtained from Strem Chemical and Sigma-Aldrich respectively and used as received. Photocatalysts 3DPAFIPN and 4CZIPN were synthesized according to Molander et al.[2]

Preparation of Salicylaldehyde Derived Arylidene Malonates

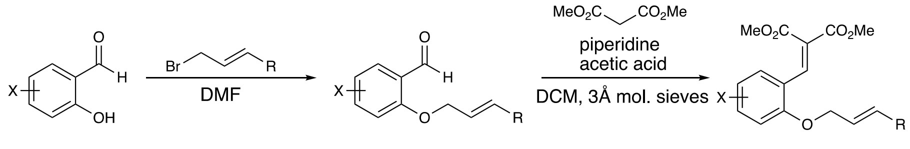

To an oven-dried scintillation vial under nitrogen was added NaH (60 wt %, 1.2 equiv) and DMF (0.5 M), and the mixture was cooled to 0 ºC. A solution of salicylaldehyde (1 equiv) dissolved in DMF (1.0 M) was slowly added, and upon addition completion, the mixture was allowed to stir at 0 ºC for 30 minutes. A solution of allyl bromide electrophile (1.2 equiv) dissolved in DMF (1.0 M) was slowly added, and then reaction mixture was allowed to stir overnight as it warmed to room temperature. Upon reaction completion, sat. aq. NH_4Cl was added, and the aqueous layer was extracted 3x with EtOAc. The combined organic layers were washed with H_2O and sat. aq. NaCl, passed through a

Biotage Isolute phase separator, and concentrated to dryness under reduced pressure on a rotary evaporator to obtain the crude SN2 product, which was directly used in the next reaction without purification.

To a scintillation vial was added the crude aldehyde from the previous step (1 equiv). Malonate or ketoester was added (1.1 equiv), along with 250 wt % activated 4 Å molecular sieves (powder). A magnetic stir bar and CH_2Cl_2 (2.0 M) were added, followed by acetic acid (0.1 equiv) and piperidine (0.1 equiv). The mixture was stirred overnight. Silica was added and the reaction mixture was concentrated under reduced pressure. The resulting mixture was loaded onto a column of silica and purified via flash column chromatography (10-20% EtOAc/hexanes) to yield the desired product.

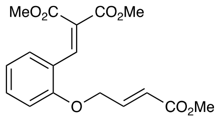

Prepared according to the general procedure with 78% overall yield over two steps.

Analytical Data: ^{1}H NMR (500 MHz, Chloroform-*d*) δ 7.98 (s, 1H), 7.36 (t, *J* = 8.2 Hz, 2H), 7.09 (dt, *J* = 15.8, 4.1 Hz, 1H), 6.96 (t, *J* = 7.6 Hz, 1H), 6.85 (d, *J* = 8.2 Hz, 1H), 6.16 (dd, *J* = 15.9, 2.3 Hz, 1H), 4.77 (dd, *J* = 4.1, 2.0 Hz, 2H), 3.78 (s, 3H), 3.76 (s, 3H), 2.44 (s, 3H). ^{13}C NMR (126 MHz, $CDCl_3$) δ 168.1, 166.3, 156.4, 141.9, 137.1, 134.7, 132.3, 129.1, 122.7, 122.0, 121.5, 112.1, 67.0, 66.6, 60.6, 52.5, 52.5, 52.4, 51.8, 51.6. LRMS (ESI): Mass calcd for $C_{17}H_{18}O_7$ [M+H]+: 335.1; found 335.2 HRMS (ESI): Mass calcd for $C_{17}H_{18}O_7$ [M+H]+: 335.1053; found 335.1051 FTIR (neat): 2970, 2732, 1790, 1675, 1656, 1618, 1531, 1466, 1271, 1261, 1200, 1186, 1152, 1045, 1011, 937, 852, 808

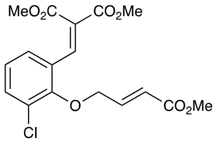

Prepared according to the general procedure with 72% overall yield over two steps.

Analytical Data: ^{1}H NMR (500 MHz, Chloroform-*d*) δ 8.11 (s, 1H), 7.60 (dd, *J* = 8.0, 1.4 Hz, 1H), 7.46 – 7.39 (m, 1H), 7.26 – 7.17 (m, 2H), 6.42 (dt, *J* = 15.7, 1.7 Hz, 1H), 4.78 (dd, *J* = 4.7, 1.9 Hz, 2H), 4.02 (s, 3H), 3.93 (d, *J* = 2.2 Hz, 6H). ^{13}C NMR (126 MHz, $CDCl_3$) δ 166.4, 166.2, 164.0, 153.3, 141.7, 137.9, 132.5, 129.4, 128.6, 128.3, 127.4, 125.4, 122.3, 72.6, 52.8, 52.6, 51.7. LRMS (ESI): Mass calcd for $C_{17}H_{17}ClO_7$ [M+H]+: 369.1; found 369.1 HRMS (ESI): Mass calcd for $C_{17}H_{17}ClO_7$ [M+H]+: 369.0663; found 369.0660 FTIR (neat): 2988, 2738, 1762, 1685, 1637, 1600, 1518, 1315, 1292, 1246, 1209, 1179, 1158, 1075, 1017, 975, 914, 736

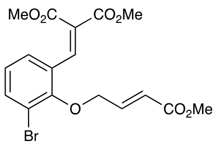

Prepared according to the general procedure with 71% overall yield over two steps.

Analytical Data: ^{1}H NMR (500 MHz, Chloroform-*d*) δ 7.93 (s, 1H), 7.62 – 7.56 (m, 1H), 7.27 (d, *J* = 7.7 Hz, 1H), 7.08 – 6.96 (m, 2H), 6.25 (d, *J* = 15.4 Hz, 1H), 4.60 – 4.54 (m, 2H), 3.83 (s, 3H), 3.75 (d, *J* = 5.3 Hz, 6H). ^{13}C NMR (126 MHz, $CDCl_3$) δ 166.4, 166.2, 163.9, 154.3, 141.6, 138.1, 135.5, 129.4, 128.4, 128.2, 125.9, 122.4, 118.0, 72.7, 52.8, 52.7, 51.7. LRMS (ESI): Mass calcd for $C_{17}H_{17}BrO_7$ [M+H]+: 413.0; found 413.1 HRMS (ESI): Mass calcd for $C_{17}H_{17}BrO_7$ [M+H]+: 413.0158; found 413.0160 FTIR (neat): 2984, 2784, 2345, 1676, 1659, 1628, 1552, 1481, 1280, 1248, 1214, 1166, 1129, 1044, 1008, 961, 884, 708

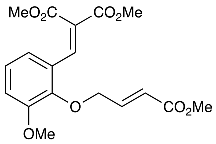

Prepared according to the general procedure with 67% overall yield over two steps.

Analytical Data: ^{1}H NMR (500 MHz, Chloroform-*d*) δ 8.24 (s, 1H), 7.29 – 7.20 (m, 2H), 7.19 – 7.09 (m, 2H), 6.41 (dd, *J* = 15.8, 2.2 Hz, 1H), 4.86 (dd, *J* = 4.9, 1.9 Hz, 2H), 4.10 – 4.02 (m, 6H), 3.98 – 3.93 (m, 6H). ^{13}C NMR (126 MHz, $CDCl_3$) δ 166.8, 166.6, 164.3, 152.6, 146.5, 143.0, 138.6, 127.7, 127.0, 124.6, 121.7, 120.2, 114.6, 72.0, 55.9, 52.6, 52.5, 51.6. LRMS (ESI): Mass calcd for $C_{18}H_{20}O_8$[M+H]+: 365.1; found 365.2 HRMS (ESI): Mass calcd for $C_{18}H_{20}O_8$[M+H]+: 365.1158; found 365.1160 FTIR (neat): 2984, 2444, 2241, 1683, 1641, 1625, 1511, 1467, 1276, 1253, 1210, 1167, 1163, 1102, 1016, 961, 878, 701

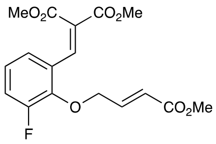

Prepared according to the general procedure with 73% overall yield over two steps.

Analytical Data: ^{1}H NMR (500 MHz, Chloroform-*d*) δ 8.12 (s, 1H), 7.31 – 7.22 (m, 2H), 7.22 – 7.11 (m, 2H), 6.33 (dt, *J* = 15.7, 1.9 Hz, 1H), 4.90 (dd, *J* = 4.8, 1.8 Hz, 2H), 3.98 (s, 3H), 3.89 (d, *J* = 6.2 Hz, 6H). ^{13}C NMR (126 MHz, $CDCl_3$) δ 166.4, 166.3, 164.1, 155.0 (d, *J* = 248.0 Hz), 144.8 (d, *J* = 11.4 Hz), 142.0, 137.4 (d, *J* = 3.6 Hz), 128.5 (d, *J* = 3.2 Hz), 127.8, 124.2 (d, *J* = 7.7 Hz), 124.1 (d, *J* = 3.2 Hz), 122.2, 119.0 (d, *J* = 19.5 Hz), 72.5 (d, *J* = 5.9 Hz), 52.7, 52.6, 51.7. LRMS (ESI): Mass calcd for $C_{17}H_{17}FO_7$ [M+H]+: 353.1; found 353.1 HRMS (ESI): Mass calcd for $C_{17}H_{17}FO_7$ [M+H]+: 353.0959;

found 353.0961 FTIR (neat): 2999, 2398, 2107, 1706, 1657, 1605, 1586, 1379, 1303, 1259, 1195, 1187, 1133, 1089, 997, 967, 883, 707

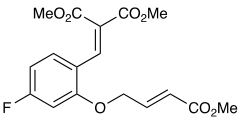

Prepared according to the general procedure with 61% overall yield over two steps.
Analytical Data: ^{1}H NMR (500 MHz, Chloroform-*d*) δ 7.93 (s, 1H), 7.24 (dd, *J* = 8.6, 6.6 Hz, 1H), 6.94 (dt, *J* = 15.8, 4.2 Hz, 1H), 6.56 (td, *J* = 8.3, 2.1 Hz, 1H), 6.47 (dd, *J* = 10.4, 2.3 Hz, 1H), 6.07 – 6.00 (m, 1H), 4.64 (dd, *J* = 4.0, 2.0 Hz, 2H), 3.74 (t, *J* = 1.1 Hz, 3H), 3.70 – 3.63 (m, 6H). ^{13}C NMR (126 MHz, $CDCl_3$) δ 167.0, 166.1, 164.5, 164.9 (d, *J* = 252.3 Hz), 157.8 (d, *J* = 10.2 Hz), 141.1, 137.3, 130.4 (d, *J* = 10.4 Hz), 125.6, 122.3, 118.7 (d, *J* = 3.2 Hz), 108.3 (d, *J* = 21.8 Hz), 100.5 (d, *J* = 26.0 Hz), 67.2, 52.6, 52.5, 51.8. LRMS (ESI): Mass calcd for $C_{17}H_{17}FO_7$ [M+H]+: 353.1; found 353.1 HRMS (ESI): Mass calcd for $C_{17}H_{17}FO_7$ [M+H]+: 353.0959; found 353.0960 FTIR (neat): 2993, 2942, 1766, 1690, 1655, 1616, 1562, 1445, 1298, 1249, 1191, 1186, 1151, 1042, 1000, 961, 922, 807

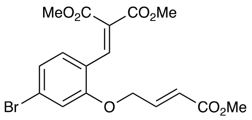

Prepared according to the general procedure with 75% overall yield over two steps.
Analytical Data: ^{1}H NMR (500 MHz, Chloroform-*d*) δ 8.26 (s, 1H), 7.46 (d, *J* = 8.2 Hz, 1H), 7.37 – 7.32 (m, 1H), 7.35 – 7.26 (m, 1H), 7.24 (d, *J* = 1.8 Hz, 1H), 6.39 (dt, *J* = 15.8, 2.0 Hz, 1H), 5.00 (dd, *J* = 4.2, 2.1 Hz, 2H), 4.10 (s, 3H), 4.02 (d, *J* = 7.2 Hz, 6H). ^{13}C NMR (126 MHz, $CDCl_3$) δ 165.8, 165.1, 163.3, 155.7, 140.1, 136.3, 129.0, 125.4, 124.7, 123.6, 121.3, 120.6, 114.7, 66.2, 51.7, 51.6, 50.8. LRMS (ESI): Mass calcd for $C_{17}H_{17}BrO_7$ [M+H]+: 413.0; found 413.1 HRMS (ESI): Mass calcd for $C_{17}H_{17}BrO_7$ [M+H]+: 413.0158; found 413.0156 FTIR (neat): 2969, 2582, 1919, 1688, 1652, 1615, 1491, 1320, 1271, 1256, 1215, 1165, 1129, 1077, 1001, 933, 891, 813

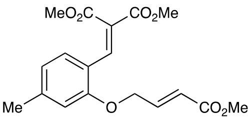

^{1}H NMR (500 MHz, Chloroform-*d*) δ = 8.14 (s, 1H), 7.25 (d, *J*=7.9, 1H), 7.09 (dt, *J*=15.8, 4.1, 1H), 6.77 (d, *J*=7.9, 1H), 6.66 (s, 1H), 6.17 (dt, *J*=15.8, 2.0, 1H), 4.75 (dd, *J*=4.1, 2.0, 2H), 3.85 (s, 3H), 3.78 (d, *J*=16.8, 6H), 2.34 (s, 3H). ^{13}C NMR (126 MHz, $CDCl_3$) δ 167.3, 166.3, 164.7, 156.5, 143.2, 142.1,

138.3, 128.8, 124.7, 122.2, 121.9, 119.7, 112.9, 66.9, 52.5, 52.5, 51.7, 21.9. LRMS (ESI): Mass calcd for $C_{18}H_{20}O_7$ [M+H]+: 349.1; found 349.1 HRMS (ESI): Mass calcd for $C_{18}H_{20}O_7$ [M+H]+: 349.1209; found 349.1208 FTIR (neat): 3000, 2616, 2232, 1664, 1633, 1611, 1552, 1442, 1275, 1227, 1212, 1165, 1126, 1022, 1012, 925, 905, 803

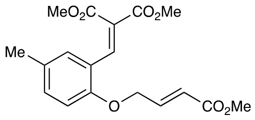

^{1}H NMR (500 MHz, Chloroform-*d*) δ = 8.22 (s, 1H), 7.23 (t, *J* = 3.1 Hz, 2H), 7.17 (dt, *J* = 15.7, 4.1 Hz, 1H), 6.86 – 6.81 (m, 1H), 6.25 (dt, *J* = 15.8, 2.1 Hz, 1H), 4.82 (dd, *J* = 4.2, 2.1 Hz, 2H), 3.95 (s, 3H), 3.89 (s, 3H), 3.85 (s, 3H), 2.35 (s, 3H). ^{13}C NMR (126 MHz, $CDCl_3$) δ 167.1, 166.3, 164.6, 154.5, 142.2, 138.6, 132.6, 130.6, 129.5, 125.5, 122.3, 121.9, 112.1, 67.1, 52.6, 52.4, 51.7, 20.5. LRMS (ESI): Mass calcd for $C_{18}H_{20}O_7$ [M+H]+: 349.1; found 349.1 HRMS (ESI): Mass calcd for $C_{18}H_{20}O_7$ [M+H]+: 349.1209; found 349.1210 FTIR (neat): 2967, 2772, 1975, 1678, 1650, 1612, 1571, 1435, 1283, 1231, 1192, 1188, 1143, 1070, 992, 943, 897, 792

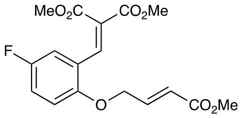

^{1}H NMR (500 MHz, Chloroform-*d*) δ 8.22 (s, 1H), 7.29 – 7.17 (m, 3H), 7.01 – 6.91 (m, 1H), 6.36 – 6.25 (m, 1H), 4.93 – 4.87 (m, 2H), 4.02 (d, *J* = 3.0 Hz, 3H), 4.00 – 3.95 (m, 3H), 3.94 – 3.89 (m, 3H). ^{13}C NMR (126 MHz, $CDCl_3$) δ 166.6, 166.2, 164.3, 156.9 (d, *J* = 240.7 Hz), 152.7, 141.7, 137.0, 127.0, 123.7 (d, *J* = 8.0 Hz), 122.1, 118.2 (d, *J* = 23.3 Hz), 115.5 (d, *J* = 24.5 Hz), 113.4 (d, *J* = 8.2 Hz), 67.7, 52.8, 52.7, 51.8. LRMS (ESI): Mass calcd for $C_{17}H_{17}FO_7$ [M+H]+: 353.1; found 353.1 HRMS (ESI): Mass calcd for $C_{17}H_{17}FO_7$ [M+H]+: 353.0959; found 353.0961 FTIR (neat): 2992, 2745, 2037, 1696, 1634, 1608, 1500, 1439, 1278, 1224, 1205, 1175, 1162, 1027, 998, 962, 920, 815

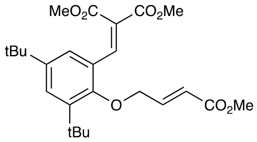

^{1}H NMR (500 MHz, Chloroform-*d*) δ = 7.78 (s, 1H), 7.24 (d, *J*=2.5, 1H), 7.06 (d, *J*=2.3, 1H), 6.89 (dt, *J*=15.7, 3.9, 1H), 6.15 (dd, *J*=15.7, 2.2, 1H), 4.34 (dd, *J*=3.9, 2.2, 2H), 3.69 – 3.54 (m, 9H), 1.21 (s, 9H), 1.12 (s, 9H). ^{13}C NMR (126 MHz, $CDCl_3$) δ 166.8, 166.6, 164.3, 154.9, 146.5, 142.8, 142.2, 141.1, 127.0, 126.9, 126.3, 124.4, 121.2, 73.6, 52.6, 52.6, 51.7, 35.3, 34.7, 31.4, 30.7. LRMS (ESI): Mass

calcd for $C_{25}H_{34}O_7$ [M+H]+: 447.2; found 447.1 HRMS (ESI): Mass calcd for $C_{25}H_{34}O_7$ [M+H]+: 447.2305; found 447.2307 FTIR (neat): 2978, 2578, 2191, 1689, 1632, 1606, 1592, 1310, 1291, 1267, 1207, 1185, 1138, 1066, 1002, 967, 851, 786

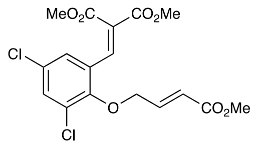

^{1}H NMR (500 MHz, Chloroform-*d*) δ = 7.65 (s, 1H), 7.24 (d, *J*=2.4, 1H), 7.03 (d, *J*=2.4, 1H), 6.83 (dt, *J*=15.8, 4.7, 1H), 6.03 (dt, *J*=15.7, 1.9, 1H), 4.40 (dd, *J*=4.8, 1.8, 2H), 3.67 (d, *J*=1.0, 3H), 3.59 (dd, *J*=16.2, 1.1, 6H). ^{13}C NMR (126 MHz, $CDCl_3$) δ 166.2, 165.6, 163.6, 151.9, 141.2, 136.5, 131.8, 130.3, 130.2, 129.4, 129.3, 127.3, 122.6, 72.7, 52.9, 52.7, 51.7. LRMS (ESI): Mass calcd for $C_{17}H_{16}Cl_2O_7$ [M+H]+: 403.1; found 403.1 HRMS (ESI): Mass calcd for $C_{17}H_{16}Cl_2O_7$ [M+H]+: 403.0273; found 403.0271 FTIR (neat): 2967, 2537, 1842, 1708, 1654, 1614, 1538, 1391, 1306, 1262, 1206, 1188, 1131, 1120, 993, 935, 902, 748

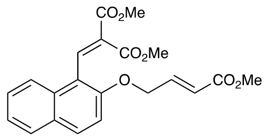

Prepared according to the general procedure with 78% overall yield over two steps.

Analytical Data: ^{1}H NMR (500 MHz, Chloroform-*d*) δ = 8.37 (s, 1H), 7.95 – 7.85 (m, 3H), 7.63 – 7.55 (m, 1H), 7.52 – 7.44 (m, 1H), 7.26 – 7.12 (m, 2H), 6.22 (dt, *J*=15.8, 2.1, 1H), 4.92 (dd, *J*=4.3, 2.0, 2H), 4.01 (s, 3H), 3.83 (s, 3H), 3.58 (s, 3H). ^{13}C NMR (126 MHz, $CDCl_3$) δ 166.3, 165.6, 164.9, 153.0, 142.4, 140.5, 131.9, 131.6, 129.9, 129.0, 128.4, 127.5, 124.5, 123.8, 122.1, 117.4, 113.9, 68.0, 52.7, 52.0, 51.7. LRMS (ESI): Mass calcd for $C_{21}H_{20}O_7$ [M+H]+: 385.1; found 385.1 HRMS (ESI): Mass calcd for $C_{21}H_{20}O_7$ [M+H]+: 385.1209; found 385.1210 FTIR (neat): 2996, 2543, 2196, 1677, 1641, 1622, 1543, 1441, 1306, 1266, 1192, 1163, 1148, 1046, 990, 930, 831, 792

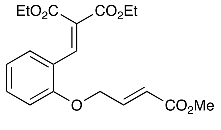

Prepared according to the general procedure with 85% overall yield over two steps.

Analytical Data: ^{1}H NMR (500 MHz, Chloroform-*d*) δ = 7.96 (s, 1H), 7.25 (dd, *J*=7.9, 1.6, 1H), 7.19 – 7.12 (m, 1H), 6.91 (dt, *J*=15.8, 3.9, 1H), 6.77 (t, *J*=7.5, 1H), 6.68 (d, *J*=8.3, 1H), 6.01 (dt, *J*=15.8, 2.1, 1H), 4.57 (dd, *J*=4.1, 2.1, 2H), 4.12 (dq, *J*=14.1, 7.1, 4H), 3.56 (s, 3H), 1.18 (t, *J*=7.1, 3H), 1.07 (t, *J*=7.1, 3H). ^{13}C NMR (126 MHz, $CDCl_3$) δ 166.5, 166.1, 164.1, 156.2, 142.0, 137.3, 131.9, 129.0,

126.6, 122.5, 121.6, 121.1, 112.0, 66.7, 61.4, 61.4, 51.5, 14.0, 13.8. LRMS (ESI): Mass calcd for $C_{19}H_{22}O_7$ [M+H]+: 363.1; found 363.2 HRMS (ESI): Mass calcd for $C_{19}H_{22}O_7$ [M+H]+: 363.1365; found 363.1363 FTIR (neat): 2991, 2700, 2301, 1699, 1655, 1602, 1512, 1400, 1296, 1250, 1200, 1181, 1131, 1043, 1002, 965, 840, 802

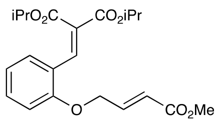

Prepared according to the general procedure with 75% overall yield over two steps.
Analytical Data: ^{1}H NMR (500 MHz, Chloroform-*d*) δ = 7.86 (s, 1H), 7.23 (dd, *J*=7.8, 1.5, 1H), 7.15 – 7.04 (m, 1H), 6.86 (dt, *J*=15.8, 3.9, 1H), 6.71 (t, *J*=7.6, 1H), 6.61 (d, *J*=8.3, 1H), 5.97 (dd, *J*=15.8, 2.0, 1H), 5.02 – 4.88 (m, 2H), 4.53 (dd, *J*=4.1, 2.1, 2H), 3.56 – 3.42 (m, 3H), 1.10 (d, *J*=6.3, 6H), 1.02 (d, *J*=6.1, 6H). ^{13}C NMR (126 MHz, $CDCl_3$) δ 166.3, 166.2, 166.1, 163.7, 156.3, 142.1, 136.6, 131.8, 129.2, 128.2, 127.4, 127.0, 122.8, 121.7, 121.1, 111.9, 69.1, 69.0, 66.8, 51.7, 21.5. LRMS (ESI): Mass calcd for $C_{21}H_{26}O_7$ [M+H]+: 390.2; found 390.2 HRMS (ESI): Mass calcd for $C_{21}H_{26}O_7$ [M+H]+: 390.1679; found 390.1680 FTIR (neat): 2998, 2486, 1967, 1703, 1639, 1614, 1546, 1385, 1280, 1237, 1203, 1179, 1124, 1030, 994, 930, 846, 705

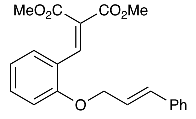

Prepared according to the general procedure with 82% overall yield over two steps.
Analytical Data: ^{1}H NMR (500 MHz, Chloroform-*d*) δ = 8.14 (s, 1H), 7.35 – 7.20 (m, 5H), 7.21 – 7.13 (m, 1H), 6.89 – 6.81 (m, 2H), 6.63 (dt, *J*=16.0, 1.6, 1H), 6.31 (dt, *J*=16.0, 5.7, 1H), 4.65 (dd, *J*=5.8, 1.6, 2H), 3.74 (s, 2H), 3.70 (s, 3H), 3.65 (s, 3H). ^{13}C NMR (126 MHz, $CDCl_3$) δ 167.2, 166.9, 164.7, 157.2, 139.0, 136.3, 133.1, 132.2, 129.0, 128.6, 128.0, 126.6, 125.5, 123.9, 122.6, 120.9, 112.5, 69.3, 69.2, 54.5, 52.5, 52.5, 52.5, 41.1. LRMS (ESI): Mass calcd for $C_{21}H_{20}O_5$[M+H]+: 353.1; found 353.2 HRMS (ESI): Mass calcd for $C_{21}H_{20}O_5$[M+H]+: 353.1311; found 353.1310 FTIR (neat): 2990, 2600, 2211, 1666, 1636, 1606, 1515, 1453, 1293, 1251, 1220, 1172, 1143, 1103, 993, 956, 831, 741

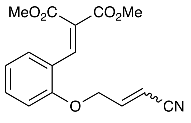

Prepared according to the general procedure with 55% overall yield over two steps. Isolated as an inseparable 1:1 mixture of Z/E isomers (4-bromobut-2-enenitrile used for SN2 reaction was an inseparable 1:1 mixture of Z/E isomers)

Analytical Data: ^{1}H NMR (500 MHz, Chloroform-*d*) δ = 7.96 (d, *J*=2.7, 1H), 7.56 – 7.46 (m, 0.5H), 7.32 – 7.19 (m, 2.5H), 6.90 – 6.81 (m, 1H), 6.84 – 6.67 (m, 1H), 5.63 (dt, *J*=16.4, 2.3, 0.5H), 5.51 – 5.44 (m, 0.5H), 5.17 (s, 0.5H), 4.88 – 4.76 (m, 0.5H), 4.62 (dd, *J*=3.7, 2.3, 1H), 3.75 – 3.59 (m, 6H). ^{13}C NMR (126 MHz, $CDCl_3$) δ 167.0, 166.9, 164.6, 164.5, 156.0, 155.8, 149.2, 148.8, 147.9, 138.8, 138.2, 134.5, 132.2, 132.1, 129.6, 129.4, 129.3, 126.4, 126.1, 124.9, 122.8, 122.0, 121.7, 116.9, 116.6, 114.7, 112.1, 112.0, 101.8, 101.2, 66.8, 66.5, 52.8, 52.7, 52.6, 52.5, 41.1. LRMS (ESI): Mass calcd for $C_{16}H_{15}NO_5$ [M+H]+: 302.1; found 302.2 HRMS (ESI): Mass calcd for $C_{16}H_{15}NO_5$ [M+H]+: 302.0950; found 302.0951 FTIR (neat): 2975, 2818, 2044, 1670, 1638, 1625, 1592, 1373, 1297, 1270, 1197, 1181, 1135, 1114, 1019, 924, 886, 745

Preparation of Salicylaldehyde Derived Arylidene Malonates Requiring Metathesis Reactions

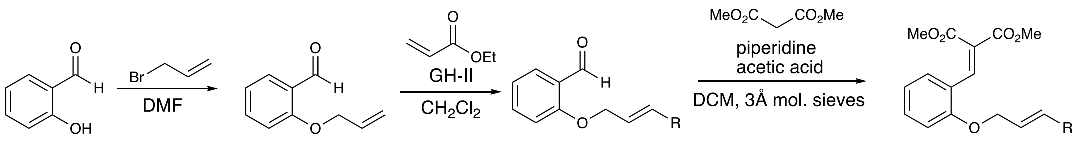

To an oven-dried scintillation vial under nitrogen was added NaH (60 wt %, 1.2 equiv) and DMF (0.5 M), and the mixture was cooled to 0 ºC. A solution of salicylaldehyde (1 equiv) dissolved in DMF (1.0 M) was slowly added, and upon addition completion, the mixture stirred at 0 ºC for 30 minutes. A solution of allyl bromide electrophile (1.2 equiv) dissolved in DMF (1.0 M) was slowly added and then reaction was allowed to stir overnight as it warmed to room temperature. Upon reaction completion, sat. aq. NH_4Cl was added, and the aqueous layer was extracted 3x with EtOAc. The combined organic layers were washed with H_2O and sat. aq. NaCl, passed through a Biotage Isolute phase separator, and concentrated to dryness under reduced pressure on a rotary evaporator to obtain the crude SN2 product, which was directly used in the next reaction without purification.

A flame dried round bottom flask was charged with alkene starting material (1.0 equiv) in CH_2Cl_2 (0.5 M), ethyl acrylate (5.0 equiv) and flushed with Ar. Grubbs-Hoveyeda second generation catalyst (2.5 mol %) was added in one portion and the reaction was stirred at room temperature under an atmosphere of Ar. The homogeneous solution was allowed to stir for 6 hours. Once the reaction was complete, the reaction was concentrated to dryness under reduced pressure on a rotary evaporator. The crude product was directly used in the next reaction without purification

To a scintillation vial was added the crude aldehyde from the previous step (1 equiv). Malonate or ketoester was added (1.1 equiv), along with 250 wt % activated 4 Å molecular sieves (powder). A magnetic stir bar and CH_2Cl_2 (2.0 M) were added, followed by acetic acid (0.1 equiv) and piperidine (0.1 equiv). The mixture was stirred overnight. Silica was added, and the reaction concentrated under

reduced pressure. The resulting mixture was loaded onto a column of silica and purified via flash column chromatography (10-20% EtOAc/hexanes) to yield the desired product.

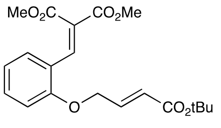

Prepared according to the general procedure with 65% overall yield over three steps.

Analytical Data: ^{1}H NMR (500 MHz, Chloroform-*d*) δ = 8.05 (s, 1H), 7.28 – 7.15 (m, 3H), 6.90 – 6.80 (m, 1H), 6.77 – 6.71 (m, 1H), 5.95 (dt, *J*=15.7, 2.0, 1H), 4.63 (dd, *J*=4.3, 2.0, 2H), 3.66 (d, *J*=18.0, 6H), 1.39 (s, 9H). ^{13}C NMR (126 MHz, $CDCl_3$) δ 167.1, 165.2, 164.6, 156.5, 140.5, 138.6, 132.1, 130.2, 129.0, 128.7, 128.2, 124.3, 121.3, 112.1, 80.8, 67.1, 60.4, 52.5, 41.1, 28.1. LRMS (ESI): Mass calcd for $C_{20}H_{24}O_7$[M+H]+: 377.2; found 377.2 HRMS (ESI): Mass calcd for $C_{20}H_{24}O_7$[M+H]+: 377.1522; found 377.1520 FTIR (neat): 2982, 2944, 1952, 1669, 1647, 1607, 1595, 1384, 1276, 1244, 1197, 1180, 1138, 1066, 992, 950, 893, 688

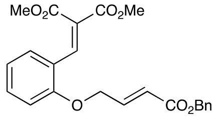

Prepared according to the general procedure with 52% overall yield over three steps.

Analytical Data: ^{1}H NMR (500 MHz, Chloroform-*d*) δ = 8.01 (s, 1H), 7.28 – 7.15 (m, 7H), 6.98 (dt, *J*=15.7, 4.1, 1H), 6.81 (t, *J*=7.6, 1H), 6.70 (d, *J*=8.1, 1H), 6.07 (dt, *J*=15.8, 2.0, 1H), 5.07 (s, 2H), 4.63 (dd, *J*=4.2, 2.1, 2H), 3.66 (d, *J*=23.6, 6H). ^{13}C NMR (126 MHz, $CDCl_3$) δ 167.1, 165.7, 164.6, 156.4, 142.3, 138.5, 135.8, 132.1, 129.1, 128.6, 128.3, 125.9, 122.7, 122.1, 121.4, 112.1, 67.0, 66.5, 52.6, 52.5. LRMS (ESI): Mass calcd for $C_{23}H_{22}O_7$[M+H]+: 411.1; found 411.1 HRMS (ESI): Mass calcd for $C_{23}H_{22}O_7$[M+H]+: 411.1365; found 411.1367 FTIR (neat): 2964, 2533, 2087, 1691, 1640, 1607, 1571, 1384, 1297, 1230, 1192, 1186, 1123, 1027, 992, 987, 882, 765

Preparation of Salicylaldehyde Derived Alkyne Electrophiles

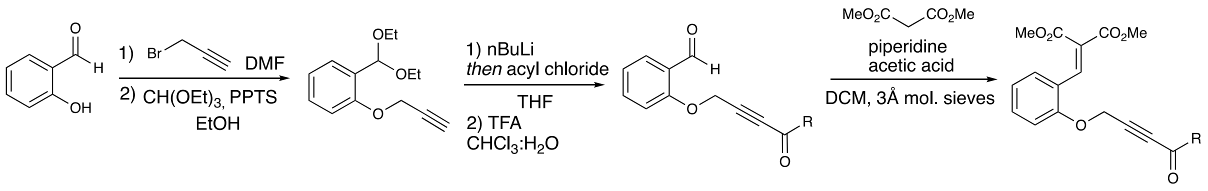

To an oven- dried scintillation vial under nitrogen was added NaH (60 wt %, 1.2 equiv) and DMF (0.5 M), and the mixture was cooled to 0 ºC. A solution of salicylaldehyde (1 equiv) dissolved in DMF (1.0 M) was slowly added, and upon addition completion, the mixture stirred at 0 ºC for 30 minutes. A solution of propargyl bromide (1.2 equiv) dissolved in DMF (1.0 M) was slowly added and then reaction was allowed to stir overnight as it warmed to room temperature. Upon reaction completion, sat. aq. NH_4Cl was added, and the aqueous layer was extracted 3x with EtOAc. The combined organic layers were washed with H_2O and sat. aq. NaCl, passed through a Biotage Isolute phase separator, and concentrated to dryness under reduced pressure on a rotary evaporator to obtain the crude SN2 product, which was directly used in the next reaction without purification.

In an oven dried round-bottom flask, salicylaldehyde propargyl ether (1 equiv) was dissolved in dry ethanol (0.2 M) under N_2 atmosphere. Triethyl orthoformate (1.7 equiv) and PPTS (1 mol %) were added, and the resulting solution was refluxed for 3 h. Upon reaction completion, the reaction mixture was quenched with few drops of Et_3N and concentrated to dryness under reduced pressure on a rotary evaporator. The oil was diluted in EtOAc (50 mL), washed with 10 % $NaHCO_3$ (2 × 20 mL), followed by saturated NaCl (2 × 10 mL) and then dried over Na_2SO_4. The combined organic layers were concentrated to dryness under reduced pressure on a rotary evaporator. The crude product was directly used in the next reaction without purification.

In an oven dried round-bottom flask, the acetal-protected salicylaldehyde (1.0 equiv) was dissolved in dry THF (0.2 M). The solution was stirred at 78 °C, and *n*-BuLi (1.1 equiv, 2.5 M in hexanes) was slowly added to the flask over 10 min, and the reaction was stirred for another 30 min. At the same temperature, acyl chloride (1.7 equiv) dissolved in THF (0.2 M) was slowly added to the reaction mixture and stirred for an additional 2 h. Upon reaction completion, the reaction mixture was allowed to warm to room temperature and quenched with sat. aq. NH_4Cl (20 mL). Then the reaction mixture was diluted with EtOAc (50 mL), washed with water (3 × 50 mL), followed by saturated NaCl (2 × 10 mL), and then dried over Na_2SO_4. The combined organic layers were concentrated to dryness under reduced pressure on a rotary evaporator. The crude product was directly used in the next reaction without purification.

To a scintillation vial with the crude acetal protected alkyne was added $CHCl_3$:H_2O (3:1), and the reaction was stirred until the alkyne completely dissolved. Trifluoroacetic acid (5.0 equiv) was added, and the reaction was stirred at room temperature for 3 h. Saturated $NaHCO_3$ was added, and the aqueous layer was extracted with CH_2Cl_2. The combined organic layers were passed through a Biotage

Isolute phase separator and concentrated to dryness under reduced pressure on a rotary evaporator to obtain the crude product, which was directly used in the next reaction without purification.

To a scintillation vial was added the crude aldehyde from the previous step (1 equiv). Malonate or ketoester was added (1.1 equiv), along with 250 wt % activated 4 Å molecular sieves (powder). A magnetic stir bar and CH_2Cl_2 (2.0 M) were added, followed by acetic acid (0.1 equiv) and piperidine (0.1 equiv). The mixture was stirred overnight. Silica was added and the reaction concentrated under reduced pressure. The resulting mixture was loaded onto a column of silica and purified via flash column chromatography (10-20% EtOAc/hexanes) to yield the desired product.

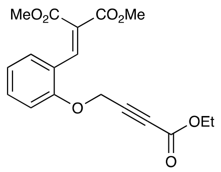

Prepared according to the general procedure with 61% overall yield over five steps.

Analytical Data: ^{1}H NMR (500 MHz, Chloroform-*d*) δ = 7.95 (d, *J*=15.6, 1H), 7.24 (pd, *J*=8.7, 8.3, 3.7, 2H), 6.96 – 6.84 (m, 1H), 6.88 – 6.79 (m, 1H), 4.74 (s, 2H), 4.16 – 3.97 (m, 2H), 3.71 (d, *J*=4.1, 3H), 3.65 (s, 3H), 1.20 – 1.08 (m, 3H). ^{13}C NMR (126 MHz, $CDCl_3$) δ 167.0, 164.6, 155.6, 152.8, 138.5, 131.9, 129.3, 126.2, 123.0, 122.0, 112.4, 80.9, 79.1, 62.4, 55.8, 52.6, 28.6, 14.0. LRMS (ESI): Mass calcd for $C_{18}H_{18}O_7$[M+H]+: 347.1; found 347.1 HRMS (ESI): Mass calcd for $C_{18}H_{18}O_7$[M+H]+: 347.1053; found 347.1051 FTIR (neat): 2980, 2888, 2311, 1668, 1643, 1605, 1566, 1417, 1287, 1269, 1214, 1164, 1130, 1028, 1001, 977, 853, 774

Preparation of Salicylaldehyde Derived Alkyne-Aryl Arylidene Malonates

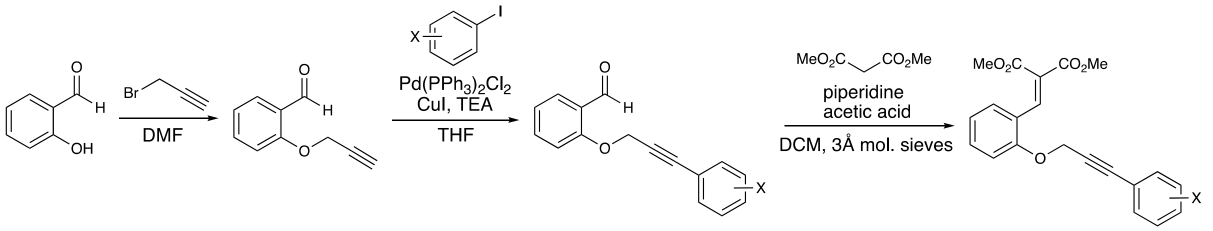

To an oven dried scintillation vial under nitrogen was added NaH (60 wt %, 1.2 equiv) and DMF (0.5 M), and the mixture was cooled to 0 ºC. A solution of salicylaldehyde (1 equiv) dissolved in DMF (1.0 M) was slowly added, and upon addition completion, the mixture stirred at 0 ºC for 30 minutes. A solution of propargyl bromide (1.2 equiv) dissolved in DMF (1.0 M) was slowly added and then reaction was allowed to stir overnight as it warmed to room temperature. Upon reaction completion, sat. aq. NH_4Cl was added, and the aqueous layer was extracted 3x with EtOAc. Upon reaction completion, sat. aq. NH_4Cl was added, and the aqueous layer was extracted 3x with EtOAc. The combined organic layers were washed with H_2O and sat. aq. NaCl, passed through a Biotage Isolute phase separator, and concentrated to dryness under reduced pressure on a rotary evaporator to obtain the crude SN2 product, which was directly used in the next reaction without purification.

To a scintillation vial was added the crude 2-prop-2-ynyloxy-benzaldehyde derivatives (1.0 equiv) with substituted iodobenzene (1.2 equiv), $Pd(PPh_3)_2Cl_2$ (2 mol %), CuI (4 mol %) and triethylamine (1.5 equiv) in dry THF (0.2 M). The reaction was stirred for 18 hours until reaction completion NH_4Cl was added, and the aqueous layer was extracted 3x with EtOAc. The combined organic layers were washed with H_2O and sat. aq. NaCl, passed through a Biotage Isolute phase separator, and concentrated to dryness under reduced pressure on a rotary evaporator to obtain the crude product, which was directly used in the next reaction without purification.

To a scintillation vial was added the crude aldehyde from the previous step (1 equiv). Malonate or ketoester was added (1.1 equiv) as well as 250 wt % activated 4 Å molecular sieves (powder). A magnetic stirbar and CH_2Cl_2 (2.0 M) were added, followed by acetic acid (0.1 equiv) and piperidine (0.1 equiv). The mixture was stirred overnight. Silica was added and the reaction concentrated under reduced pressure. The resulting mixture was loaded onto a column of silica and purified via flash column chromatography (10-20% EtOAc/hexanes) to yield the desired product.

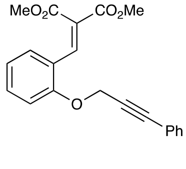

Prepared according to the general procedure with 70% overall yield over three steps.

Analytical Data: ^{1}H NMR (500 MHz, Chloroform-*d*) δ 8.08 (s, 1H), 7.39 – 7.26 (m, 4H), 7.29 – 7.17 (m, 3H), 7.08 (dd, *J* = 8.4, 1.0 Hz, 1H), 6.90 (td, *J* = 7.6, 1.0 Hz, 1H), 4.90 (s, 2H), 3.70 (d, *J* = 19.1 Hz, 6H). ^{13}C NMR (126 MHz, $CDCl_3$) δ 167.2, 166.9, 164.7, 156.3, 138.9, 132.0, 131.8, 131.6, 129.2, 128.8, 128.3, 125.7, 122.9, 122.1, 121.4, 112.8, 87.7, 83.4, 57.1, 52.6, 41.1. LRMS (ESI): Mass calcd for $C_{21}H_{18}O_5$[M+H]+: 351.1; found 351.1 HRMS (ESI): Mass calcd for $C_{21}H_{18}O_5$ [M+H]+: 351.1154; found 351.1153 FTIR (neat): 2972, 2382, 2316, 1708, 1637, 1599, 1489, 1355, 1277, 1234, 1212, 1182, 1134, 1060, 1008, 976, 900, 798

Preparation of Tetrahydroquinoline-Precursor Arylidene Malonate

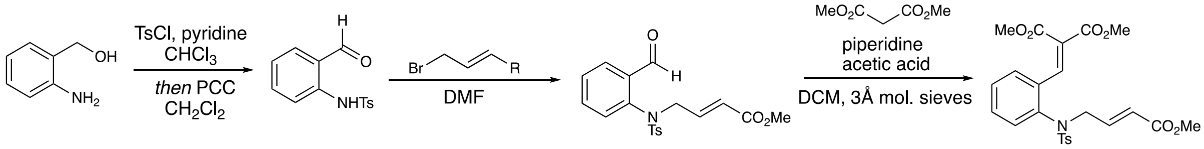

To a dry and N_2-flushed round bottom flask, equipped with a magnetic stirring bar and a septum, was charged with a solution of 2-aminobenzyl alcohol (1.0 equiv) in $CHCl_3$ (0.2 M). TsCl (1.1 equiv) and pyridine (5 mol %) were added, and the reaction mixture was stirred for 12 h at room temperature. Once the reaction was complete, the reaction was concentrated to dryness under reduced pressure on a rotary evaporator. Without purification, the crude product was dissolved in CH_2Cl_2 (0.5 M) and PCC (1.2 equiv) was added. The reaction mixture was stirred for 4 h at room temperature and then filtered through celite followed by washing with CH_2Cl_2. The combined organic layers were concentrated to dryness under reduced pressure on a rotary evaporator. The crude product was directly used in the next reaction without purification.

To an oven dried scintillation vial under nitrogen was added NaH (60 wt %, 1.2 equiv) and DMF (0.5 M), and the mixture was cooled to 0 ºC. A solution of N-tosyl-aldehyde (1 equiv) dissolved in DMF (1.0 M) was slowly added, and upon addition completion, the mixture stirred at 0 ºC for 30 minutes. A solution of allyl bromide (1.2 equiv) dissolved in DMF (1.0 M) was slowly added and then reaction was allowed to stir overnight as it warmed to room temperature. Upon reaction completion, sat. aq. NH_4Cl was added, and the aqueous layer was extracted 3x with EtOAc. The combined organic layers were washed with H_2O and saturated NaCl followed by passage through a Biotage Isolute phase separator and concentration concentrated to dryness under reduced pressure on a rotary evaporator to obtain the crude SN2 product, which was directly used in the next reaction without purification.

To a scintillation vial was added the crude aldehyde from the previous step (1 equiv). Malonate or ketoester was added (1.1 equiv), as well as 250 wt % activated 4 Å molecular sieves (powder). A magnetic stirbar and mL of CH_2Cl_2 (2.0 M) were added, followed by acetic acid (0.1 equiv) and piperidine (0.1 equiv). The mixture was stirred overnight. Silica was added and the reaction

concentrated under reduced pressure. The resulting mixture was loaded onto a column of silica and purified via flash column chromatography (10-20% EtOAc/hexanes) to yield the desired product.

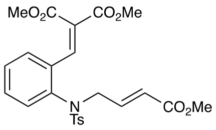

Prepared according to the general procedure with 62% overall yield over four steps.

Analytical Data: ^{1}H NMR (500 MHz, Chloroform-*d*) δ = 7.64 (s, 1H), 7.57 – 7.48 (m, 2H), 7.32 – 7.20 (m, 4H), 7.02 – 6.93 (m, 1H), 6.72 (dt, *J*=15.7, 6.6, 1H), 5.73 (dt, *J*=15.7, 1.5, 1H), 5.24 (s, 1H), 4.20 (dd, *J*=6.6, 1.4, 2H), 3.78 (s, 3H), 3.69 (s, 1H), 3.61 (d, *J*=3.8, 5H), 2.38 (s, 3H). ^{13}C NMR (126 MHz, $CDCl_3$) δ 166.2, 165.8, 163.7, 147.1, 144.2, 141.3, 140.1, 138.1, 135.8, 134.4, 130.9, 130.5, 129.8, 129.0, 128.3, 127.8, 124.6, 119.7, 61.8, 52.9, 52.5, 51.7, 21.6. LRMS (ESI): Mass calcd for $C_{24}H_{25}NO_8S$ [M+H]+: 487.1; found 487.2 HRMS (ESI): Mass calcd for $C_{24}H_{25}NO_8S$ [M+H]+: 487.1309; found 487.1311 FTIR (neat): 2977, 2957, 2293, 1671, 1644, 1602, 1488, 1342, 1295, 1265, 1195, 1172, 1143, 1067, 1012, 956, 845, 764

Preparation of Tetrahydronapthalene-Precursor Arylidene Malonate

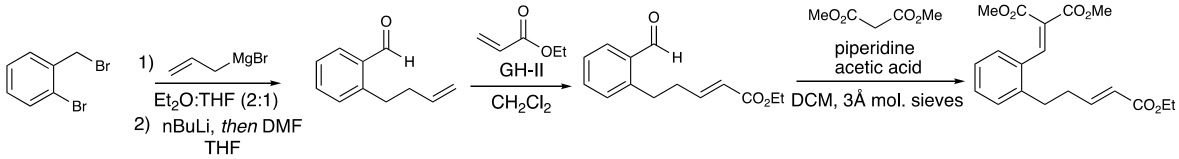

To a round-bottom flask flushed with N_2 was charged Mg turnings (5.0 equiv) and anhydrous ether (0.5 M). A tip of iodine and a drop of 1,2-dibromoethane were successively added. After leaving the mixture at 0 °C for 30 min, a solution of allyl bromide (2.0 equiv) in dry ether (0.5 M) was added dropwise to the mixture to prepare a solution of a Grignard reagent. To a solution of 2-bromobenzyl bromide (1.0 equiv) in anhydrous THF (0.5 M) was added dropwise the prepared solution of a Grignard reagent at rt, and the reaction mixture was stirred at rt overnight. The following day, 25 mL of H_2O was added to the mixture, which was then extracted with ether (15 mL × 3). The combined ethereal solution was washed with H_2O (15 mL × 3) and dried over anhydrous $MgSO_4$. The reaction was concentrated to dryness under reduced pressure on a rotary evaporator. The crude product was directly used in the next reaction without purification.

Under N_2, to a solution of 1-bromo or iodo aryl compound 1.0 equiv) in anhydrous diethyl ether (0.25 M) at –78 °C was slowly added *n*-BuLi (1.1 equiv, 2.5 M in hexanes). The reaction was stirred at the same temperature for 40 min, and DMF (3.0 equiv) was added dropwise. The reaction was allowed to warm to room temperature over 1 h before it was quenched with saturated aqueous NH_4Cl. The reaction mixture was diluted with diethyl ether (30 mL), washed with saturated NH_4Cl (10 mL)

and saturated NaCl (10 mL), dried over Na_2SO_4, and concentrated to dryness under reduced pressure on a rotary evaporator. The crude product was directly used in the next reaction without purification.

A flame dried round bottom flask was charged with alkene starting material (1.0 equiv) in CH_2Cl_2 (0.5 M), ethyl acrylate (5.0 equiv) and flushed with Ar. Grubbs-Hoveyeda second generation catalyst (2.5 mol %) was added in one portion and the reaction was stirred at room temperature under an atmosphere of Ar. The homogeneous solution was stirred for 6 hours. Once the reaction was complete, the reaction was concentrated to dryness under reduced pressure on a rotary evaporator. The crude product was directly used in the next reaction without purification.

To a scintillation vial was added the crude aldehyde from the previous step (1 equiv). Malonate or ketoester was added (1.1 equiv), as well as 250 wt % activated 4 Å molecular sieves (powder). A magnetic stirbar and CH_2Cl_2 (2.0 M) were added, followed by acetic acid (0.1 equiv) and piperidine (0.1 equiv). The mixture was stirred overnight. Silica was added and the reaction concentrated under reduced pressure. The resulting mixture was loaded onto a column of silica and purified via flash column chromatography (10-20% EtOAc/hexanes) to yield the desired product.

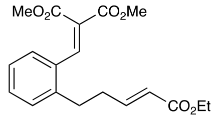

Prepared according to the general procedure with 58% overall yield over four steps. Isolated as a ~2:1 mixture of E/Z isomers

Analytical Data: ^{1}H NMR (500 MHz, Chloroform-*d*) δ = 7.99 (s, 1H), 7.33 – 7.24 (m, 2H), 7.23 – 7.13 (m, 2H), 6.93 (dt, *J*=15.7, 6.9, 1H), 5.79 (dt, *J*=15.7, 1.6, 1H), 4.15 (q, *J*=7.1, 2H), 3.85 (s, 3H), 3.70 (d, *J*=31.0, 3H), 2.82 (dd, *J*=8.8, 6.8, 2H), 2.49 – 2.40 (m, 2H), 1.26 (t, *J*=7.1, 3H). ^{13}C NMR (126 MHz, $CDCl_3$) δ 166.5, 166.4, 164.2, 147.2, 142.2, 140.2, 132.4, 130.3, 129.6, 128.1, 126.7, 122.3, 60.2, 52.7, 52.5, 41.1, 33.2, 32.2, 14.3. LRMS (ESI): Mass calcd for $C_{19}H_{22}O_6$[M+H]+: 347.1; found 347.1 HRMS (ESI): Mass calcd for $C_{19}H_{22}O_6$[M+H]+: 347.1416; found 347.1418 FTIR (neat): 2997, 2710, 2244, 1663, 1637, 1613, 1562, 1374, 1283, 1254, 1206, 1181, 1146, 1104, 1021, 939, 865, 728

Preparation of Dihydrobenzofuran-Precursor Arylidene Malonate

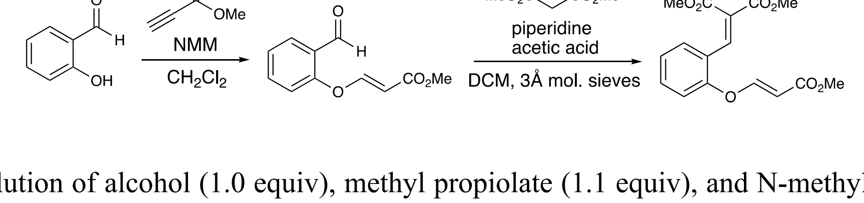

A solution of alcohol (1.0 equiv), methyl propiolate (1.1 equiv), and N-methylmorpholine (5 mol %) in CH_2Cl_2 (0.4 M) was stirred at room temperature for 4 h. The solution was then washed with water and saturated aqueous NaCl and dried over anhydrous sodium sulfate. The combined organic

layers were concentrated to dryness under reduced pressure on a rotary evaporator. The crude product was directly used in the next reaction without purification.

To a scintillation vial was added the crude aldehyde from the previous step (1 equiv). Malonate or ketoester was added (1.1 equiv) along with 250 wt % activated 4 Å molecular sieves (powder). A magnetic stir bar and CH_2Cl_2 (2.0 M) were added, followed by acetic acid (0.1 equiv) and piperidine (0.1 equiv). The mixture was stirred overnight. Silica was added and the crude reaction mixture was concentrated under reduced pressure. The resulting mixture was loaded onto a column of silica and purified via flash column chromatography (10-20% EtOAc/hexanes) to yield the desired product.

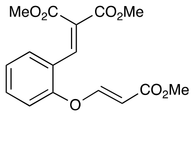

Prepared according to the general procedure with 85% overall yield over two steps.

Analytical Data: ^{1}H NMR (500 MHz, Chloroform-*d*) δ = 8.07 (s, 1H), 7.90 (d, *J*=12.3, 1H), 7.62 – 7.50 (m, 2H), 7.37 – 7.29 (m, 1H), 7.23 (dd, *J*=8.1, 1.1, 1H), 5.71 (d, *J*=12.2, 1H), 4.00 (s, 3H), 3.90 (d, *J*=24.6, 6H). ^{13}C NMR (126 MHz, $CDCl_3$) δ 167.1, 166.4, 164.2, 158.3, 153.9, 137.2, 132.1, 129.4, 127.7, 125.3, 124.5, 118.0, 103.2, 52.8, 51.4, 50.4. LRMS (ESI): Mass calcd for $C_{16}H_{16}O_7$[M+H]+: 321.1; found 321.1 HRMS (ESI): Mass calcd for $C_{16}H_{16}O_7$[M+H]+: 321.0896; found 321.0899 FTIR (neat): 2994, 2495, 2353, 1669, 1633, 1605, 1531, 1484, 1281, 1226, 1208, 1168, 1122, 1094, 994, 983, 868, 743

Selected Optimization Data:

Table 1. Effect of Solvent

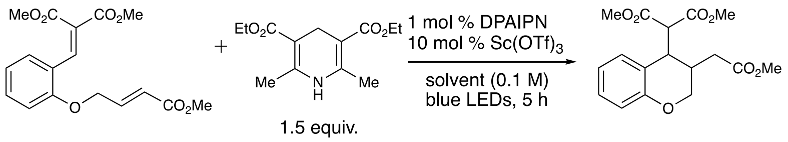

Entry:	Conditions:	GC Yield:
1	CH_3CN	84
2	DMF	38
3	MeOH	<5
4	DMSO	45
5	DMPU	38
6	CH_2Cl_2	<5
7	THF	<5

[a] Yield determined by GC with bibenzyl as internal standard.

Table 2. Chiral Ligand Screen

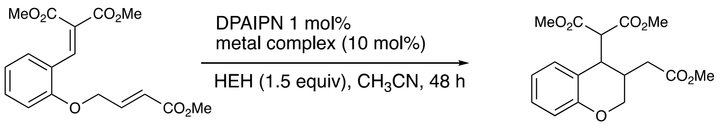

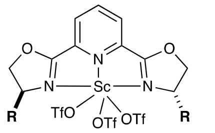

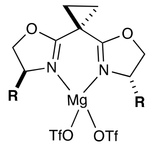

entry	R	yield*	dr	er
1	Ph	53	1.5:1	50:50
2	Inda	51	2:1	50:50
3	*i*Pr	50	2.5:1	50:50
4	Bn	10	1.5:1	50:50

entry	R	yield	dr	er
5	Inda	52	3:1	50:50
6	*t*Bu	56	2.5:1	50:50

**all reactions showed incomplete conversion*

[a] Yield determined by GC with bibenzyl as internal standard.
[b] dr determined by ^{1}H NMR
[c] er determined by SFC on a chiral stationary phase

Table 3. Effect of Concentration and Catalyst Loading

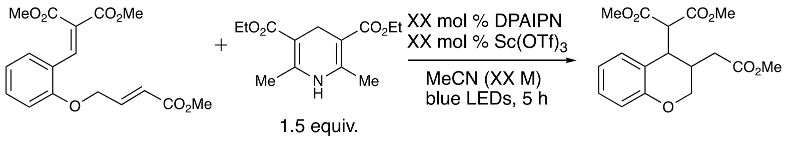

Entry:	Conditions:	GC Yield:
1	10% $Sc(OTf)_3$, 0.5% DPAIPN, 0.2 M	62
2	5% $Sc(OTf)_3$, 0.5% DPAIPN, 0.2 M	51
3	5% $Sc(OTf)_3$, 1% DPAIPN, 0.2 M	65
4	10% $Sc(OTf)_3$, 1% DPAIPN, 0.2 M	81
5	10% $Sc(OTf)_3$, 0.5% DPAIPN, 0.1 M	65
6	5% $Sc(OTf)_3$, 0.5% DPAIPN, 0.1 M	55
7	10% $Sc(OTf)_3$, 0.5% DPAIPN, 0.05 M	42
8	5% $Sc(OTf)_3$, 1% DPAIPN, 0.05 M	41

[a] Yield determined by GC with bibenzyl as internal standard.

General Procedure for Reductive Cyclization

To a 2 dram vial was added arylidene malonate (1.0 equiv). The reaction vessel was equipped with a cap and stir bar and was then taken into a glovebox. DPAIPN (1 mol %) and $Sc(OTf)_3$ (10 mol %) were added to the vial, which was then removed from the glovebox. The vial was then charged with a solution of HEH (1.5 equiv) and sparged CH3CN (0.1 M). The mixture was stirred until homogenous. The vial was then placed between 3 Kessil blue LED lights and irradiated for 5 hours (with a small fan placed for cooling). Conversion of the malonate was monitored by UPLC/MS. Upon complete conversion, the reaction was concentrated under reduced pressure onto silica gel. This silica was loaded onto a column of silica gel and isolated via flash column chromatography (2-20% ethyl acetate/hexanes) to yield the product as a mixture of diastereomers.

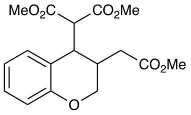

Prepared according to the general procedure in 86% yield in a 1.2:1 ratio of diastereomers
^{1}H NMR (500 MHz, Chloroform-*d*) δ = 7.11 (qd, *J*=8.1, 1.7, 1H), 7.04 – 6.93 (m, 1H), 6.93 – 6.74 (m, 2H), 4.27 – 4.15 (m, 1H), 3.95 – 3.83 (m, 1H), 3.77 (d, *J*=3.8, 3H), 3.74 – 3.53 (m, 6H), 3.46 – 3.35 (m, 2H), 2.69 (dddd, *J*=13.1, 9.0, 6.2, 3.6, 1H), 2.44 (dq, *J*=13.6, 7.1, 1H), 2.41 – 2.26 (m, 2H), 2.28 – 2.13 (m, 1H). ^{13}C NMR (126 MHz, $CDCl_3$) δ 172.3, 171.7, 168.9, 168.5, 168.2, 168.0, 153.5, 130.6, 129.0, 128.9, 128.8, 121.2, 120.5, 120.1, 118.9, 117.0, 116.5, 66.5, 64.2, 58.8, 54.1, 53.0, 52.9, 52.6, 52.5, 51.9, 51.8, 38.8, 37.5, 35.4, 32.1, 32.1, 31.5. LRMS (ESI): Mass calcd for $C_{17}H_{20}O_7$[M+H]+: 337.1; found 337.1 HRMS (ESI): Mass calcd for $C_{17}H_{20}O_7$[M+H]+: 337.1209; found 337.1211 FTIR (neat): 3090, 2816, 2624, 1887, 1670, 1642, 1628, 1495, 1335, 1304, 1247, 1220, 1168, 1138, 1103, 998, 987, 860, 709

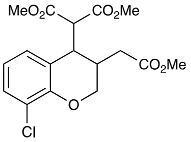

Prepared according to the general procedure in 61% yield in a 1.2:1 ratio of diastereomers
^{1}H NMR (500 MHz, Chloroform-*d*) δ = 7.22 (td, *J*=9.1, 8.5, 1.6, 1H), 6.93 (dd, *J*=7.8, 1.5, 1H), 6.73 (q, *J*=7.6, 1H), 4.40 – 4.31 (m, 1H), 4.26 (dd, *J*=12.4, 2.1, 1H), 3.96 – 3.89 (m, 1H), 3.78 (s, 1H), 3.77 (s, 2H), 3.68 (s, 1H), 3.67 (s, 2H), 3.67 – 3.54 (m, 1H), 3.55 (s, 2H), 3.44 (d, *J*=16.6, 2H), 2.48 – 2.23 (m, 3H) ^{13}C NMR (126 MHz, $CDCl_3$) δ 172.0, 171.4, 168.8, 168.3, 168.0, 167.7, 149.5, 149.3, 129.7, 129.5, 129.2, 127.6, 123.1, 121.6, 121.4, 120.7, 120.6, 120.1, 67.0, 65.2, 58.6, 53.9, 53.1, 53.0, 52.6, 52.6, 52.0, 51.9, 38.8, 37.4, 35.3, 32.1, 32.0, 31.3. LRMS (ESI): Mass calcd for $C_{17}H_{19}ClO_7$ [M+H]+: 371.1; found 371.1 HRMS (ESI): Mass calcd for $C_{17}H_{19}ClO_7$ [M+H]+: 371.0819; found 371.0821

FTIR (neat): 3090, 2877, 2475, 1734, 1664, 1658, 1622, 1499, 1473, 1304, 1254, 1211, 1176, 1128, 1076, 1016, 985, 849, 825

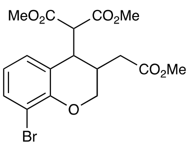

Prepared according to the general procedure in 70% yield in a 1.2:1 ratio of diastereomers
^{1}H NMR (500 MHz, Chloroform-*d*) δ = 7.38 (ddd, *J*=9.7, 7.8, 1.6, 1H), 6.97 (dd, *J*=7.8, 1.5, 1H), 6.67 (q, *J*=7.7, 1H), 4.39 – 4.30 (m, 1H), 4.26 (dd, *J*=12.0, 2.2, 1H), 4.20 – 4.05 (m, 1H), 3.92 (dd, *J*=8.0, 4.1, 1H), 3.77 (s, 2H), 3.67 (s, 1H), 3.66 (s, 2H), 3.64 – 3.51 (m, 1H), 3.55 (s, 2H), 3.48 – 3.32 (m, 2H), 2.77 – 2.66 (m, 1H), 2.48 – 2.32 (m, 2H), 2.31 – 2.14 (m, 1H) ^{13}C NMR (126 MHz, $CDCl_3$) δ 172.0, 171.4, 168.8, 168.2, 168.0, 167.7, 150.1, 132.8, 132.6, 129.9, 128.4, 123.0, 121.2, 120.7, 120.7, 110.9, 110.6, 67.1, 65.4, 58.6, 53.8, 53.1, 53.0, 52.6, 52.6, 52.0, 51.9, 39.0, 37.5, 35.3, 32.1, 32.1, 31.3, 14.2. LRMS (ESI): Mass calcd for $C_{17}H_{19}BrO_7$ [M+H]+: 415.1; found 415.2 HRMS (ESI): Mass calcd for $C_{17}H_{19}BrO_7$ [M+H]+: 415.0314; found 415.0316 FTIR (neat): 3090, 2964, 2557, 2069, 1708, 1647, 1606, 1550, 1354, 1292, 1229, 1189, 1168, 1122, 1048, 1016, 978, 900, 778

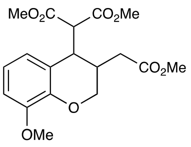

Prepared according to the general procedure in 62% yield in a 1.2:1 ratio of diastereomers
^{1}H NMR (500 MHz, Chloroform-*d*) δ = 6.87 – 6.73 (m, 1H), 6.77 – 6.65 (m, 1H), 6.61 (dd, *J*=6.5, 2.9, 1H), 4.37 – 4.19 (m, 1H), 4.21 – 4.06 (m, 1H), 3.96 – 3.80 (m, 3H), 3.76 (s, 2H), 3.84 – 3.67 (m, 2H), 3.65 (s, 2H), 3.70 – 3.59 (m, 1H), 3.56 (s, 2H), 3.62 – 3.46 (m, 1H), 3.48 – 3.32 (m, 2H), 2.80 – 2.55 (m, 1H), 2.47 (dt, *J*=15.9, 7.9, 1H), 2.40 – 2.27 (m, 1H) ^{13}C NMR (126 MHz, $CDCl_3$) δ 172.2, 168.2, 167.9, 148.1, 142.9, 122.3, 120.7, 120.0, 119.6, 119.5, 117.6, 110.4, 110.4, 66.9, 64.6, 58.9, 58.8, 56.0, 55.9, 55.8, 54.2, 53.0, 53.0, 52.9, 52.6, 52.5, 51.9, 51.8, 50.9, 39.1, 38.6, 37.4, 35.2, 32.0, 31.9, 31.3. LRMS (ESI): Mass calcd for $C_{18}H_{22}O_8$ [M+H]+: 367.1; found 367.1 HRMS (ESI): Mass calcd for $C_{18}H_{22}O_8$ [M+H]+: 367.1315; found 367.1317 FTIR (neat): 3028, 2893, 2704, 2196, 1693, 1631, 1600, 1531, 1428, 1273, 1269, 1218, 1165, 1128, 1091, 995, 938, 896, 696

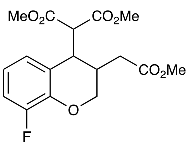

Prepared according to the general procedure in 74% yield in a 1.5:1 ratio of diastereomers

^{1}H NMR (500 MHz, Chloroform-*d*) δ = 7.00 – 6.88 (m, 1H), 6.84 – 6.66 (m, 2H), 4.35 – 4.27 (m, 1H), 4.25 – 4.10 (m, 1H), 3.78 (s, 1H), 3.77 (s, 2H), 3.67 (s, 2H), 3.72 – 3.60 (m, 2H), 3.59 (t, *J*=7.4, 1H), 3.56 (s, 2H), 3.51 – 3.42 (m, 2H), 2.50 – 2.27 (m, 3H) ^{13}C NMR (126 MHz, $CDCl_3$) δ 172.0, 171.5, 168.7, 168.3, 168.0, 167.7, 152.3, 150.4, 142.1, 142.0, 125.6, 125.5, 124.0, 123.9, 123.8, 121.4, 119.9, 119.8, 119.4, 119.4, 115.4, 115.2, 115.1, 66.7, 64.6, 58.6, 53.9, 53.1, 53.0, 52.7, 52.6, 52.0, 51.9, 38.3, 38.2, 37.1, 35.3, 32.0, 31.9, 31.3. LRMS (ESI): Mass calcd for $C_{17}H_{19}FO_7$ [M+H]+: 355.1; found 355.1 HRMS (ESI): Mass calcd for $C_{17}H_{19}FO_7$ [M+H]+: 355.1115; found 355.1110 FTIR (neat): 3093, 2766, 2601, 1918, 1667, 1639, 1599, 1489, 1346, 1278, 1238, 1215, 1183, 1151, 1082, 1016, 983, 854, 747

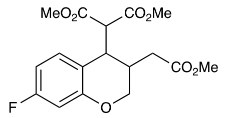

Prepared according to the general procedure in 68% yield in a 1.5:1 ratio of diastereomers
^{1}H NMR (500 MHz, Chloroform-*d*) δ = 6.93 (ddd, *J*=41.9, 8.3, 6.5, 1H), 6.57 – 6.46 (m, 2H), 4.25 – 4.15 (m, 2H), 4.12 – 4.02 (m, 1H), 3.87 (dd, *J*=7.9, 4.2, 1H), 3.77 (d, *J*=3.4, 1H), 3.77 (s, 2H), 3.77 – 3.64 (m, 4H), 3.62 – 3.51 (m, 3H), 3.44 (d, *J*=1.0, 1H), 3.39 (d, *J*=10.3, 1H), 2.74 – 2.63 (m, 1H), 2.45 – 2.24 (m, 2H) ^{13}C NMR (126 MHz, $CDCl_3$) δ 172.1, 171.5, 168.9, 168.4, 168.1, 167.8, 163.8, 161.9, 154.7, 154.6, 131.8, 131.7, 130.1, 114.7, 114.7, 108.0, 107.8, 107.3, 107.2, 104.1, 103.9, 103.5, 66.3, 64.4, 58.7, 53.9, 53.1, 52.9, 52.6, 52.6, 52.0, 51.8, 38.3, 36.9, 35.3, 32.2, 32.1, 31.4. LRMS (ESI): Mass calcd for $C_{17}H_{19}FO_7$ [M+H]+: 355.1; found 355.1 HRMS (ESI): Mass calcd for $C_{17}H_{19}FO_7$ [M+H]+: 355.1115; found 355.1114 FTIR (neat): 3052, 2886, 2485, 2252, 1708, 1656, 1626, 1544, 1331, 1286, 1255, 1208, 1186, 1121, 1109, 994, 952, 839, 719

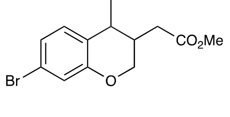

Prepared according to the general procedure in 84% yield in a 1.2:1 ratio of diastereomers
^{1}H NMR (500 MHz, Chloroform-*d*) δ = 7.02 – 6.94 (m, 1H), 6.97 – 6.84 (m, 2H), 4.16 (d, *J*=2.1, 1H), 4.07 (td, *J*=11.8, 11.1, 8.2, 1H), 3.77 (s, 2H), 3.76 (d, *J*=7.1, 1H), 3.72 – 3.52 (m, 7H), 3.46 (s, 1H), 3.37 (d, *J*=10.1, 1H), 2.45 – 2.23 (m, 3H) ^{13}C NMR (126 MHz, $CDCl_3$) δ 172.1, 171.5, 168.3, 168.0, 167.7, 154.3, 141.6, 132.4, 131.9, 130.2, 123.7, 123.1, 122.0, 121.6, 120.1, 119.7, 118.0, 114.8, 66.6,

66.5, 64.4, 58.5, 53.7, 53.0, 52.6, 51.9, 38.4, 37.1, 35.3, 32.0, 32.0, 31.3, 29.9. LRMS (ESI): Mass calcd for $C_{17}H_{19}BrO_7$ [M+H]+: 415.1; found 415.2 HRMS (ESI): Mass calcd for $C_{17}H_{19}BrO_7$ [M+H]+: 415.0314; found 415.0318 FTIR (neat): 3100, 2967, 2561, 2235, 1686, 1650, 1625, 1567, 1371, 1303, 1258, 1217, 1185, 1124, 1120, 1013, 933, 900, 802

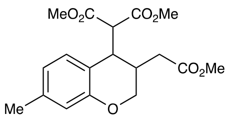

Prepared according to the general procedure in 88% yield in a 1.2:1 ratio of diastereomers
^{1}H NMR (500 MHz, Chloroform-*d*) δ = 6.91 – 6.70 (m, 1H), 6.67 – 6.56 (m, 2H), 4.23 – 4.03 (m, 2H), 3.91 – 3.80 (m, 1H), 3.76 (d, *J*=2.9, 2H), 3.74 – 3.52 (m, 5H), 3.48 (s, 1H), 3.36 (d, *J*=10.3, 1H), 2.48 – 2.34 (m, 1H), 2.37 – 2.22 (m, 2H), 2.25 – 2.13 (m, 3H) ^{13}C NMR (126 MHz, $CDCl_3$) δ 172.4, 171.8, 168.3, 168.0, 153.3, 139.1, 138.8, 130.4, 128.5, 121.6, 121.4, 121.1, 118.1, 117.3, 116.9, 115.8, 66.6, 64.1, 58.9, 54.1, 53.0, 52.8, 52.6, 52.5, 51.9, 51.8, 38.6, 37.3, 35.3, 32.2, 31.9, 31.6, 21.1. LRMS (ESI): Mass calcd for $C_{18}H_{22}O_7$ [M+H]+: 351.1; found 351.1 HRMS (ESI): Mass calcd for $C_{18}H_{22}O_7$ [M+H]+: 351.1366; found 351.1365 FTIR (neat): 3093, 2893, 2538, 1936, 1699, 1631, 1625, 1522, 1451, 1290, 1247, 1200, 1182, 1121, 1059, 997, 945, 895, 756

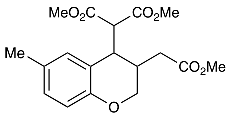

Prepared according to the general procedure in 91% yield in a 1.2:1 ratio of diastereomers
^{1}H NMR (500 MHz, Chloroform-*d*) δ = 6.91 (td, *J*=8.2, 2.1, 1H), 6.80 – 6.71 (m, 1H), 6.74 – 6.60 (m, 1H), 4.23 – 4.01 (m, 2H), 3.91 – 3.79 (m, 1H), 3.81 – 3.69 (m, 3H), 3.72 – 3.63 (m, 3H), 3.66 – 3.55 (m, 1H), 3.51 (d, *J*=56.4, 3H), 3.35 (d, *J*=10.2, 1H), 2.44 (dd, *J*=18.0, 10.2, 1H), 2.38 – 2.26 (m, 2H), 2.28 – 2.14 (m, 1H), 2.19 (s, 2H). ^{13}C NMR (126 MHz, $CDCl_3$) δ 172.4, 171.8, 169.0, 168.6, 168.3, 168.0, 151.5, 151.3, 130.8, 129.7, 129.6, 129.4, 129.2, 129.1, 120.8, 118.5, 116.7, 116.2, 66.4, 64.1, 58.9, 54.1, 53.0, 52.8, 52.5, 52.4, 51.9, 51.8, 38.9, 37.5, 35.4, 32.2, 32.1, 31.6, 20.5, 20.5. LRMS (ESI): Mass calcd for $C_{18}H_{22}O_7$ [M+H]+: 351.1; found 351.1 HRMS (ESI): Mass calcd for $C_{18}H_{22}O_7$ [M+H]+: 351.1366; found 351.1367 FTIR (neat): 3042, 2854, 2608, 2018, 1689, 1636, 1628, 1580, 1467, 1299, 1238, 1219, 1179, 1120, 1086, 1012, 951, 856, 759

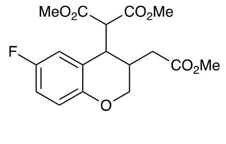

Prepared according to the general procedure in 92% yield in a 1.3:1 ratio of diastereomers
^{1}H NMR (500 MHz, Chloroform-*d*) δ = 6.93 – 6.76 (m, 1H), 6.80 – 6.70 (m, 1H), 6.68 (ddd, *J*=30.4, 8.4, 3.1, 1H), 4.23 – 4.02 (m, 2H), 3.77 (s, 2H), 3.91 – 3.73 (m, 1H), 3.75 – 3.65 (m, 2H), 3.67 (s, 2H), 3.61 (s, 2H), 3.52 (s, 1H), 3.38 (d, *J*=10.0, 1H), 2.48 – 2.25 (m, 3H) ^{13}C NMR (126 MHz, $CDCl_3$) δ 172.1, 171.6, 168.7, 168.0, 167.7, 157.6, 155.7, 149.6, 119.9, 118.0, 117.9, 117.4, 116.5, 116.1, 115.9, 115.5, 115.0, 114.8, 66.6, 64.4, 58.6, 53.8, 53.1, 52.9, 52.7, 51.8, 38.8, 37.5, 35.3, 31.88, 31.3. LRMS (ESI): Mass calcd for $C_{17}H_{19}FO_7$ [M+H]+: 355.1; found 355.1 HRMS (ESI): Mass calcd for $C_{17}H_{19}FO_7$ [M+H]+: 355.1115; found 355.1117 FTIR (neat): 3081, 2939, 2530, 2298, 1666, 1635, 1620, 1564, 1423, 1303, 1223, 1194, 1188, 1158, 1089, 1007, 928, 901, 775

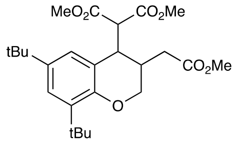

Prepared according to the general procedure in 91% yield in a 1.1:1 ratio of diastereomers
^{1}H NMR (500 MHz, Chloroform-*d*) δ = 7.14 (d, *J*=2.5, 1H), 6.85 (d, *J*=2.4, 1H), 4.22 – 4.14 (m, 1H), 4.09 (dt, *J*=11.5, 1.9, 1H), 3.95 – 3.83 (m, 1H), 3.86 – 3.73 (m, 1H), 3.76 (s, 2H), 3.76 – 3.65 (m, 2H), 3.66 (s, 2H), 3.55 (s, 2H), 3.46 (s, 1H), 3.44 – 3.33 (m, 1H), 2.45 – 2.28 (m, 2H), 1.33 (d, *J*=24.0, 1H), 1.32 (s, 8H), 1.30 – 1.18 (m, 2H), 1.22 (s, 7H). ^{13}C NMR (126 MHz, $CDCl_3$) δ 172.5, 168.3, 168.3, 150.2, 141.9, 136.8, 125.1, 123.2, 118.8, 66.3, 64.2, 59.1, 52.8, 52.5, 51.7, 39.9, 35.8, 35.0, 34.9, 34.2, 31.7, 31.6, 31.6, 31.6, 29.7. LRMS (ESI): Mass calcd for $C_{25}H_{36}O_7$ [M+H]+: 449.2; found 449.2 HRMS (ESI): Mass calcd for $C_{25}H_{36}O_7$ [M+H]+: 449.2461; found 449.2462 FTIR (neat): 3039, 2804, 2587, 1770, 1690, 1630, 1615, 1569, 1413, 1305, 1243, 1216, 1168, 1134, 1084, 1002, 956, 852, 759

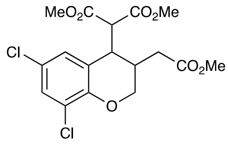

Prepared according to the general procedure in 90% yield in a 1.2:1 ratio of diastereomers
^{1}H NMR (500 MHz, Chloroform-*d*) δ = 7.26 – 7.19 (m, 1H), 6.97 – 6.93 (m, 1H), 4.34 (dt, *J*=12.1, 1.7, 1H), 4.26 – 4.04 (m, 2H), 3.77 (s, 3H), 3.67 (s, 3H), 3.61 (s, 3H), 3.68 – 3.46 (m, 2H), 3.47 – 3.38 (m, 1H), 2.45 – 2.13 (m, 3H). ^{13}C NMR (126 MHz, $CDCl_3$) δ 171.8, 167.8, 167.5, 148.1, 129.4, 129.2, 128.8, 127.5, 124.9, 122.5, 121.8, 67.0, 65.4, 58.4, 53.7, 53.2, 53.1, 52.8, 52.7, 52.1, 51.9, 38.7, 37.3, 35.2, 32.0, 31.8, 31.1. LRMS (ESI): Mass calcd for $C_{17}H_{18}Cl_2O_7$ [M+H]+: 405.1; found 405.1 HRMS

(ESI): Mass calcd for $C_{17}H_{18}Cl_2O_7$ [M+H]+: 405.0430; found 405.0428 FTIR (neat): 3097, 2839, 2540, 2264, 1682, 1658, 1622, 1496, 1398, 1304, 1254, 1218, 1165, 1128, 1033, 1001, 941, 887, 799

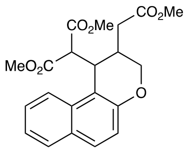

Prepared according to the general procedure in 82% yield in a 1.5:1 ratio of diastereomers
^{1}H NMR (500 MHz, Chloroform-*d*) δ = 7.83 (d, *J*=8.5, 1H), 7.78 – 7.57 (m, 2H), 7.44 (dddd, *J*=8.4, 6.6, 5.2, 1.3, 1H), 7.33 – 7.24 (m, 1H), 7.00 (dd, *J*=8.9, 3.0, 1H), 4.38 – 4.28 (m, 1H), 4.29 – 4.06 (m, 2H), 3.81 (s, 1H), 3.81 – 3.55 (m, 6H), 3.03 (s, 2H), 2.78 (s, 1H), 2.58 – 2.38 (m, 2H), 2.33 (dd, *J*=16.5, 5.8, 1H) ^{13}C NMR (126 MHz, $CDCl_3$) δ 172.4, 171.2, 169.7, 168.7, 168.3, 168.1, 151.5, 133.0, 132.3, 129.4, 129.2, 128.8, 128.6, 128.1, 126.4, 126.3, 123.3, 123.3, 122.9, 121.9, 118.7, 118.4, 110.6, 64.8, 64.2, 57.8, 54.0, 53.0, 52.8, 52.1, 52.0, 51.9, 51.8, 35.9, 34.6, 33.1, 32.9, 32.7, 32.0, 14.2. LRMS (ESI): Mass calcd for $C_{21}H_{22}O_7$ [M+H]+: 387.1; found 387.1 HRMS (ESI): Mass calcd for $C_{21}H_{22}O_7$ [M+H]+: 387.1366; found 387.1364 FTIR (neat): 3006, 2943, 2689, 2235, 1669, 1657, 1608, 1515, 1340, 1305, 1264, 1199, 1166, 1140, 1046, 1000, 987, 832, 747

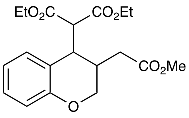

Prepared according to the general procedure in 86% yield in a 1.2:1 ratio of diastereomers
^{1}H NMR (500 MHz, Chloroform-*d*) δ = 7.20 – 6.89 (m, 2H), 6.90 – 6.73 (m, 2H), 4.35 – 4.07 (m, 5H), 4.09 – 3.96 (m, 1H), 3.98 – 3.88 (m, 1H), 3.77 – 3.62 (m, 3H), 3.62 – 3.50 (m, 1H), 3.45 – 3.33 (m, 1H), 2.52 – 2.26 (m, 2H), 1.35 – 1.12 (m, 4H), 1.04 (dt, *J*=30.5, 7.1, 2H). ^{13}C NMR (126 MHz, $CDCl_3$) δ 172.3, 171.8, 167.9, 167.6, 153.6, 130.9, 129.1, 128.9, 128.7, 120.4, 120.0, 119.1, 116.8, 116.4, 66.5, 64.2, 62.1, 61.9, 61.7, 61.6, 59.1, 54.3, 51.9, 51.8, 38.7, 37.3, 35.4, 32.1, 32.0, 31.6, 14.1, 13.8, 13.7. LRMS (ESI): Mass calcd for $C_{19}H_{24}O_7$ [M+H]+: 365.1; found 365.2 HRMS (ESI): Mass calcd for $C_{19}H_{24}O_7$ [M+H]+: 365.1522; found 365.1525 FTIR (neat): 3033, 2854, 2391, 1822, 1663, 1650, 1626, 1544, 1486, 1300, 1252, 1210, 1184, 1151, 1039, 1008, 935, 904, 742

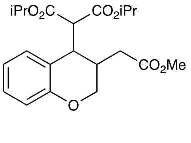

Prepared according to the general procedure in 82% yield in a 1.2:1 ratio of diastereomers

^{1}H NMR (500 MHz, Chloroform-*d*) δ = 7.15 – 6.99 (m, 2H), 6.76 (q, *J*=8.4, 7.6, 2H), 5.14 – 5.06 (m, 1H), 5.09 – 4.94 (m, 1H), 4.89 (hept, *J*=6.3, 1H), 4.78 (dq, *J*=12.5, 6.4, 1H), 4.25 – 4.09 (m, 2H), 3.79 – 3.66 (m, 1H), 3.66 (s, 1H), 3.66 (s, 2H), 3.55 – 3.45 (m, 1H), 3.40 (d, *J*=9.7, 1H), 2.48 – 2.38 (m, 1H), 2.42 – 2.17 (m, 2H), 1.28 – 1.19 (m, 5H), 1.22 – 1.10 (m, 3H), 1.08 (d, *J*=6.3, 1H), 0.98 (s, 1H), 0.99 – 0.88 (m, 2H), 0.87 (s, 1H). ^{13}C NMR (126 MHz, $CDCl_3$) δ 172.4, 171.9, 167.8, 167.4, 167.2, 153.9, 153.6, 131.1, 129.2, 128.7, 128.6, 120.4, 120.0, 119.3, 116.8, 116.4, 69.7, 69.6, 69.5, 69.4, 69.3, 66.7, 64.3, 59.4, 54.6, 51.9, 51.7, 38.4, 37.0, 35.5, 32.0, 32.0, 31.7, 21.7, 21.5, 21.5, 21.4, 21.17.

LRMS (ESI): Mass calcd for $C_{21}H_{28}O_7$ [M+H]+: 393.2; found 393.2 HRMS (ESI): Mass calcd for $C_{21}H_{28}O_7$ [M+H]+: 393.1835; found 393.1837 FTIR (neat): 3072, 2984, 2652, 1843, 1671, 1653, 1601, 1501, 1474, 1283, 1270, 1199, 1180, 1161, 1049, 1007, 971, 884, 740

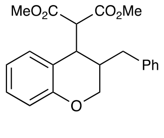

Prepared according to the general procedure in 85% yield in a 1.1:1 ratio of diastereomers

^{1}H NMR (500 MHz, Chloroform-*d*) δ = 7.32 – 7.19 (m, 5H), 7.15 (ddd, *J*=8.5, 7.3, 1.7, 1H), 7.06 (dd, *J*=7.7, 1.6, 1H), 6.96 – 6.86 (m, 2H), 4.60 (d, *J*=10.3, 1H), 4.21 (d, *J*=9.2, 1H), 4.09 (dd, *J*=11.8, 1.5, 1H), 3.79 (dd, *J*=11.7, 2.4, 1H), 3.47 (dddd, *J*=10.5, 9.1, 2.4, 1.5, 1H), 3.40 (s, 3H), 3.22 (s, 3H). ^{13}C NMR (126 MHz, $CDCl_3$) δ 169.6, 168.7, 156.0, 137.2, 131.5, 128.4, 128.3, 127.9, 127.4, 121.2, 120.6, 118.0, 65.0, 64.3, 52.1, 51.9, 42.5, 36.1, 34.4. LRMS (ESI): Mass calcd for $C_{21}H_{22}O_5$ [M+H]+: 355.1; found 355.1 HRMS (ESI): Mass calcd for $C_{21}H_{22}O_5$ [M+H]+: 355.1311; found 355.1310 FTIR (neat): 3067, 2980, 2871, 1654, 1651, 1605, 1555, 1504, 1421, 1281, 1270, 1190, 1180, 1161, 1079, 1021, 925, 880, 742

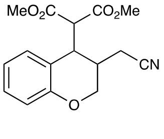

Prepared according to the general procedure in 81% yield in a 1.2:1 ratio of diastereomers

¹H NMR (500 MHz, Chloroform-*d*) δ = 7.51 – 7.34 (m, 1H), 7.31 – 7.20 (m, 1H), 7.18 – 7.02 (m, 2H), 4.53 – 4.46 (m, 1H), 4.42 (dd, *J*=12.2, 2.3, 1H), 4.00 (s, 3H), 3.97 – 3.85 (m, 2H), 3.85 (s, 3H), 3.70 (dd, *J*=9.8, 1.7, 1H), 2.66 (d, *J*=7.9, 2H), 2.61 – 2.51 (m, 1H). ¹³C NMR (126 MHz, $CDCl_3$) δ 167.9, 167.8, 153.1, 144.5, 130.5, 129.4, 121.1, 117.8, 117.6, 117.3, 115.2, 63.2, 58.4, 53.0, 52.7, 52.6, 38.1, 32.5, 29.8, 19.6, 12.8. LRMS (ESI): Mass calcd for $C_{16}H_{17}NO_5$ [M+H]+: 304.1; found 304.1 HRMS (ESI): Mass calcd for $C_{16}H_{17}NO_5$ [M+H]+: 304.1107; found 304.1105 FTIR (neat): 3037, 2910, 2591, 1791, 1694, 1653, 1623, 1585, 1365, 1283, 1227, 1197, 1176, 1162, 1063, 999, 924, 885, 773

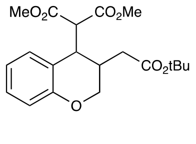

Prepared according to the general procedure in 77% yield in a 1.5:1 ratio of diastereomers
¹H NMR (500 MHz, Chloroform-*d*) δ = 7.11 (qd, *J*=7.9, 1.7, 1H), 6.99 (dd, *J*=8.0, 1.8, 1H), 6.95 – 6.86 (m, 1H), 6.81 – 6.74 (m, 2H), 4.25 – 4.12 (m, 2H), 4.07 (dd, *J*=11.1, 9.4, 1H), 3.93 – 3.87 (m, 1H), 3.77 (d, *J*=6.2, 3H), 3.64 – 3.55 (m, 1H), 3.55 (s, 2H), 3.42 (s, 1H), 2.71 – 2.61 (m, 1H), 2.39 – 2.15 (m, 2H), 2.07 (dd, *J*=15.9, 9.2, 1H), 1.43 (d, *J*=3.6, 9H). ¹³C NMR (126 MHz, $CDCl_3$) δ 171.1, 170.5, 169.0, 168.6, 168.3, 168.0, 153.8, 153.6, 130.6, 129.0, 128.9, 128.8, 121.4, 120.4, 120.0, 119.1, 116.9, 116.5, 81.2, 80.9, 66.4, 64.4, 58.8, 54.0, 53.0, 52.8, 52.5, 52.5, 38.9, 37.5, 36.8, 33.7, 32.3, 31.6, 28.1, 28.1. LRMS (ESI): Mass calcd for $C_{20}H_{26}O_7$ [M+H]+: 379.1; found 379.2,HRMS (ESI): Mass calcd for $C_{20}H_{26}O_7$ [M+H]+: 379.1679; found 379.1681 FTIR (neat): 3057, 2972, 2548, 1877, 1676, 1643, 1616, 1541, 1374, 1297, 1252, 1211, 1177, 1126, 1068, 1009, 955, 867, 782

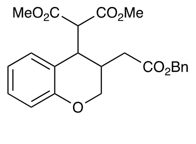

Prepared according to the general procedure in 88% yield in a 1.2:1 ratio of diastereomers
¹H NMR (500 MHz, Chloroform-*d*) δ = 7.39 – 7.25 (m, 5H), 7.11 (qd, *J*=7.1, 6.2, 1.6, 1H), 6.98 (dd, *J*=8.2, 1.7, 1H), 6.78 (dt, *J*=8.1, 5.6, 2H), 5.16 – 5.05 (m, 2H), 4.26 – 4.15 (m, 2H), 4.09 (dd, *J*=11.2, 8.9, 1H), 3.93 (dd, *J*=8.2, 4.1, 1H), 3.75 (s, 2H), 3.71 (s, 1H), 3.66 – 3.55 (m, 1H), 3.56 (s, 2H), 3.43 (d, *J*=11.2, 2H), 2.71 (tq, *J*=8.9, 4.2, 1H), 2.49 (dd, *J*=18.0, 10.1, 1H), 2.44 – 2.31 (m, 2H). ¹³C NMR (126 MHz, $CDCl_3$) δ 171.7, 171.1, 168.2, 168.0, 153.5, 135.7, 130.6, 129.0, 128.9, 128.8, 128.6, 128.5, 128.4, 128.3, 128.2, 120.5, 120.1, 118.9, 117.0, 116.5, 66.8, 66.5, 64.2, 58.8, 54.1, 53.0, 52.8, 52.6, 52.5, 38.9, 37.5, 35.6, 32.3, 32.1, 31.5. LRMS (ESI): Mass calcd for $C_{23}H_{24}O_7$ [M+H]+: 413.2; found 413.2 HRMS (ESI): Mass calcd for $C_{23}H_{24}O_7$ [M+H]+: 413.1522; found 413.1525 FTIR (neat): 3004, 2936, 2750, 1987, 1683, 1629, 1627, 1524, 1394, 1279, 1254, 1194, 1186, 1153, 1110, 998, 945, 849, 698

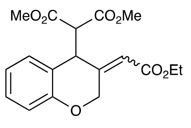

Prepared according to the general procedure in 81% yield in a 1:1 ratio of *Z*/*E* isomers
^{1}H NMR (500 MHz, Chloroform-*d*) δ = 7.30 (dd, *J*=7.7, 1.7, 1H), 7.28 (s, 2H), 7.21 – 7.10 (m, 1H), 6.99 – 6.82 (m, 1H), 5.89 (p, *J*=0.9, 1H), 5.62 (d, *J*=8.4, 1H), 5.01 (dd, *J*=14.3, 1.9, 1H), 4.53 (dt, *J*=14.4, 1.2, 1H), 4.26 – 4.12 (m, 2H), 3.86 (d, *J*=8.5, 1H), 3.71 (s, 2H), 3.68 (s, 1H), 3.59 (s, 2H), 1.55 (s, 1H), 1.31 (t, *J*=7.1, 3H), 1.26 (t, *J*=9.8, 1H). ^{13}C NMR (126 MHz, $CDCl_3$) δ 167.8, 167.6, 165.1, 154.3, 151.2, 130.1, 129.1, 122.6, 121.7, 117.5, 117.0, 69.3, 60.5, 57.2, 52.7, 52.5, 52.4, 37.8, 28.6, 14.2. LRMS (ESI): Mass calcd for $C_{18}H_{20}O_7$ [M+H]+: 349.1; found 349.1 HRMS (ESI): Mass calcd for $C_{18}H_{20}O_7$ [M+H]+: 349.1209; found 349.1210 FTIR (neat): 3095, 2750, 2389, 1840, 1691, 1658, 1619, 1525, 1471, 1276, 1264, 1204, 1163, 1129, 1099, 1019, 946, 862, 727

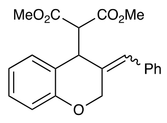

Prepared according to the general procedure in 86% yield in a 1:1 ratio of *Z*/*E* isomers
^{1}H NMR (500 MHz, Chloroform-*d*) δ = 7.45 – 7.16 (m, 5H), 7.13 (s, 1H), 7.19 – 7.03 (m, 1H), 7.03 (d, *J*=8.2, 1H), 7.00 – 6.77 (m, 2H), 5.12 – 4.94 (m, 1H), 4.93 (s, 1H), 4.59 (dd, *J*=12.9, 1.5, 1H), 4.25 (d, *J*=10.8, 1H), 3.97 – 3.85 (m, 1H), 3.80 – 3.71 (m, 1H), 3.68 (s, 1H), 3.65 (s, 2H), 3.61 (d, *J*=13.1, 1H), 3.51 (s, 1H), 3.24 (d, *J*=7.7, 1H). ^{13}C NMR (126 MHz, $CDCl_3$) δ 169.6, 167.5, 155.8, 154.1, 135.5, 133.2, 132.1, 132.0, 131.8, 131.1, 129.5, 129.3, 129.0, 128.7, 128.7, 128.6, 128.5, 128.4, 128.3, 128.2, 127.6, 127.5, 126.6, 126.5, 122.3, 121.3, 121.2, 120.6, 117.2, 117.1, 112.5, 112.0, 87.2, 84.0, 70.4, 65.2, 57.5, 57.4, 56.7, 52.7, 52.5, 52.4, 51.4, 45.6, 37.9, 30.3. LRMS (ESI): Mass calcd for $C_{21}H_{20}O_5$ [M+H]+: 353.1; found 353.1 HRMS (ESI): Mass calcd for $C_{21}H_{20}O_5$ [M+H]+: 353.1311; found 353.1312 FTIR (neat): 3080, 2843, 2561, 1773, 1672, 1655, 1618, 1552, 1486, 1295, 1268, 1215, 1187, 1134, 1062, 1017, 987, 855, 766

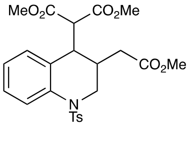

Prepared according to the general procedure in 85% yield in a 1.3:1 ratio of diastereomers
^{1}H NMR (500 MHz, Chloroform-*d*) δ = 7.84 (ddd, *J*=13.3, 8.4, 1.1, 1H), 7.71 – 7.59 (m, 2H), 7.37 – 7.19 (m, 3H), 7.07 (dd, *J*=7.7, 1.8, 1H), 7.06 – 6.92 (m, 1H), 3.89 – 3.79 (m, 1H), 3.77 (d, *J*=2.6, 4H),

3.77 – 3.67 (m, 1H), 3.69 (s, 1H), 3.67 (s, 2H), 3.64 – 3.43 (m, 1H), 3.44 (s, 2H), 3.35 (s, 1H), 2.78 (d, *J*=11.0, 1H), 2.62 (dp, *J*=10.0, 3.8, 3.2, 1H), 2.42 (d, *J*=2.1, 3H), 2.18 (d, *J*=7.1, 1H). ^{13}C NMR (126 MHz, $CDCl_3$) δ 171.8, 167.7, 167.6, 144.1, 136.2, 135.5, 130.7, 129.8, 129.8, 128.5, 128.2, 128.1, 127.2, 127.1, 126.4, 124.1, 122.9, 122.3, 56.2, 53.0, 52.6, 52.4, 52.2, 52.0, 51.8, 48.0, 47.0, 41.8, 37.4, 34.5, 34.2, 32.8, 21.6. LRMS (ESI): Mass calcd for $C_{24}H_{27}NO_8S$ [M+H]+: 490.2; found 490.2 HRMS (ESI): Mass calcd for $C_{24}H_{27}NO_8S$ [M+H]+: 490.1457; found 490.1455 FTIR (neat): 3029, 2819, 2633, 2317, 1706, 1632, 1604, 1546, 1331, 1304, 1247, 1192, 1168, 1141, 1072, 1008, 957, 886, 713

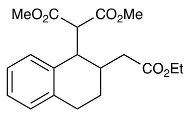

Prepared according to the general procedure in 82% yield in a 1.2:1 ratio of diastereomers
^{1}H NMR (500 MHz, Chloroform-*d*) δ = 7.31 – 7.19 (m, 4H), 7.12 (dt, *J*=15.7, 6.8, 1H), 4.34 – 4.19 (m, 2H), 3.80 (s, 6H), 3.85 – 3.72 (m, 1H), 3.35 (d, *J*=7.7, 2H), 2.95 – 2.85 (m, 2H), 2.60 (dtd, *J*=8.1, 6.7, 1.6, 2H), 1.43 – 1.31 (m, 3H). ^{13}C NMR (126 MHz, $CDCl_3$) δ 169.2, 166.5, 147.8, 139.0, 135.4, 129.9, 129.6, 129.5, 129.4, 129.3, 127.2, 126.6, 122.0, 60.4, 60.3, 52.7, 52.6, 52.6, 38.1, 35.7, 33.4, 31.3, 30.9, 30.7, 29.7, 28.5, 14.3. LRMS (ESI): Mass calcd for $C_{19}H_{24}O_6$ [M+H]+: 349.1; found 349.1 HRMS (ESI): Mass calcd for $C_{19}H_{24}O_6$ [M+H]+: 349.1573; found 349.1573 FTIR (neat): 3090, 2759, 2533, 2099, 1660, 1630, 1623, 1531, 1475, 1302, 1259, 1209, 1178, 1133, 1075, 1000, 968, 904, 804

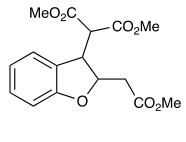

Prepared according to the general procedure in 72% yield in a 1.1:1 ratio of diastereomers
^{1}H NMR (500 MHz, Chloroform-*d*) δ = 7.24 – 7.00 (m, 2H), 6.86 – 6.76 (m, 2H), 5.07 – 5.00 (m, 1H), 3.86 – 3.79 (m, 1H), 3.82 – 3.72 (m, 1H), 3.75 – 3.64 (m, 8H), 3.67 – 3.56 (m, 1H), 2.85 – 2.67 (m, 2H) ^{13}C NMR (126 MHz, $CDCl_3$) δ 170.7, 168.1, 168.0, 158.8, 129.6, 125.4, 125.1, 120.8, 110.4, 82.4, 56.0, 52.9, 52.7, 51.9, 46.4, 40.3. LRMS (ESI): Mass calcd for $C_{16}H_{18}O_7$ [M+H]+: 323.1; found 323.1 HRMS (ESI): Mass calcd for $C_{16}H_{18}O_7$ [M+H]+: 323.1053; found 323.1051 FTIR (neat): 3016, 2953, 2588, 1766, 1667, 1633, 1628, 1580, 1446, 1306, 1250, 1212, 1178, 1138, 1107, 991, 990, 906, 798

Procedure for Krapcho Decarboxylation and Dieckmann Condensation

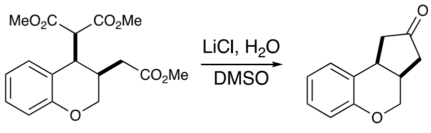

To a 0.5-2 mL Biotage microwave vial outfitted with a magnetic stir bar was added 2a (1 equiv) and LiCl (4.1 equiv). Water (3 equiv) was then added, followed by DMSO (0.5 M). The vial was then sealed and heated in an oil bath at 140 ˚C for 18 hours. Upon observation of complete conversion, the vial was removed from oil bath and allowed to cool to room temperature. The solution was diluted with water (15 mL) and extracted with EtOAc (3 x 10 mL). The organic extracts were then pooled and washed with water (4 x 15 mL) and saturated brine solution (4 x 15 mL). The organic extracts were then concentrated under reduced pressure onto silica gel and loaded onto a column of silica gel. 3a was then isolated via flash chromatography (5-40% ethyl acetate/hexanes) as a thick, clear oil (85%).

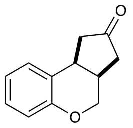

^{1}H NMR (500 MHz, Chloroform-*d*) δ = 7.17 – 7.07 (m, 2H), 7.00 – 6.89 (m, 1H), 6.86 (d, *J*=8.2, 1H), 4.21 – 4.11 (m, 1H), 3.95 (dd, *J*=11.2, 7.5, 1H), 3.61 (dt, *J*=8.7, 6.7, 1H), 2.86 – 2.75 (m, 2H), 2.56 – 2.42 (m, 2H), 2.25 (ddd, *J*=19.1, 6.4, 1.5, 1H). ^{13}C NMR (126 MHz, $CDCl_3$) δ 217.2, 153.9, 130.0, 127.9, 121.5, 117.3, 65.6, 45.8, 39.5, 34.7, 34.6, 33.9, 22.7. LRMS (ESI): Mass calcd for $C_{12}H_{21}O_2$ [M+H]+: 189.1; found 189.1 HRMS (ESI): Mass calcd for $C_{12}H_{21}O_2$ [M+H]+: 189.0837; found 189.0835 FTIR (neat): 3095, 1720, 1472, 1325, 1241, 1170, 1111, 1032, 968, 904, 804

Stern-Volmer Fluorescence Quenching Experiments

Stern-Volmer fluorescence quenching experiments were run with freshly prepared solutions of 3DPAFIPN in acetonitrile at room temperature under an inert atmosphere. The solutions were irradiated at 425 nm and fluorescence was measured at 523 nm. Control experiments show that, at the concentrations employed in these studies, arylidene malonate didn't measurably quench the excited state of 3DPAFIPN. The data summarized in the tables is the fluorescence intensity measured three times for each sample. The data shown in the graphs is the average of three experiments.

Quencher	Substrate Conc (M)	PC Conc (mM)	I0	I	I0/I
AM (no $Sc(OTf)_3$)	0.0125	0.01	386.2906667	371.2617	1.040480883
AM (no $Sc(OTf)_3$)	0.025	0.01	386.2906667	382.7520	1.009245325
AM (no $Sc(OTf)_3$)	0.0375	0.01	386.2906667	374.7523	1.030789223
AM (no $Sc(OTf)_3$)	0.05	0.01	386.2906667	383.7777	1.006548062
AM (no $Sc(OTf)_3$)	0.075	0.01	386.2906667	391.8463	0.985821823
AM (no $Sc(OTf)_3$)	0.1	0.01	386.2906667	388.6503	0.993928561
AM (with $Sc(OTf)_3$)	0.0125	0.01	386.2906667	292.9457	1.318642706
AM (with $Sc(OTf)_3$)	0.025	0.01	386.2906667	261.5277	1.47705469
AM (with $Sc(OTf)_3$)	0.0375	0.01	386.2906667	246.2607	1.568625115
AM (with $Sc(OTf)_3$)	0.05	0.01	386.2906667	210.7503	1.832930276
AM (with $Sc(OTf)_3$)	0.075	0.01	386.2906667	195.9150	1.971725833
AM (with $Sc(OTf)_3$)	0.1	0.01	386.2906667	140.3707	2.751932977
HEH	0.0125	0.01	386.2906667	360.4603	1.071659295
HEH	0.025	0.01	386.2906667	345.3357	1.118594759
HEH	0.0375	0.01	386.2906667	324.0363	1.19212146
HEH	0.05	0.01	386.2906667	285.1877	1.354513928
HEH	0.075	0.01	386.2906667	254.0113	1.520761541
HEH	0.1	0.01	386.2906667	238.4723	1.619855273

Procedure for Determination of Quantum Yield

The photon flux of the fluorimeter was determined using a ferrioxolate Hatchard – Parker actinometer as described by Yoon et al.[3] Based on the average of three experiments, the photon flux at 420 nm (10 nm slit width) was determined to be 5.27712E-09 einsteins s^{-1}. UV/Vis absorbance spectra of DPAIPN in MeCN (0.1 M) indicated that essentially all light was absorbed at 420 nm (f = 0.99148). A screw-top quartz cuvette with Teflon septa was charged with **1a** (0.2 mmol, 1 equiv), DPAIPN (1 mol %), scandium triflate (10 mol %), HEH (1.5 equiv), and a small Teflon coated magnetic stirbar in a glovebox. The cuvette was sealed and removed from glovebox. The cuvette was then capped with a PTFE stopper, and 2 mL sparged MeCN added. The solution was stirred until homogenous. The sample was placed in the fluorimeter and irradiated (λ = 420 nm, slit width= 10.0 nm) for 5400 s (3 hours). 1 H NMR based on a trimethoxybenzene standard determined the yield of product formed was 30%. The average quantum yield of the two experiments was determined to be 1.0.

Procedure for Light/Dark Experiment

To verify the necessity of light to maintain the conversion of **1a** to **2a**, a “light/dark” experiment was performed. A J-Young NMR tube was charged with **1a** (0.2 mmol, 1 equiv), DPAIPN (1 mol %), scandium triflate (10 mol %), and HEH (1.5 equiv) in a glovebox. Upon removal from the glovebox, the reaction mixture was irradiated with 456 nm Kessil blue LEDs for periods of 1 hour, followed by 2 hours of no irradiation (3 cycles of 3 hours, 9 hours total). Notably, the reaction progressed steadily during periods of irradiation, while no conversion was observed during periods without irradiation. This is indicative that propagation is likely not a operational mechanistic process over the course of the reaction.

Procedure for UV-Vis Experiments

A 1 dram vial equipped with a rubber septum and a stir bar was charged with **1a** (0.1 mmol, 1 equiv), scandium triflate (0 or 100 mol %), and tertiary amine (0 or 150 mol %) in a glovebox. Upon removal from the glovebox, the reaction mixture was stirred for 2 hours, followed by measurement of the UV-Vis spectra using a Thermo Fisher Nanodrop One Spectrophotometer.

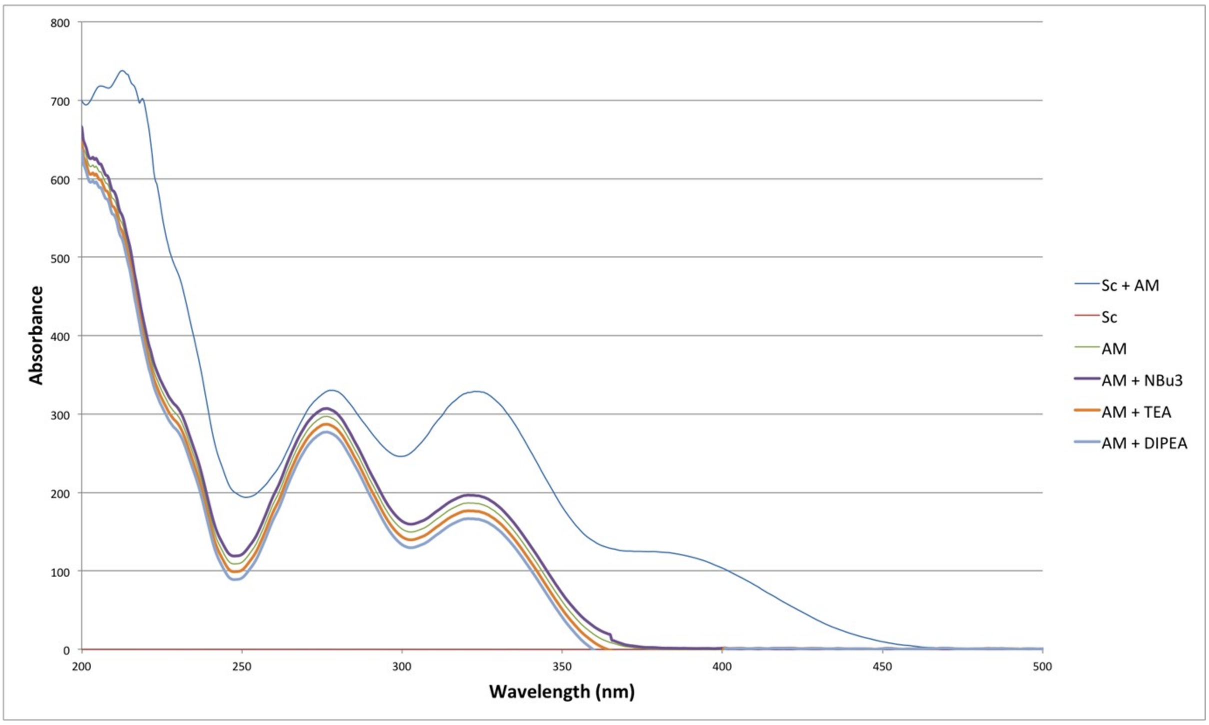

ORTEPS of Crystallographic Structure

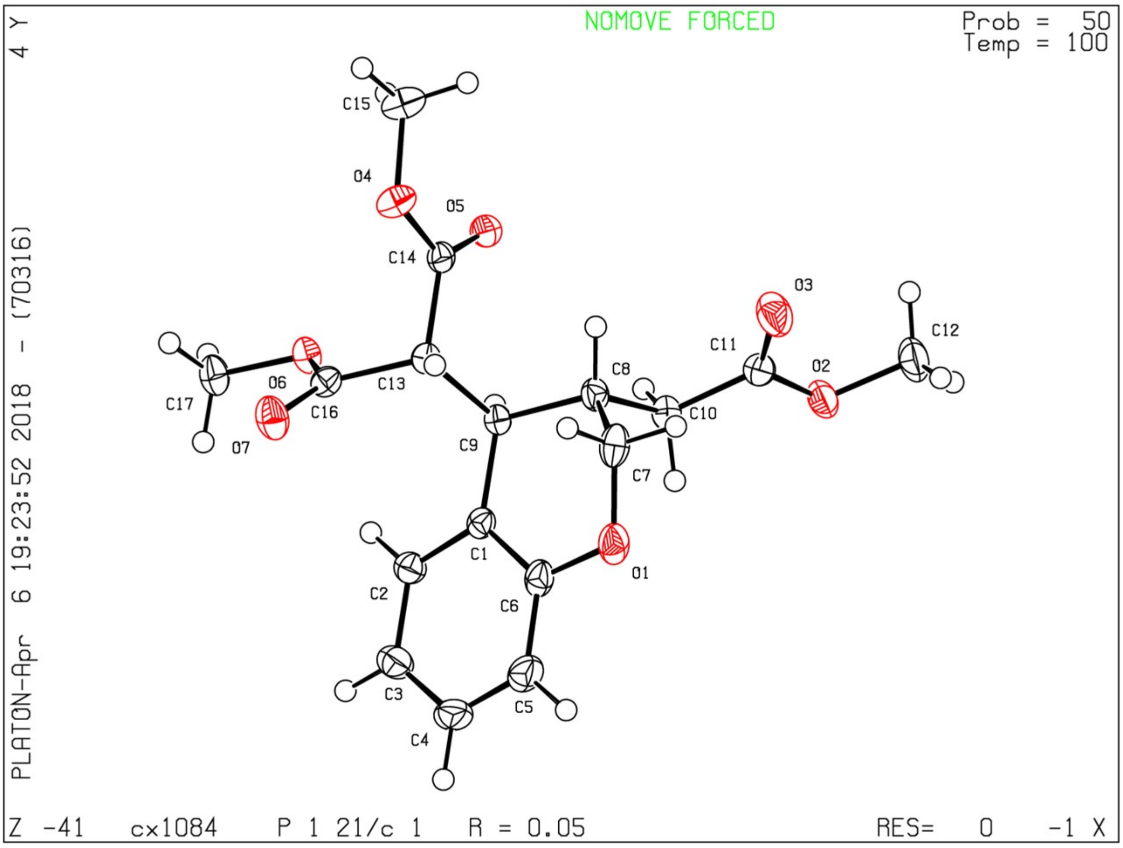

A single crystal of trans-2a was grown by evaporative diffusion in dichloromethane with hexanes as the anti-solvent at room temperature. This crystal structure was deposited in the Cambridge Crystallographic Data Centre and assigned as CCDC 1835356. Further information can be found in the CIF file.

NMR Spectra for Cyclization Starting Materials

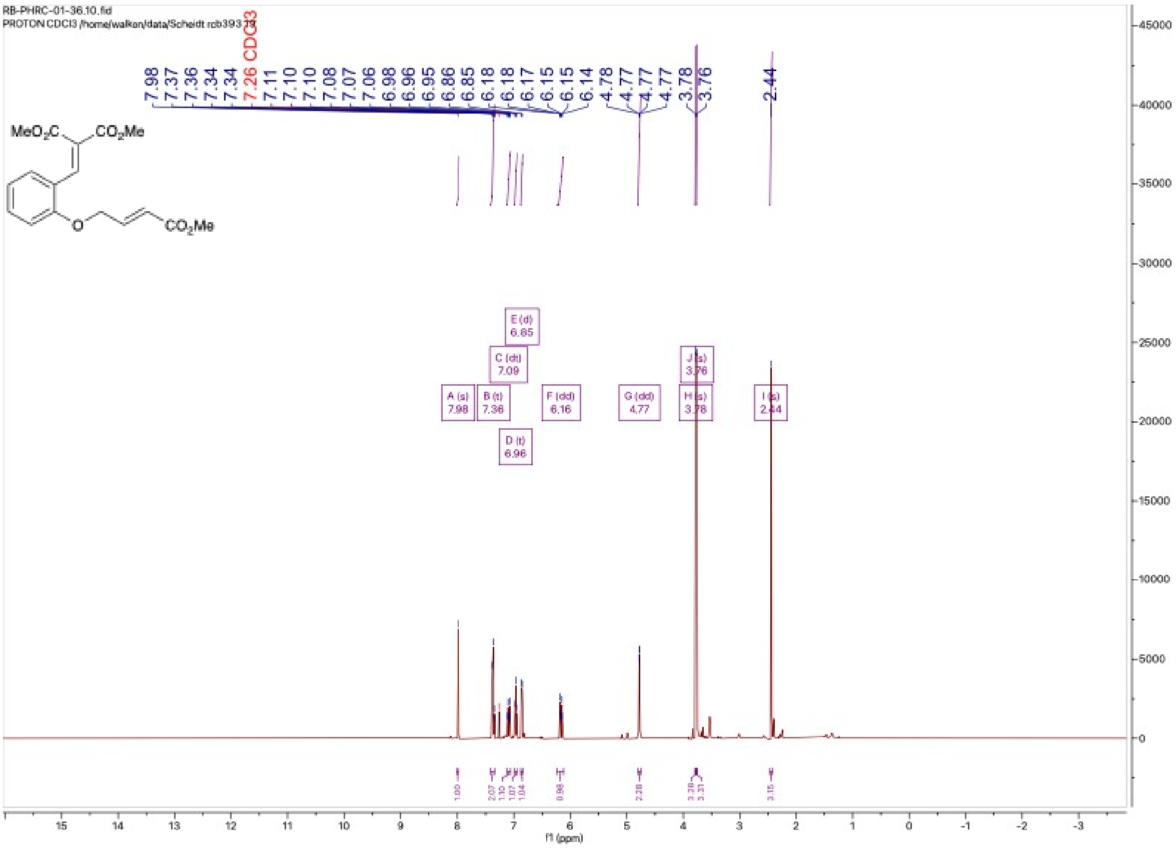

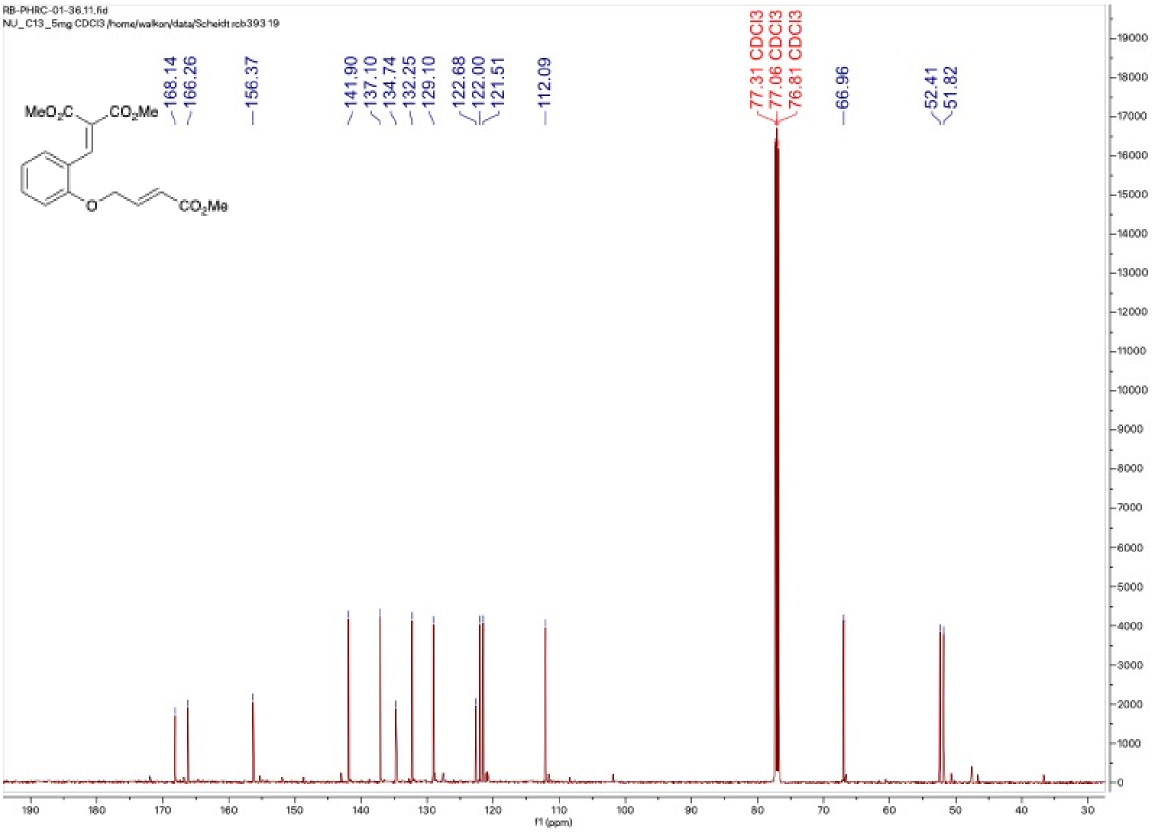

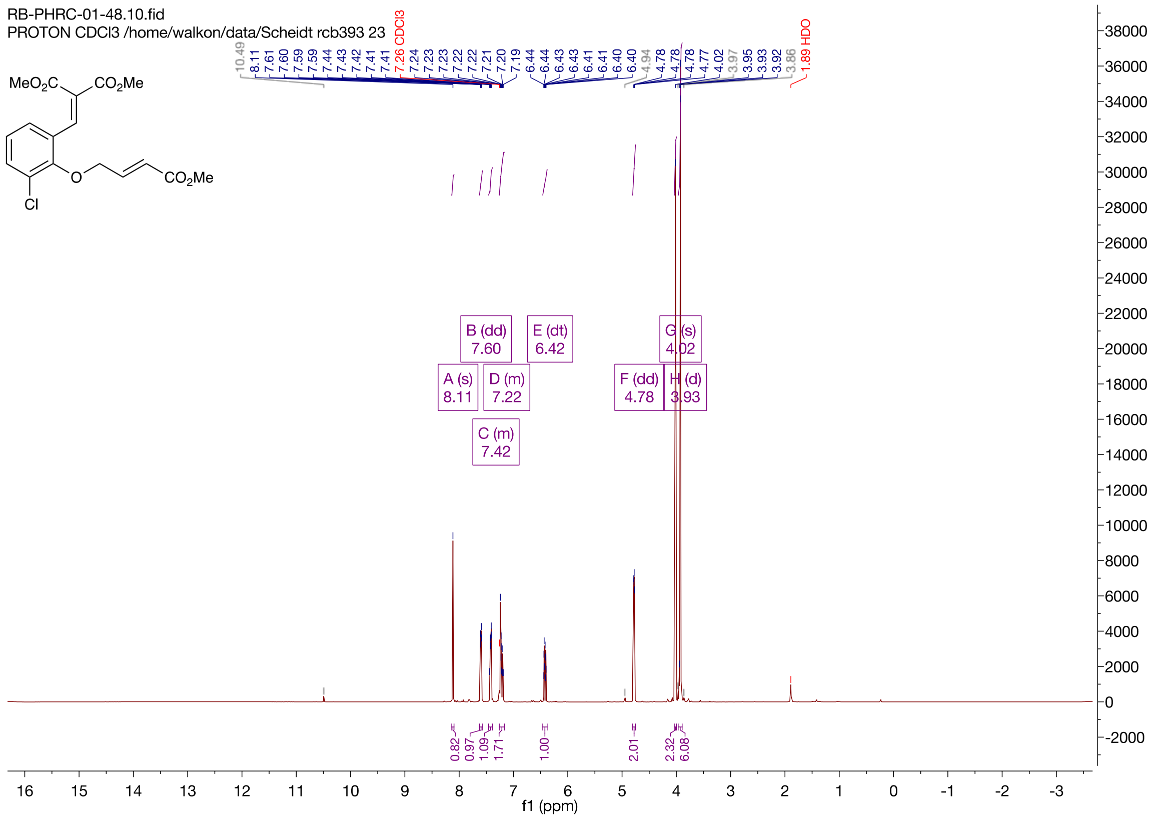
RB-PHRC-01-48.10.fid
PROTON CDCl3 /home/walkon/data/Scheidt rcb393 23
MeO2C
CO2Me
O
CO2Me
Cl
A (s) 8.11
B (dd) 7.60
C (m) 7.42
D (m) 7.22
E (dt) 6.42
F (dd) 4.78
G (s) 4.02
H (d) 3.93
1.89 HDO
7.26 CDCl3
f1 (ppm)

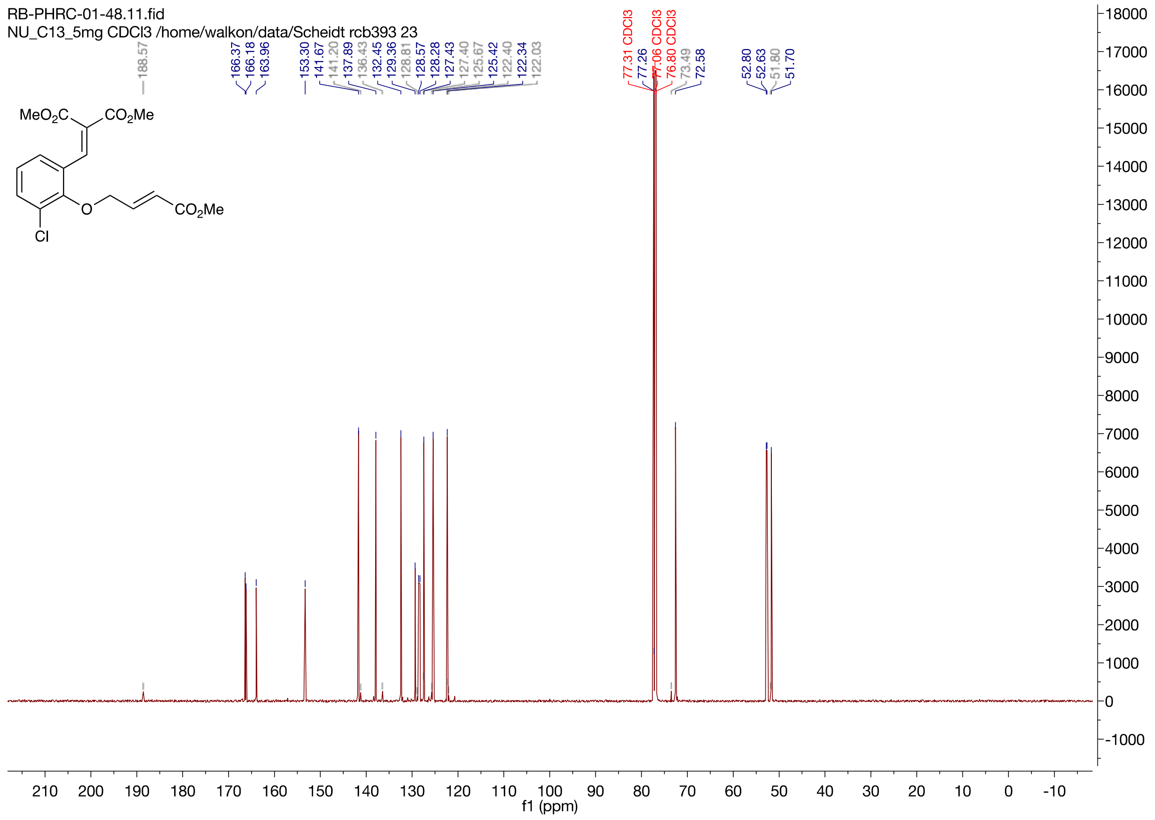
RB-PHRC-01-48.11.fid
NU_C13_5mg CDCl3 /home/walkon/data/Scheidt rcb393 23
MeO2C
CO2Me
O
CO2Me
Cl
188.57
166.37
166.18
163.96
153.30
141.67
141.20
137.89
136.43
132.45
129.36
128.81
128.57
128.28
127.43
127.40
125.67
125.42
122.40
122.34
122.03
77.31 CDCl3
77.26
77.06 CDCl3
76.80 CDCl3
73.49
72.58
52.80
52.63
51.80
51.70
f1 (ppm)

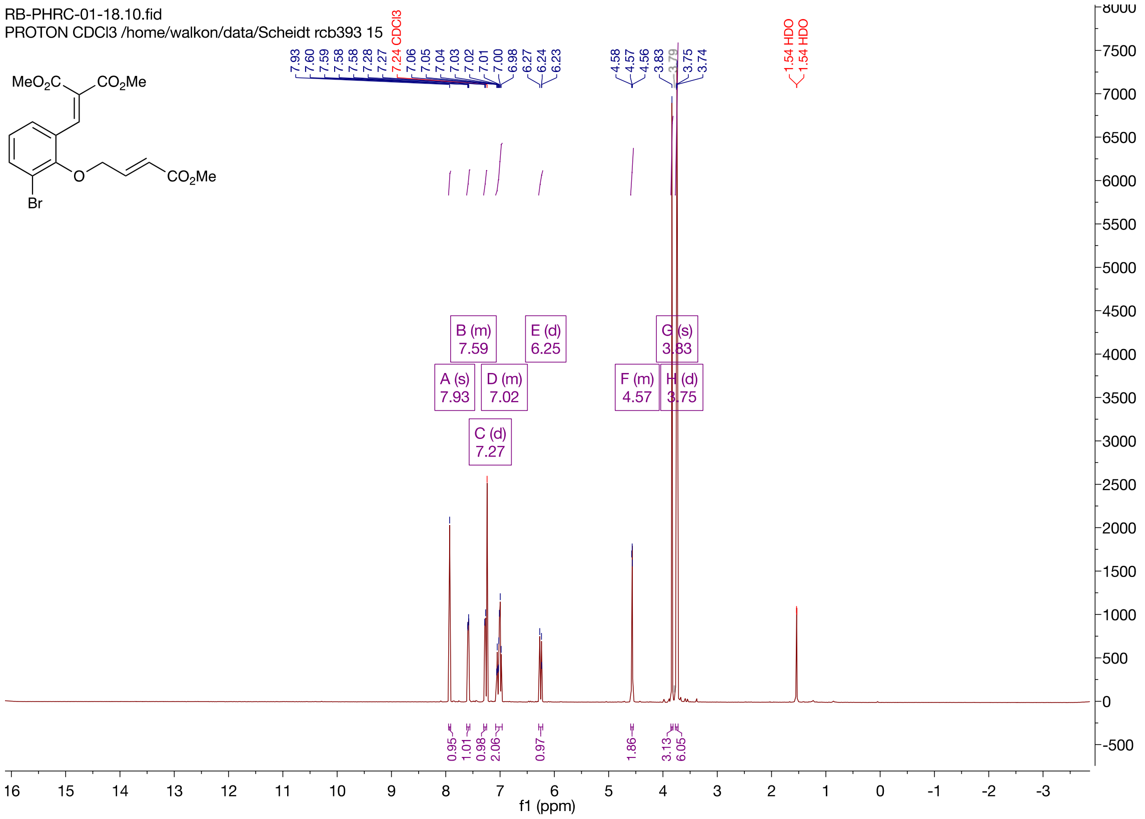
RB-PHRC-01-18.10.fid
PROTON CDCl3 /home/walkon/data/Scheidt rcb393 15
f1 (ppm)

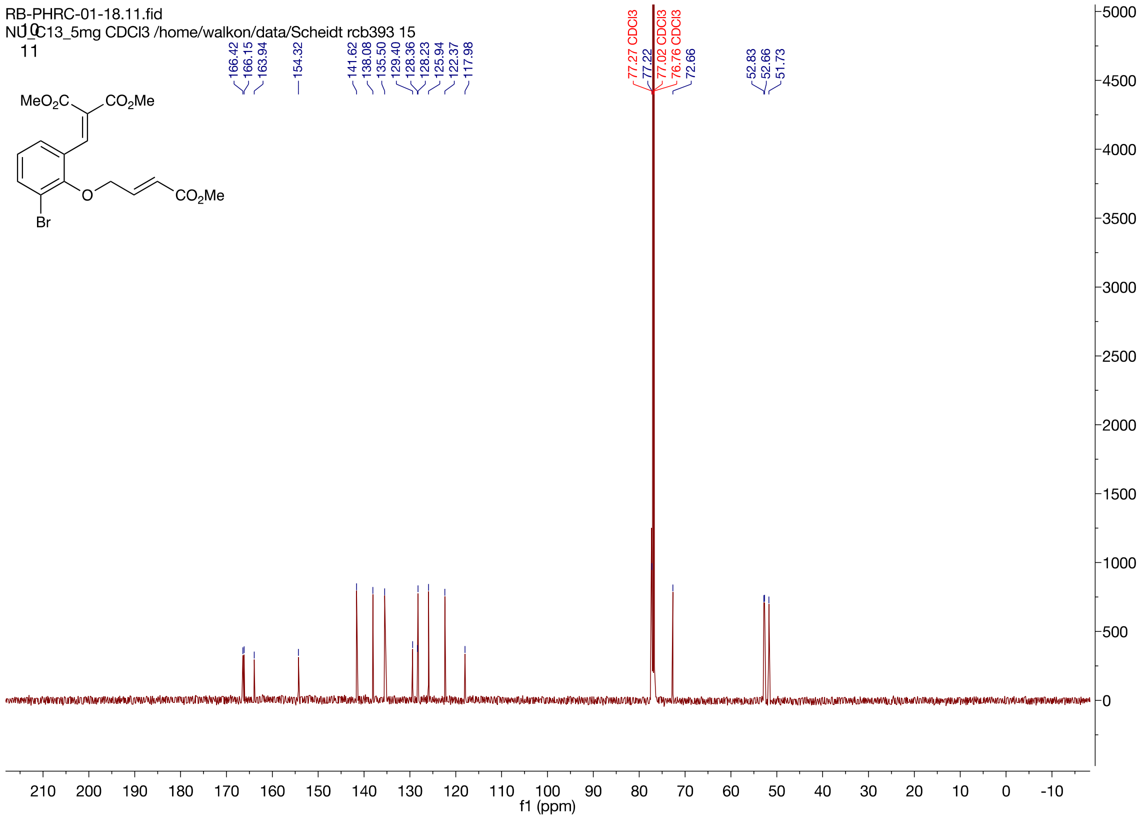
RB-PHRC-01-18.11.fid
NU_C13_5mg CDCl3 /home/walkon/data/Scheidt rcb393 15
f1 (ppm)

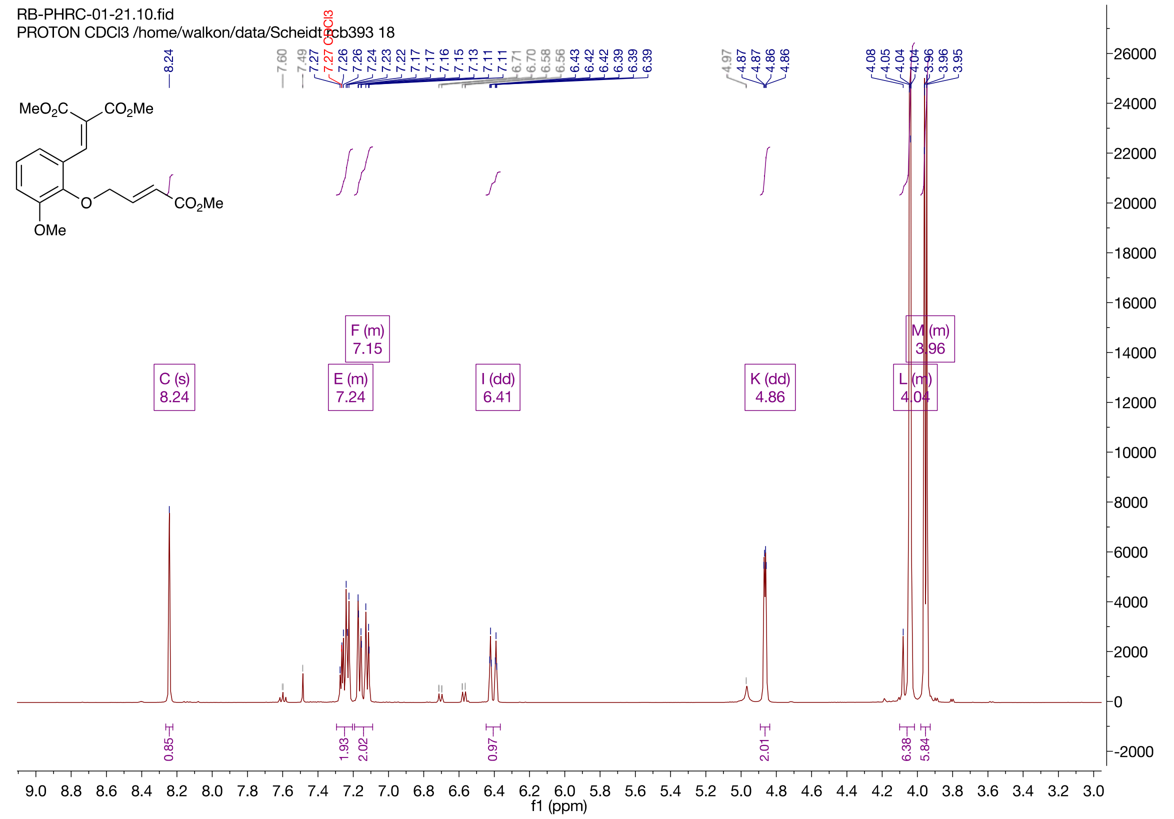
RB-PHRC-01-21.10.fid
PROTON CDCl3 /home/walkon/data/Scheidt rcb393 18
MeO2C
CO2Me
O
CO2Me
OMe
f1 (ppm)

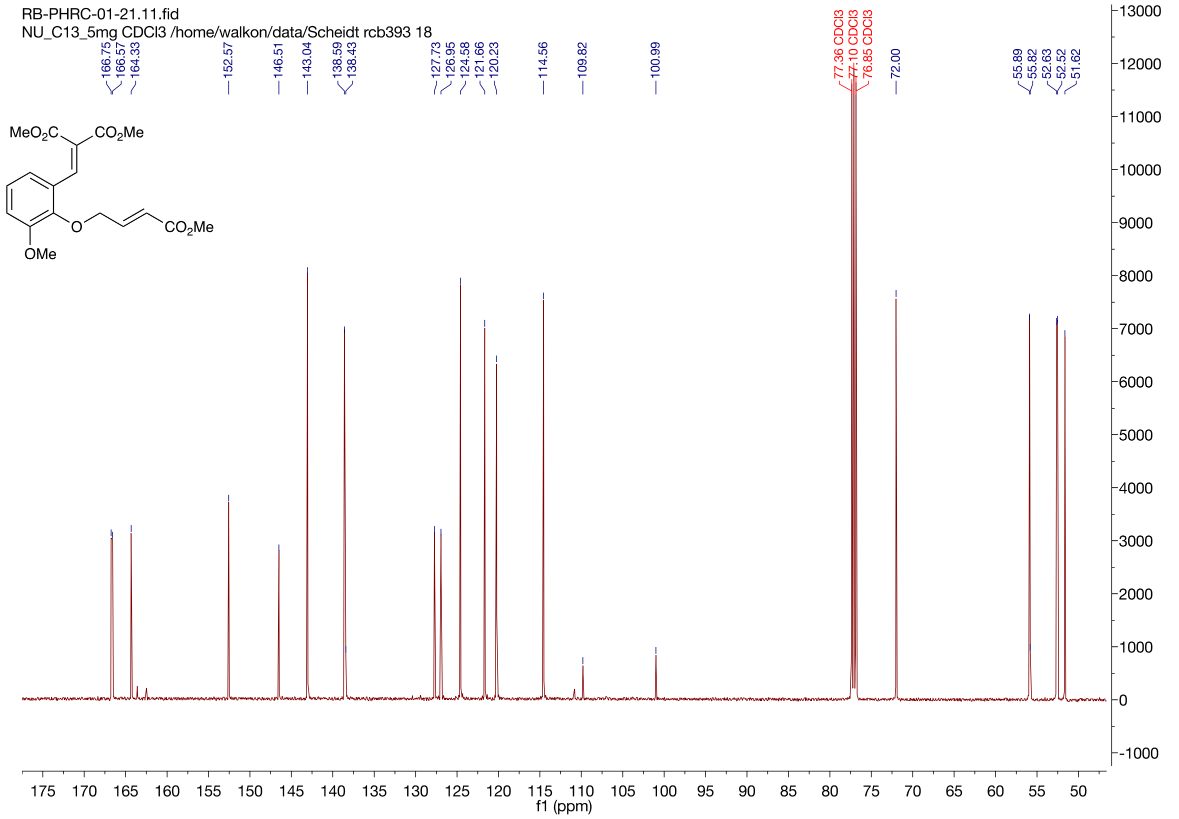
RB-PHRC-01-21.11.fid
NU_C13_5mg CDCl3 /home/walkon/data/Scheidt rcb393 18
MeO2C
CO2Me
O
CO2Me
OMe
f1 (ppm)

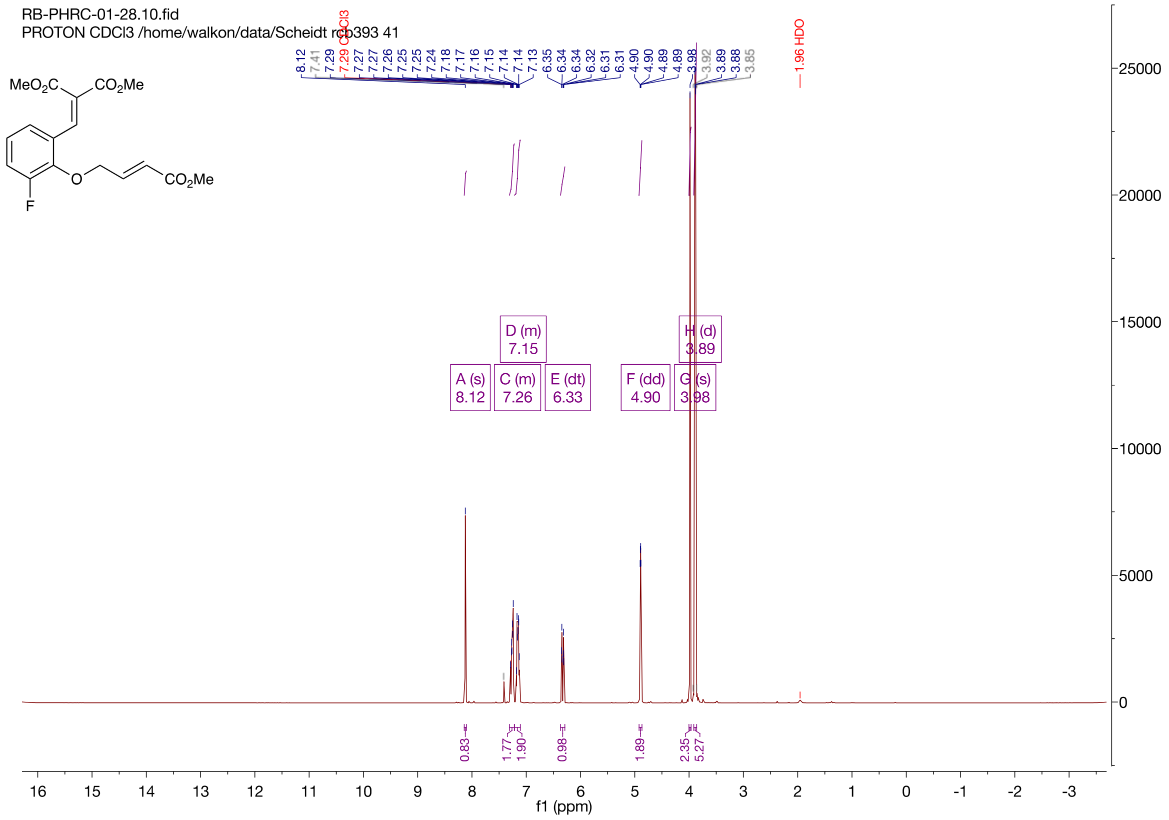
RB-PHRC-01-28.10.fid
PROTON CDCl3 /home/walkon/data/Scheidt rcb393 41
8.12 7.41 7.29 7.29 CDCl3 7.27 7.27 7.26 7.25 7.25 7.24 7.18 7.17 7.16 7.15 7.14 7.14 7.13 6.35 6.34 6.34 6.32 6.32 6.31 6.31 4.90 4.90 4.89 4.89 3.98 3.92 3.89 3.88 3.85
1.96 HDO
MeO2C CO2Me
O CO2Me
F
A (s) 8.12
C (m) 7.26
D (m) 7.15
E (dt) 6.33
F (dd) 4.90
G (s) 3.98
H (d) 3.89
0.83 1.77 1.90 0.98 1.89 2.35 5.27
f1 (ppm)

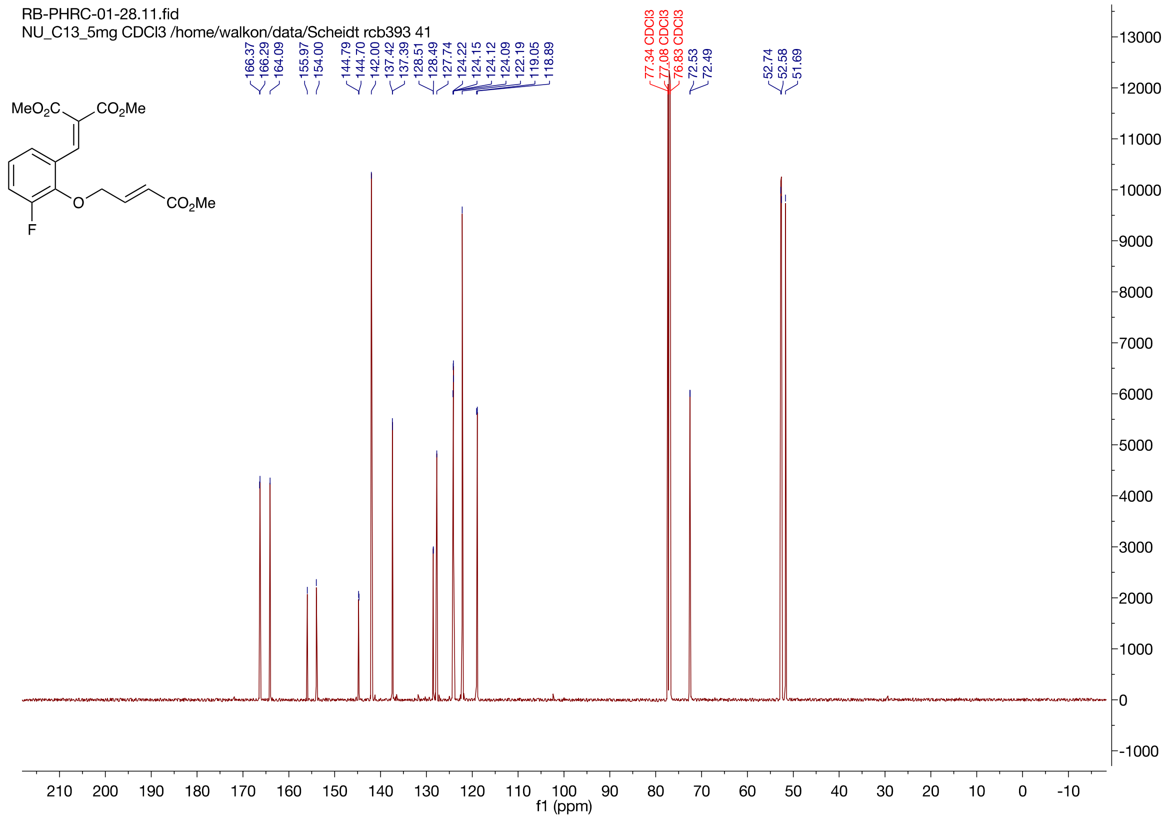
RB-PHRC-01-28.11.fid
NU_C13_5mg CDCl3 /home/walkon/data/Scheidt rcb393 41
166.37 166.29 164.09 155.97 154.00 144.79 144.70 142.00 137.42 137.39 128.51 128.49 127.74 124.22 124.15 124.12 124.09 122.19 119.05 118.89
77.34 CDCl3 77.08 CDCl3 76.83 CDCl3 72.53 72.49
52.74 52.58 51.69
MeO2C CO2Me
O CO2Me
F
f1 (ppm)

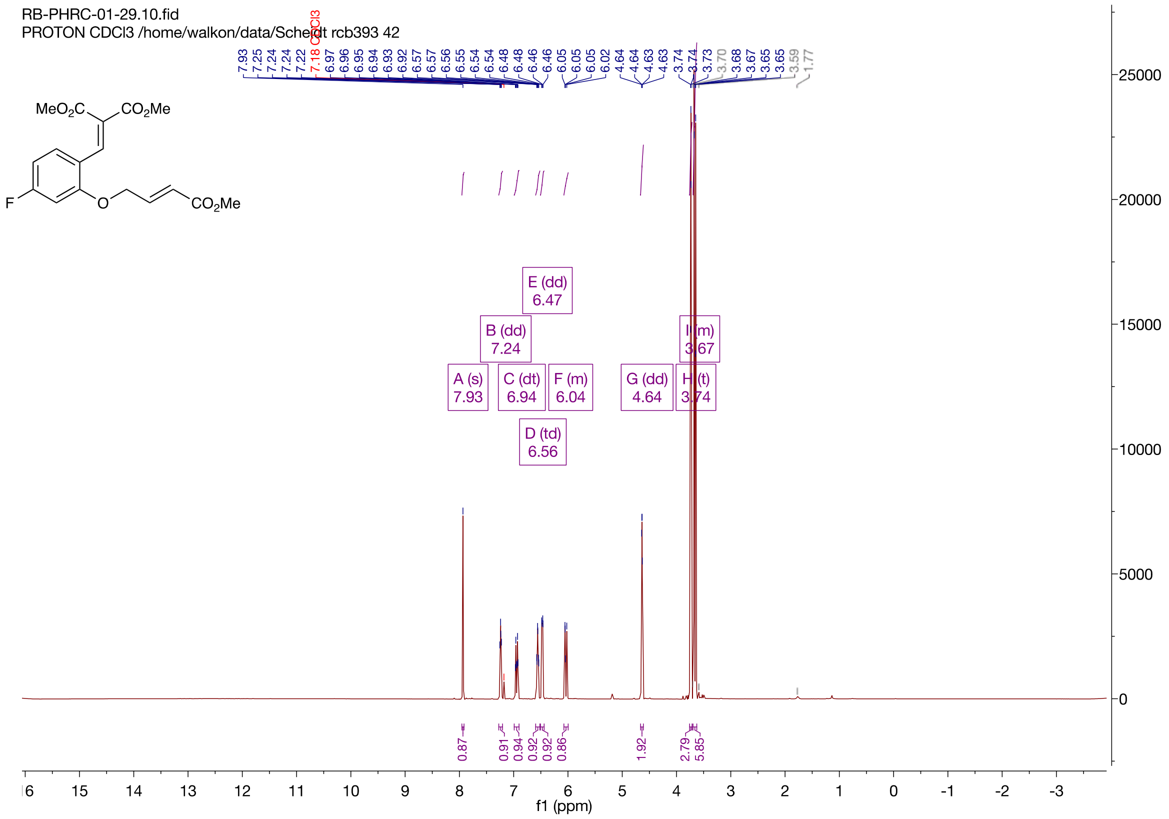

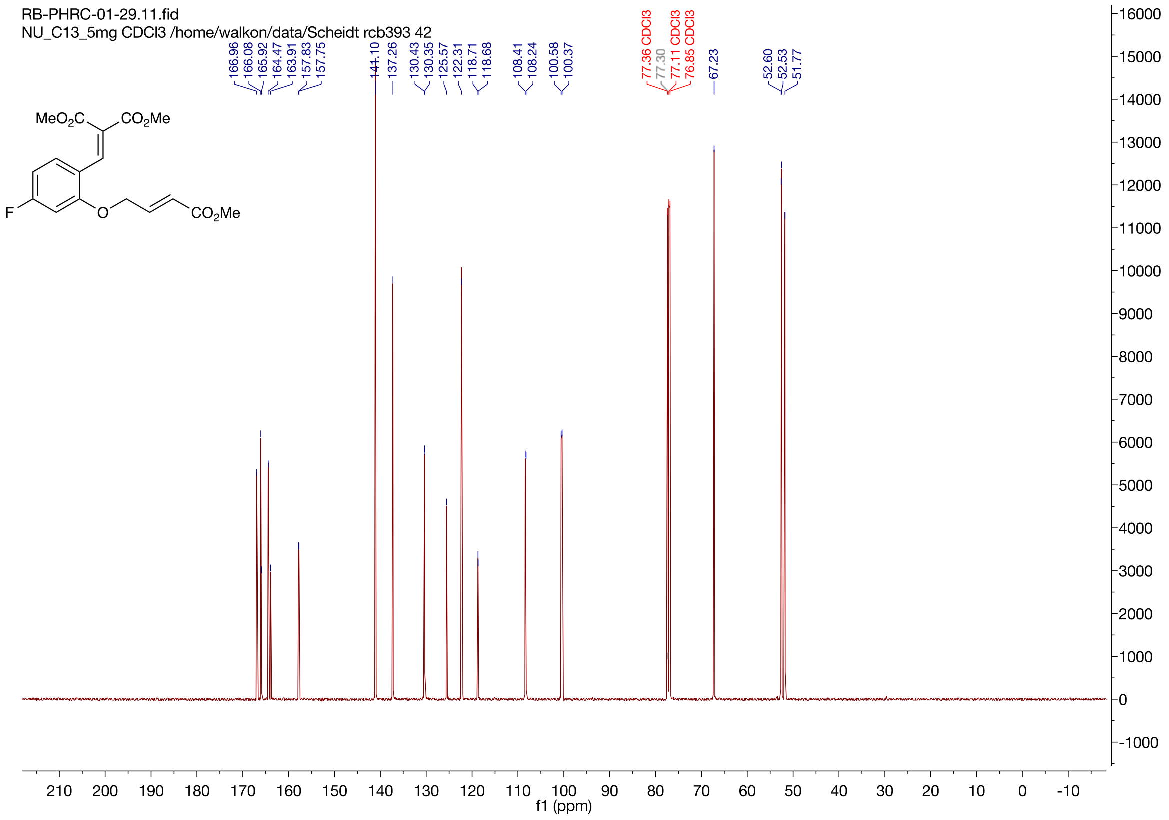

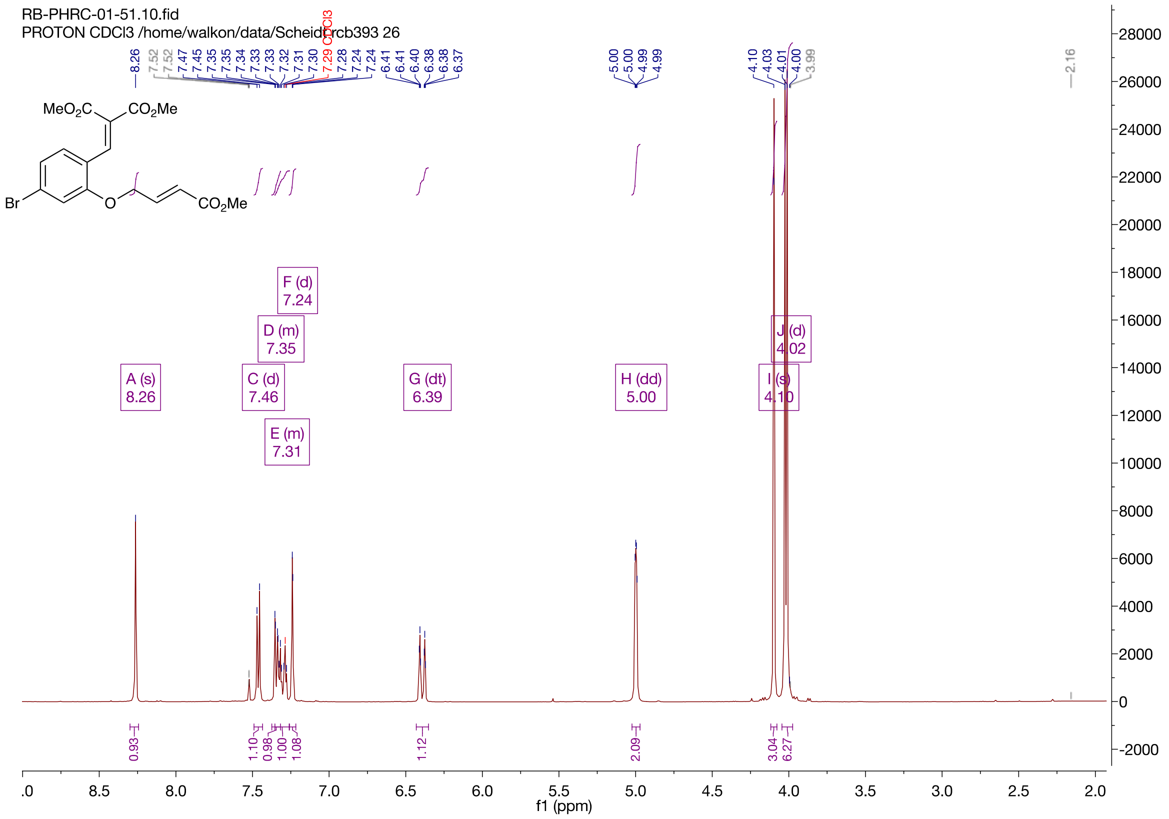
RB-PHRC-01-51.10.fid
PROTON CDCl3 /home/walkon/data/Scheidt rcb393 26
MeO$_2$C CO$_2$Me
Br O CO$_2$Me
A (s) 8.26
C (d) 7.46
D (m) 7.35
E (m) 7.31
F (d) 7.24
G (dt) 6.39
H (dd) 5.00
I (s) 4.10
J (d) 4.02
f1 (ppm)

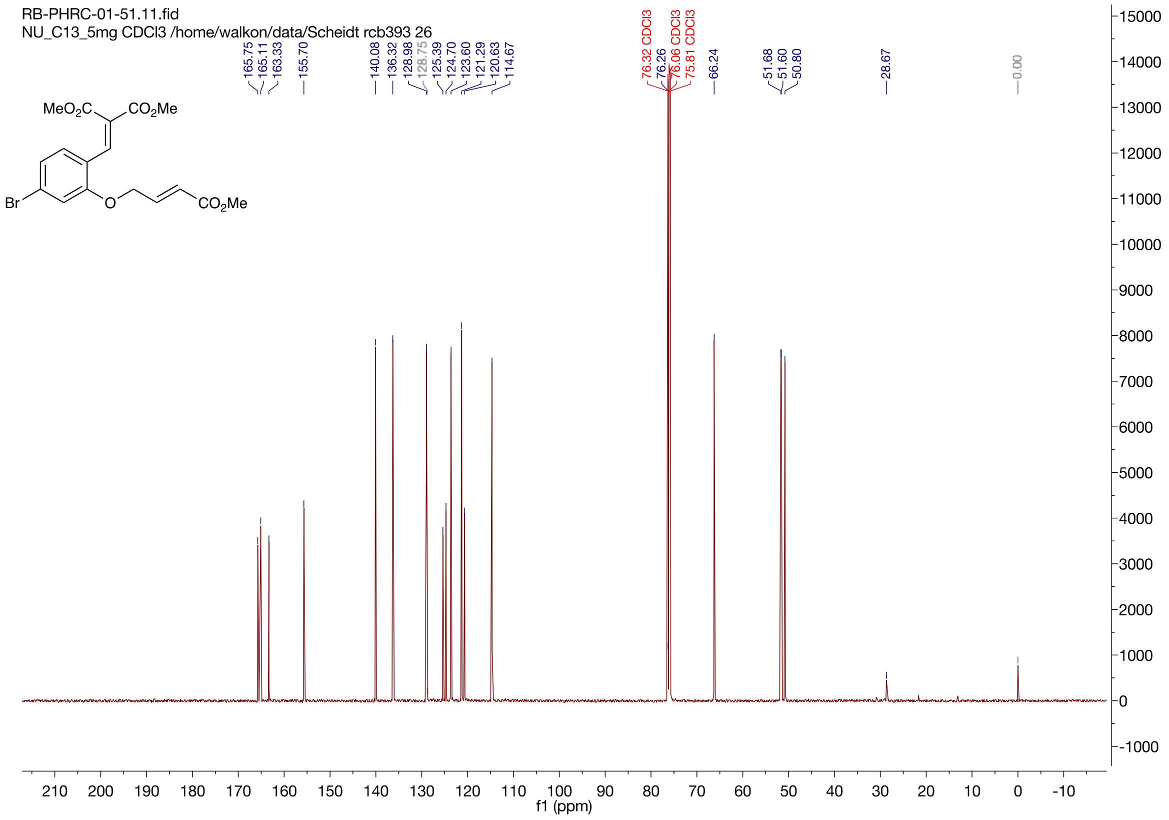
RB-PHRC-01-51.11.fid
NU_C13_5mg CDCl3 /home/walkon/data/Scheidt rcb393 26
MeO$_2$C CO$_2$Me
Br O CO$_2$Me
165.75 165.11 163.33 155.70 140.08 136.32 128.98 128.75 125.39 124.70 123.60 121.29 120.63 114.67
76.32 CDCl3 76.26 76.06 CDCl3 75.81 CDCl3 66.24 51.68 51.60 50.80 28.67 0.00
f1 (ppm)

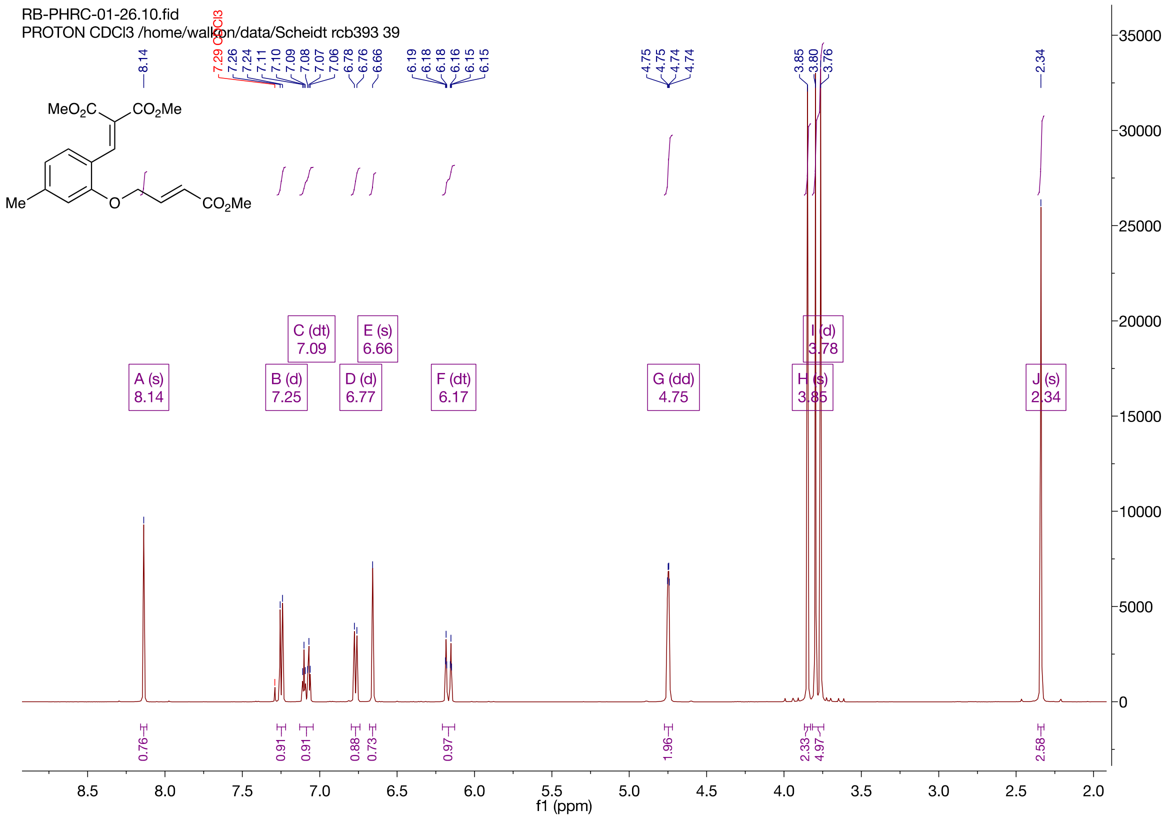

RB-PHRC-01-26.10.fid
PROTON CDCl3 /home/walkon/data/Scheidt rcb393 39
7.29 CDCl3
8.14
7.26 7.24 7.11 7.10 7.09 7.08 7.07 7.06 6.78 6.76 6.66
6.19 6.18 6.18 6.16 6.15 6.15
4.75 4.75 4.74 4.74
3.85 3.80 3.76
2.34
A (s) 8.14
B (d) 7.25
C (dt) 7.09
D (d) 6.77
E (s) 6.66
F (dt) 6.17
G (dd) 4.75
H (s) 3.85
I (d) 3.78
J (s) 2.34
0.76 0.91 0.91 0.88 0.73 0.97 1.96 2.33 4.97 2.58
f1 (ppm)

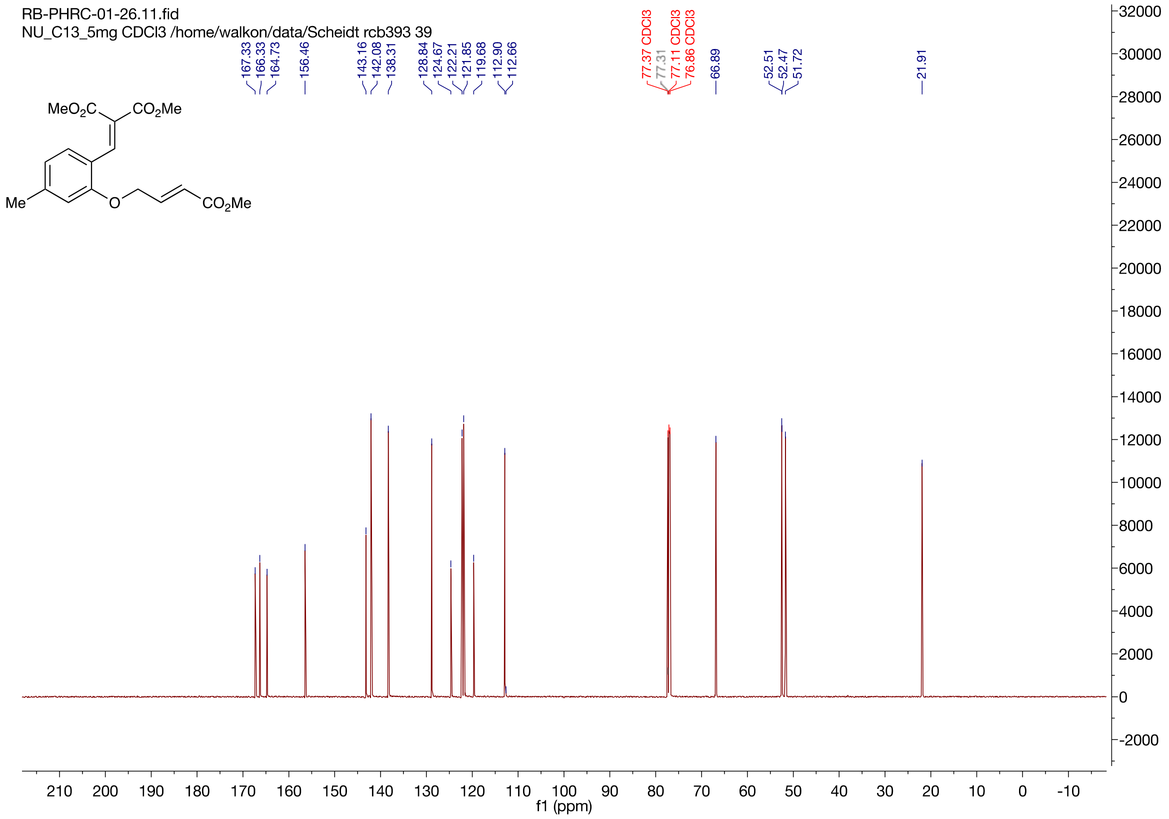

RB-PHRC-01-26.11.fid
NU_C13_5mg CDCl3 /home/walkon/data/Scheidt rcb393 39
167.33 166.33 164.73 156.46 143.16 142.08 138.31 128.84 124.67 122.21 121.85 119.68 112.90 112.66
77.37 CDCl3 77.31 77.11 CDCl3 76.86 CDCl3
66.89 52.51 52.47 51.72 21.91
f1 (ppm)

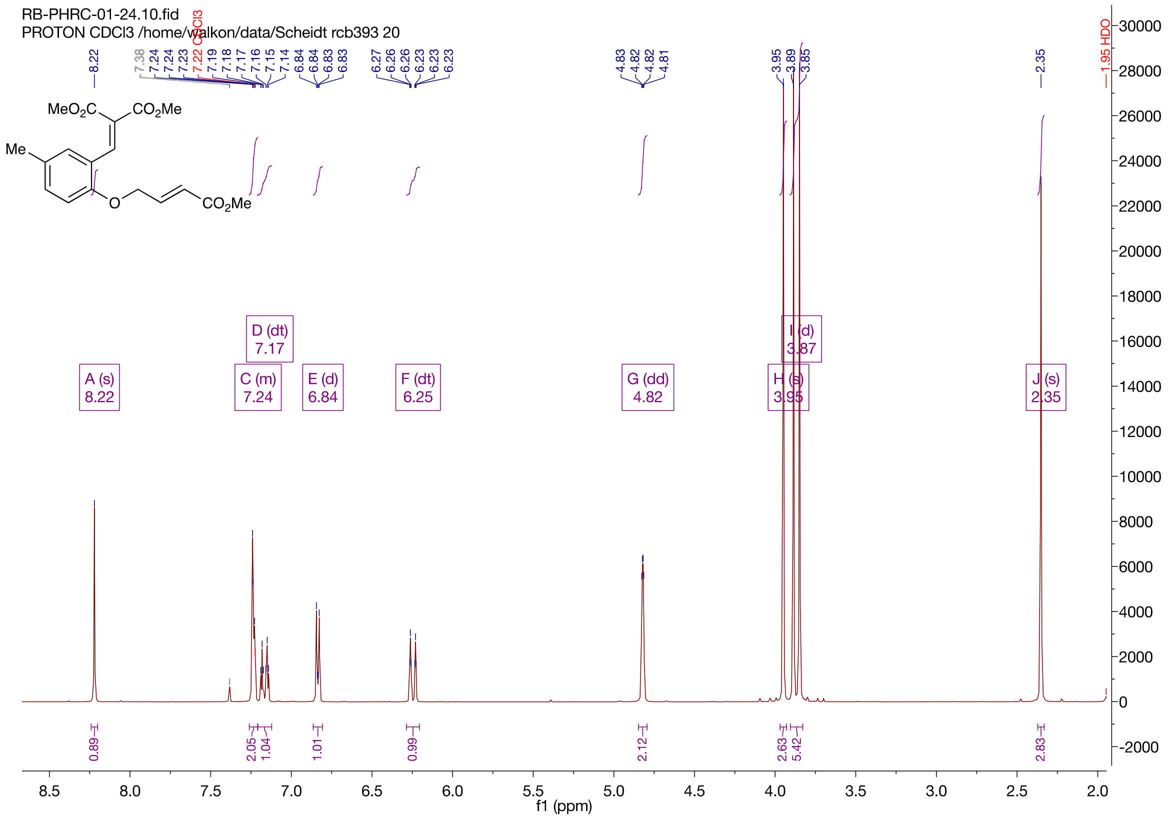
RB-PHRC-01-24.10.fid
PROTON CDCl3 /home/walkon/data/Scheidt rcb393 20
A (s) 8.22
C (m) 7.24
D (dt) 7.17
E (d) 6.84
F (dt) 6.25
G (dd) 4.82
H (s) 3.95
I (d) 3.87
J (s) 2.35
f1 (ppm)

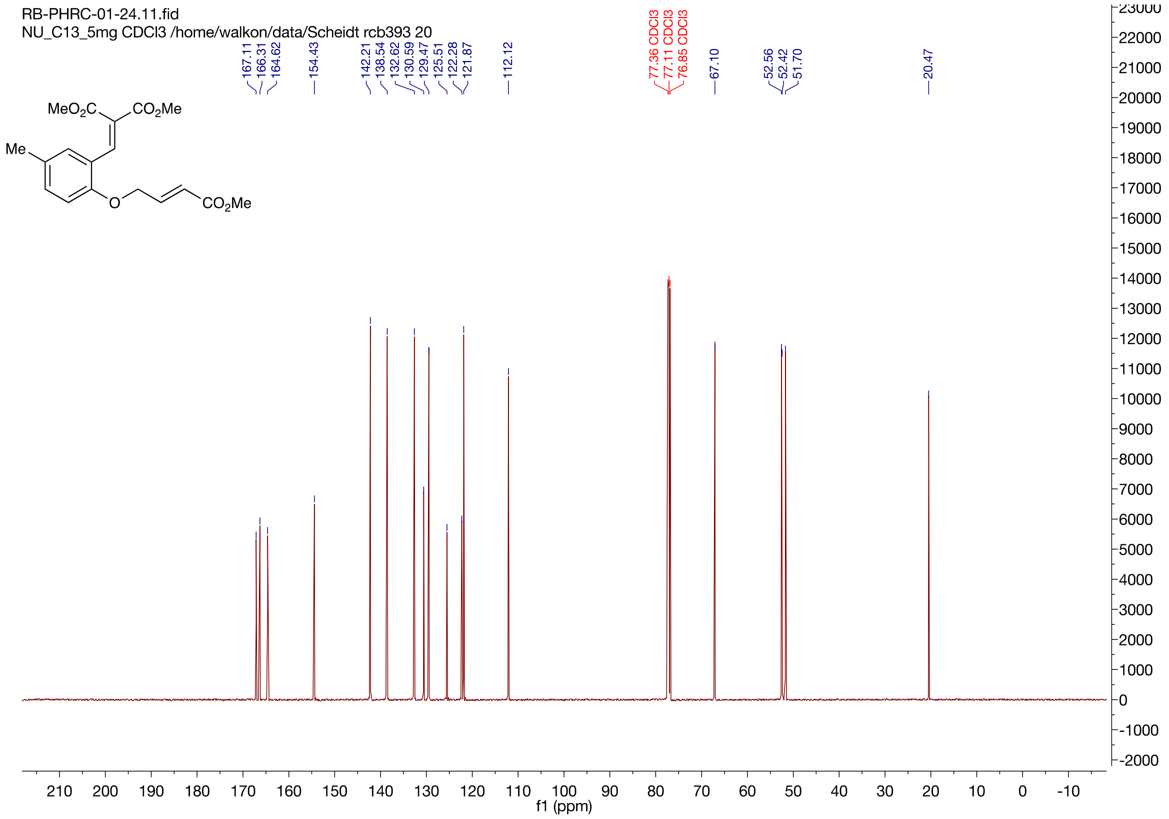
RB-PHRC-01-24.11.fid
NU_C13_5mg CDCl3 /home/walkon/data/Scheidt rcb393 20
f1 (ppm)

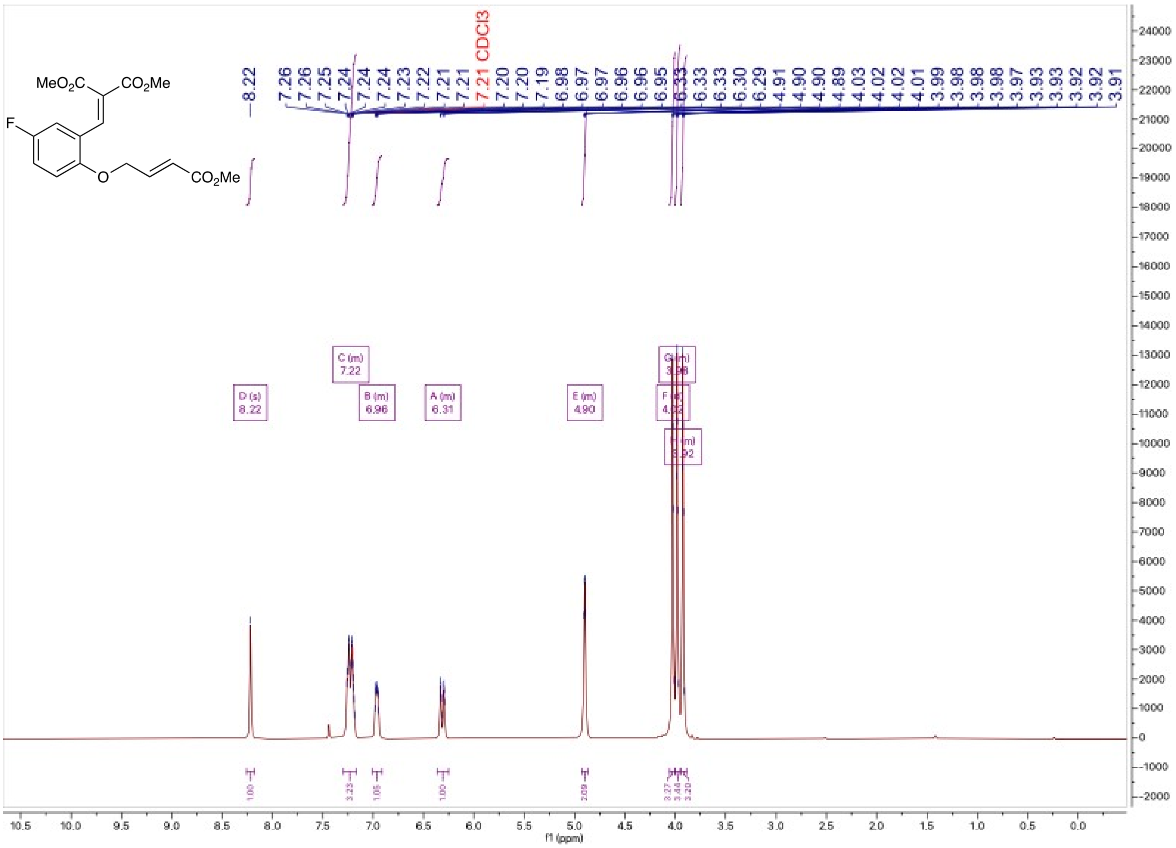

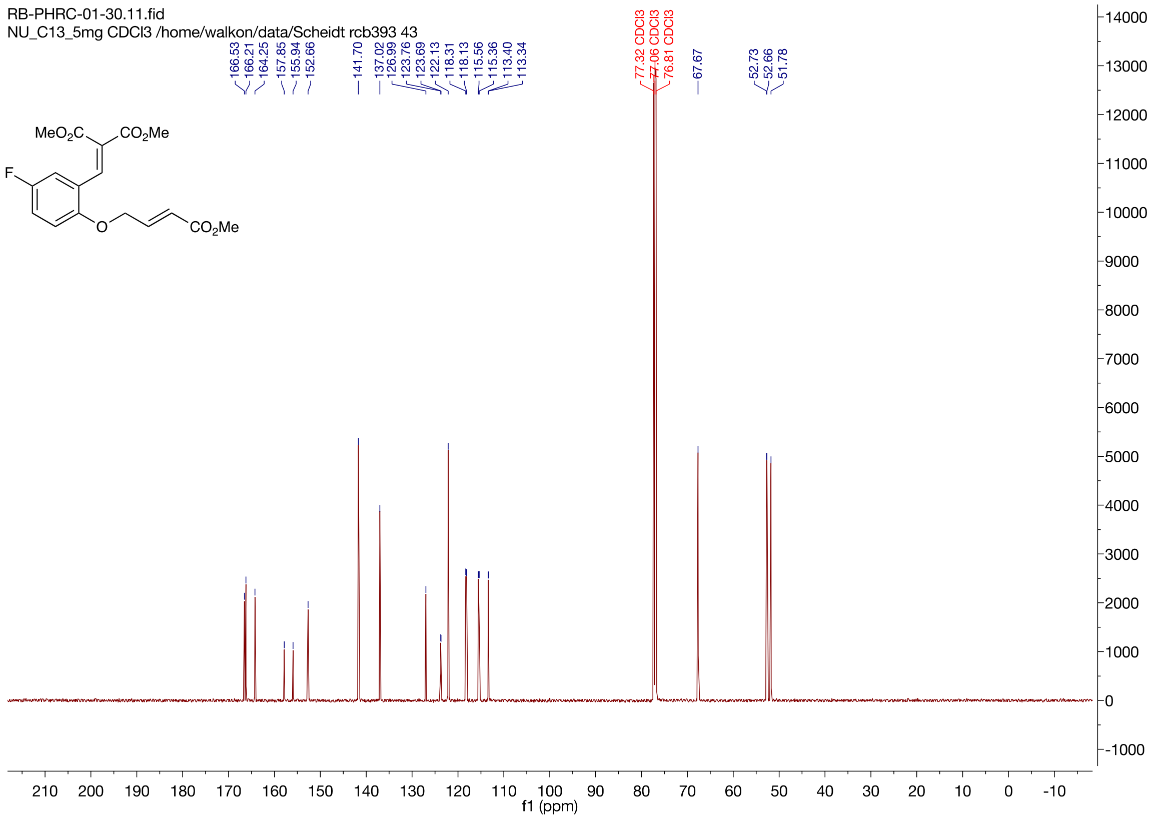

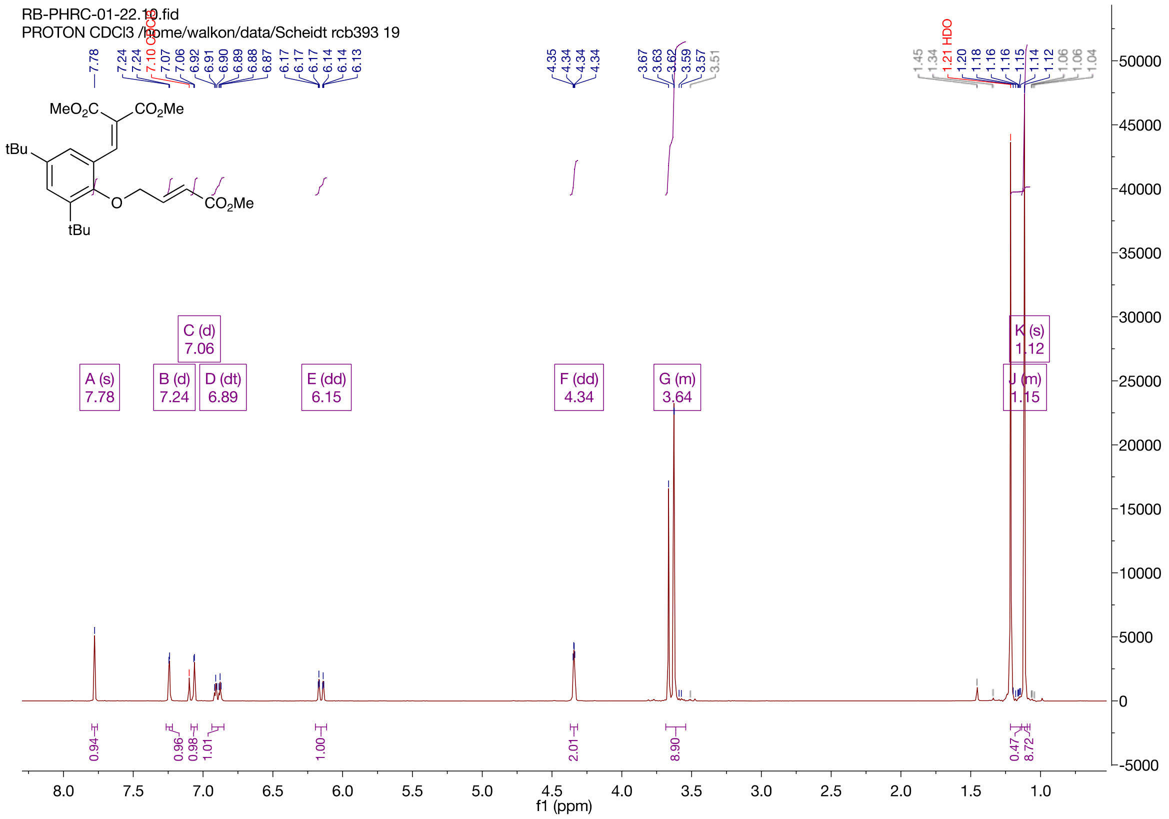
RB-PHRC-01-22.10.fid
PROTON CDCl3 /home/walkon/data/Scheidt rcb393 19
MeO2C CO2Me
tBu
O
CO2Me
tBu
A (s) 7.78
B (d) 7.24
C (d) 7.06
D (dt) 6.89
E (dd) 6.15
F (dd) 4.34
G (m) 3.64
J (m) 1.15
K (s) 1.12
f1 (ppm)

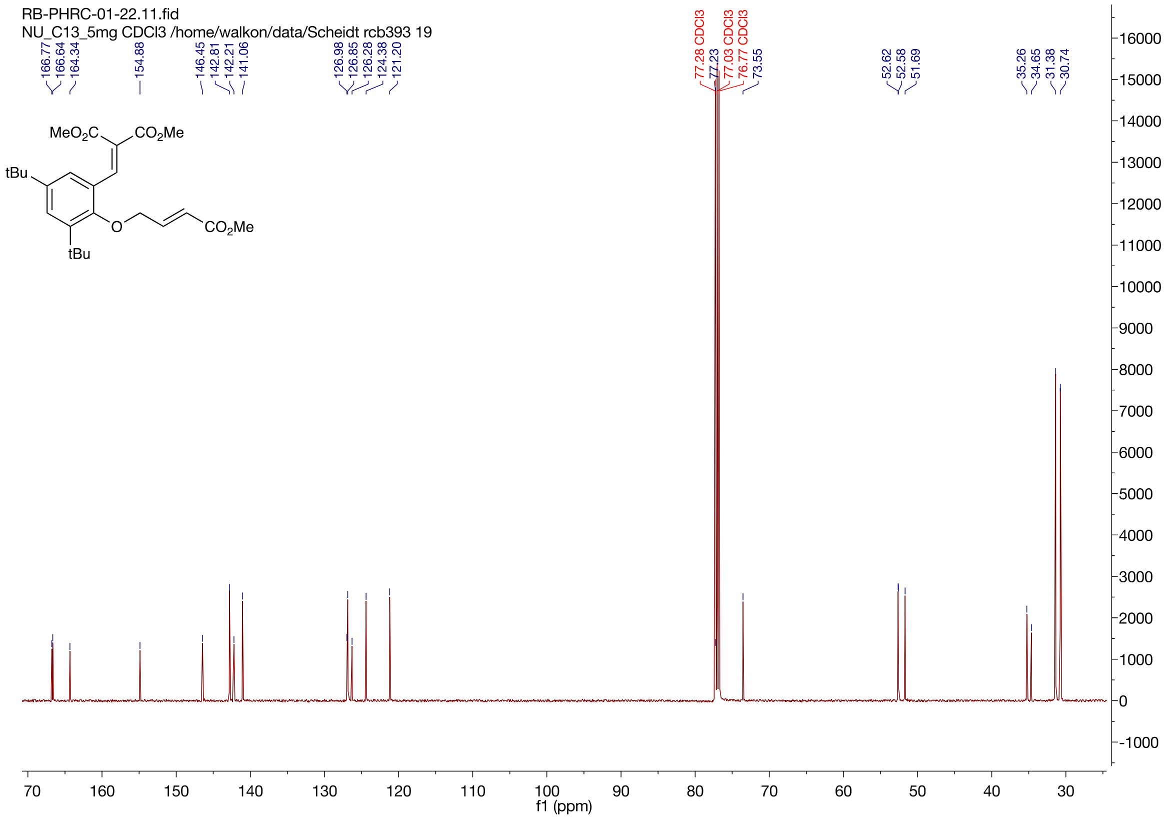
RB-PHRC-01-22.11.fid
NU_C13_5mg CDCl3 /home/walkon/data/Scheidt rcb393 19
MeO2C CO2Me
tBu
O
CO2Me
tBu
f1 (ppm)

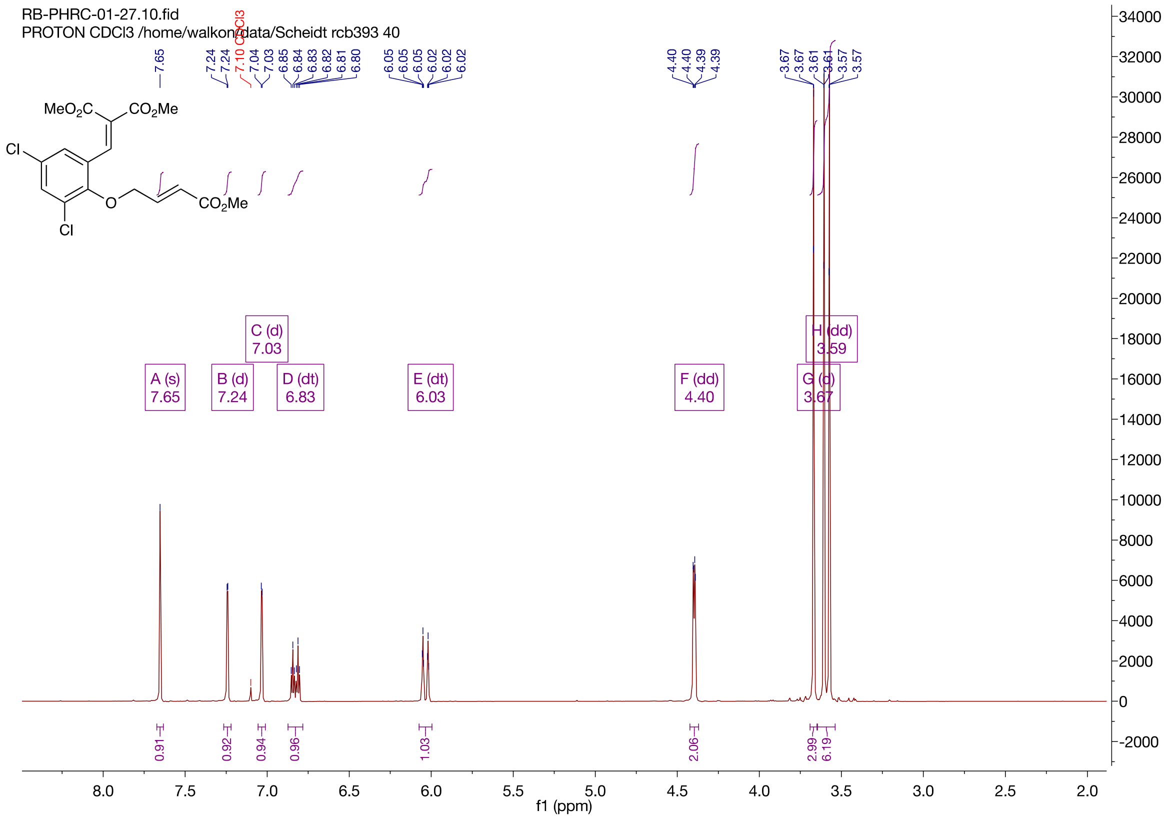
RB-PHRC-01-27.10.fid
PROTON CDCl3 /home/walkon/data/Scheidt rcb393 40
f1 (ppm)

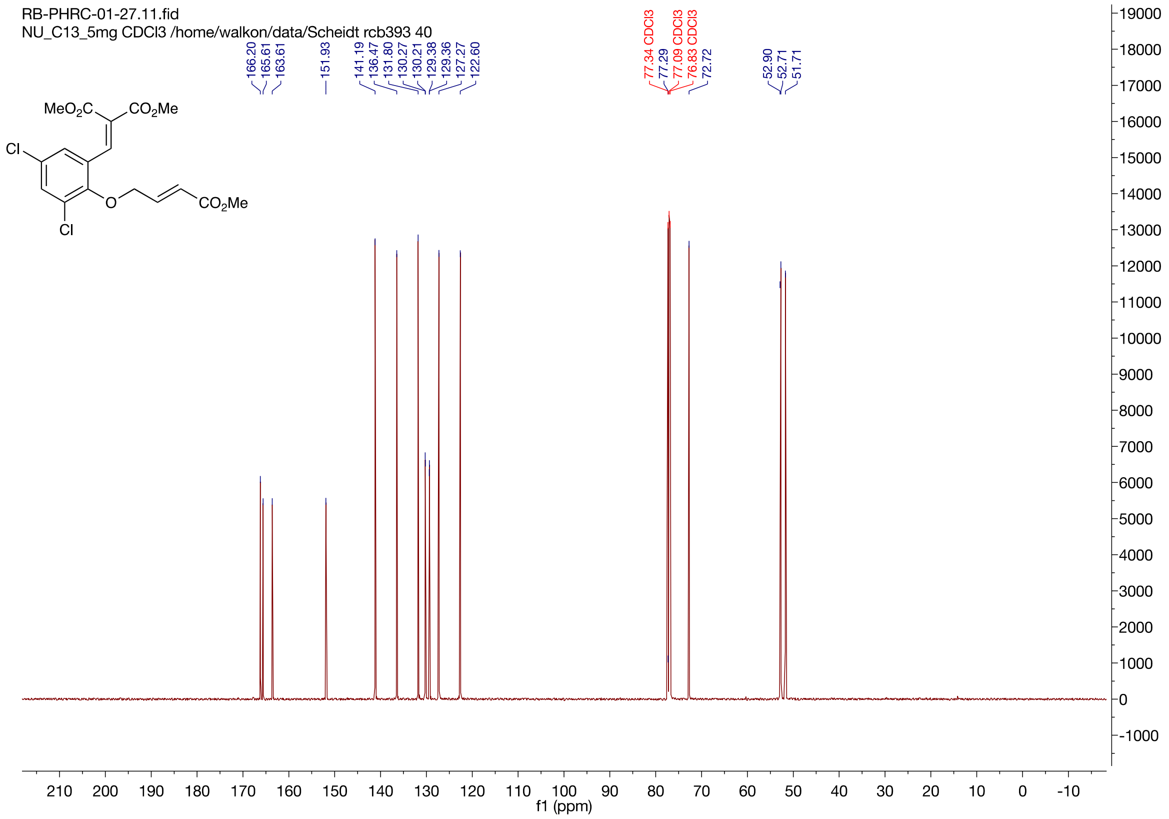
RB-PHRC-01-27.11.fid
NU_C13_5mg CDCl3 /home/walkon/data/Scheidt rcb393 40
f1 (ppm)

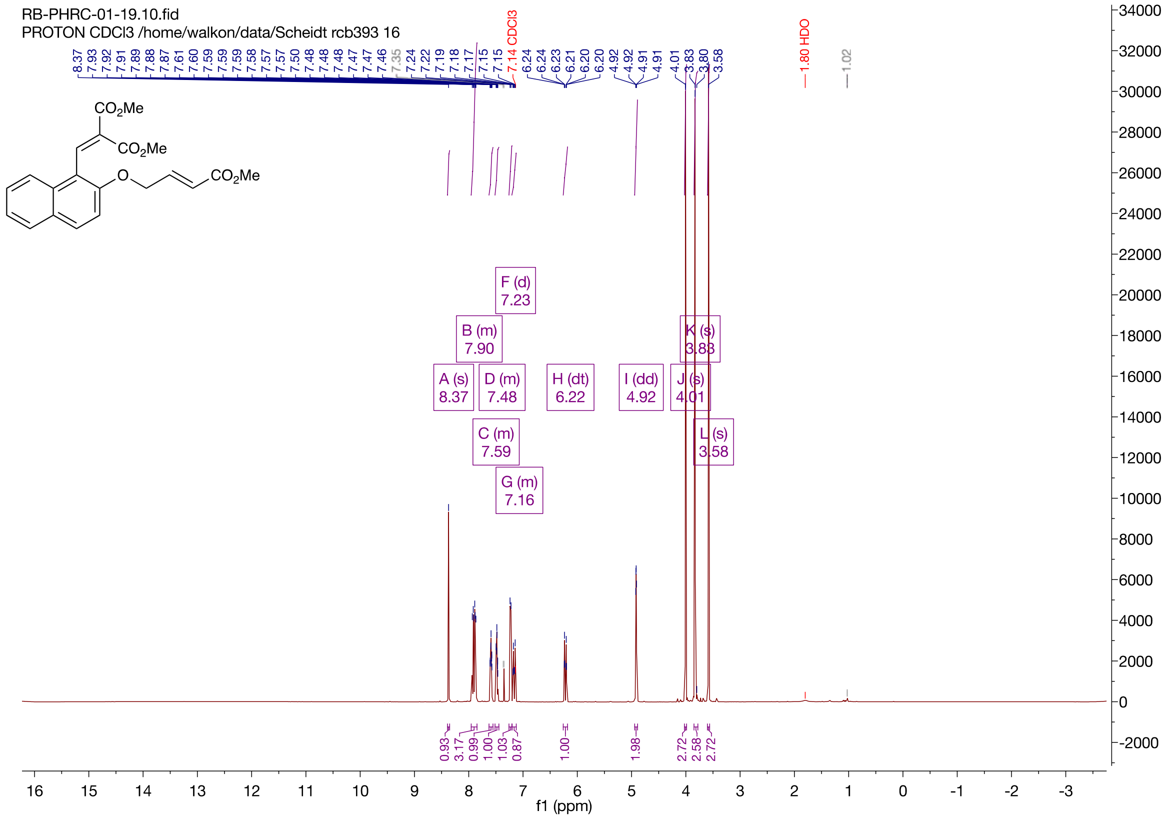
RB-PHRC-01-19.10.fid
PROTON CDCl3 /home/walkon/data/Scheidt rcb393 16
f1 (ppm)

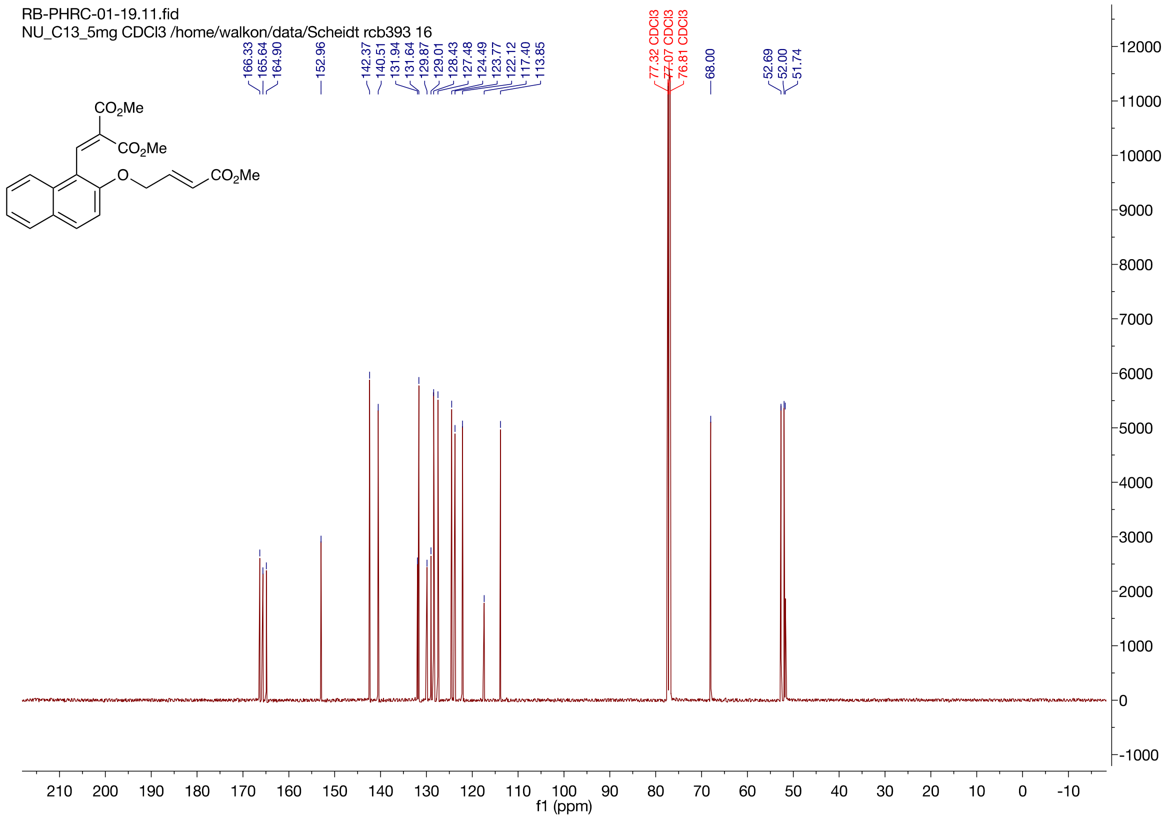
RB-PHRC-01-19.11.fid
NU_C13_5mg CDCl3 /home/walkon/data/Scheidt rcb393 16
f1 (ppm)

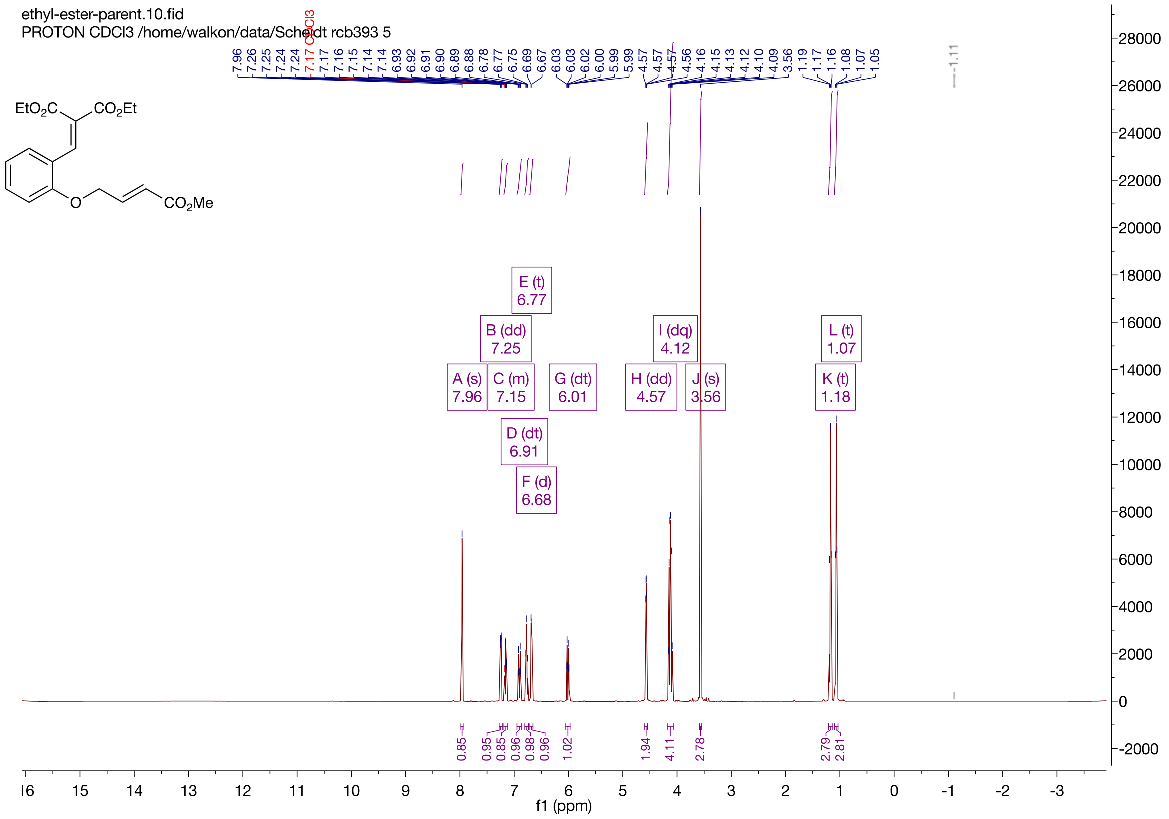
ethyl-ester-parent.10.fid
PROTON CDCl3 /home/walkon/data/Scheidt rcb393 5
EtO2C
CO2Et
O
CO2Me
A (s) 7.96
B (dd) 7.25
C (m) 7.15
D (dt) 6.91
E (t) 6.77
F (d) 6.68
G (dt) 6.01
H (dd) 4.57
I (dq) 4.12
J (s) 3.56
K (t) 1.18
L (t) 1.07
f1 (ppm)

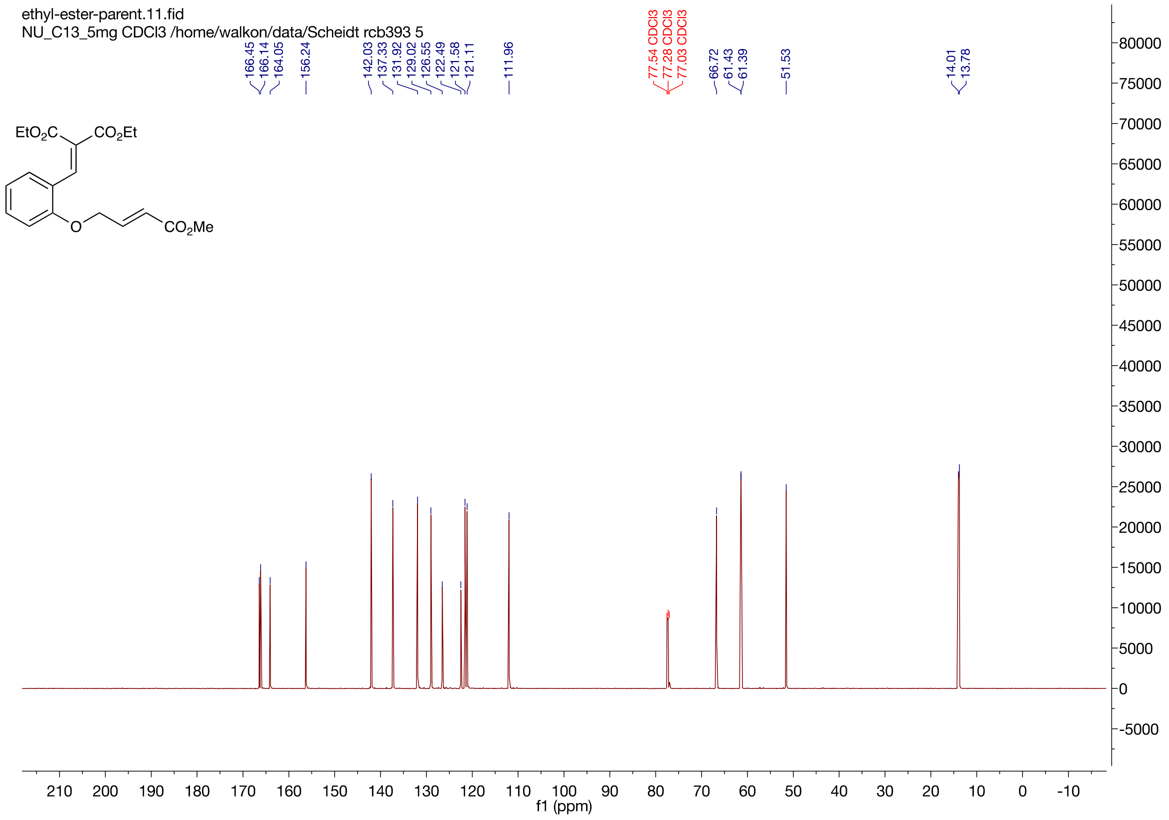
ethyl-ester-parent.11.fid
NU_C13_5mg CDCl3 /home/walkon/data/Scheidt rcb393 5
EtO2C
CO2Et
O
CO2Me
166.45
166.14
164.05
156.24
142.03
137.33
131.92
129.02
126.55
122.49
121.58
121.11
111.96
77.54 CDCl3
77.28 CDCl3
77.03 CDCl3
66.72
61.43
61.39
51.53
14.01
13.78
f1 (ppm)

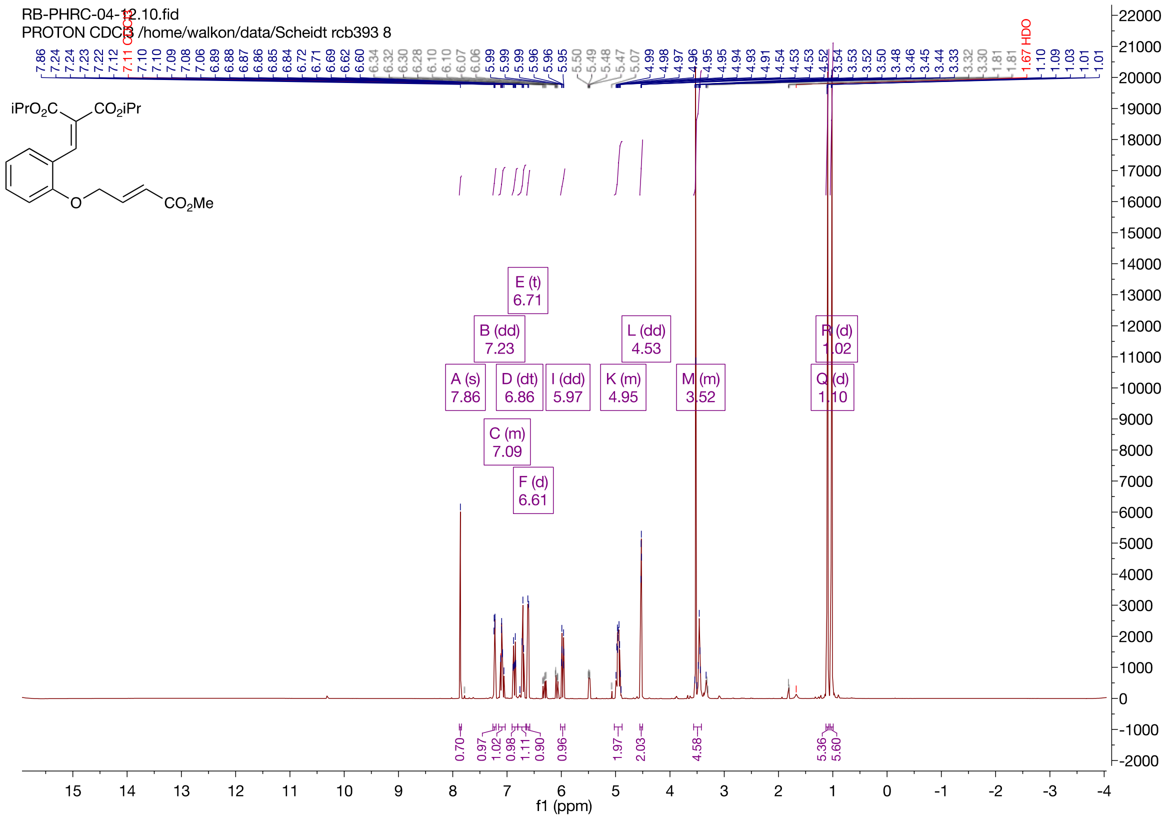
RB-PHRC-04-12.10.fid
PROTON CDCl3 /home/walkon/data/Scheidt rcb393 8
iPrO2C
CO2iPr
O
CO2Me
A (s) 7.86
B (dd) 7.23
C (m) 7.09
D (dt) 6.86
E (t) 6.71
F (d) 6.61
I (dd) 5.97
K (m) 4.95
L (dd) 4.53
M (m) 3.52
Q (d) 1.10
R (d) 1.02
f1 (ppm)

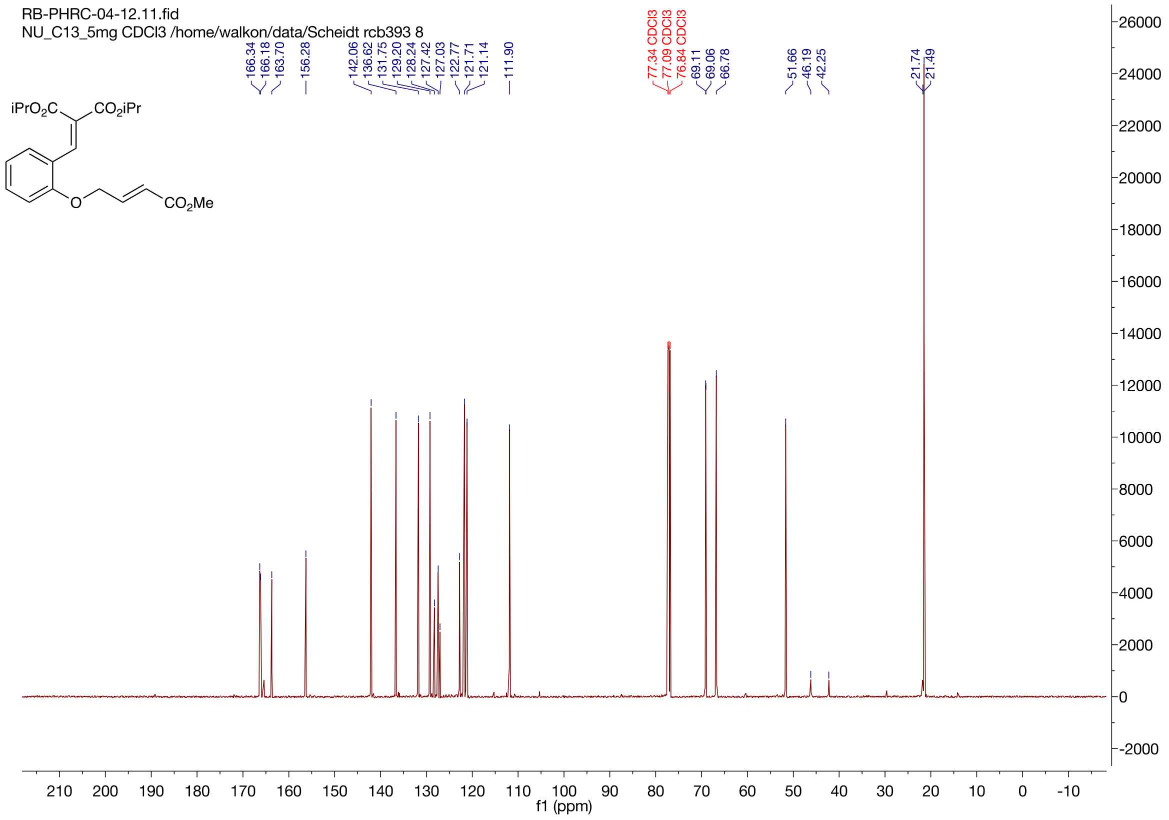
RB-PHRC-04-12.11.fid
NU_C13_5mg CDCl3 /home/walkon/data/Scheidt rcb393 8
166.34
166.18
163.70
156.28
142.06
136.62
131.75
129.20
128.24
127.42
127.03
122.77
121.71
121.14
111.90
77.34 CDCl3
77.09 CDCl3
76.84 CDCl3
69.11
69.06
66.78
51.66
46.19
42.25
21.74
21.49
iPrO2C
CO2iPr
O
CO2Me
f1 (ppm)

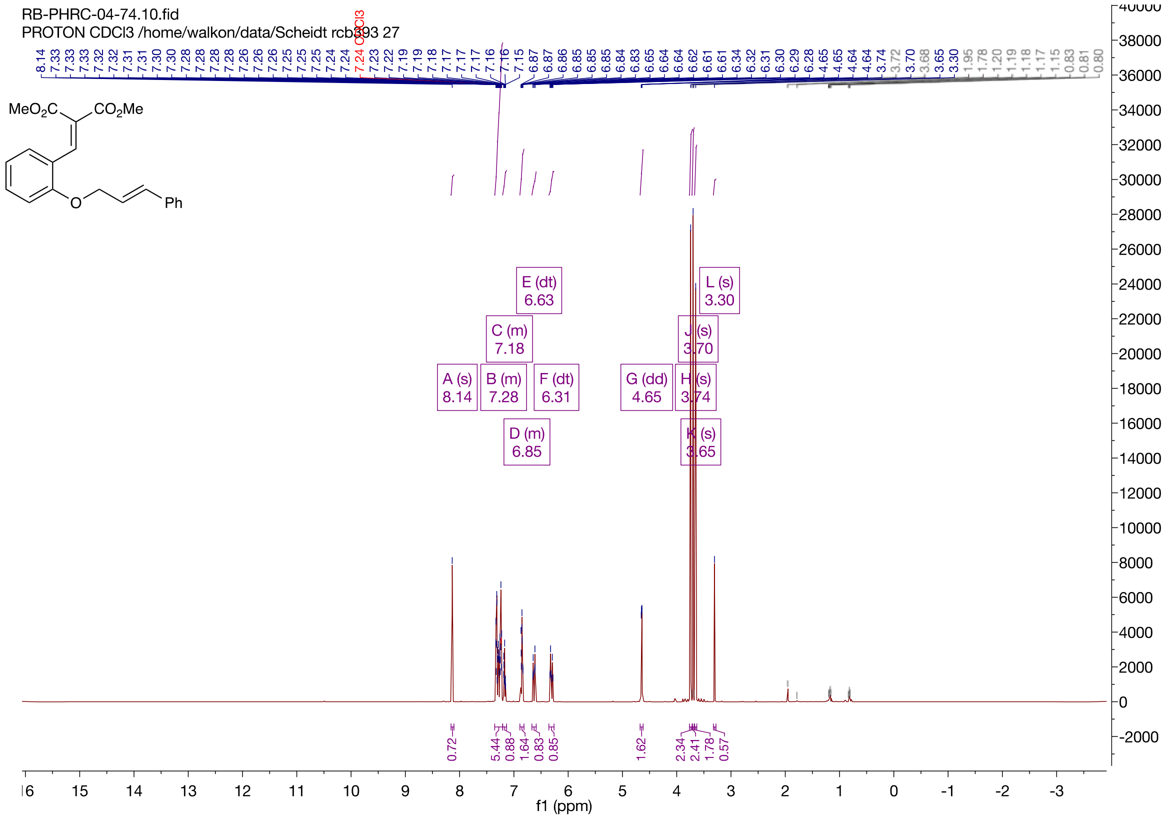
RB-PHRC-04-74.10.fid
PROTON CDCl3 /home/walkon/data/Scheidt rcb393 27
MeO2C CO2Me
O Ph
A (s) 8.14
B (m) 7.28
C (m) 7.18
D (m) 6.85
E (dt) 6.63
F (dt) 6.31
G (dd) 4.65
H (s) 3.74
J (s) 3.70
K (s) 3.65
L (s) 3.30
f1 (ppm)

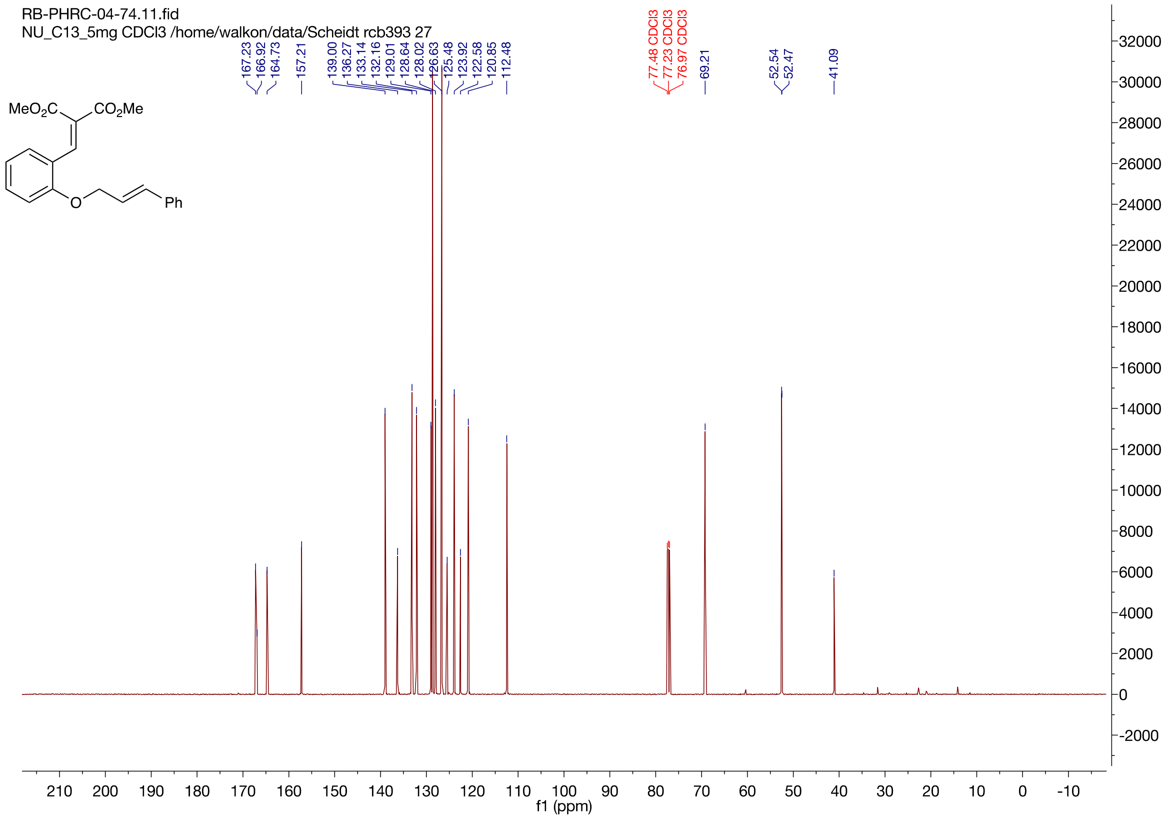
RB-PHRC-04-74.11.fid
NU_C13_5mg CDCl3 /home/walkon/data/Scheidt rcb393 27
167.23
166.92
164.73
157.21
139.00
136.27
133.14
132.16
129.01
128.64
128.02
126.63
125.48
123.92
122.58
120.85
112.48
77.48 CDCl3
77.23 CDCl3
76.97 CDCl3
69.21
52.54
52.47
41.09
MeO2C CO2Me
O Ph
f1 (ppm)

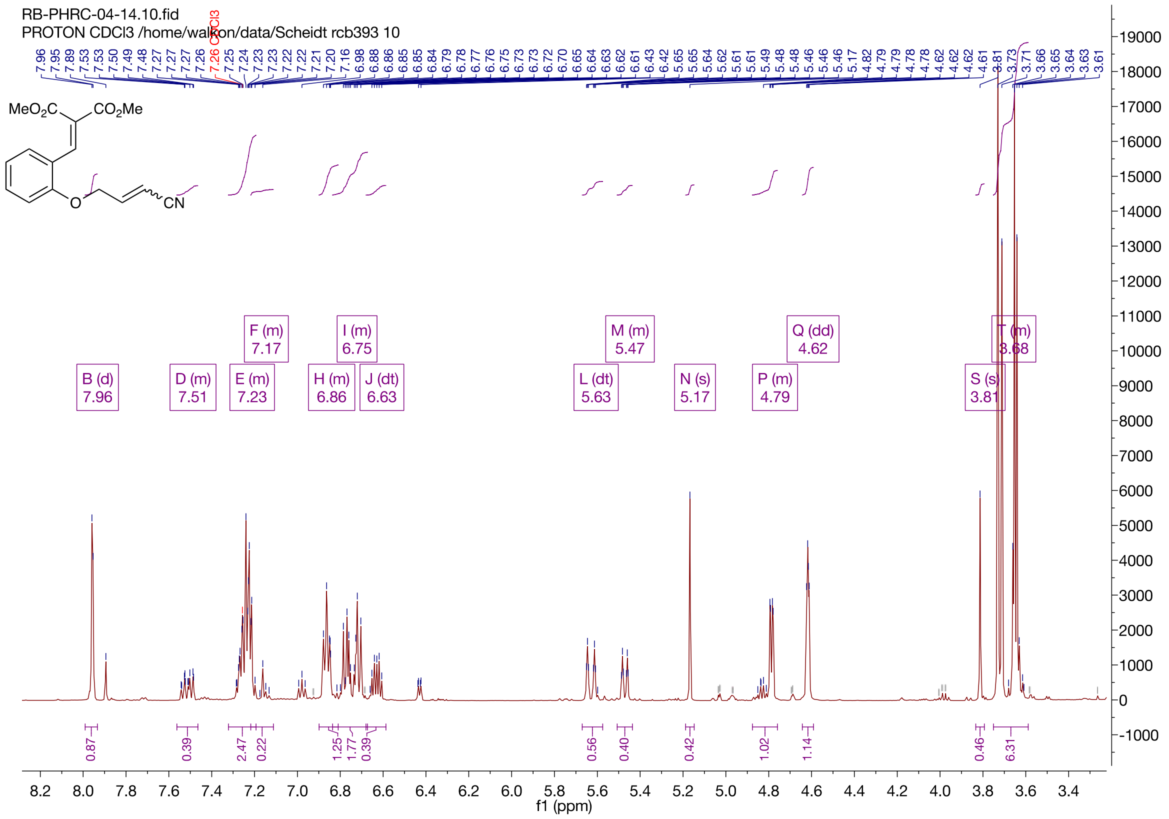
RB-PHRC-04-14.10.fid
PROTON CDCl3 /home/walkon/data/Scheidt rcb393 10
MeO2C
CO2Me
O
CN
B (d) 7.96
D (m) 7.51
E (m) 7.23
F (m) 7.17
H (m) 6.86
I (m) 6.75
J (dt) 6.63
L (dt) 5.63
M (m) 5.47
N (s) 5.17
P (m) 4.79
Q (dd) 4.62
S (s) 3.81
T (m) 3.68
f1 (ppm)

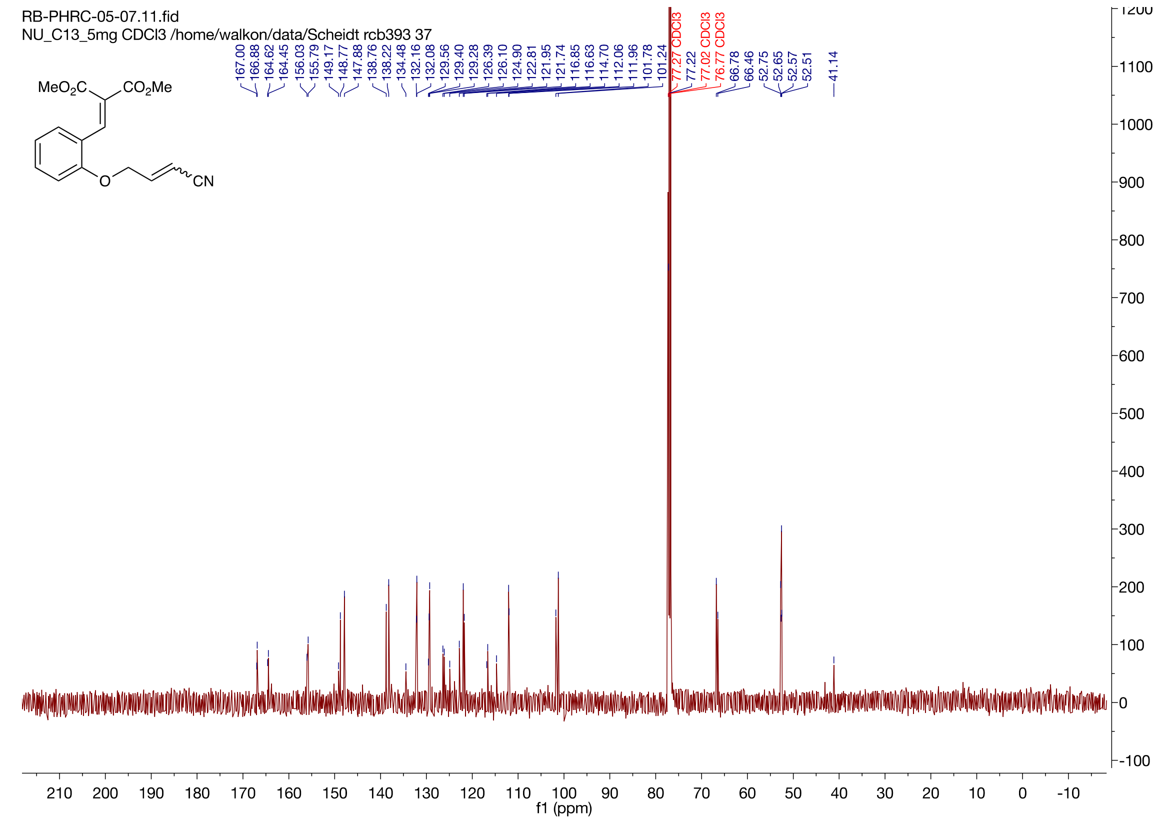
RB-PHRC-05-07.11.fid
NU_C13_5mg CDCl3 /home/walkon/data/Scheidt rcb393 37
167.00
166.88
164.62
164.45
156.03
155.79
149.17
148.77
147.88
138.76
138.22
134.48
132.16
132.08
129.56
129.40
129.28
126.39
126.10
124.90
122.81
121.95
121.74
116.85
116.63
114.70
112.06
111.96
101.78
101.24
77.27 CDCl3
77.22
77.02 CDCl3
76.77 CDCl3
66.78
66.46
52.75
52.65
52.57
52.51
41.14
MeO2C
CO2Me
O
CN
f1 (ppm)

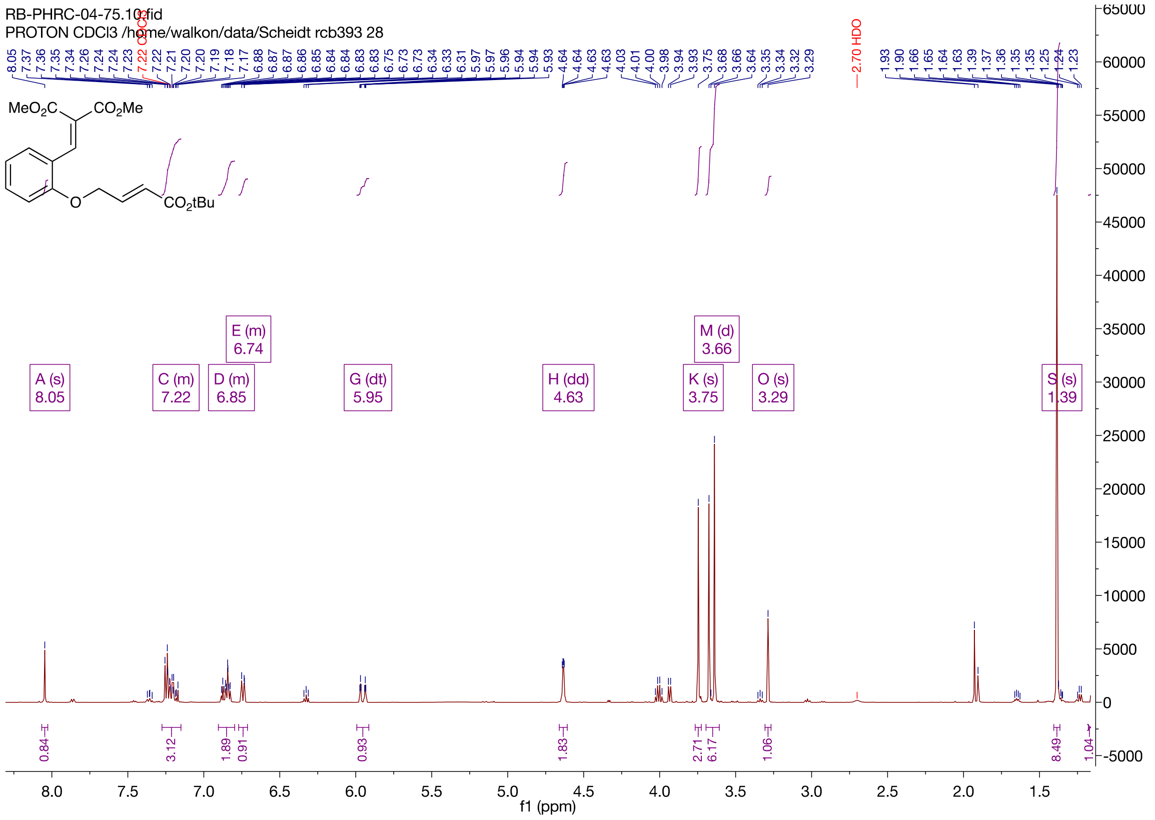
RB-PHRC-04-75.10.fid
PROTON CDCl3 /home/walkon/data/Scheidt rcb393 28
MeO2C CO2Me
O
CO2tBu
f1 (ppm)

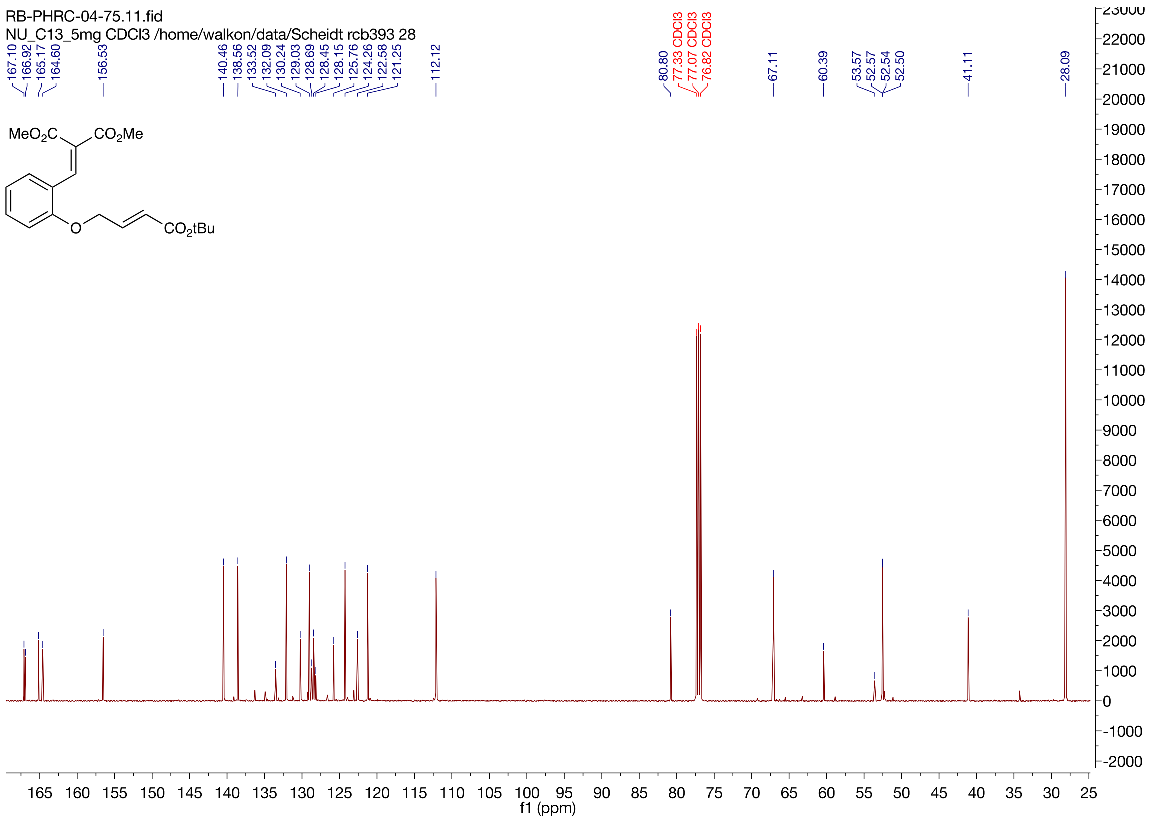
RB-PHRC-04-75.11.fid
NU_C13_5mg CDCl3 /home/walkon/data/Scheidt rcb393 28
MeO2C CO2Me
O
CO2tBu
f1 (ppm)

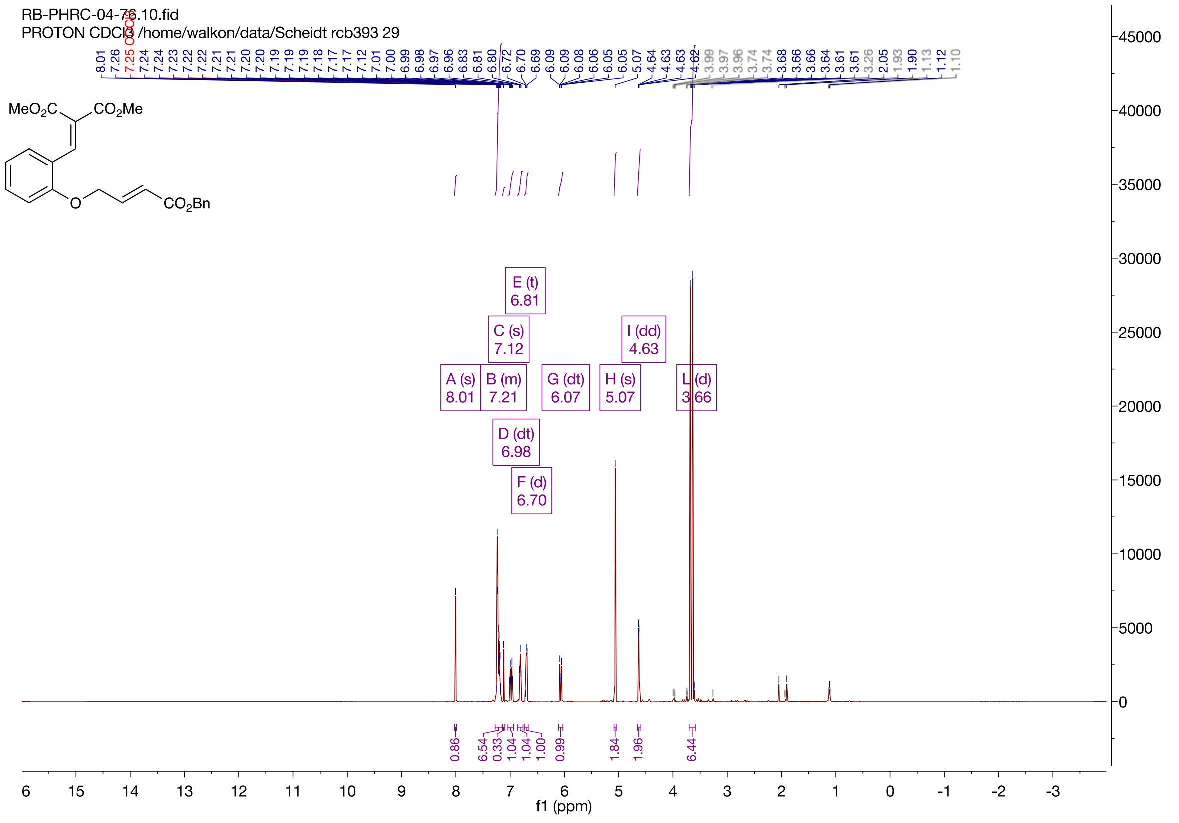

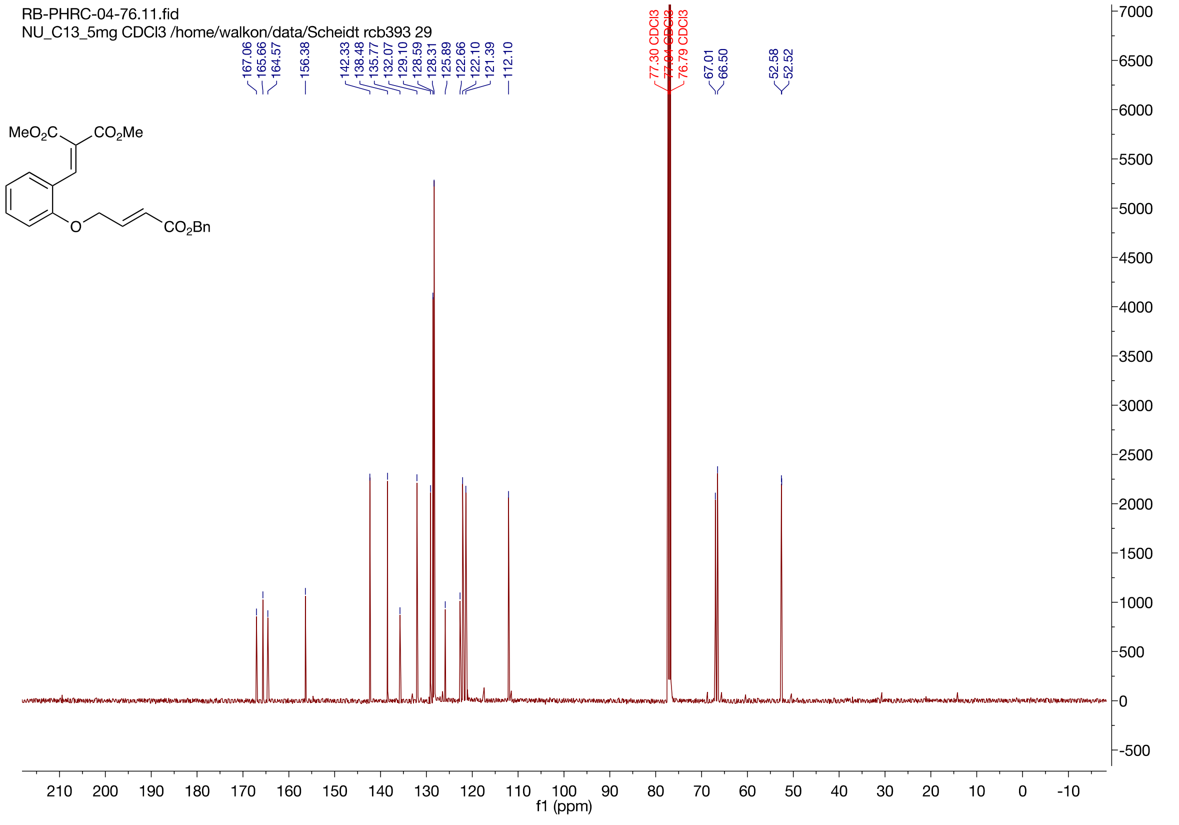

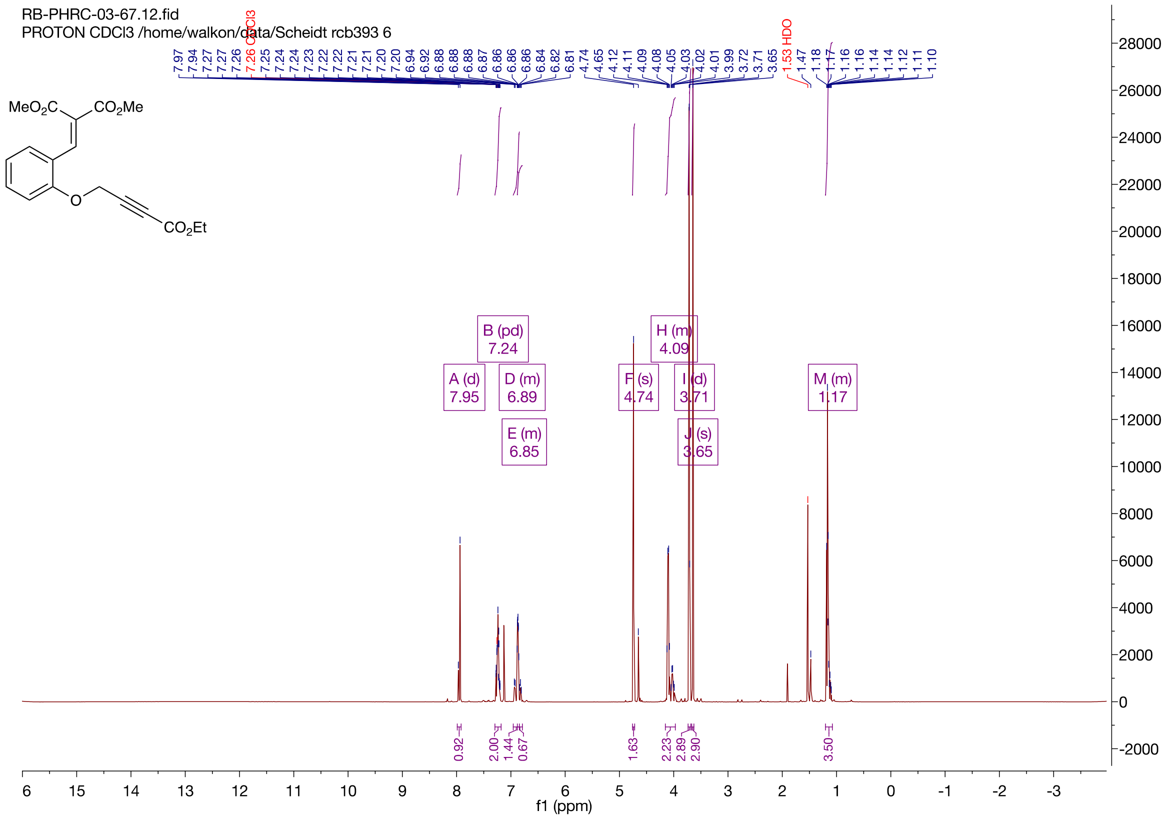
RB-PHRC-03-67.12.fid
PROTON CDCl3 /home/walkon/data/Scheidt rcb393 6
MeO2C
CO2Me
O
CO2Et
A (d) 7.95
B (pd) 7.24
D (m) 6.89
E (m) 6.85
F (s) 4.74
H (m) 4.09
I (d) 3.71
J (s) 3.65
M (m) 1.17
f1 (ppm)

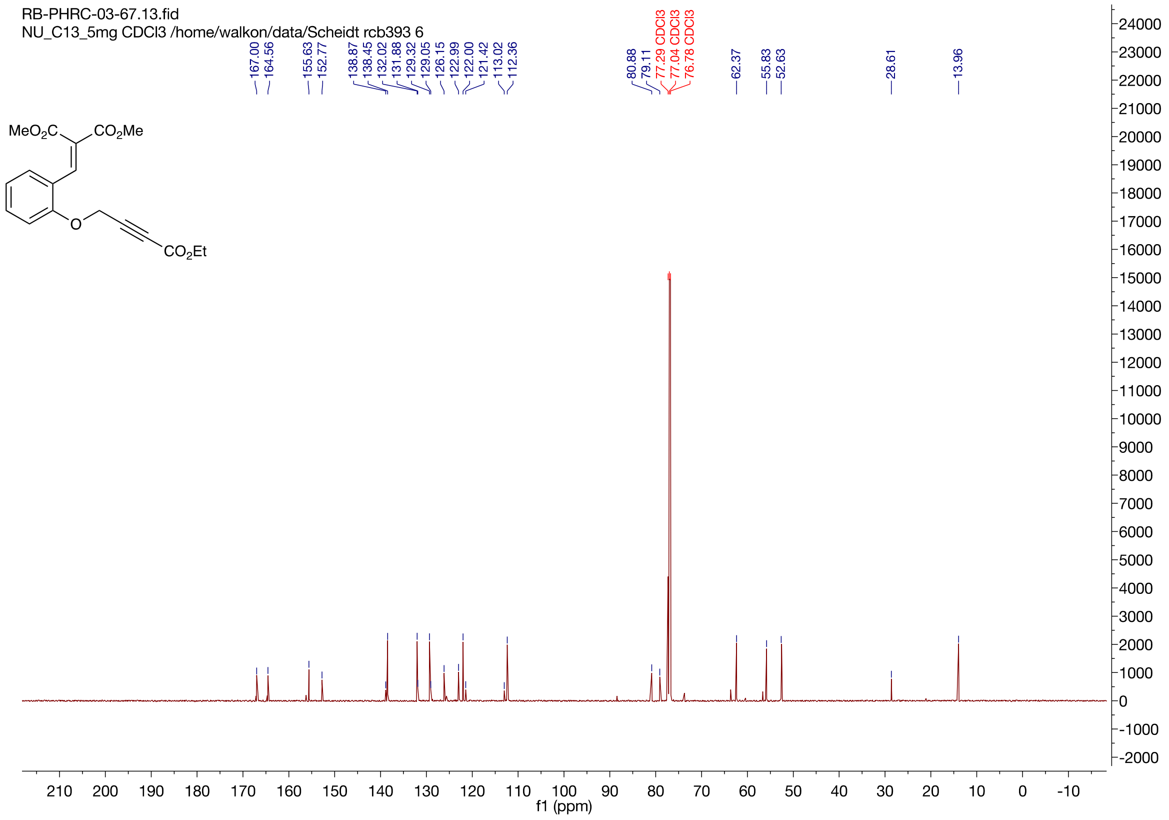
RB-PHRC-03-67.13.fid
NU_C13_5mg CDCl3 /home/walkon/data/Scheidt rcb393 6
167.00
164.56
155.63
152.77
138.87
138.45
132.02
131.88
129.32
129.05
126.15
122.99
122.00
121.42
113.02
112.36
80.88
79.11
77.29 CDCl3
77.04 CDCl3
76.78 CDCl3
62.37
55.83
52.63
28.61
13.96
MeO2C
CO2Me
O
CO2Et
f1 (ppm)

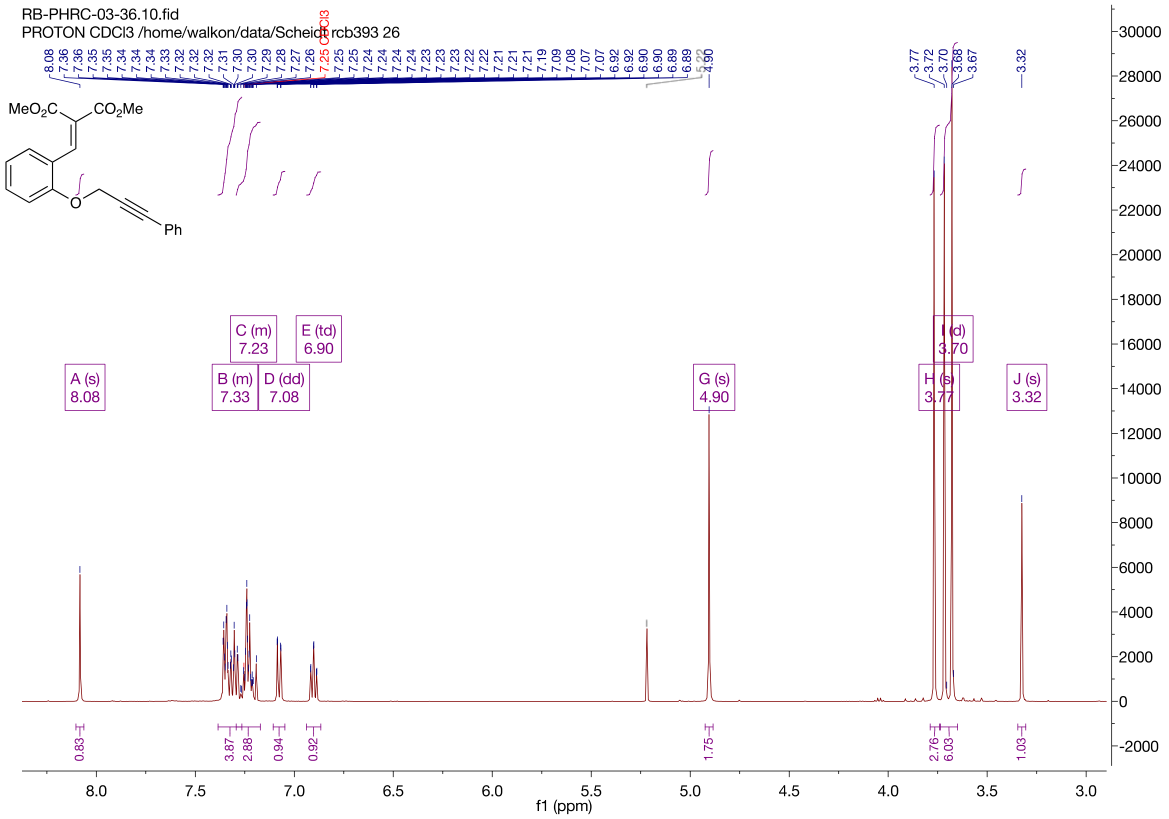
RB-PHRC-03-36.10.fid
PROTON CDCl3 /home/walkon/data/Scheidt rcb393 26
MeO2C
CO2Me
O
Ph
A (s) 8.08
B (m) 7.33
C (m) 7.23
D (dd) 7.08
E (td) 6.90
G (s) 4.90
H (s) 3.77
I (d) 3.70
J (s) 3.32
f1 (ppm)

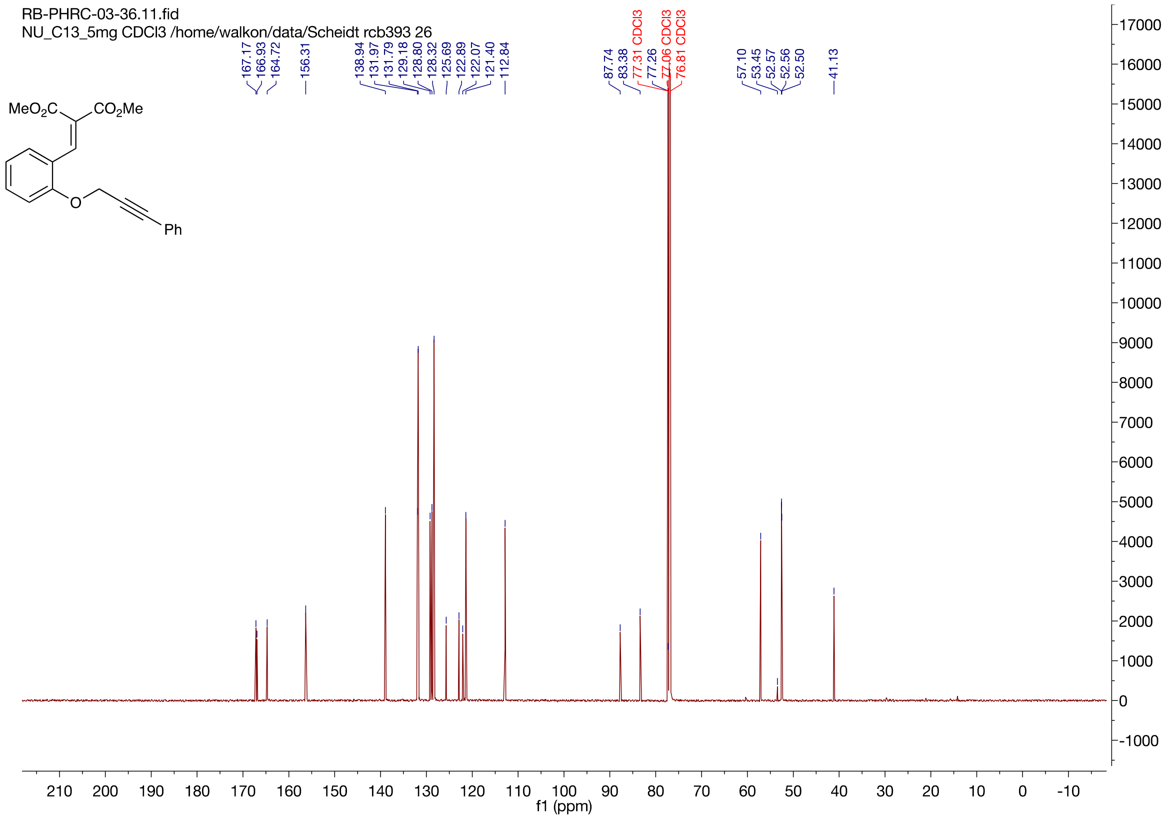
RB-PHRC-03-36.11.fid
NU_C13_5mg CDCl3 /home/walkon/data/Scheidt rcb393 26
167.17
166.93
164.72
156.31
138.94
131.97
131.79
129.18
128.80
128.32
125.69
122.89
122.07
121.40
112.84
87.74
83.38
77.31 CDCl3
77.26
77.06 CDCl3
76.81 CDCl3
57.10
53.45
52.57
52.56
52.50
41.13
MeO2C
CO2Me
O
Ph
f1 (ppm)

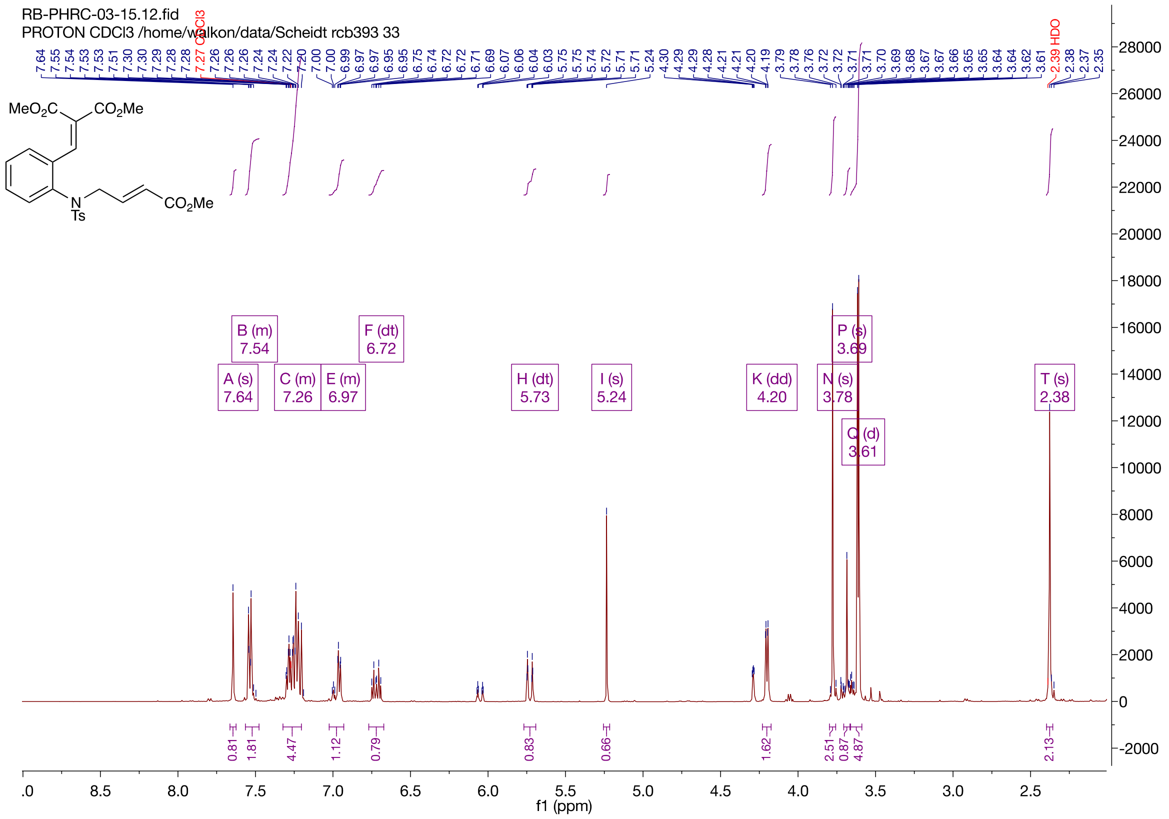
RB-PHRC-03-15.12.fid
PROTON CDCl3 /home/walkon/data/Scheidt rcb393 33
MeO2C CO2Me
N Ts CO2Me
A (s) 7.64
B (m) 7.54
C (m) 7.26
E (m) 6.97
F (dt) 6.72
H (dt) 5.73
I (s) 5.24
K (dd) 4.20
N (s) 3.78
P (s) 3.69
Q (d) 3.61
T (s) 2.38
f1 (ppm)

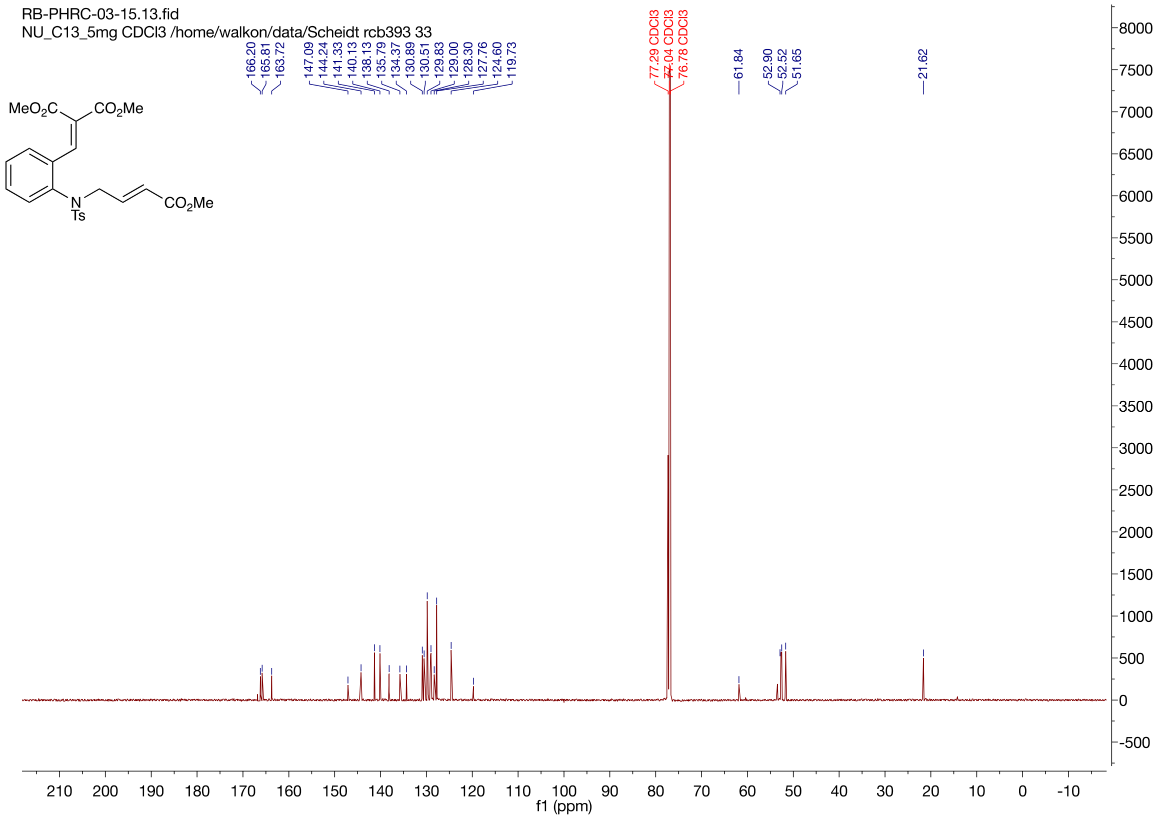
RB-PHRC-03-15.13.fid
NU_C13_5mg CDCl3 /home/walkon/data/Scheidt rcb393 33
166.20 165.81 163.72 147.09 144.24 141.33 140.13 138.13 135.79 134.37 130.89 130.51 129.83 129.00 128.30 127.76 124.60 119.73
77.29 CDCl3 77.04 CDCl3 76.78 CDCl3
61.84 52.90 52.52 51.65 21.62
MeO2C CO2Me
N Ts CO2Me
f1 (ppm)

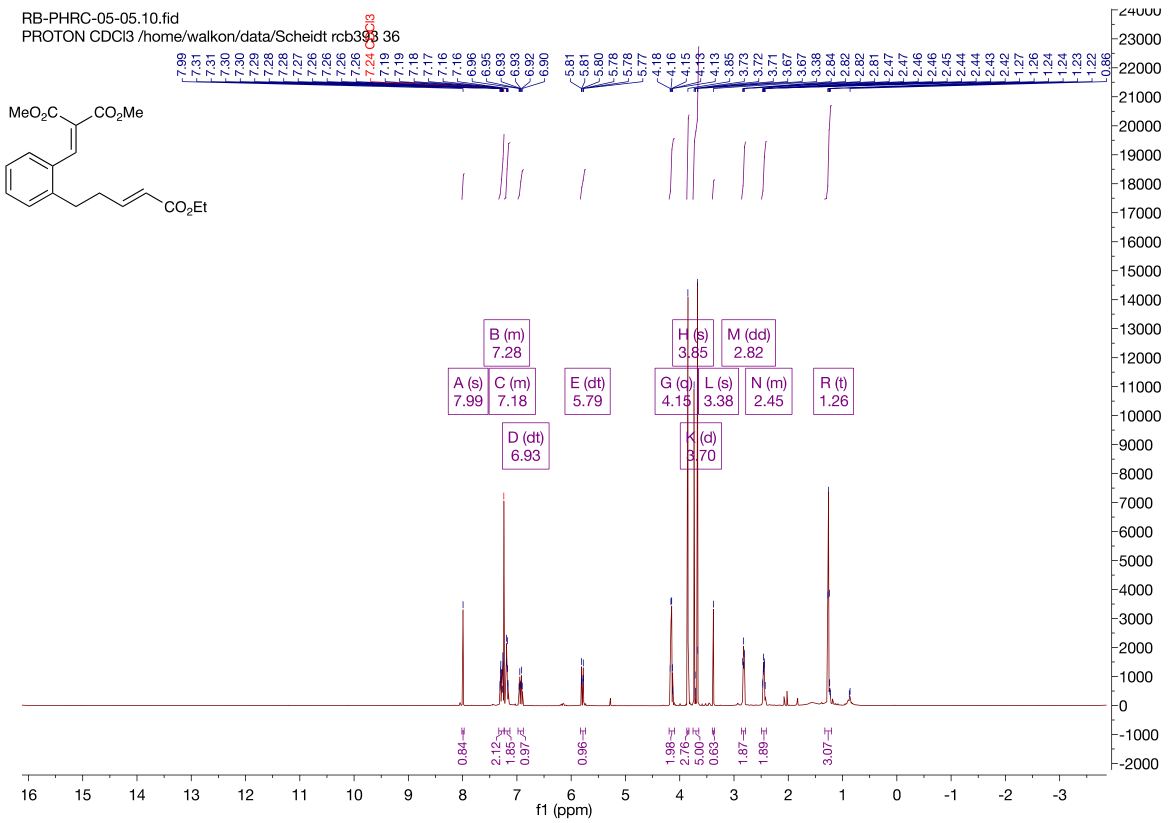
RB-PHRC-05-05.10.fid
PROTON CDCl3 /home/walkon/data/Scheidt rcb393 36
MeO2C
CO2Me
CO2Et
A (s) 7.99
B (m) 7.28
C (m) 7.18
D (dt) 6.93
E (dt) 5.79
G (q) 4.15
H (s) 3.85
K (d) 3.70
L (s) 3.38
M (dd) 2.82
N (m) 2.45
R (t) 1.26
f1 (ppm)

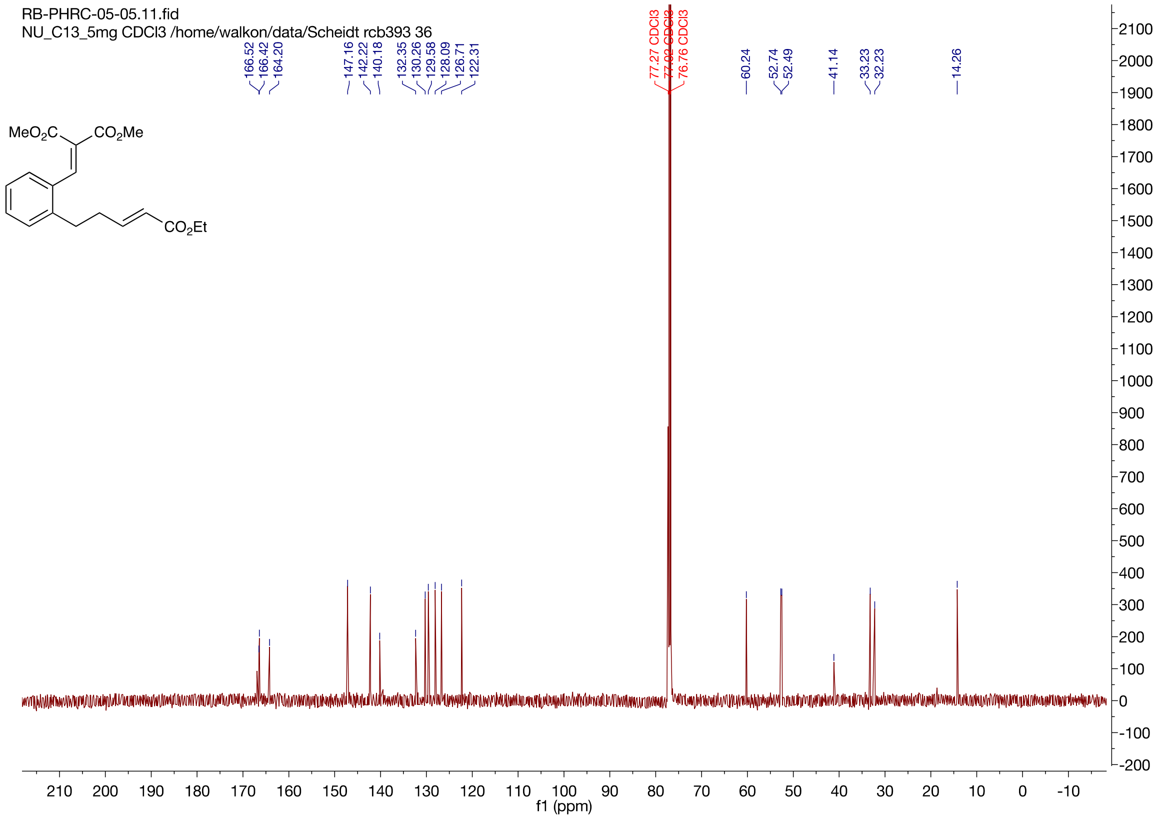
RB-PHRC-05-05.11.fid
NU_C13_5mg CDCl3 /home/walkon/data/Scheidt rcb393 36
MeO2C
CO2Me
CO2Et
166.52
166.42
164.20
147.16
142.22
140.18
132.35
130.26
129.58
128.09
126.71
122.31
77.27 CDCl3
77.02 CDCl3
76.76 CDCl3
60.24
52.74
52.49
41.14
33.23
32.23
14.26
f1 (ppm)

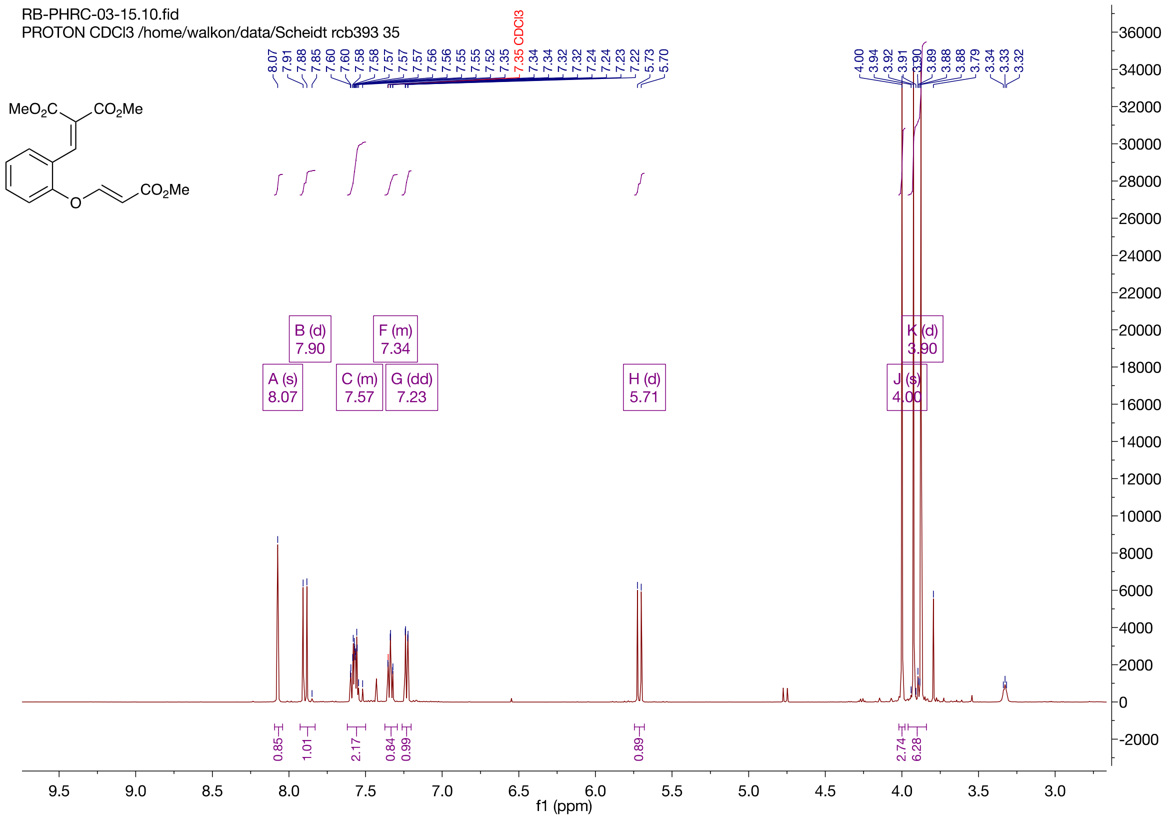
RB-PHRC-03-15.10.fid
PROTON CDCl3 /home/walkon/data/Scheidt rcb393 35
MeO2C CO2Me
O CO2Me
A (s) 8.07
B (d) 7.90
C (m) 7.57
F (m) 7.34
G (dd) 7.23
H (d) 5.71
J (s) 4.00
K (d) 3.90
f1 (ppm)

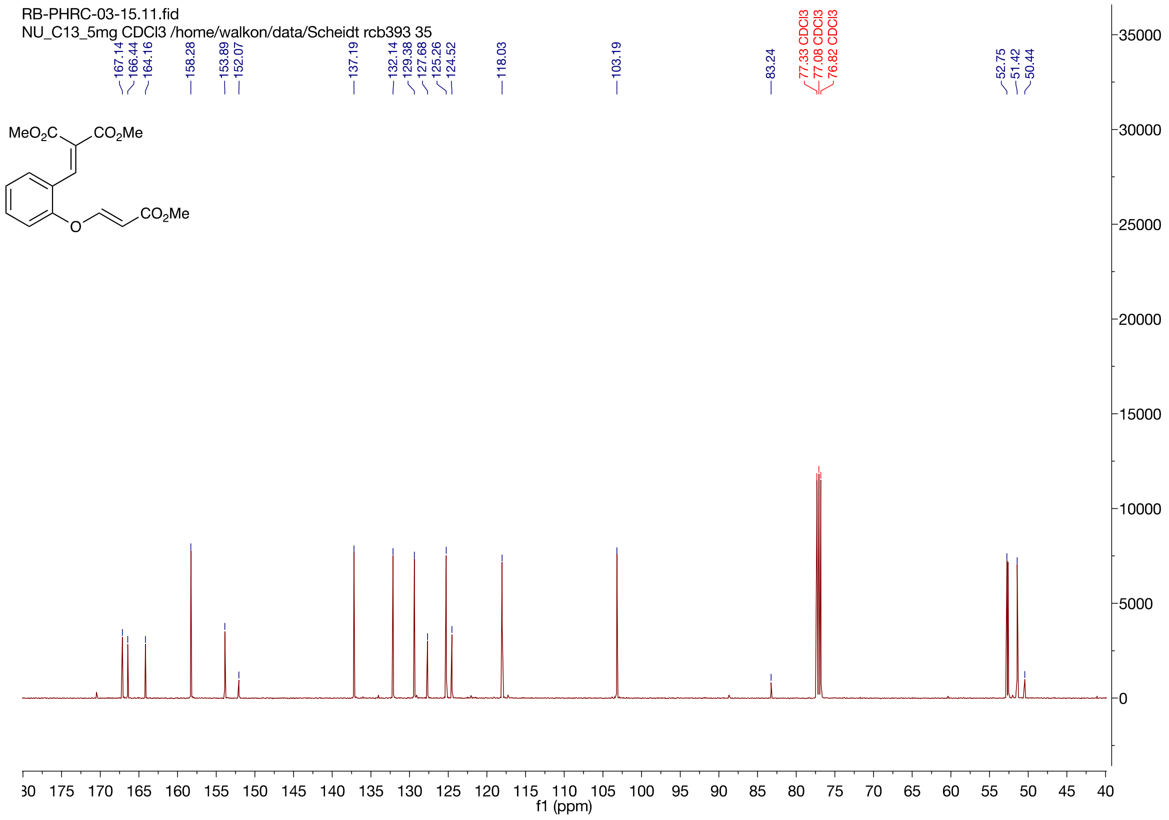
RB-PHRC-03-15.11.fid
NU_C13_5mg CDCl3 /home/walkon/data/Scheidt rcb393 35
167.14
166.44
164.16
158.28
153.89
152.07
137.19
132.14
129.38
127.68
125.26
124.52
118.03
103.19
83.24
77.33 CDCl3
77.08 CDCl3
76.82 CDCl3
52.75
51.42
50.44
MeO2C CO2Me
O CO2Me
f1 (ppm)

NMR Spectra for Cyclization Products

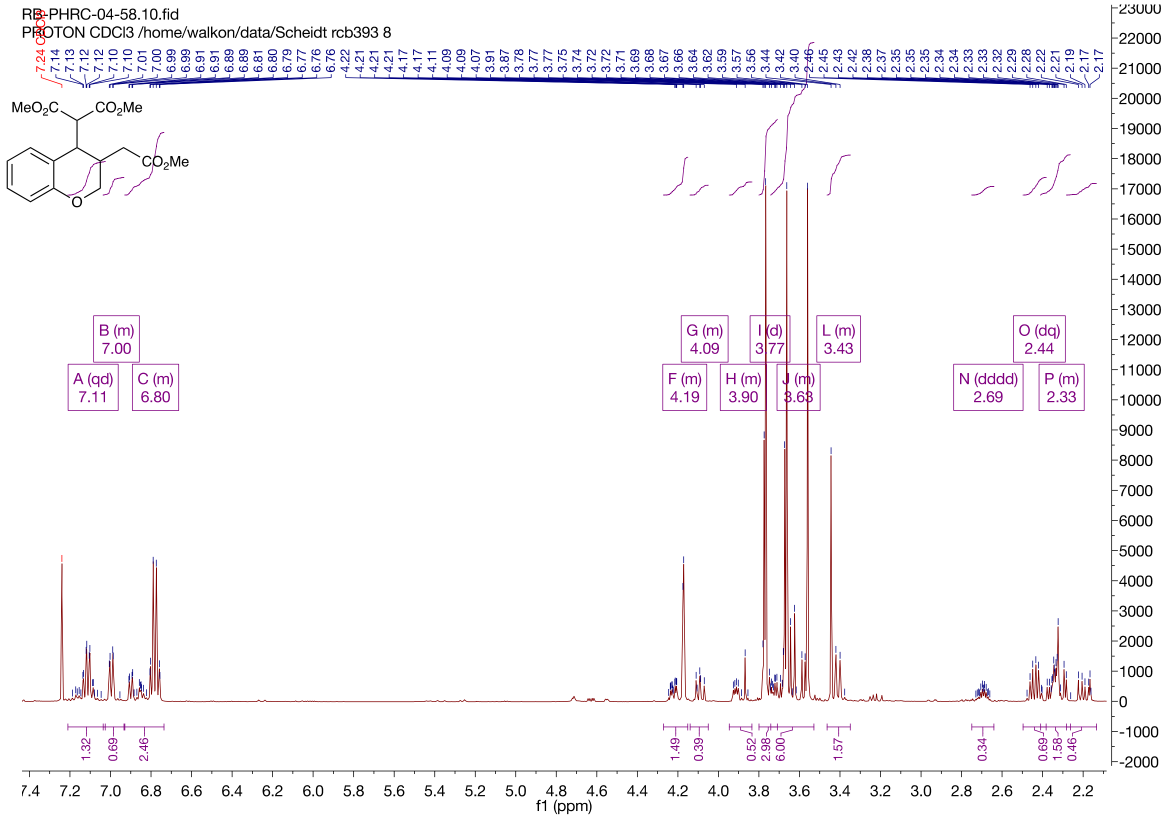

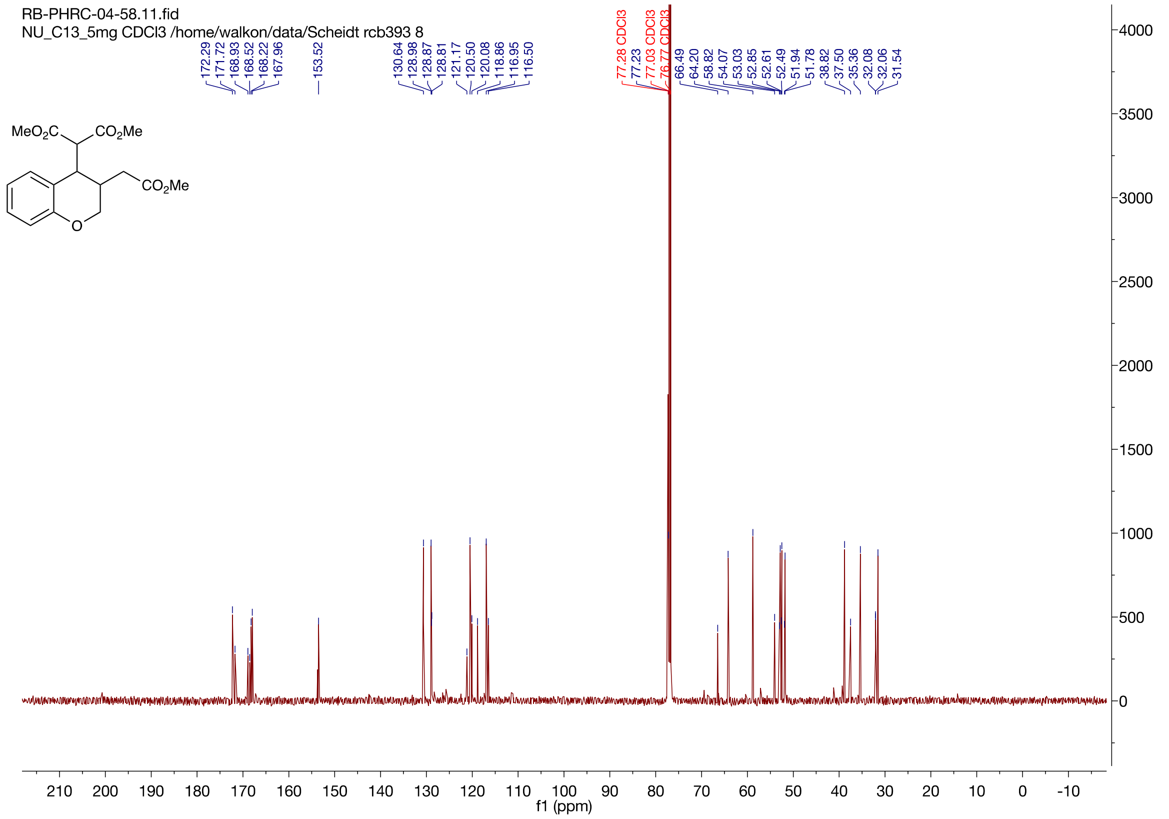

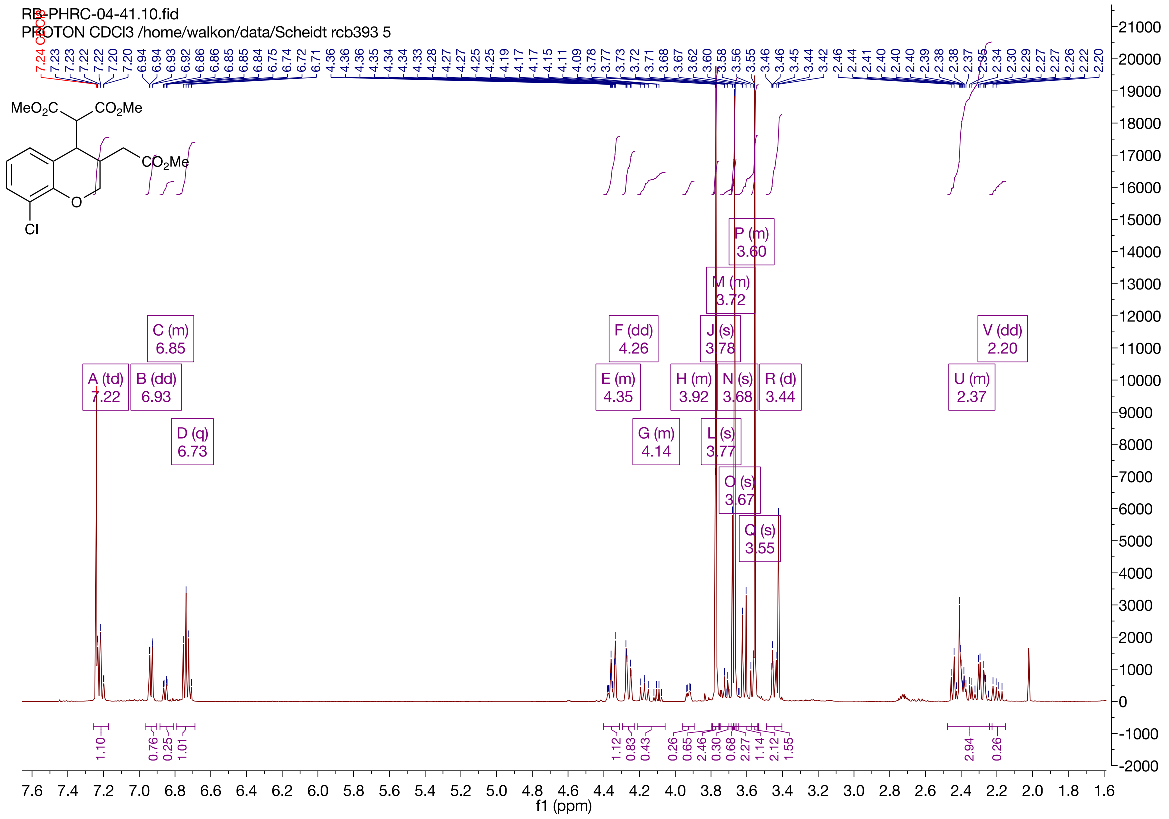
RB-PHRC-04-41.10.fid
PROTON CDCl3 /home/walkon/data/Scheidt rcb393 5
MeO2C
CO2Me
CO2Me
O
Cl
f1 (ppm)

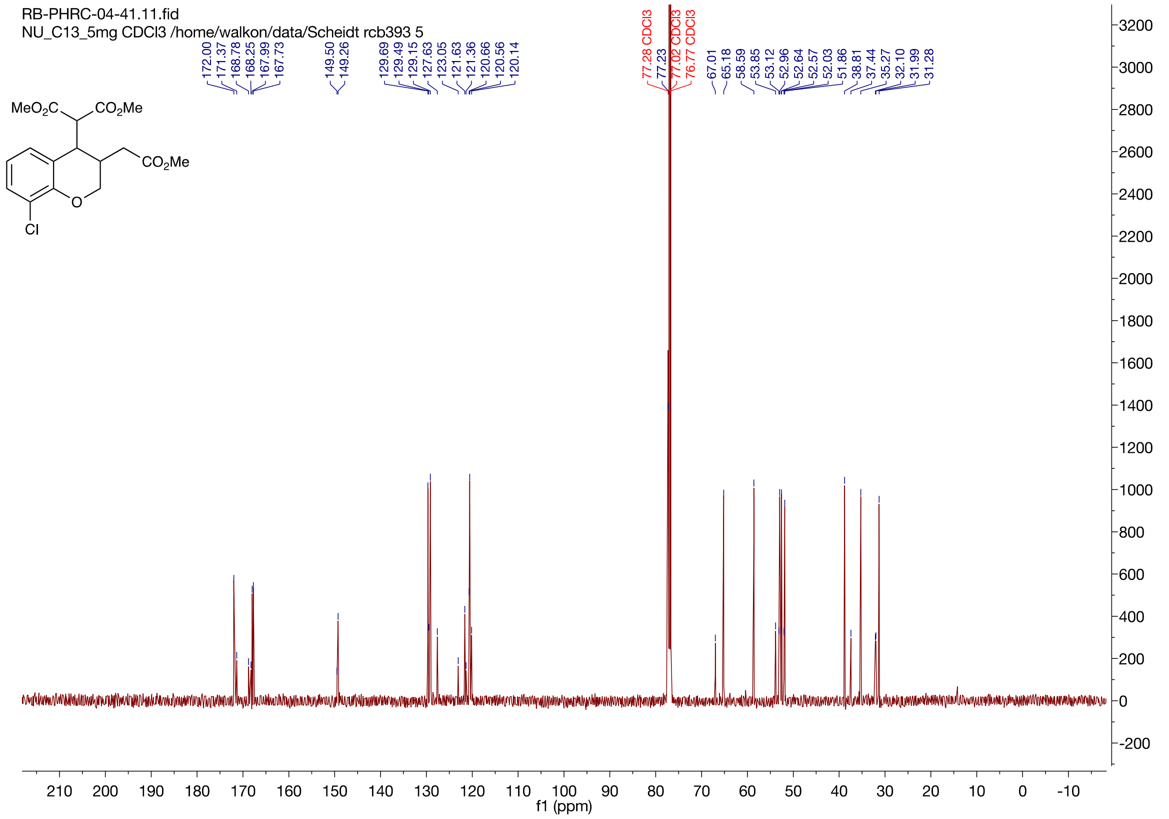
RB-PHRC-04-41.11.fid
NU_C13_5mg CDCl3 /home/walkon/data/Scheidt rcb393 5
MeO2C
CO2Me
CO2Me
O
Cl
f1 (ppm)

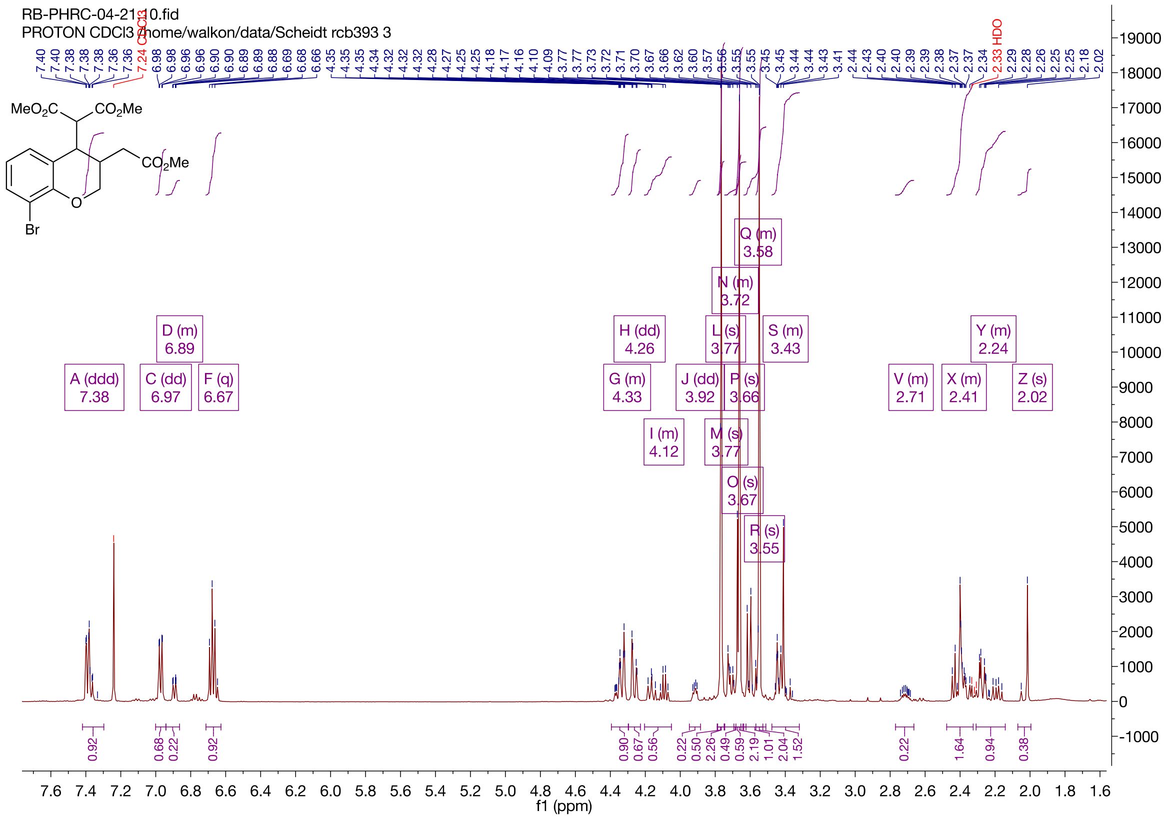
RB-PHRC-04-21.10.fid
PROTON CDCl3 /home/walkon/data/Scheidt rcb393 3
f1 (ppm)

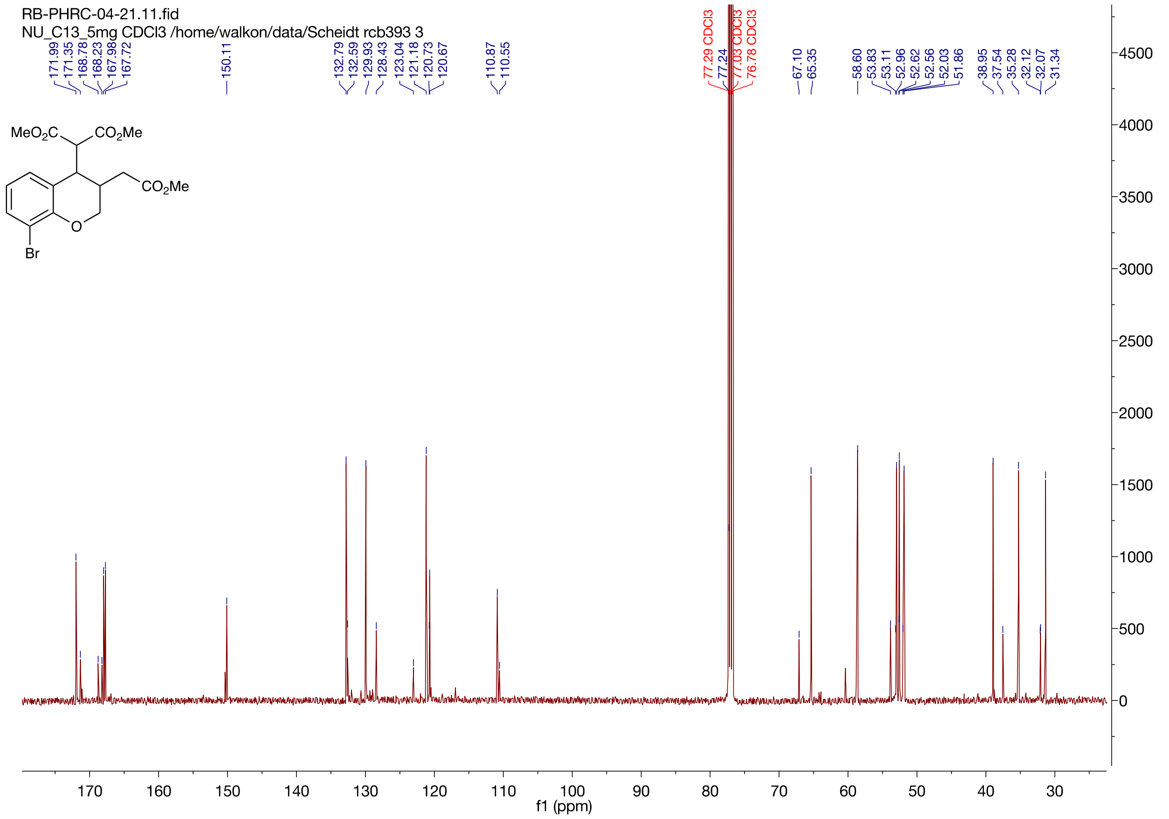
RB-PHRC-04-21.11.fid
NU_C13_5mg CDCl3 /home/walkon/data/Scheidt rcb393 3
f1 (ppm)

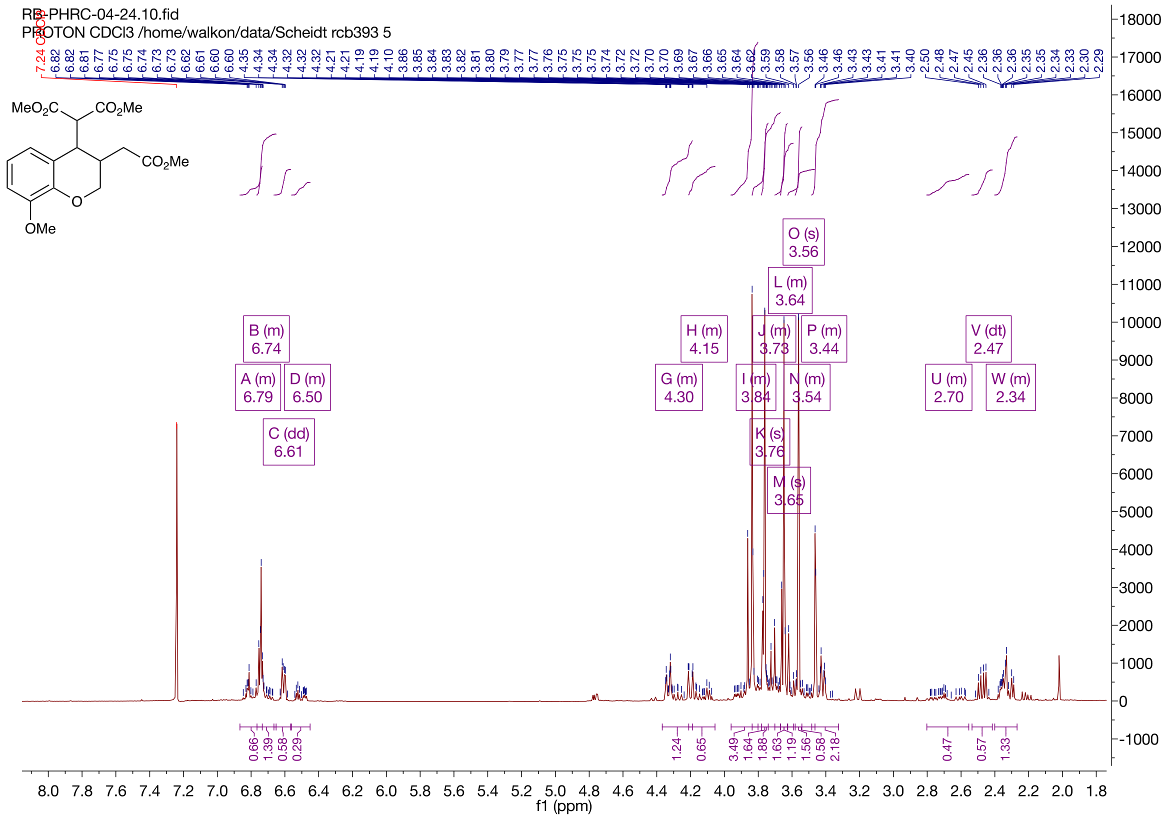
RB-PHRC-04-24.10.fid
PROTON CDCl3 /home/walkon/data/Scheidt rcb393 5
MeO2C
CO2Me
CO2Me
OMe
f1 (ppm)

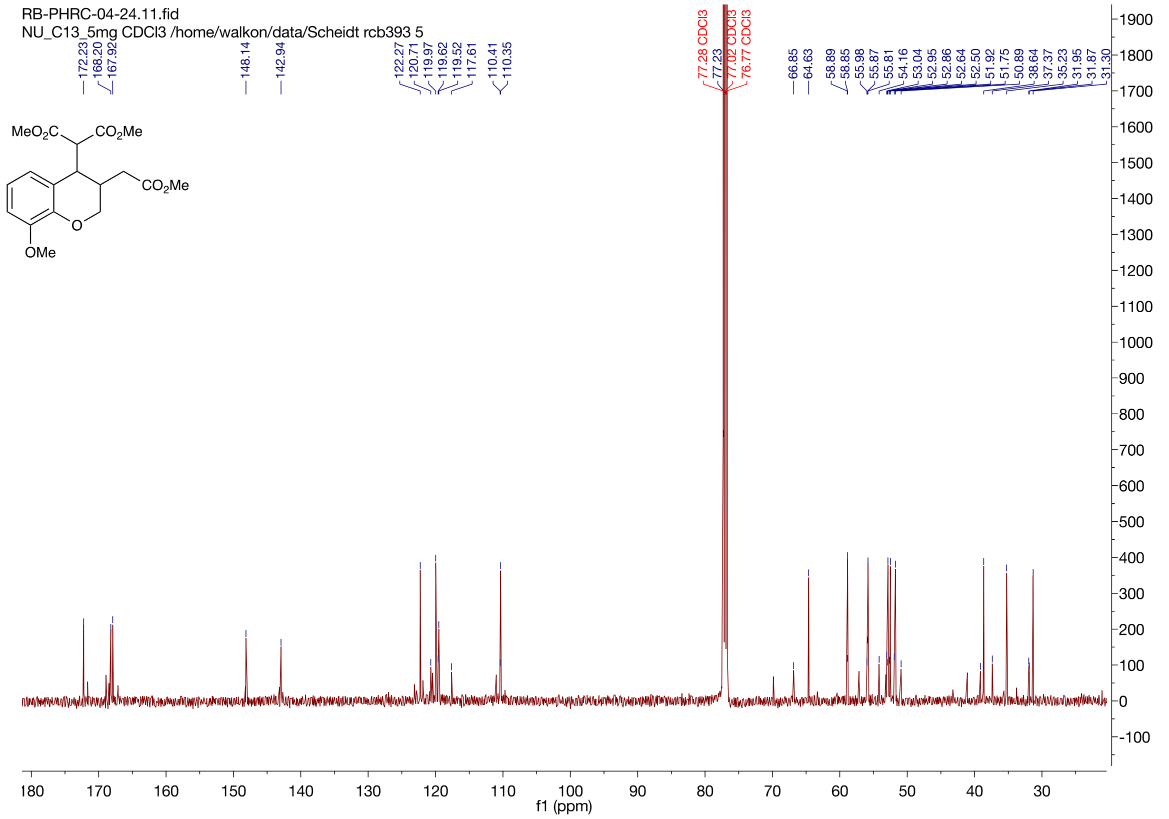
RB-PHRC-04-24.11.fid
NU_C13_5mg CDCl3 /home/walkon/data/Scheidt rcb393 5
MeO2C
CO2Me
CO2Me
OMe
f1 (ppm)

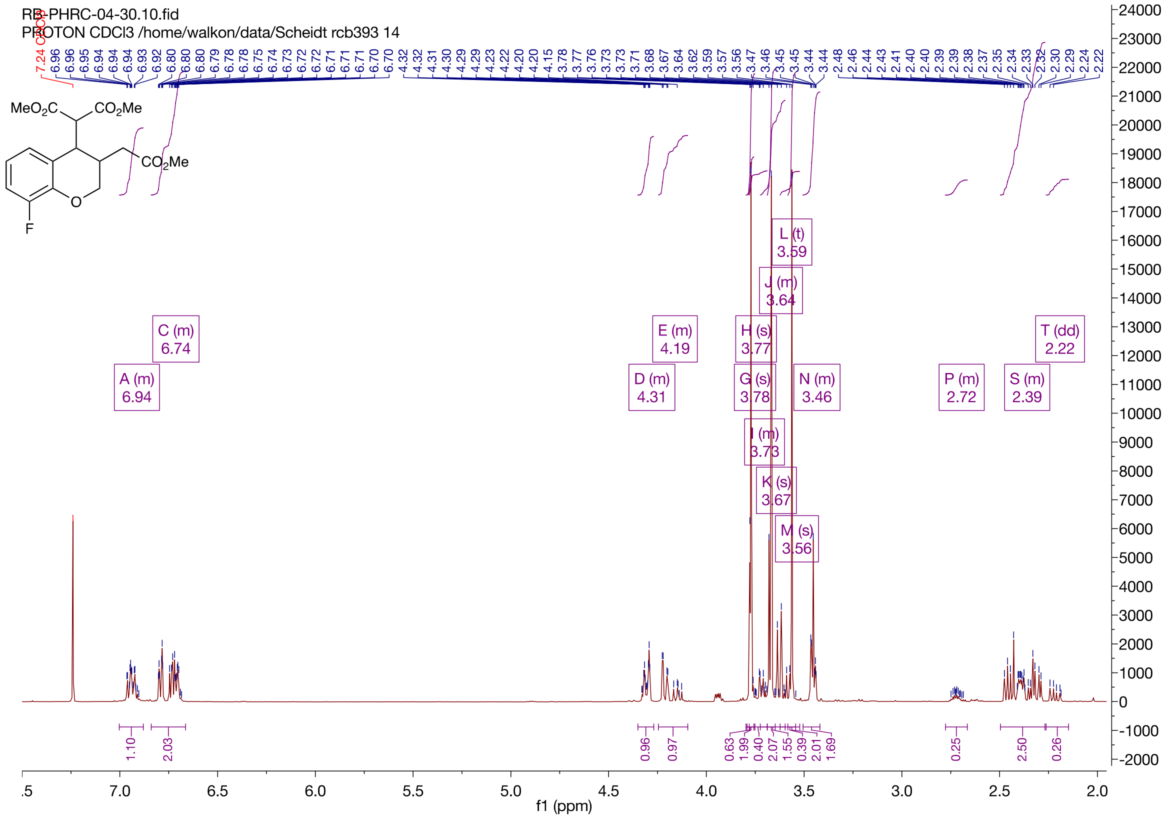
RB-PHRC-04-30.10.fid
PROTON CDCl3 /home/walkon/data/Scheidt rcb393 14
MeO2C
CO2Me
CO2Me
O
F
f1 (ppm)

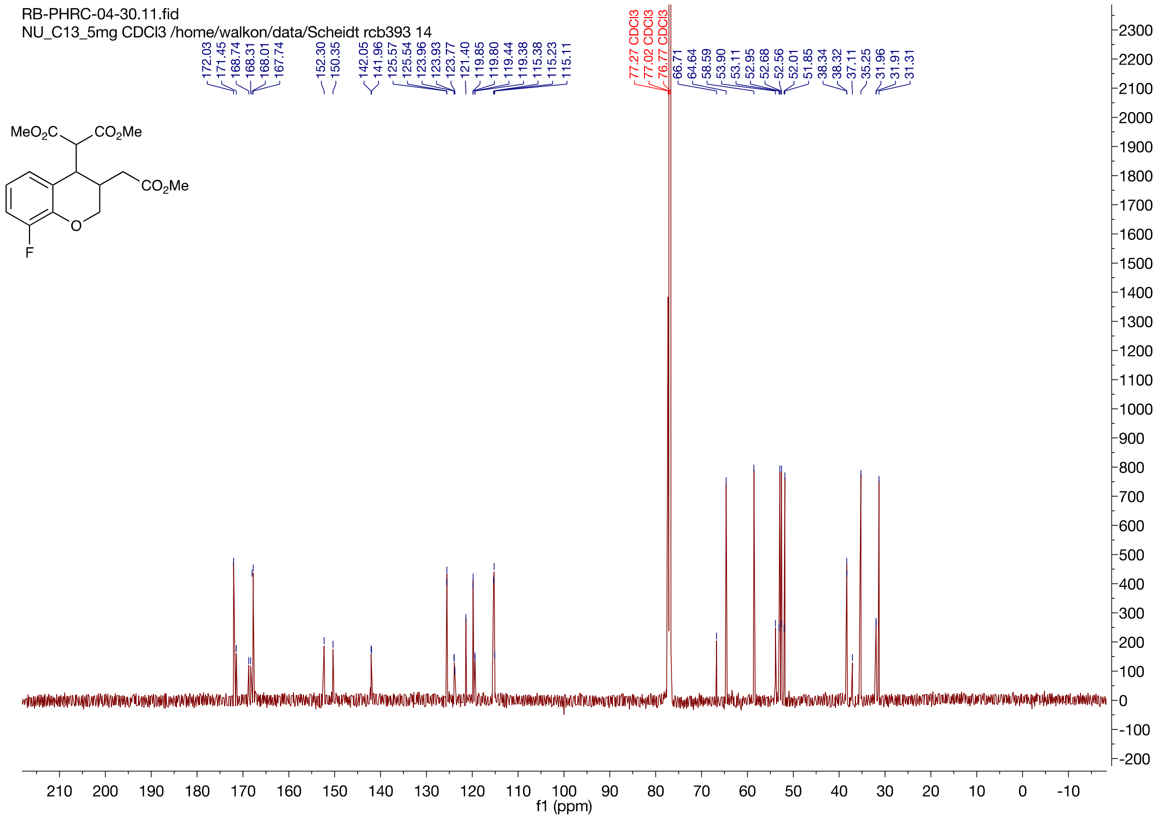
RB-PHRC-04-30.11.fid
NU_C13_5mg CDCl3 /home/walkon/data/Scheidt rcb393 14
MeO2C
CO2Me
CO2Me
O
F
f1 (ppm)

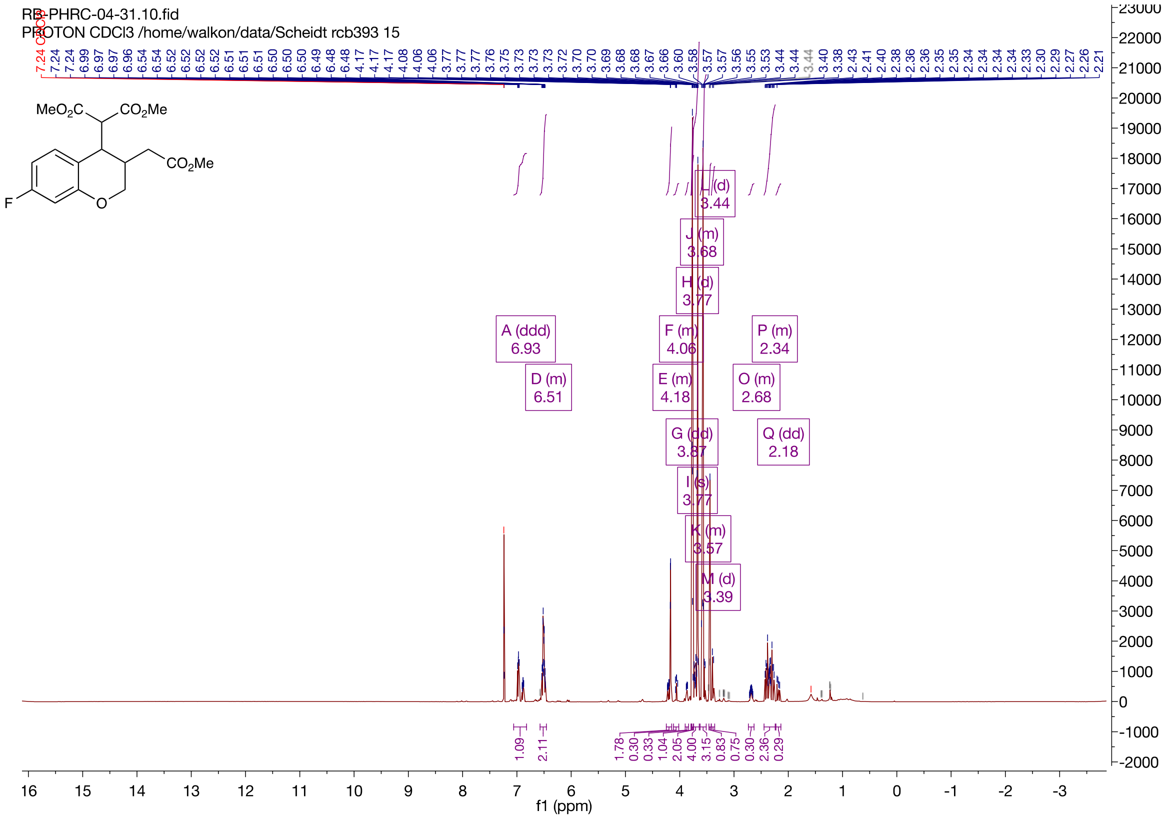
RB-PHRC-04-31.10.fid
PROTON CDCl3 /home/walkon/data/Scheidt rcb393 15
f1 (ppm)

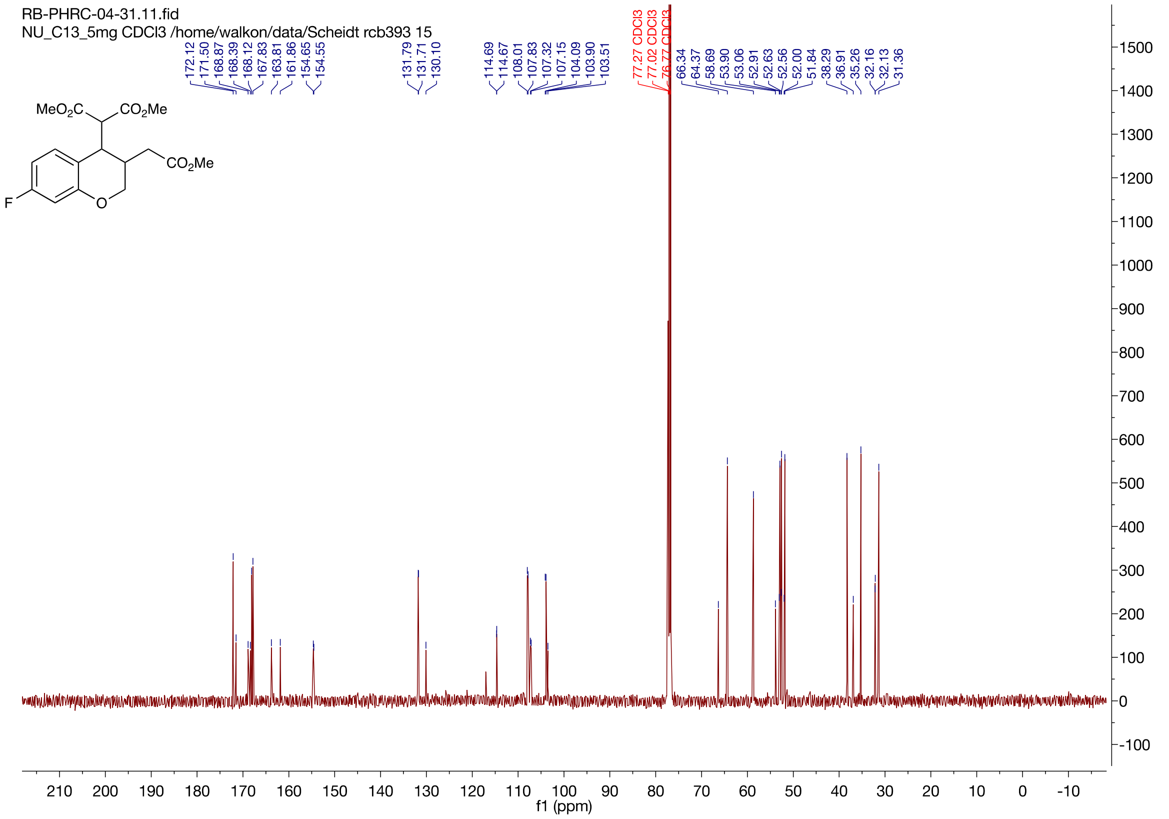
RB-PHRC-04-31.11.fid
NU_C13_5mg CDCl3 /home/walkon/data/Scheidt rcb393 15
f1 (ppm)

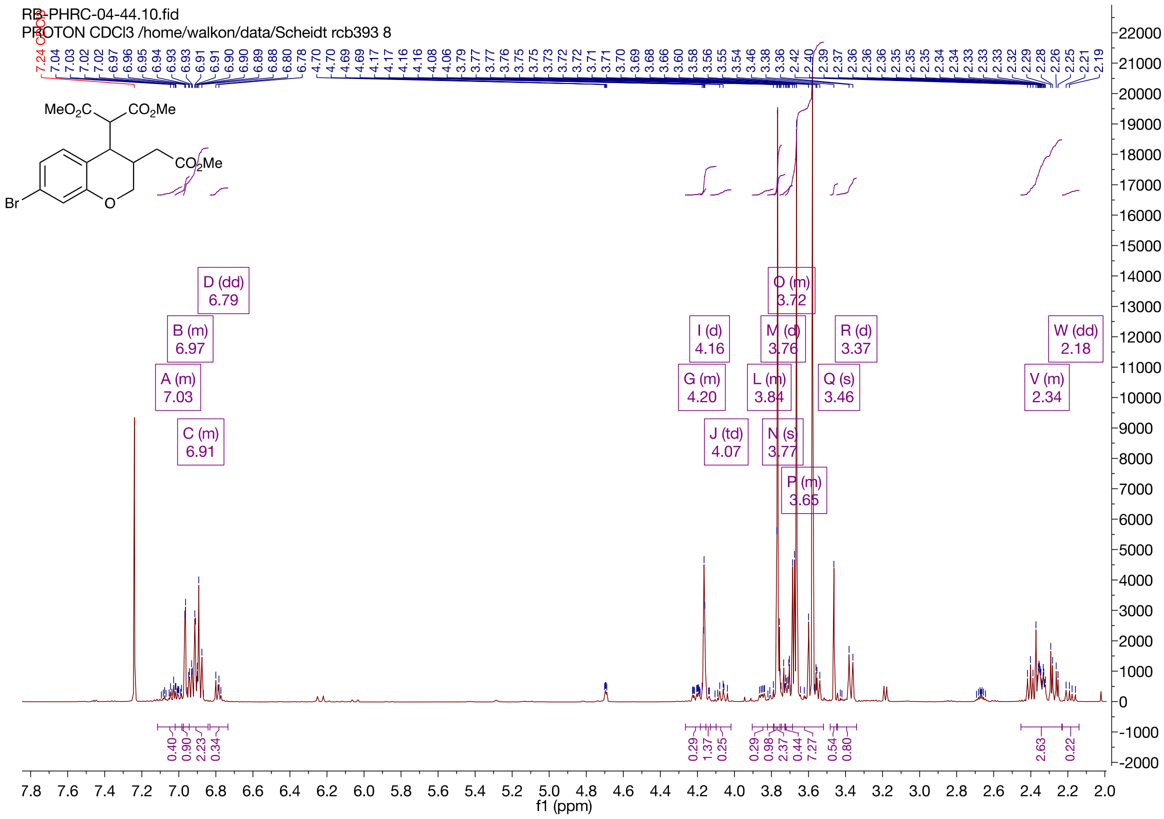
RB-PHRC-04-44.10.fid
PROTON CDCl3 /home/walkon/data/Scheidt rcb393 8
MeO2C
CO2Me
CO2Me
Br
O
f1 (ppm)

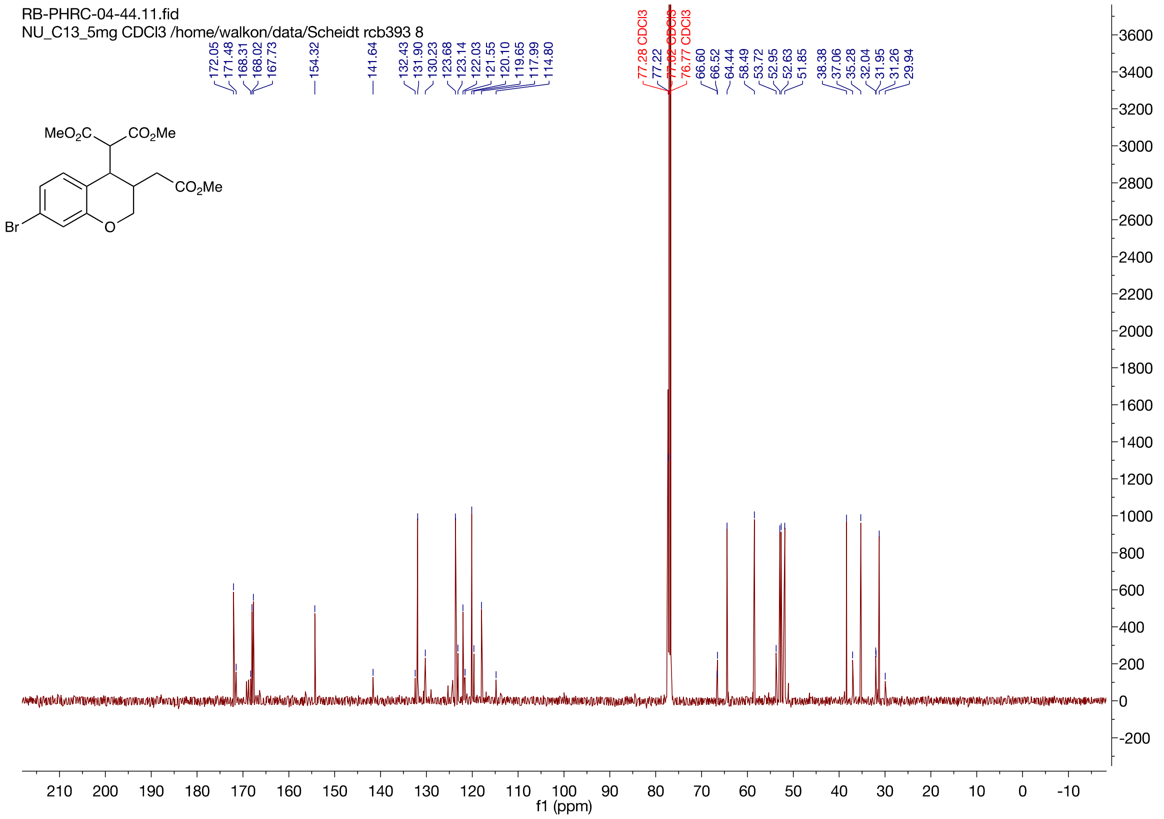
RB-PHRC-04-44.11.fid
NU_C13_5mg CDCl3 /home/walkon/data/Scheidt rcb393 8
172.05
171.48
168.31
168.02
167.73
154.32
141.64
132.43
131.90
130.23
123.68
123.14
122.03
121.55
120.10
119.65
117.99
114.80
77.28 CDCl3
77.22
76.77 CDCl3
66.60
66.52
64.44
58.49
53.72
52.95
52.63
51.85
38.38
37.06
35.28
32.04
31.95
31.26
29.94
MeO2C
CO2Me
CO2Me
Br
O
f1 (ppm)

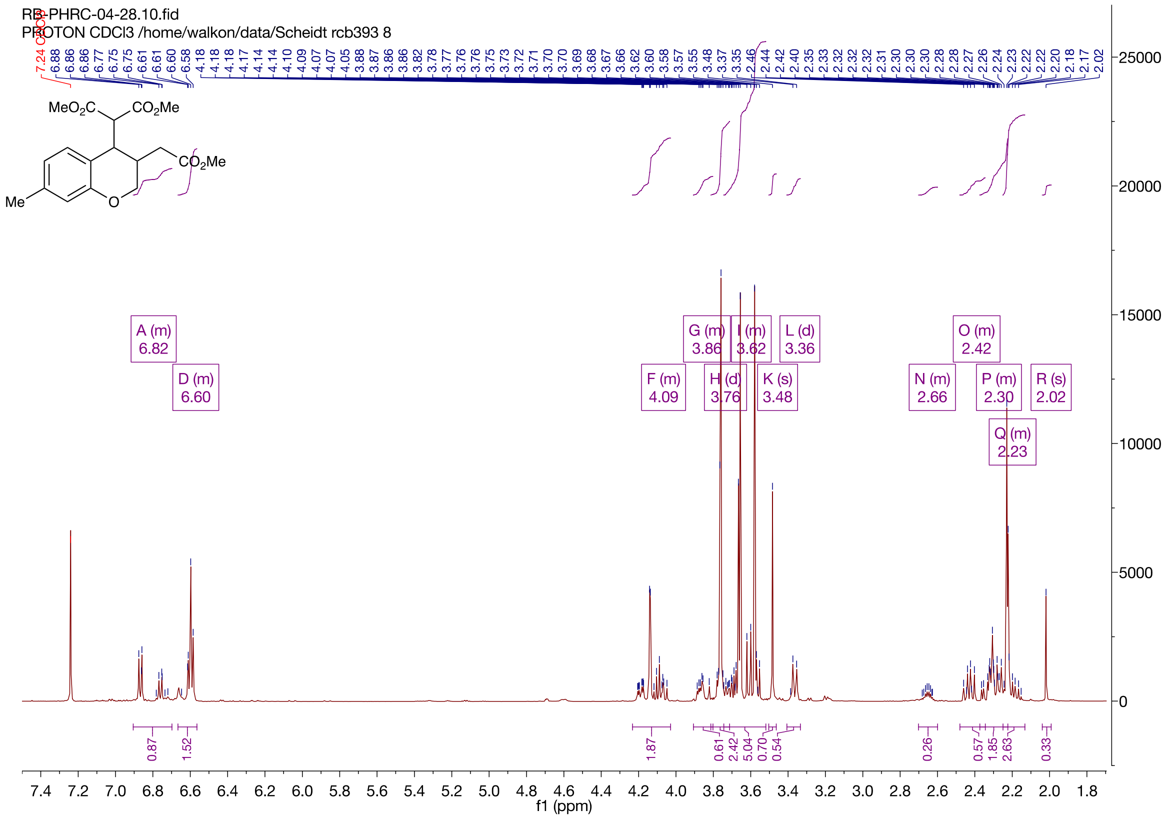
RB-PHRC-04-28.10.fid
PROTON CDCl3 /home/walkon/data/Scheidt rcb393 8
MeO2C
CO2Me
CO2Me
Me
O
A (m) 6.82
D (m) 6.60
F (m) 4.09
G (m) 3.86
H (d) 3.76
I (m) 3.62
K (s) 3.48
L (d) 3.36
N (m) 2.66
O (m) 2.42
P (m) 2.30
Q (m) 2.23
R (s) 2.02
f1 (ppm)

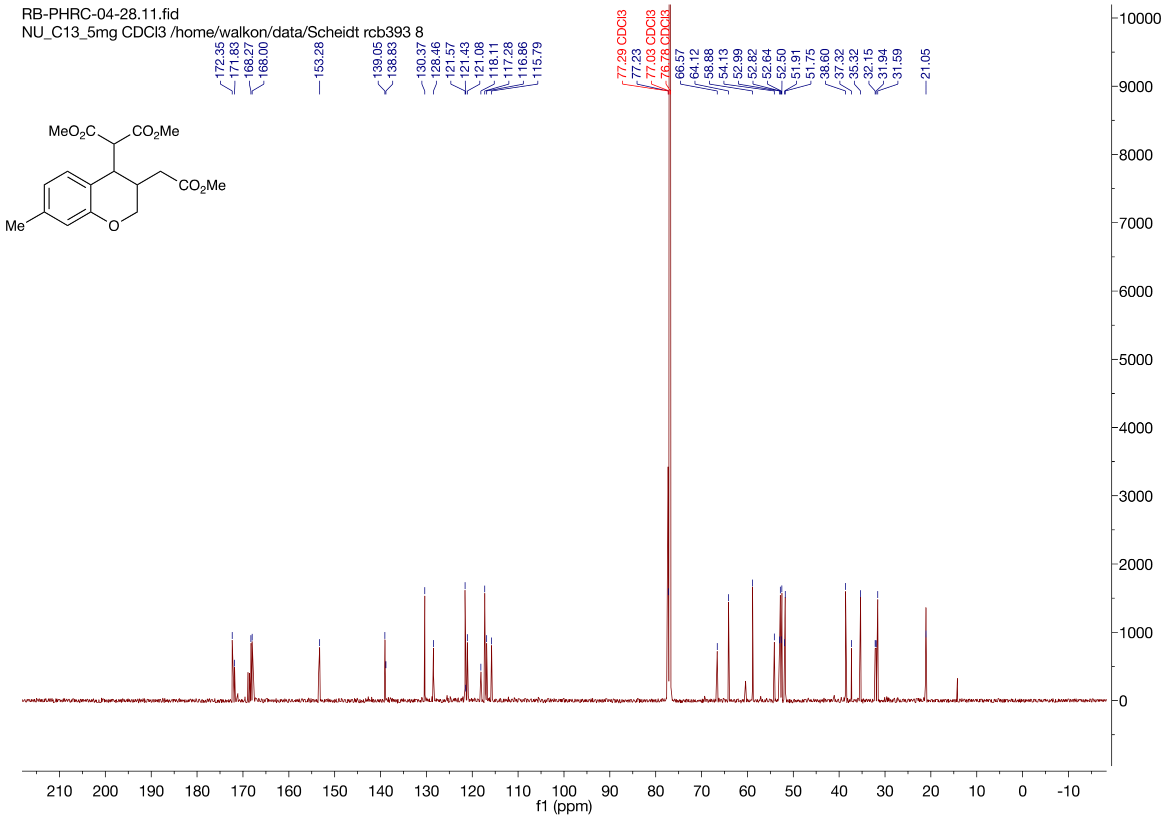
RB-PHRC-04-28.11.fid
NU_C13_5mg CDCl3 /home/walkon/data/Scheidt rcb393 8
172.35
171.83
168.27
168.00
153.28
139.05
138.83
130.37
128.46
121.57
121.43
121.08
118.11
117.28
116.86
115.79
77.29 CDCl3
77.23
77.03 CDCl3
76.78 CDCl3
66.57
64.12
58.88
54.13
52.99
52.82
52.64
52.50
51.91
51.75
38.60
37.32
35.32
32.15
31.94
31.59
21.05
MeO2C
CO2Me
CO2Me
Me
O
f1 (ppm)

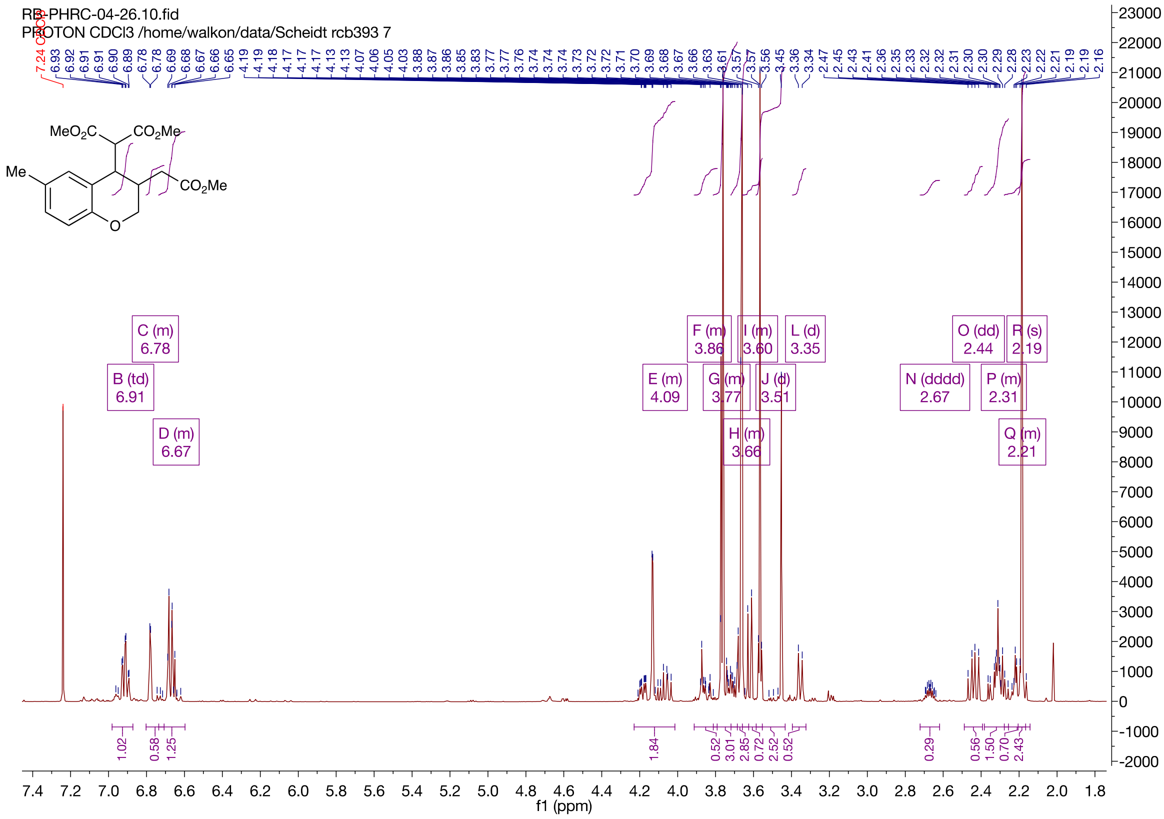

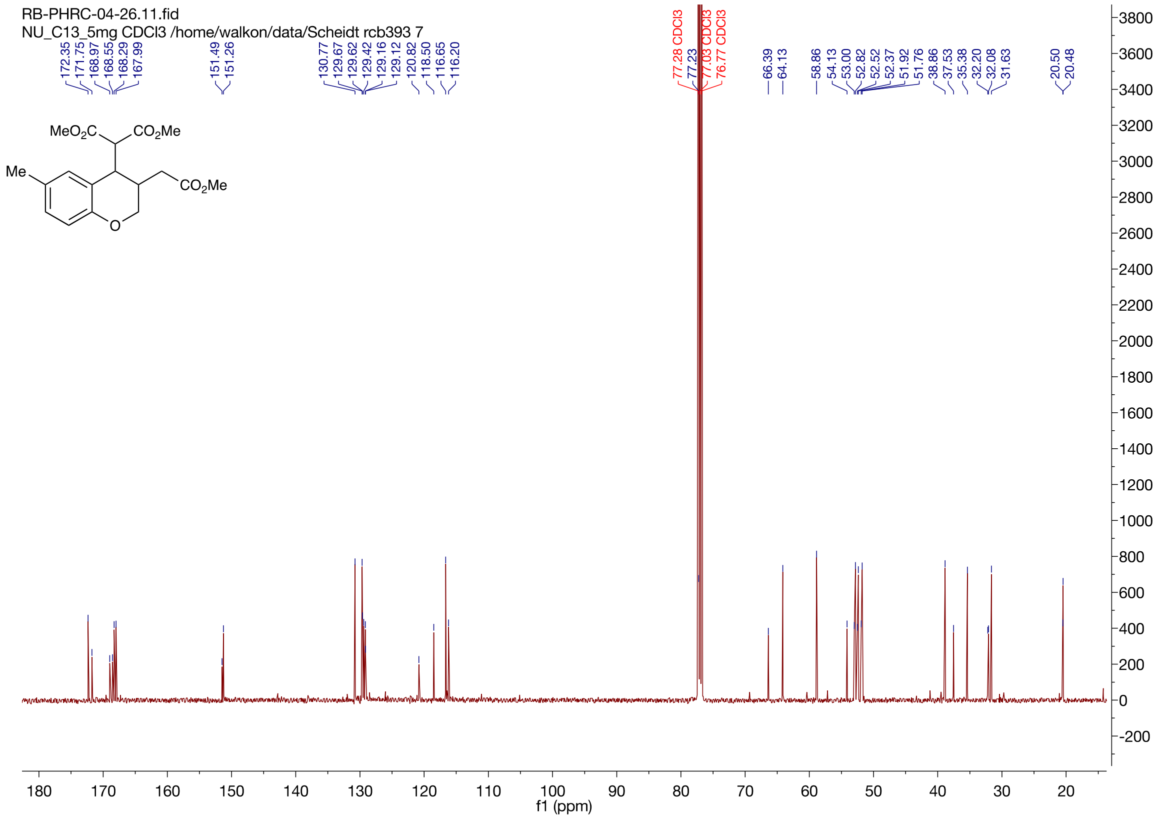

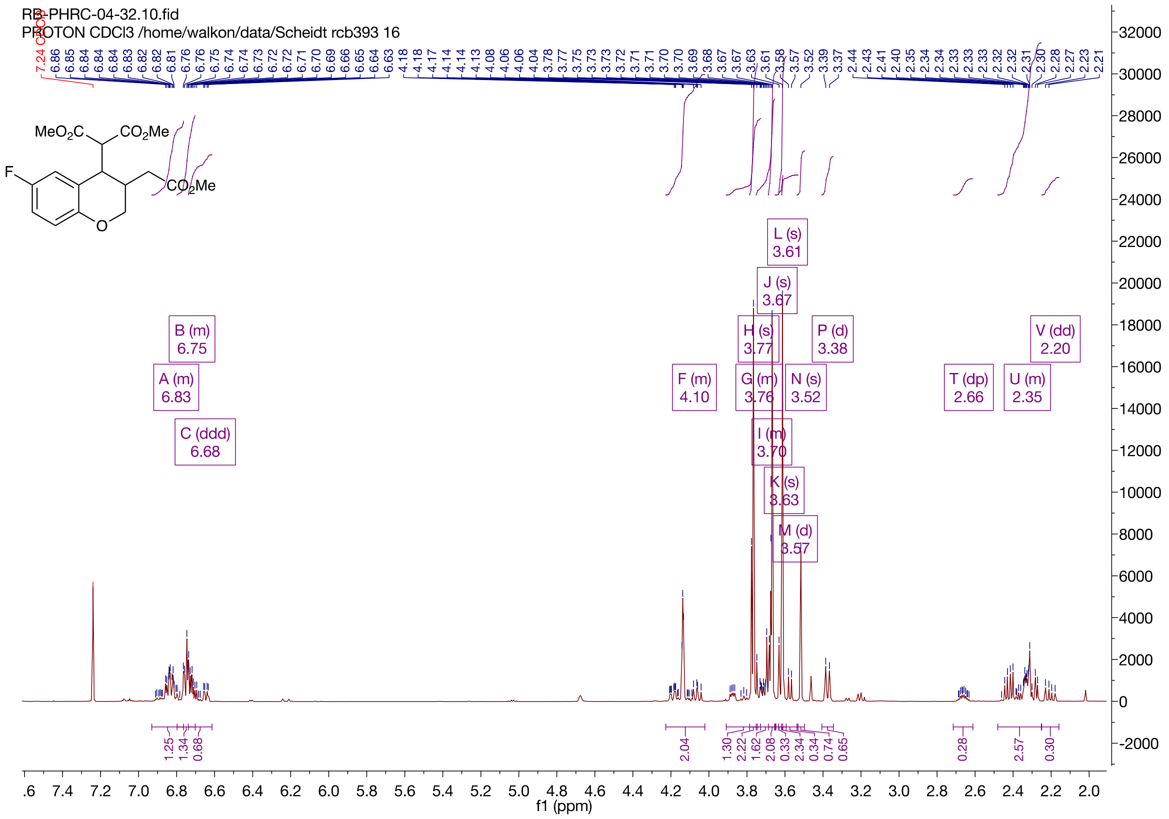

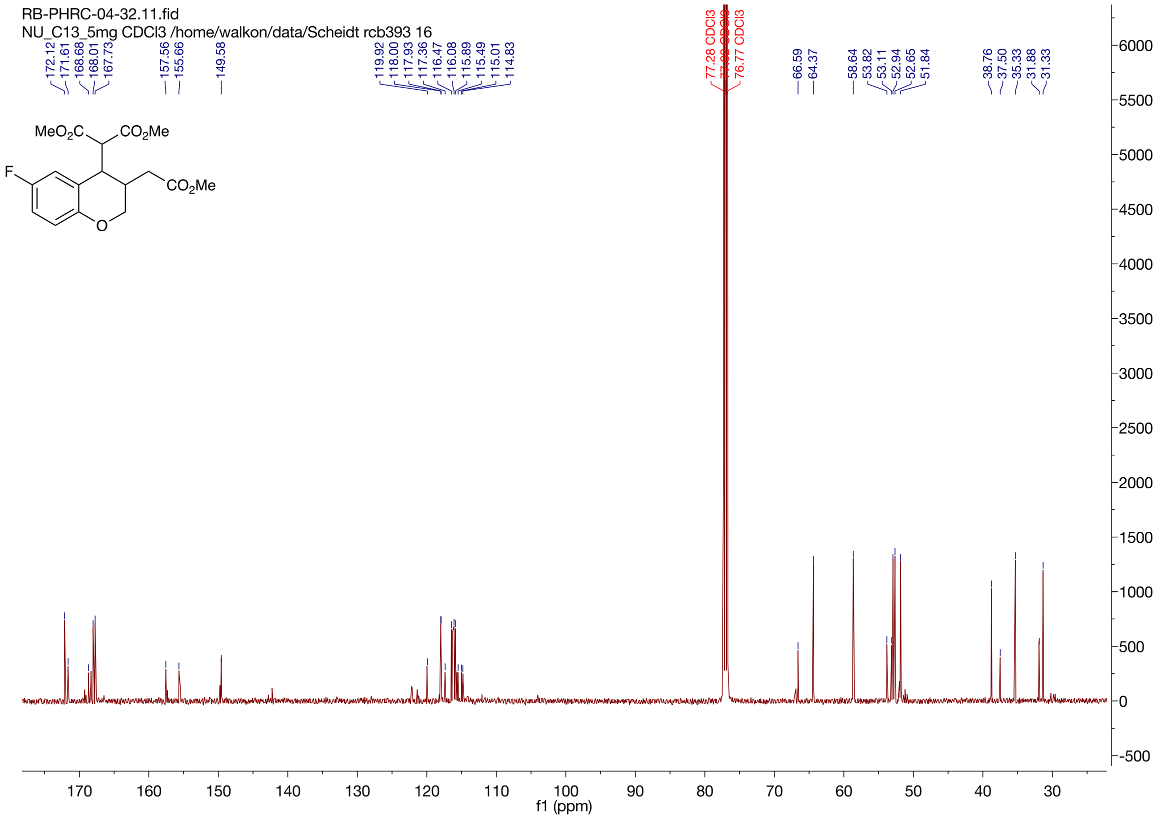

RB-PHRC-04-25.10.fid
PROTON CDCl3 /home/walkon/data/Scheidt rcb393 6

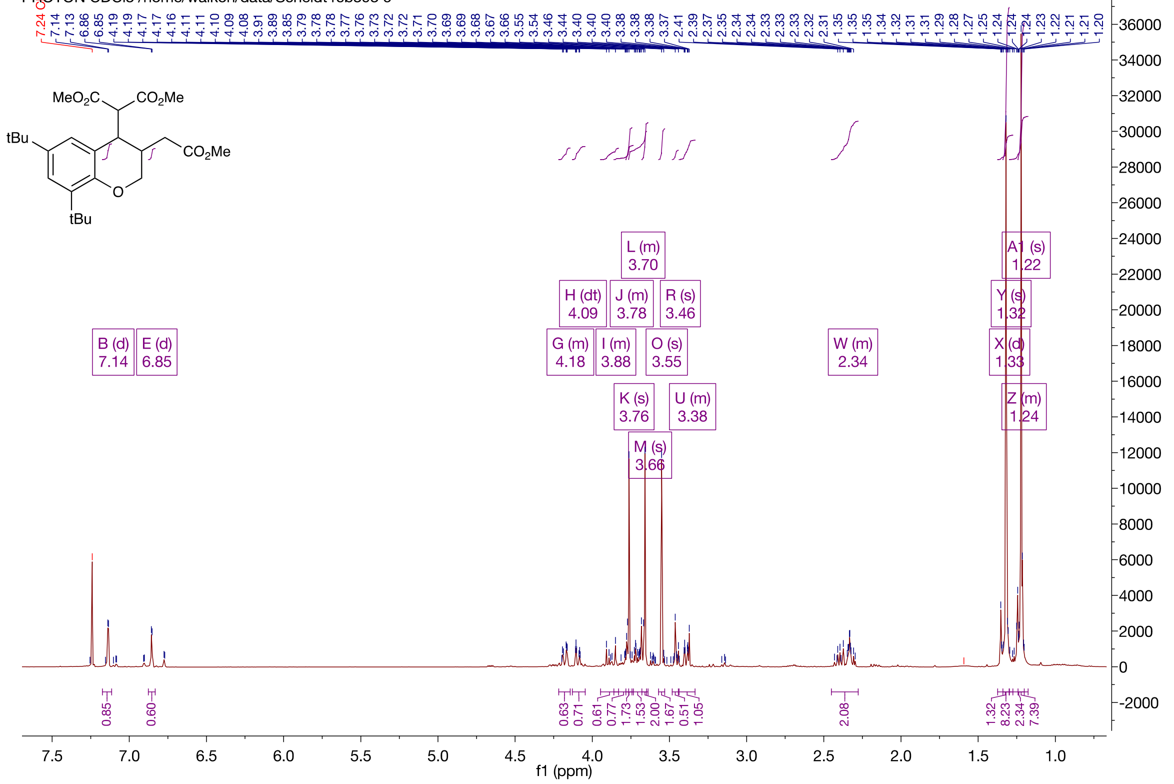

RB-PHRC-04-25.11.fid
NU_C13_5mg CDCl3 /home/walkon/data/Scheidt rcb393 6

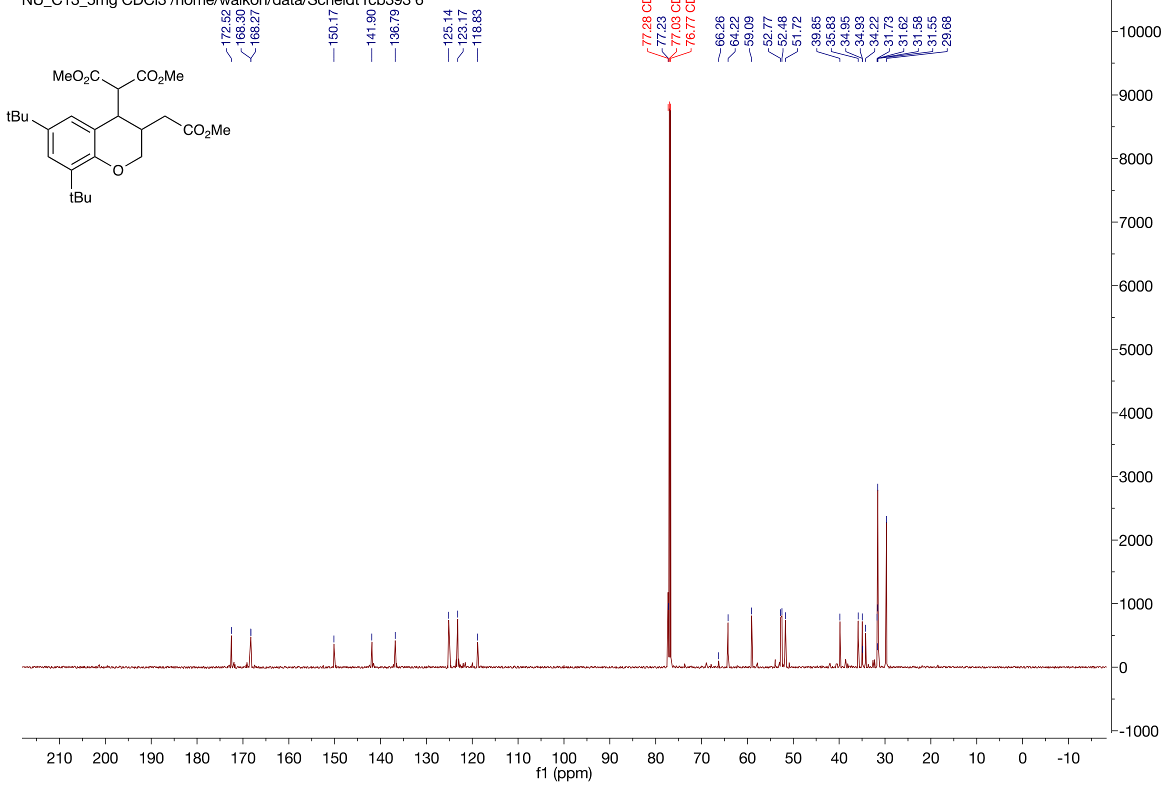

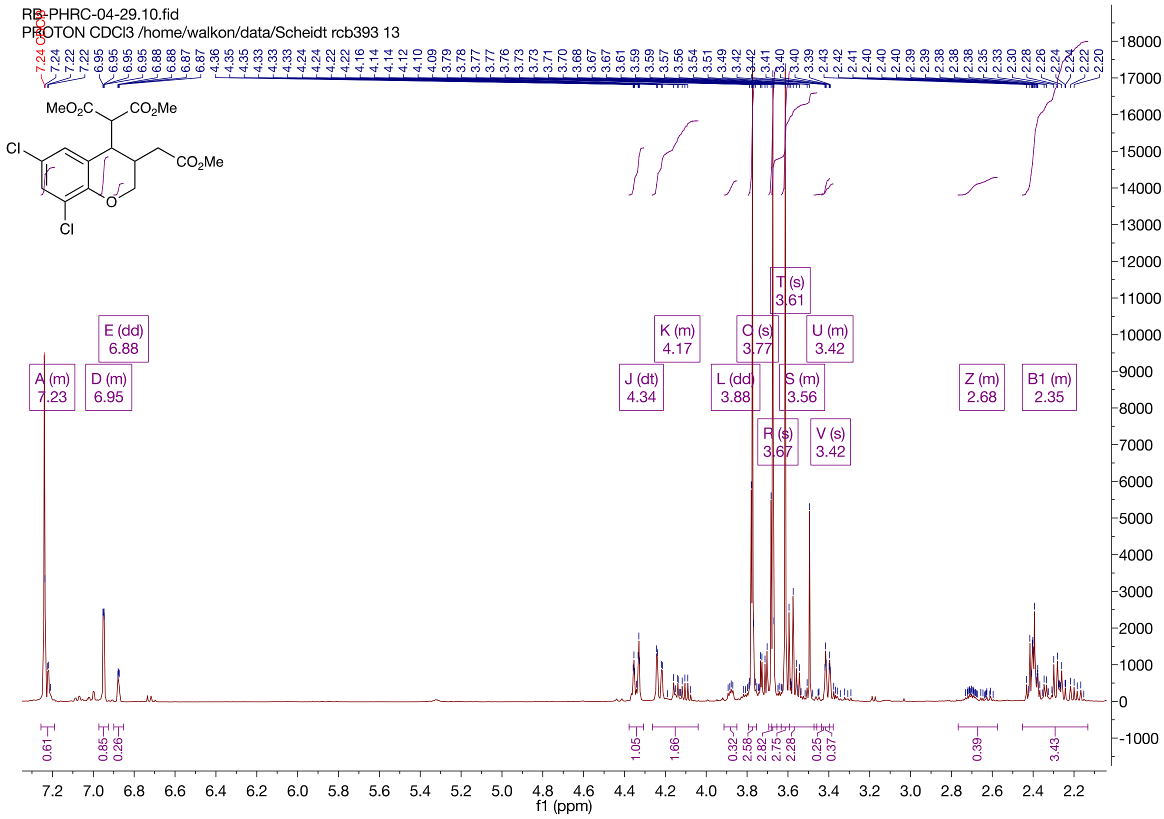
RB-PHRC-04-29.10.fid
PROTON CDCl3 /home/walkon/data/Scheidt rcb393 13
MeO2C
CO2Me
Cl
CO2Me
O
Cl
A (m) 7.23
D (m) 6.95
E (dd) 6.88
J (dt) 4.34
K (m) 4.17
L (dd) 3.88
Q (s) 3.77
R (s) 3.67
T (s) 3.61
S (m) 3.56
U (m) 3.42
V (s) 3.42
Z (m) 2.68
B1 (m) 2.35
f1 (ppm)

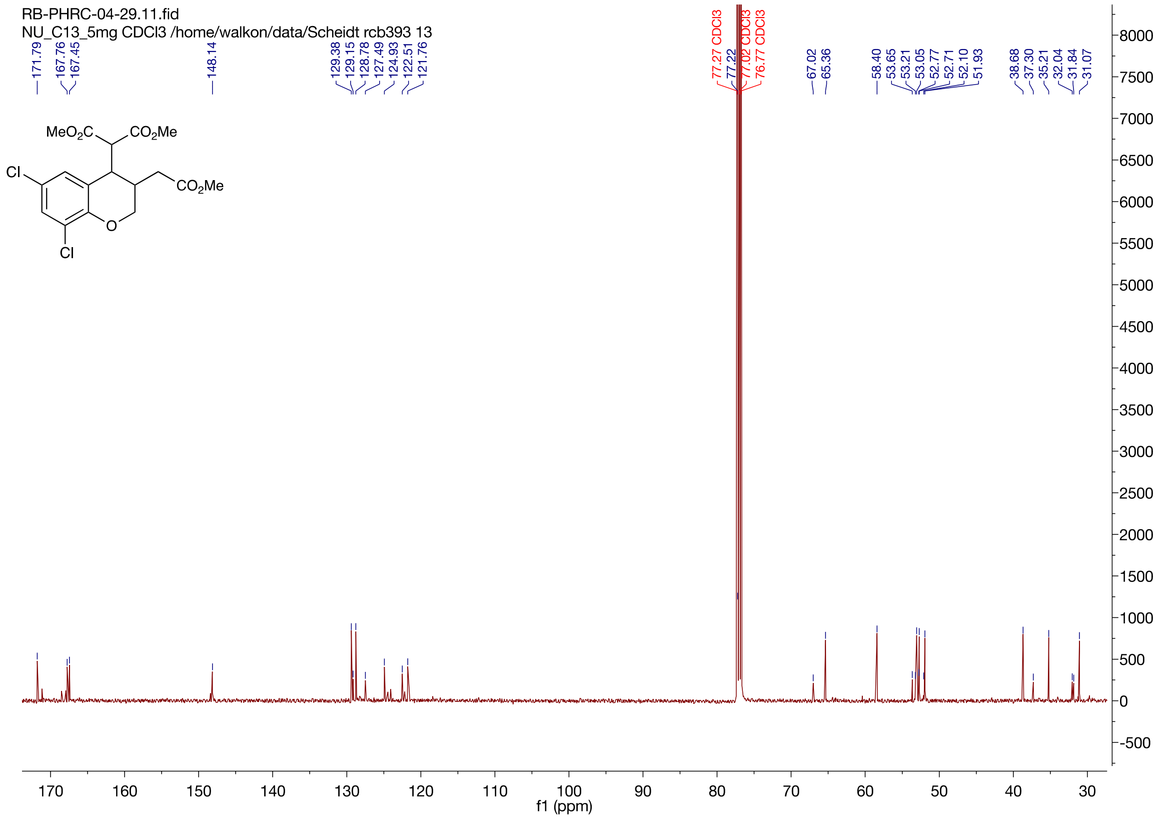
RB-PHRC-04-29.11.fid
NU_C13_5mg CDCl3 /home/walkon/data/Scheidt rcb393 13
171.79
167.76
167.45
148.14
129.38
129.15
128.78
127.49
124.93
122.51
121.76
77.27 CDCl3
77.22
77.02 CDCl3
76.77 CDCl3
67.02
65.36
58.40
53.65
53.21
53.05
52.77
52.71
52.10
51.93
38.68
37.30
35.21
32.04
31.84
31.07
MeO2C
CO2Me
Cl
CO2Me
O
Cl
f1 (ppm)

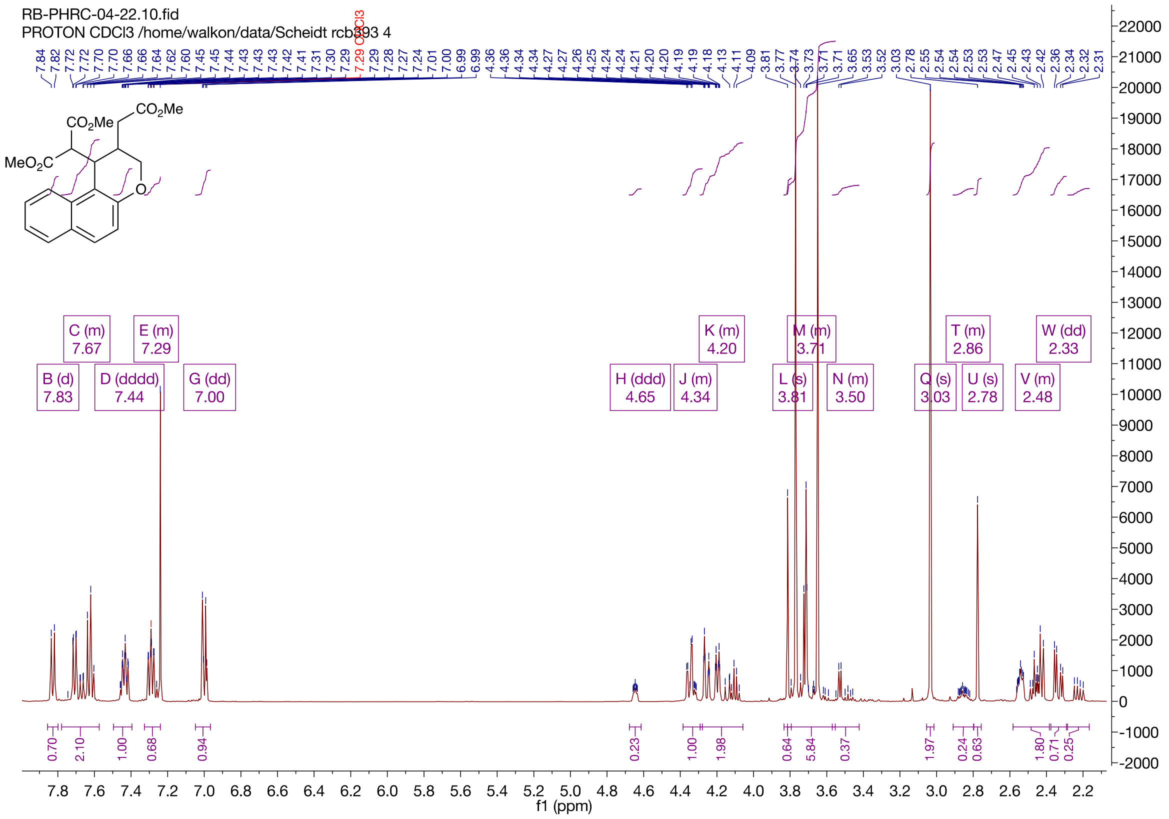
RB-PHRC-04-22.10.fid
PROTON CDCl3 /home/walkon/data/Scheidt rcb393 4
f1 (ppm)

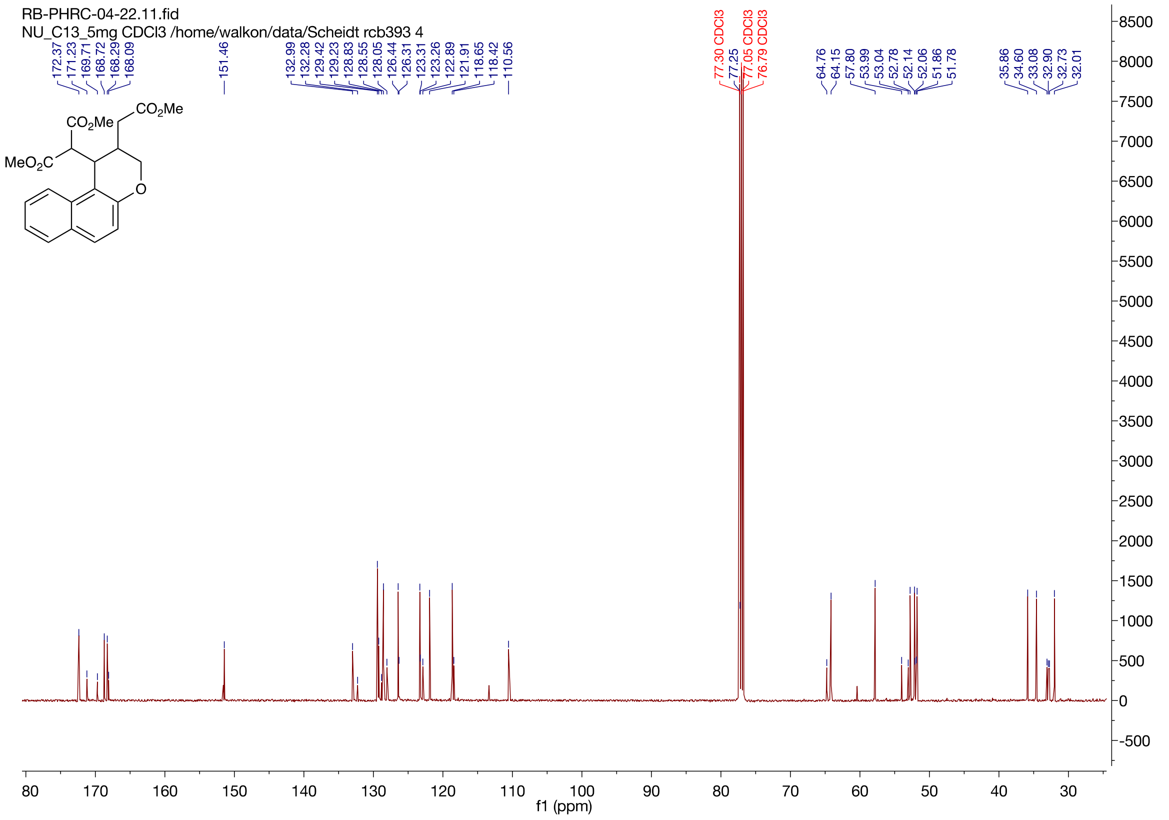
RB-PHRC-04-22.11.fid
NU_C13_5mg CDCl3 /home/walkon/data/Scheidt rcb393 4
f1 (ppm)

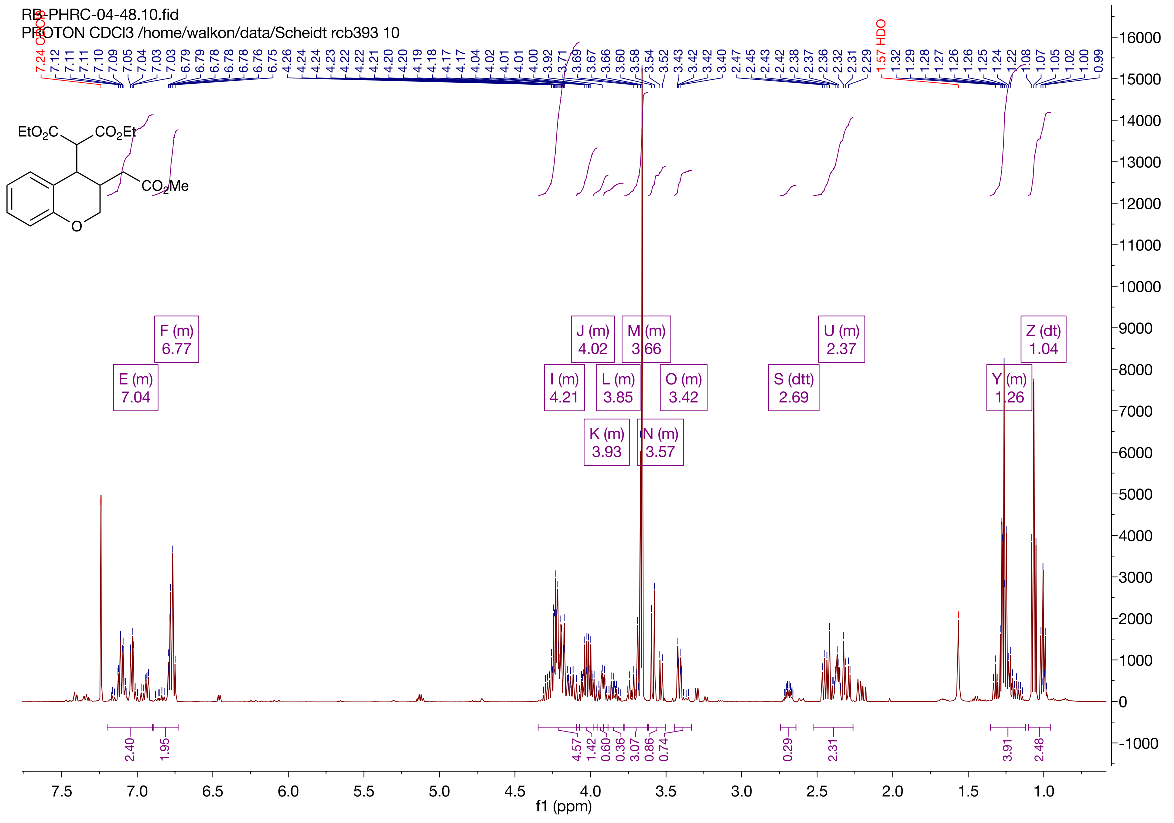
RB-PHRC-04-48.10.fid
PROTON CDCl3 /home/walkon/data/Scheidt rcb393 10
EtO2C
CO2Et
CO2Me
O
f1 (ppm)

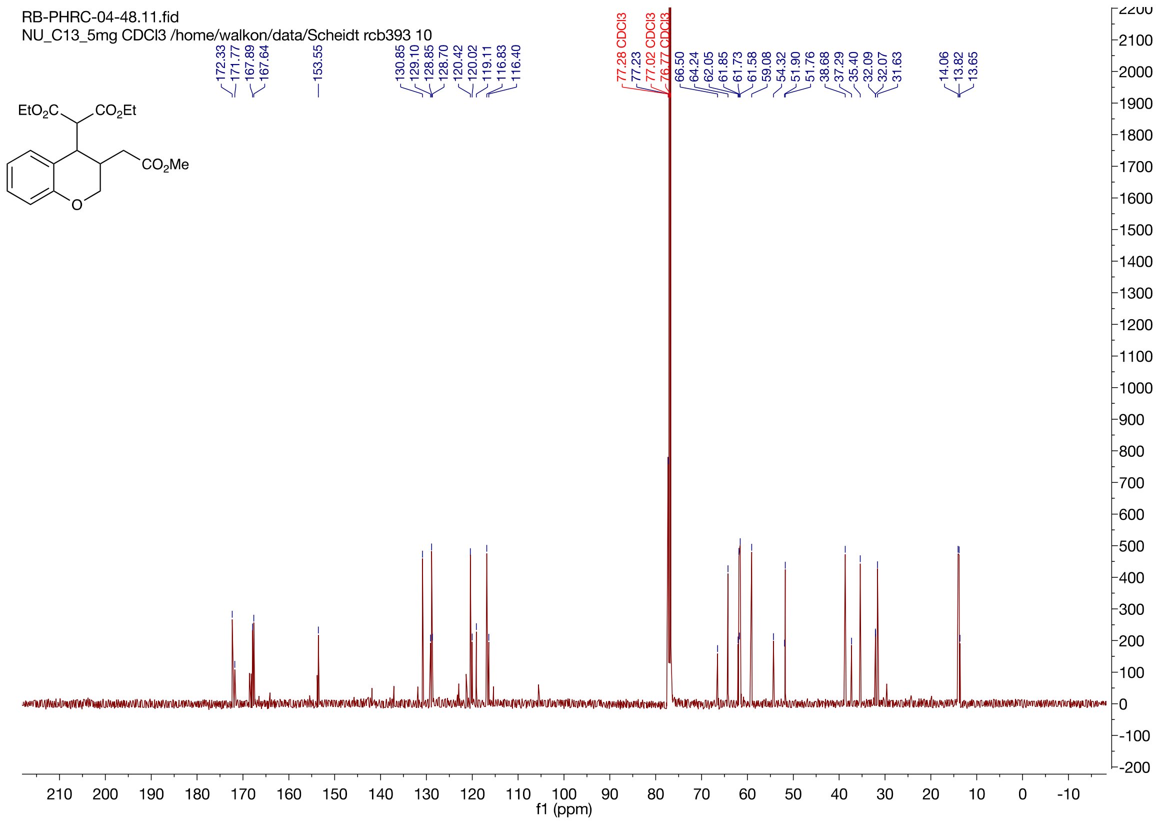
RB-PHRC-04-48.11.fid
NU_C13_5mg CDCl3 /home/walkon/data/Scheidt rcb393 10
EtO2C
CO2Et
CO2Me
O
f1 (ppm)

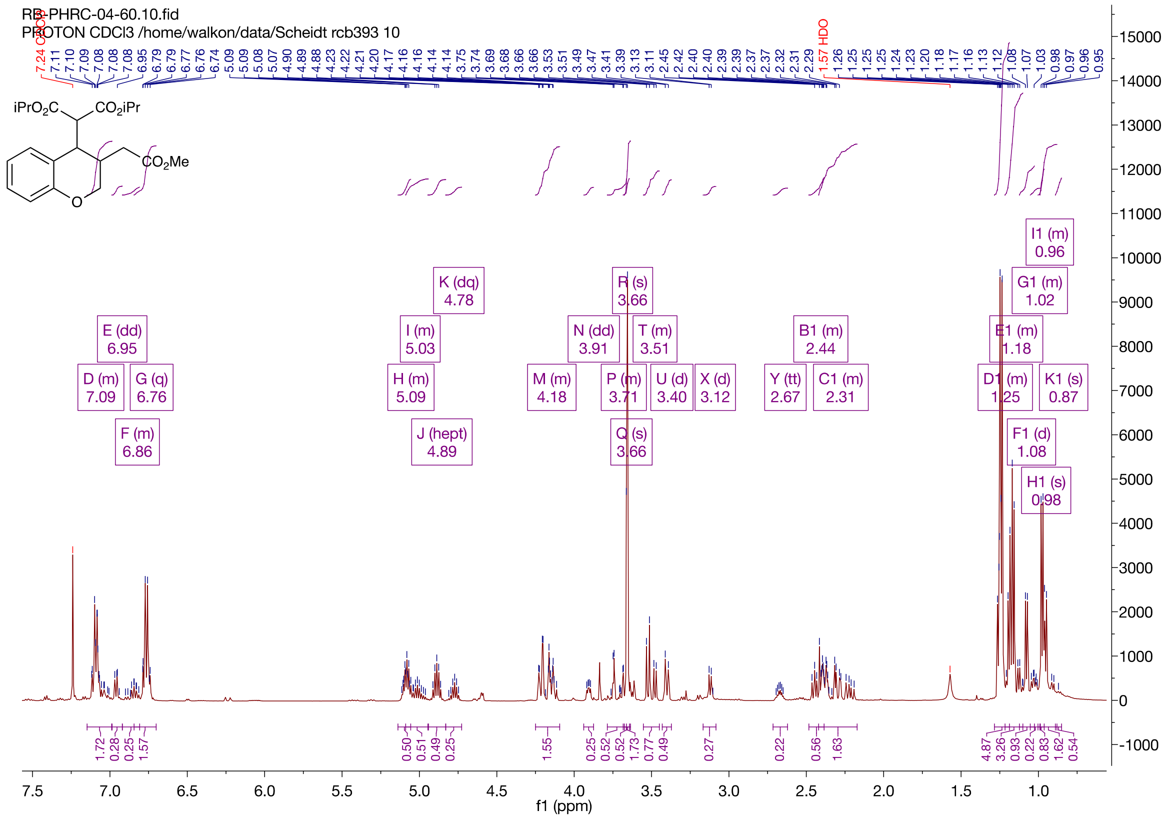

RB-PHRC-04-60.10.fid
PROTON CDCl3 /home/walkon/data/Scheidt rcb393 10
f1 (ppm)

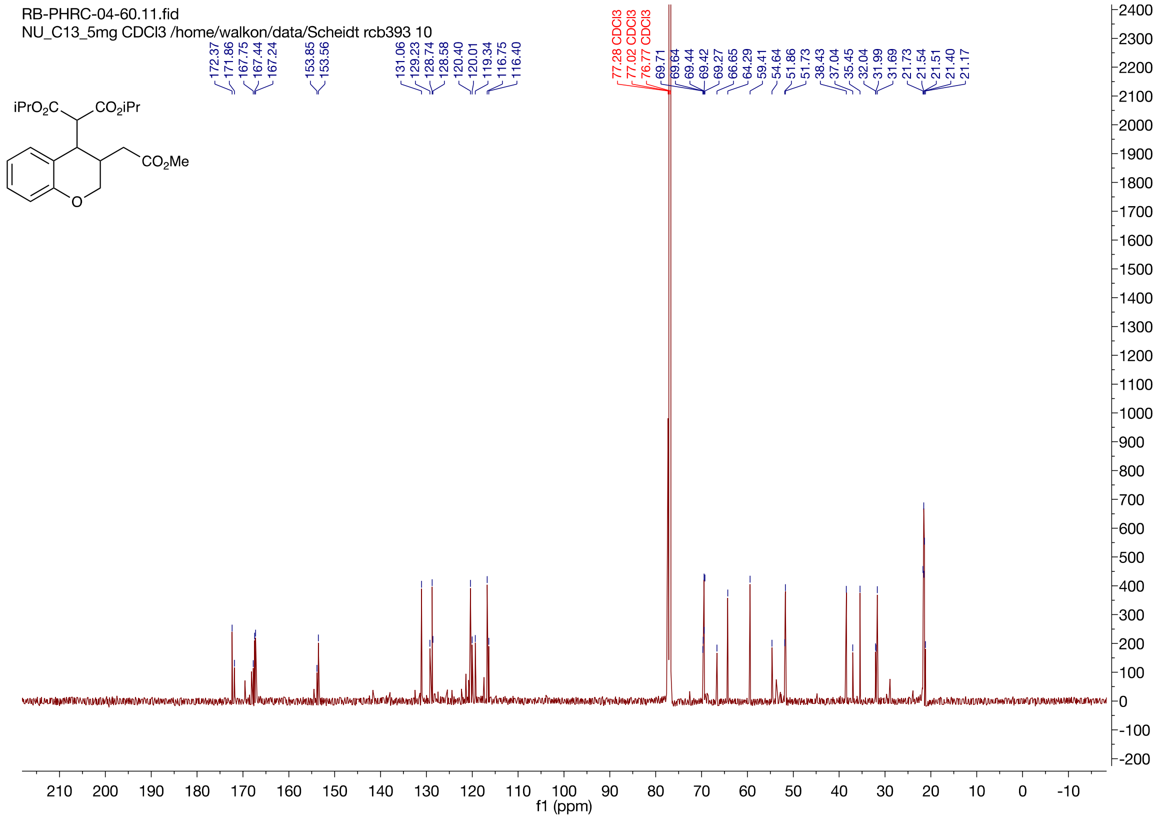

RB-PHRC-04-60.11.fid
NU_C13_5mg CDCl3 /home/walkon/data/Scheidt rcb393 10
f1 (ppm)

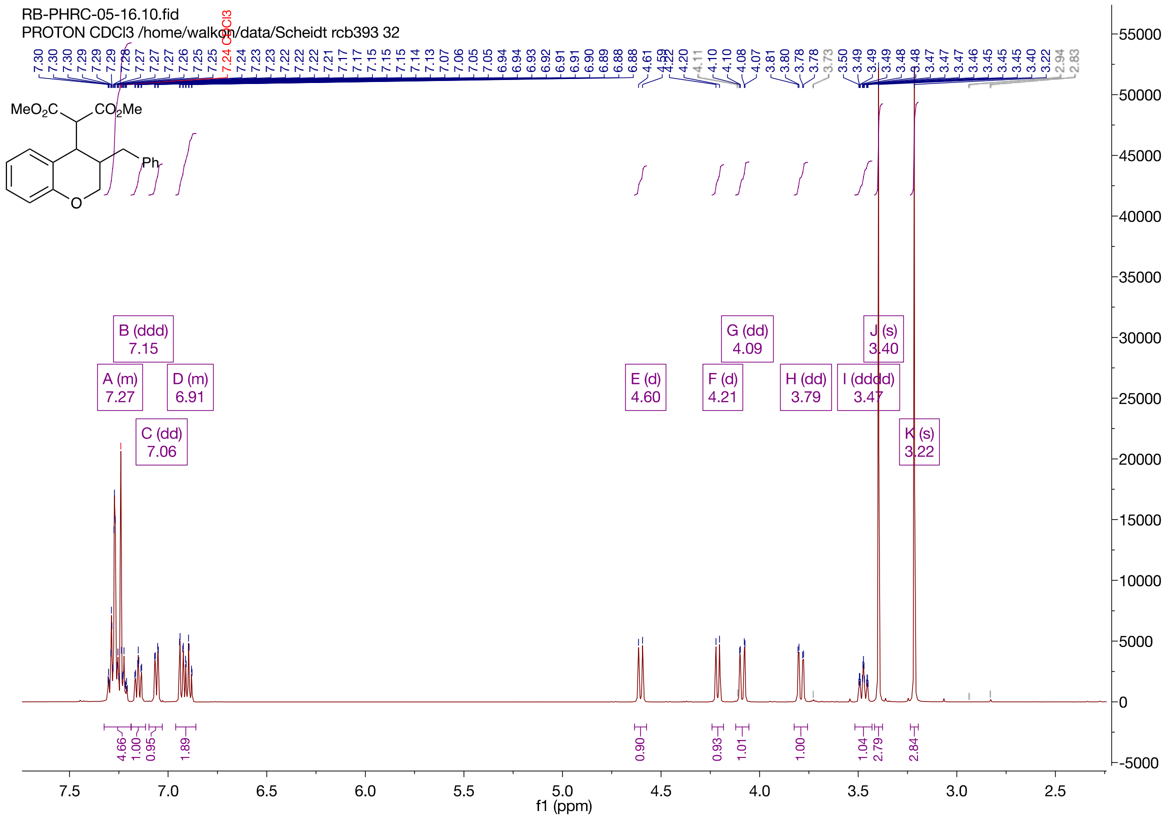
RB-PHRC-05-16.10.fid
PROTON CDCl3 /home/walkon/data/Scheidt rcb393 32
7.30 7.30 7.30 7.29 7.29 7.28 7.27 7.27 7.27 7.26 7.25 7.25 7.24 CDCl3 7.24 7.23 7.23 7.22 7.22 7.22 7.21 7.21 7.17 7.17 7.15 7.15 7.15 7.14 7.13 7.07 7.06 7.05 7.05 6.94 6.94 6.93 6.92 6.91 6.91 6.90 6.89 6.88 6.88 4.61 4.59 4.22 4.20 4.11 4.10 4.10 4.08 4.07 3.81 3.80 3.78 3.78 3.73 3.50 3.49 3.49 3.49 3.48 3.48 3.47 3.47 3.46 3.45 3.45 3.45 3.40 3.22 2.94 2.83
MeO2C
CO2Me
Ph
O
A (m) 7.27
B (ddd) 7.15
C (dd) 7.06
D (m) 6.91
E (d) 4.60
F (d) 4.21
G (dd) 4.09
H (dd) 3.79
I (dddd) 3.47
J (s) 3.40
K (s) 3.22
4.66 1.00 0.95 1.89 0.90 0.93 1.01 1.00 1.04 2.79 2.84
f1 (ppm)

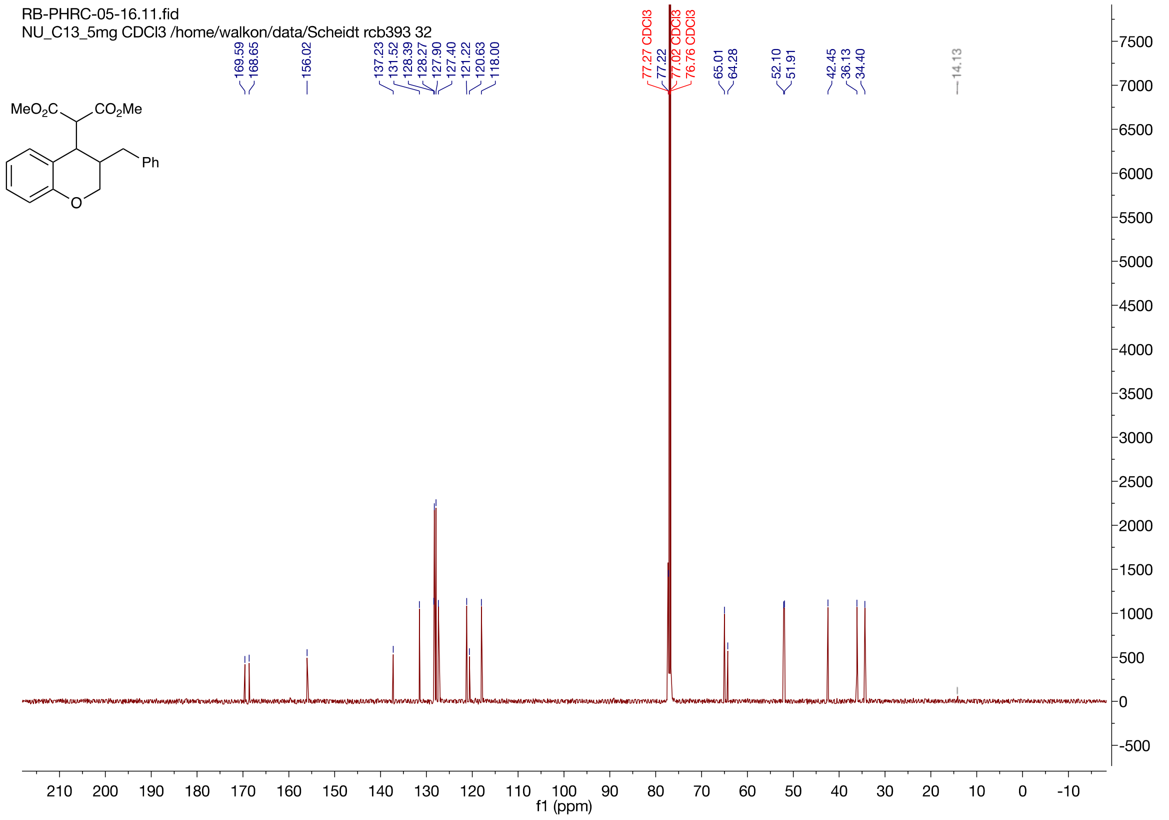
RB-PHRC-05-16.11.fid
NU_C13_5mg CDCl3 /home/walkon/data/Scheidt rcb393 32
169.59 168.65 156.02 137.23 131.52 128.39 128.27 127.90 127.40 121.22 120.63 118.00 77.27 CDCl3 77.22 77.02 CDCl3 76.76 CDCl3 65.01 64.28 52.10 51.91 42.45 36.13 34.40 14.13
MeO2C
CO2Me
Ph
O
f1 (ppm)

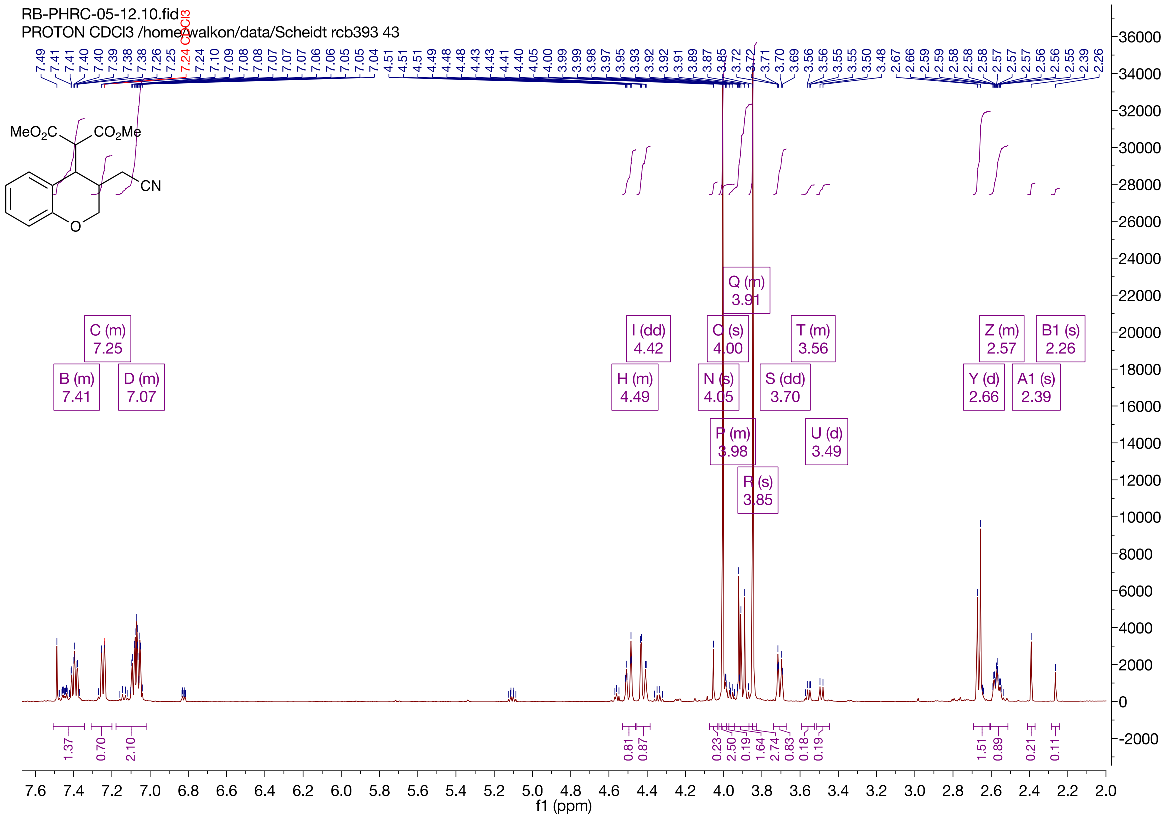
RB-PHRC-05-12.10.fid
PROTON CDCl3 /home/walkon/data/Scheidt rcb393 43
MeO2C
CO2Me
CN
O
C (m) 7.25
B (m) 7.41
D (m) 7.07
I (dd) 4.42
H (m) 4.49
O (s) 4.00
N (s) 4.05
P (m) 3.98
Q (m) 3.91
R (s) 3.85
S (dd) 3.70
T (m) 3.56
U (d) 3.49
Z (m) 2.57
Y (d) 2.66
B1 (s) 2.26
A1 (s) 2.39
f1 (ppm)

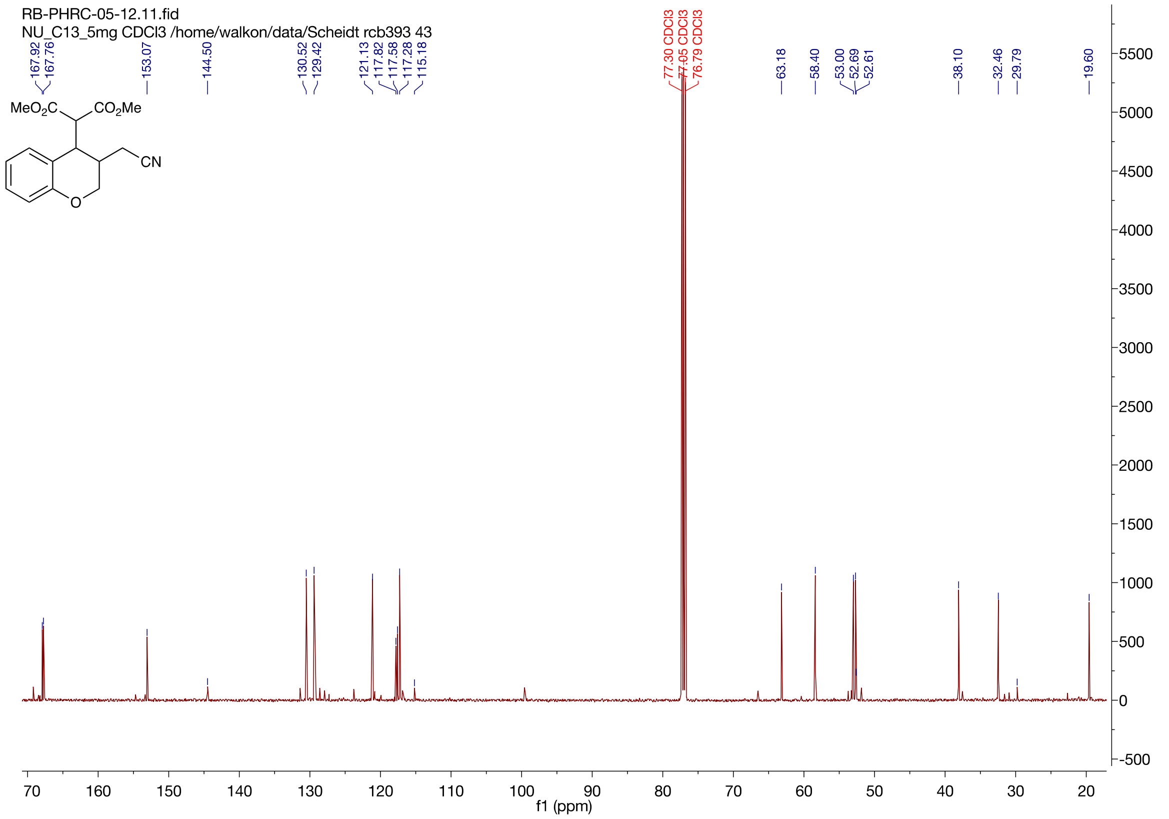
RB-PHRC-05-12.11.fid
NU_C13_5mg CDCl3 /home/walkon/data/Scheidt rcb393 43
167.92
167.76
153.07
144.50
130.52
129.42
121.13
117.82
117.58
117.28
115.18
77.30 CDCl3
77.05 CDCl3
76.79 CDCl3
63.18
58.40
53.00
52.69
52.61
38.10
32.46
29.79
19.60
MeO2C
CO2Me
CN
O
f1 (ppm)

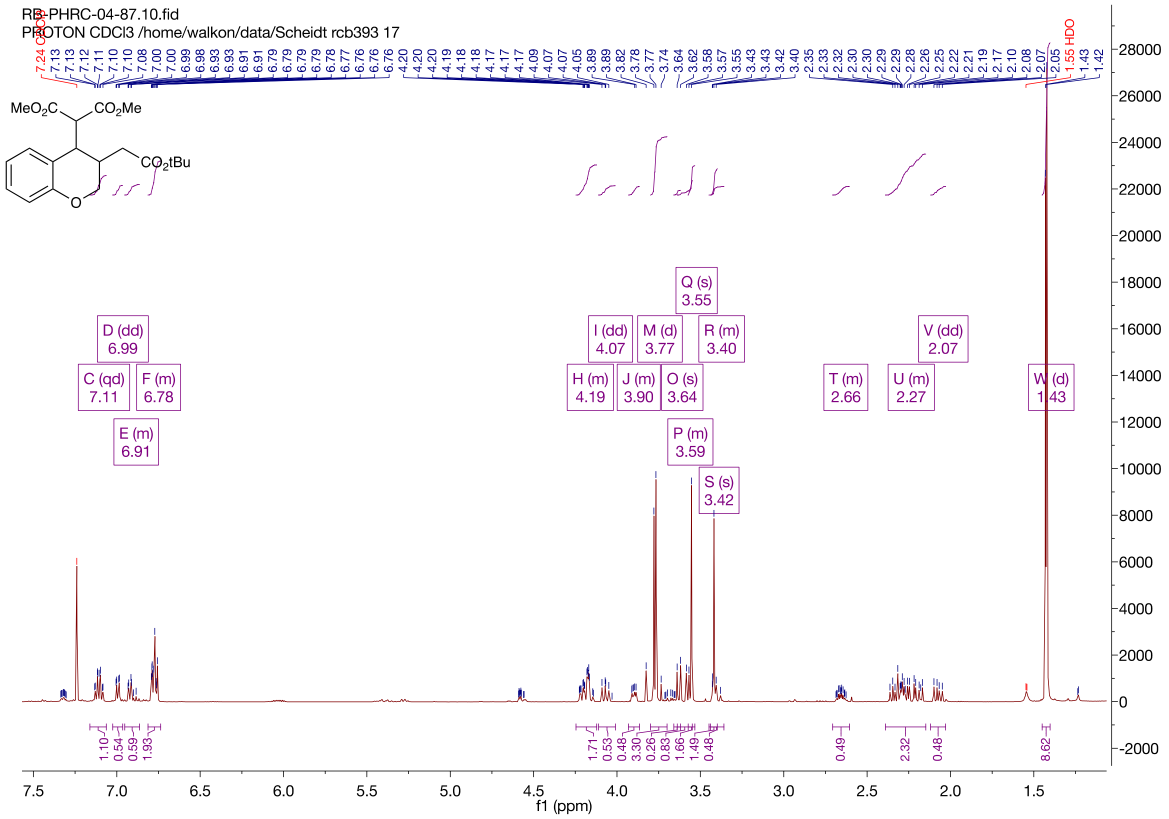

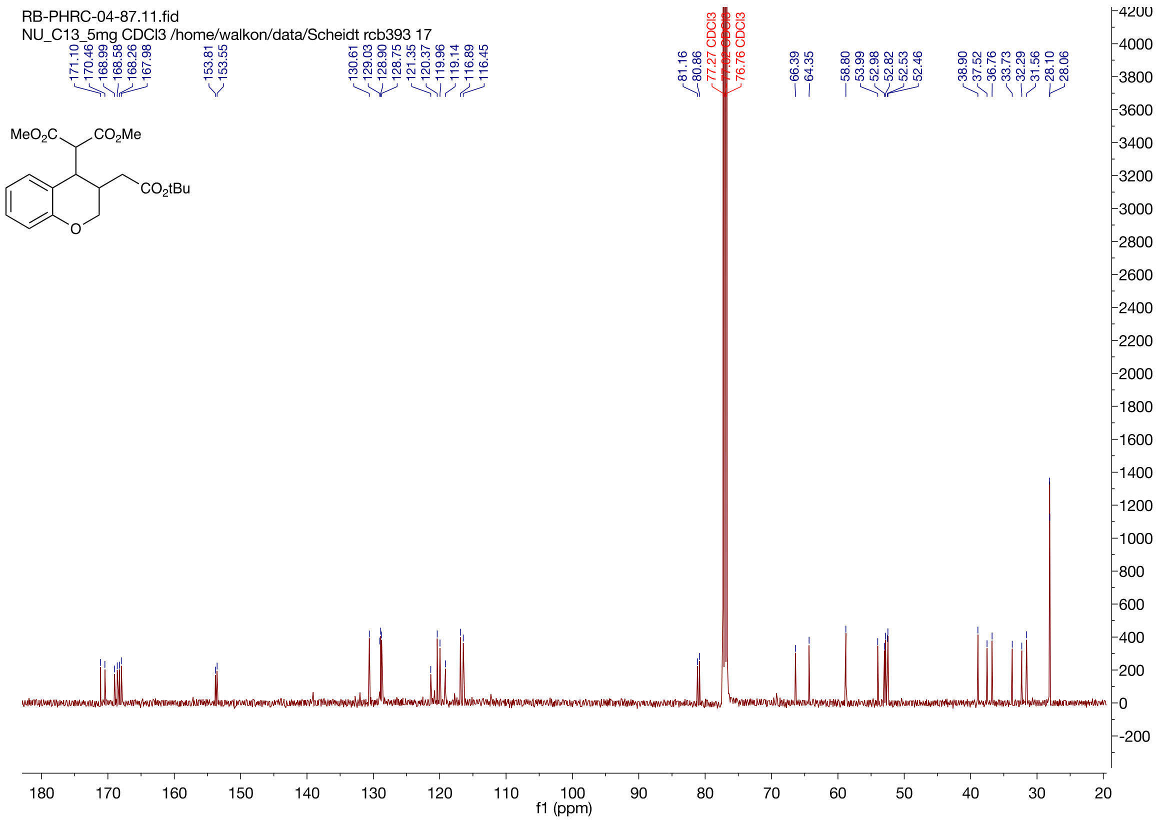

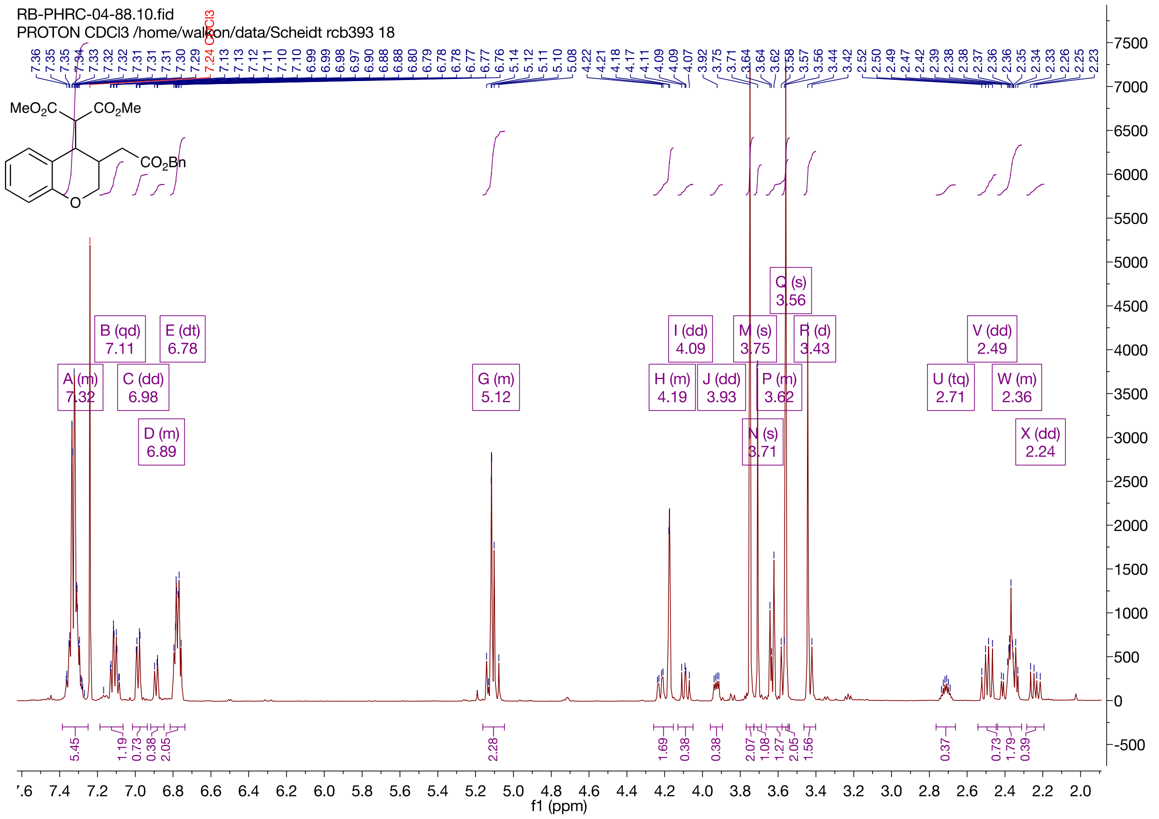
RB-PHRC-04-88.10.fid
PROTON CDCl3 /home/walkon/data/Scheidt rcb393 18
7.36 7.35 7.35 7.34 7.33 7.32 7.32 7.31 7.31 7.31 7.30 7.29 7.24 CDCl3 7.13 7.13 7.12 7.11 7.10 7.10 6.99 6.99 6.98 6.97 6.90 6.88 6.88 6.80 6.79 6.78 6.78 6.77 6.77 6.76 5.14 5.12 5.11 5.10 5.08 4.22 4.21 4.18 4.17 4.11 4.09 4.09 4.07 3.92 3.75 3.71 3.64 3.64 3.62 3.58 3.57 3.56 3.44 3.42 2.52 2.50 2.49 2.47 2.42 2.39 2.38 2.38 2.37 2.36 2.36 2.35 2.34 2.33 2.26 2.25 2.23
MeO2C CO2Me CO2Bn O
A (m) 7.32
B (qd) 7.11
C (dd) 6.98
D (m) 6.89
E (dt) 6.78
G (m) 5.12
H (m) 4.19
I (dd) 4.09
J (dd) 3.93
M (s) 3.75
N (s) 3.71
P (m) 3.62
Q (s) 3.56
R (d) 3.43
U (tq) 2.71
V (dd) 2.49
W (m) 2.36
X (dd) 2.24
5.45 1.19 0.73 0.38 2.05 2.28 1.69 0.38 0.38 2.07 1.08 1.27 2.05 1.56 0.37 0.73 1.79 0.39
f1 (ppm)

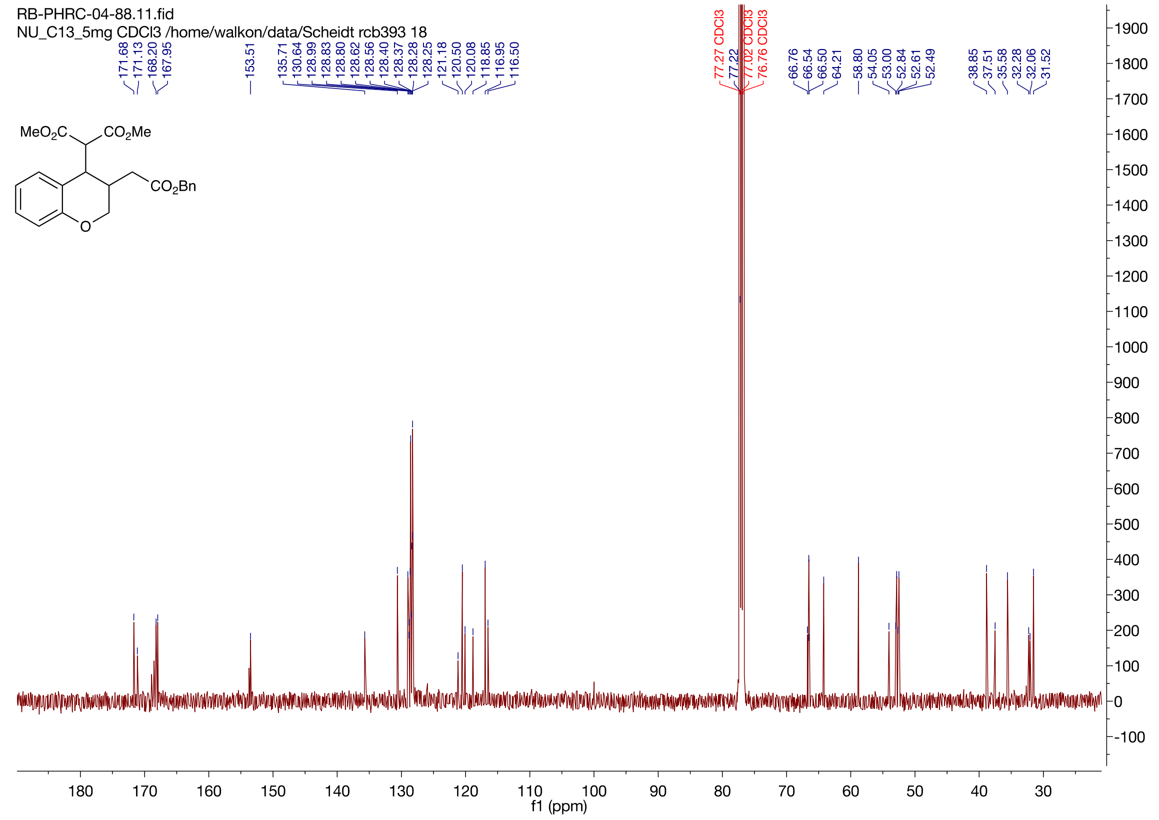
RB-PHRC-04-88.11.fid
NU_C13_5mg CDCl3 /home/walkon/data/Scheidt rcb393 18
171.68 171.13 168.20 167.95 153.51 135.71 130.64 128.99 128.83 128.80 128.62 128.56 128.40 128.37 128.28 128.25 121.18 120.50 120.08 118.85 116.95 116.50 77.27 CDCl3 77.22 77.02 CDCl3 76.76 CDCl3 66.76 66.54 66.50 64.21 58.80 54.05 53.00 52.84 52.61 52.49 38.85 37.51 35.58 32.28 32.06 31.52
MeO2C CO2Me CO2Bn O
f1 (ppm)

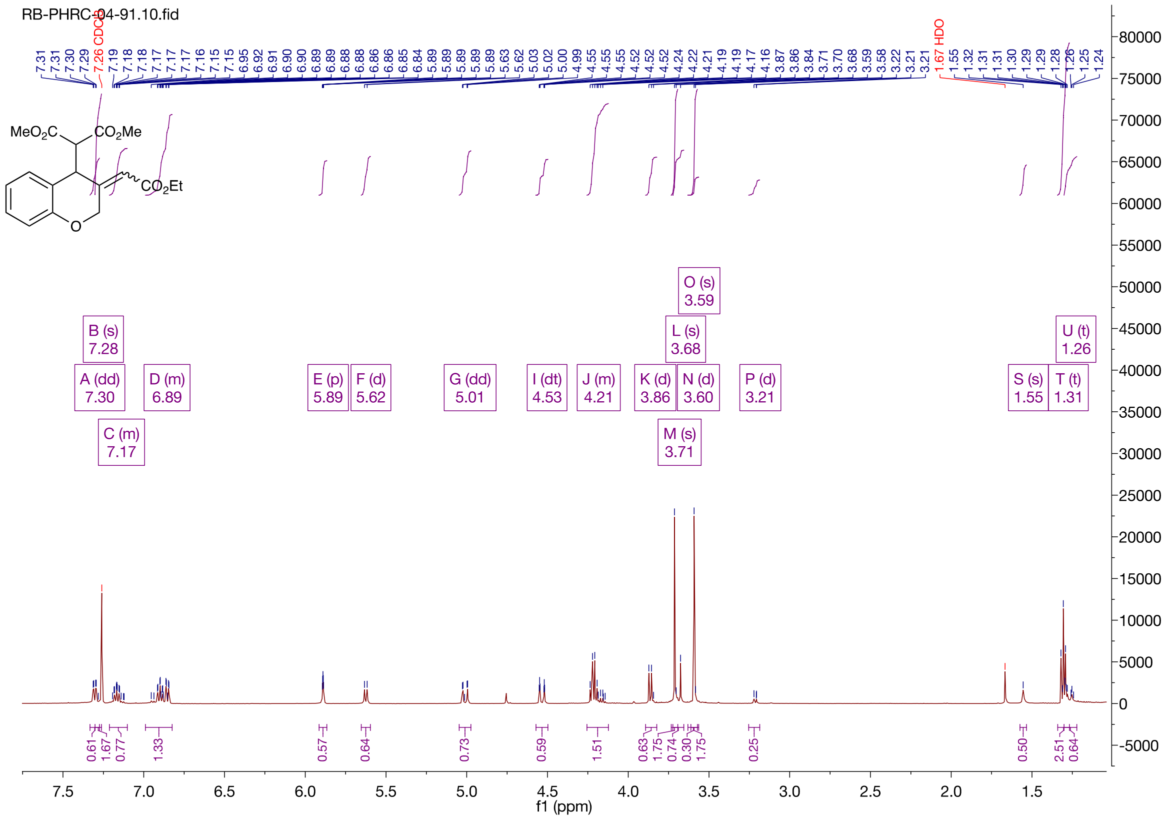
RB-PHRC-04-91.10.fid
7.31 7.31 7.30 7.29 7.26 CDCl3 7.19 7.18 7.18 7.17 7.17 7.17 7.16 7.15 7.15 6.95 6.92 6.91 6.90 6.90 6.89 6.89 6.89 6.88 6.88 6.86 6.86 6.85 6.84 5.89 5.89 5.89 5.89 5.63 5.62 5.03 5.02 5.00 4.99 4.55 4.55 4.55 4.52 4.52 4.52 4.24 4.22 4.21 4.19 4.19 4.17 4.16 3.87 3.86 3.84 3.71 3.70 3.68 3.59 3.58 3.22 3.21 1.67 HDO 1.55 1.32 1.31 1.31 1.30 1.29 1.29 1.28 1.26 1.25 1.24
MeO2C
CO2Me
CO2Et
O
A (dd) 7.30
B (s) 7.28
C (m) 7.17
D (m) 6.89
E (p) 5.89
F (d) 5.62
G (dd) 5.01
I (dt) 4.53
J (m) 4.21
K (d) 3.86
L (s) 3.68
M (s) 3.71
N (d) 3.60
O (s) 3.59
P (d) 3.21
S (s) 1.55
T (t) 1.31
U (t) 1.26
0.61 1.67 0.77 1.33 0.57 0.64 0.73 0.59 1.51 0.63 1.75 0.74 0.30 1.75 0.25 0.50 2.51 0.64
f1 (ppm)

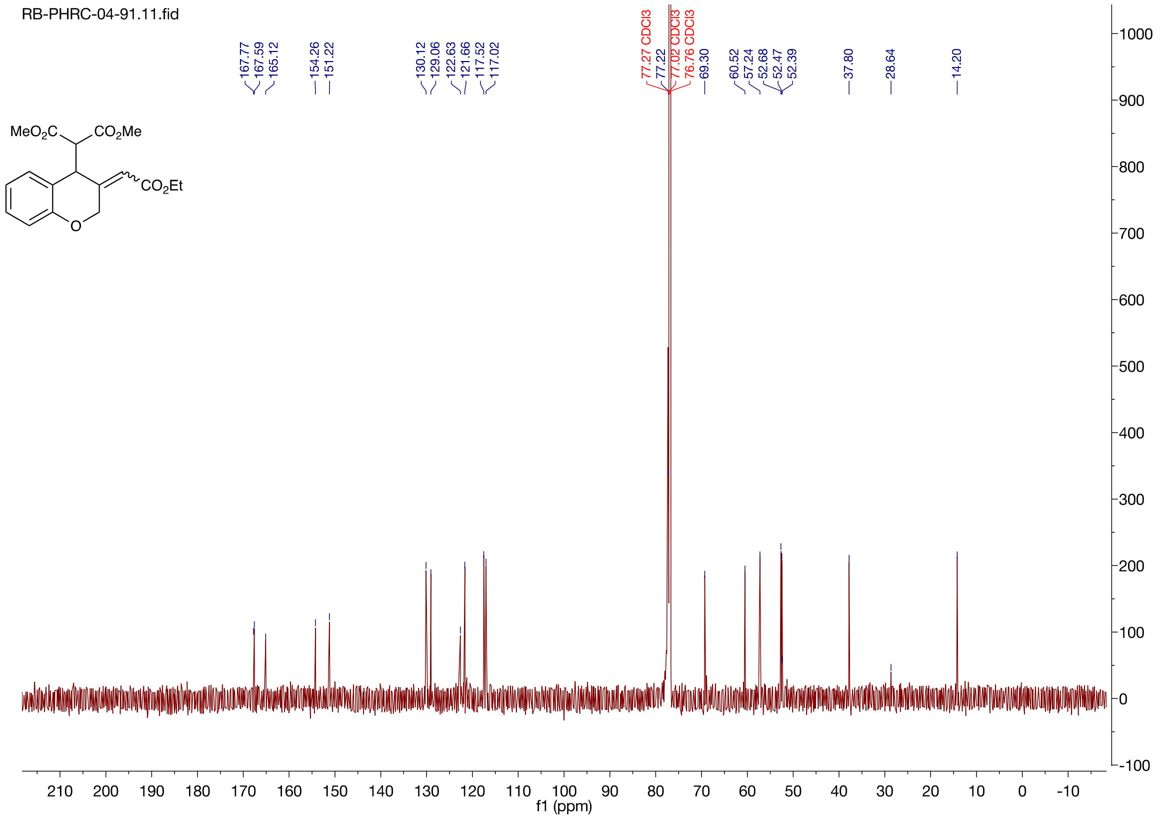
RB-PHRC-04-91.11.fid
167.77 167.59 165.12 154.26 151.22 130.12 129.06 122.63 121.66 117.52 117.02 77.27 CDCl3 77.22 77.02 CDCl3 76.76 CDCl3 69.30 60.52 57.24 52.68 52.47 52.39 37.80 28.64 14.20
MeO2C
CO2Me
CO2Et
O
f1 (ppm)

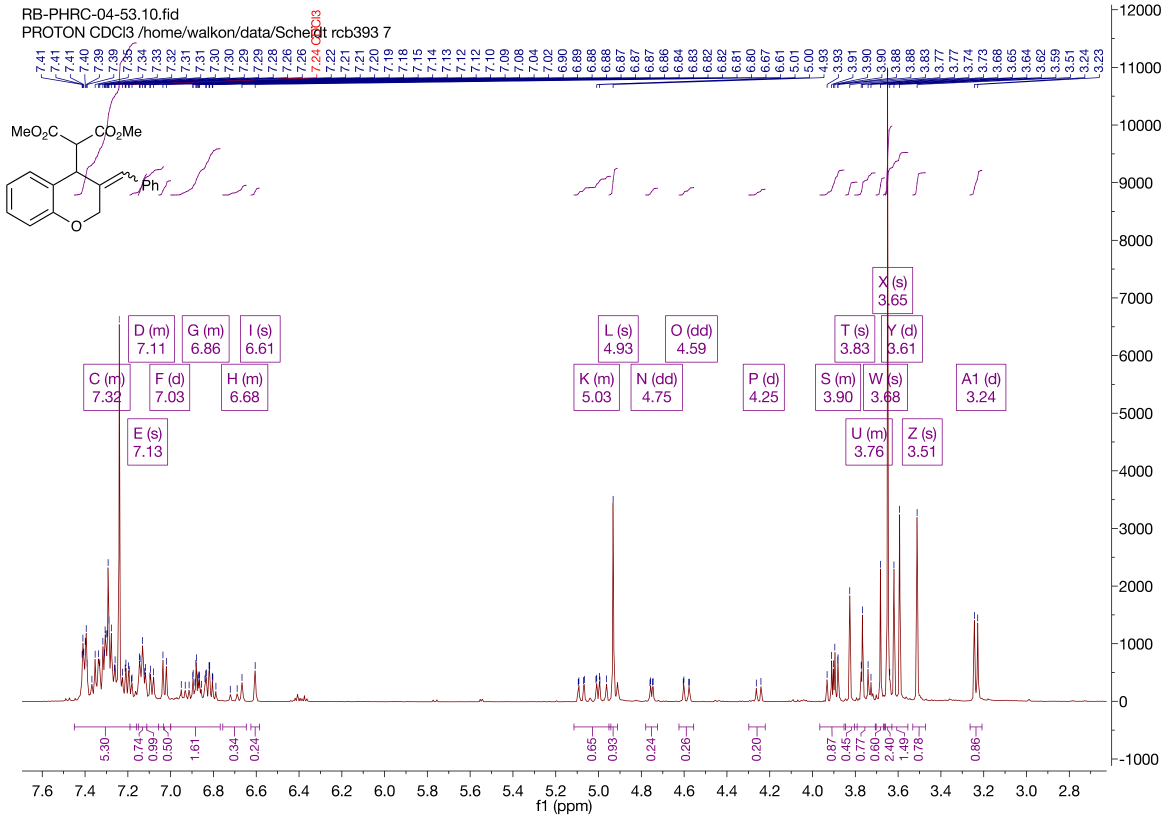
RB-PHRC-04-53.10.fid
PROTON CDCl3 /home/walkon/data/Scheidt rcb393 7
7.41 7.41 7.41 7.40 7.39 7.39 7.35 7.34 7.33 7.32 7.31 7.31 7.30 7.30 7.29 7.29 7.28 7.26 7.26 7.24 CDCl3 7.22 7.21 7.21 7.20 7.19 7.18 7.15 7.14 7.13 7.12 7.12 7.10 7.09 7.08 7.04 7.02 6.90 6.89 6.88 6.88 6.87 6.87 6.87 6.86 6.84 6.83 6.82 6.82 6.81 6.80 6.67 6.61 5.01 5.00 4.93 3.93 3.91 3.90 3.90 3.88 3.88 3.83 3.77 3.74 3.73 3.68 3.65 3.64 3.62 3.59 3.51 3.24 3.23
MeO2C
CO2Me
Ph
O
C (m) 7.32
D (m) 7.11
E (s) 7.13
F (d) 7.03
G (m) 6.86
H (m) 6.68
I (s) 6.61
K (m) 5.03
L (s) 4.93
N (dd) 4.75
O (dd) 4.59
P (d) 4.25
S (m) 3.90
T (s) 3.83
U (m) 3.76
W (s) 3.68
X (s) 3.65
Y (d) 3.61
Z (s) 3.51
A1 (d) 3.24
5.30 0.74 0.99 0.50 1.61 0.34 0.24 0.65 0.93 0.24 0.26 0.20 0.87 0.45 0.77 0.60 2.40 1.49 0.78 0.86
f1 (ppm)

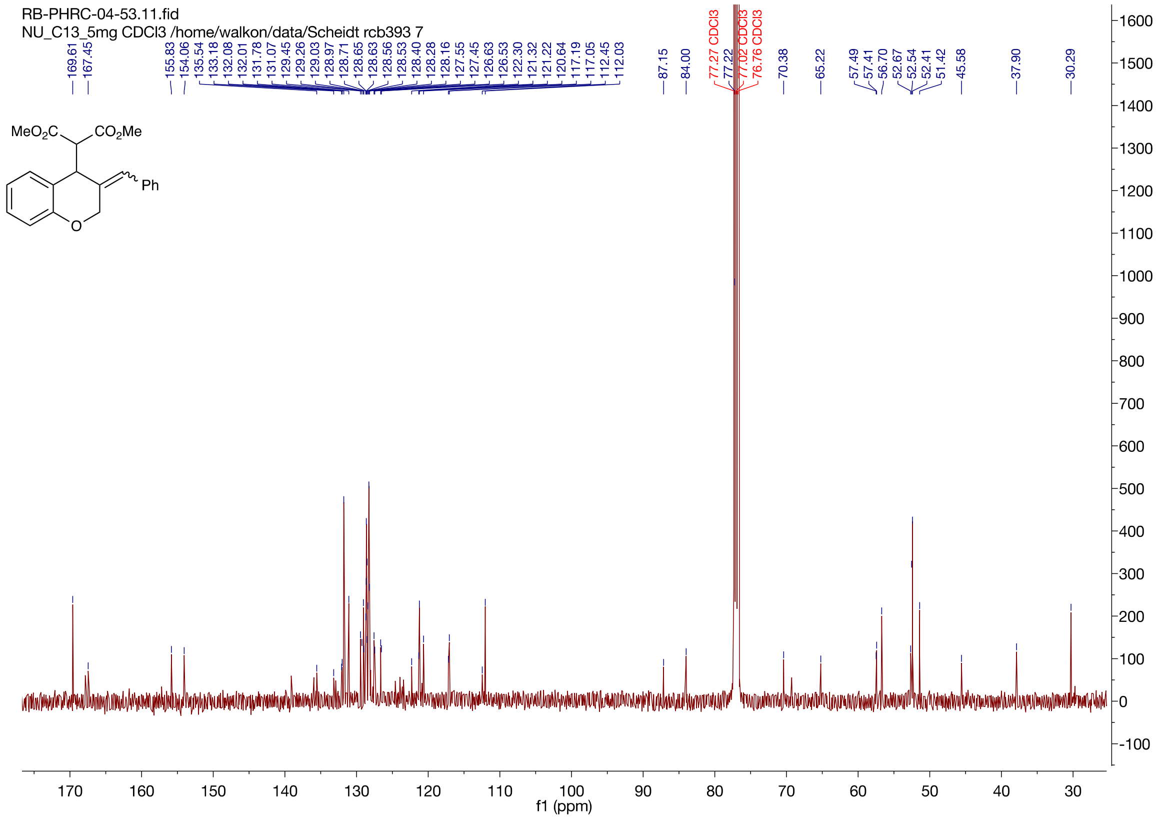
RB-PHRC-04-53.11.fid
NU_C13_5mg CDCl3 /home/walkon/data/Scheidt rcb393 7
169.61 167.45 155.83 154.06 135.54 133.18 132.08 132.01 131.78 131.07 129.45 129.26 129.03 128.97 128.71 128.65 128.63 128.56 128.53 128.40 128.28 128.16 127.55 127.45 126.63 126.53 122.30 121.32 121.22 120.64 117.19 117.05 112.45 112.03 87.15 84.00 77.27 CDCl3 77.22 77.02 CDCl3 76.76 CDCl3 70.38 65.22 57.49 57.41 56.70 52.67 52.54 52.41 51.42 45.58 37.90 30.29
MeO2C
CO2Me
Ph
O
f1 (ppm)

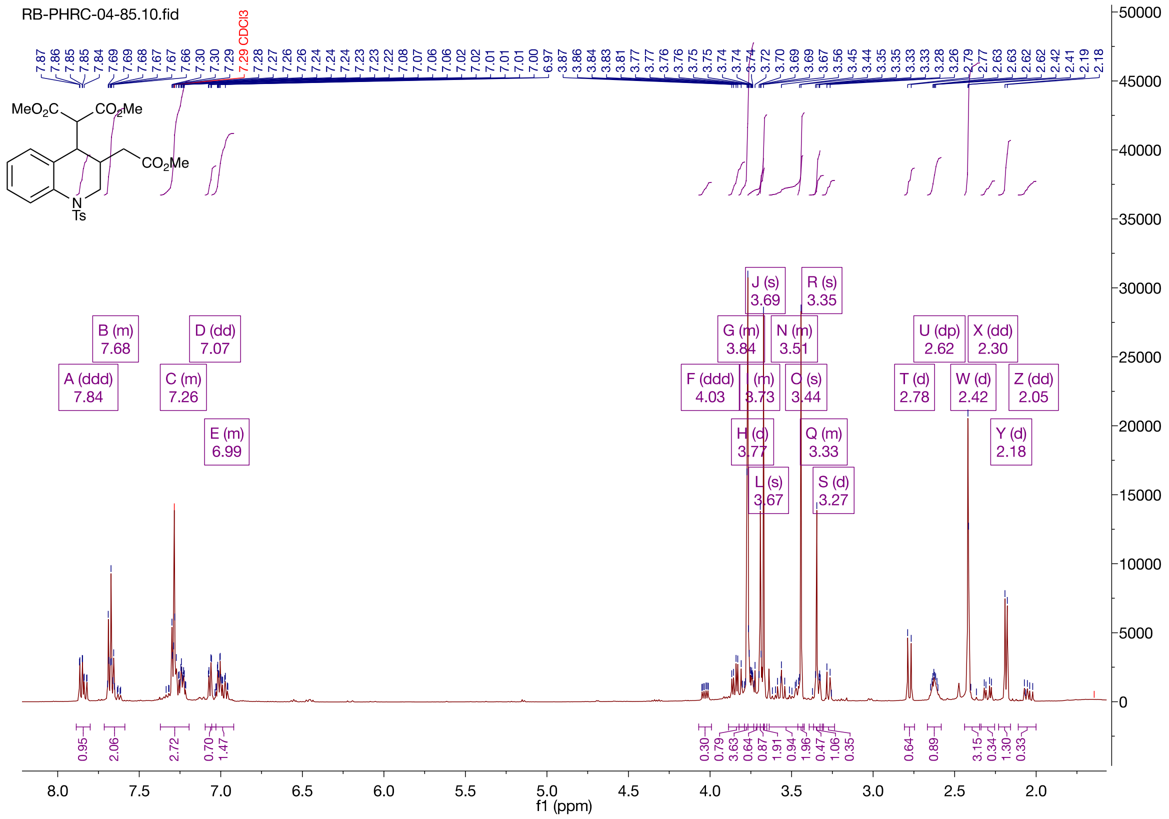
RB-PHRC-04-85.10.fid
f1 (ppm)

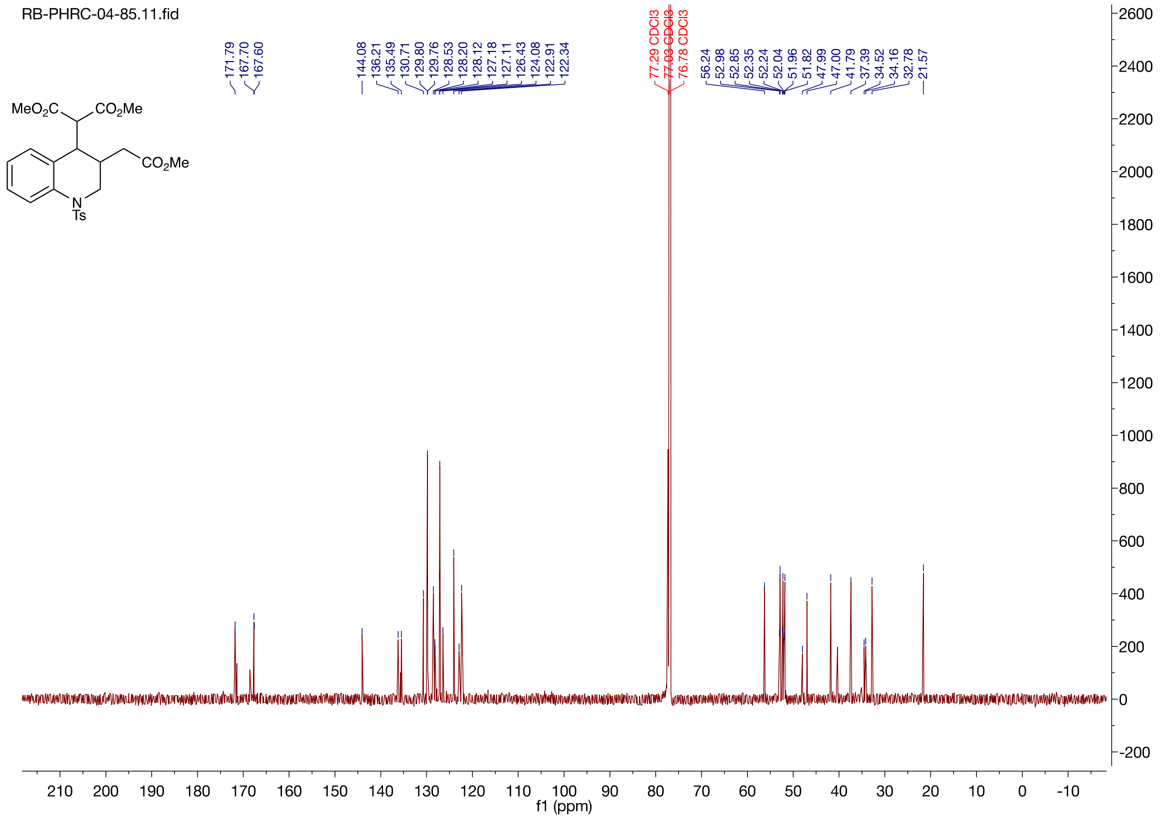
RB-PHRC-04-85.11.fid
f1 (ppm)

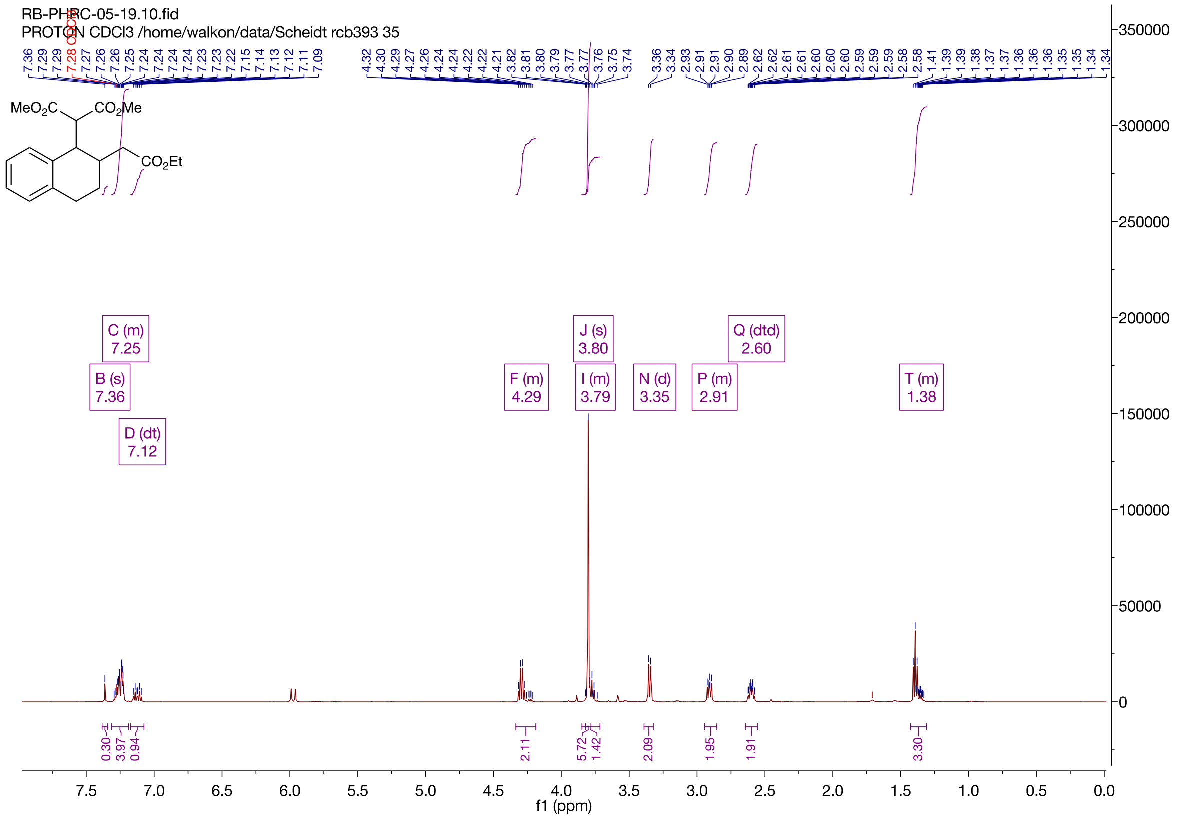
RB-PHRC-05-19.10.fid
PROTON CDCl3 /home/walkon/data/Scheidt rcb393 35
MeO2C CO2Me CO2Et
B (s) 7.36
C (m) 7.25
D (dt) 7.12
F (m) 4.29
J (s) 3.80
I (m) 3.79
N (d) 3.35
P (m) 2.91
Q (dtd) 2.60
T (m) 1.38
f1 (ppm)

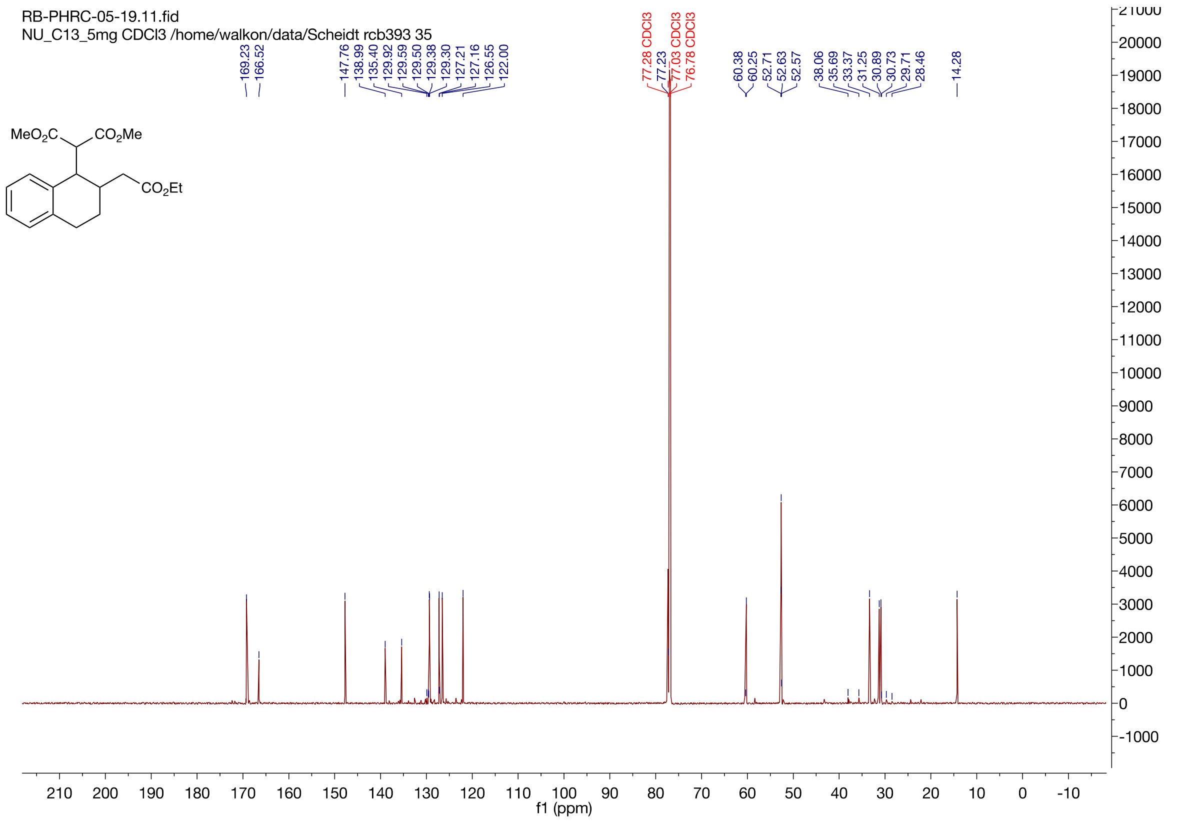
RB-PHRC-05-19.11.fid
NU_C13_5mg CDCl3 /home/walkon/data/Scheidt rcb393 35
169.23
166.52
147.76
138.99
135.40
129.92
129.59
129.50
129.38
129.30
127.21
127.16
126.55
122.00
77.28 CDCl3
77.23
77.03 CDCl3
76.78 CDCl3
60.38
60.25
52.71
52.63
52.57
38.06
35.69
33.37
31.25
30.89
30.73
29.71
28.46
14.28
MeO2C CO2Me CO2Et
f1 (ppm)

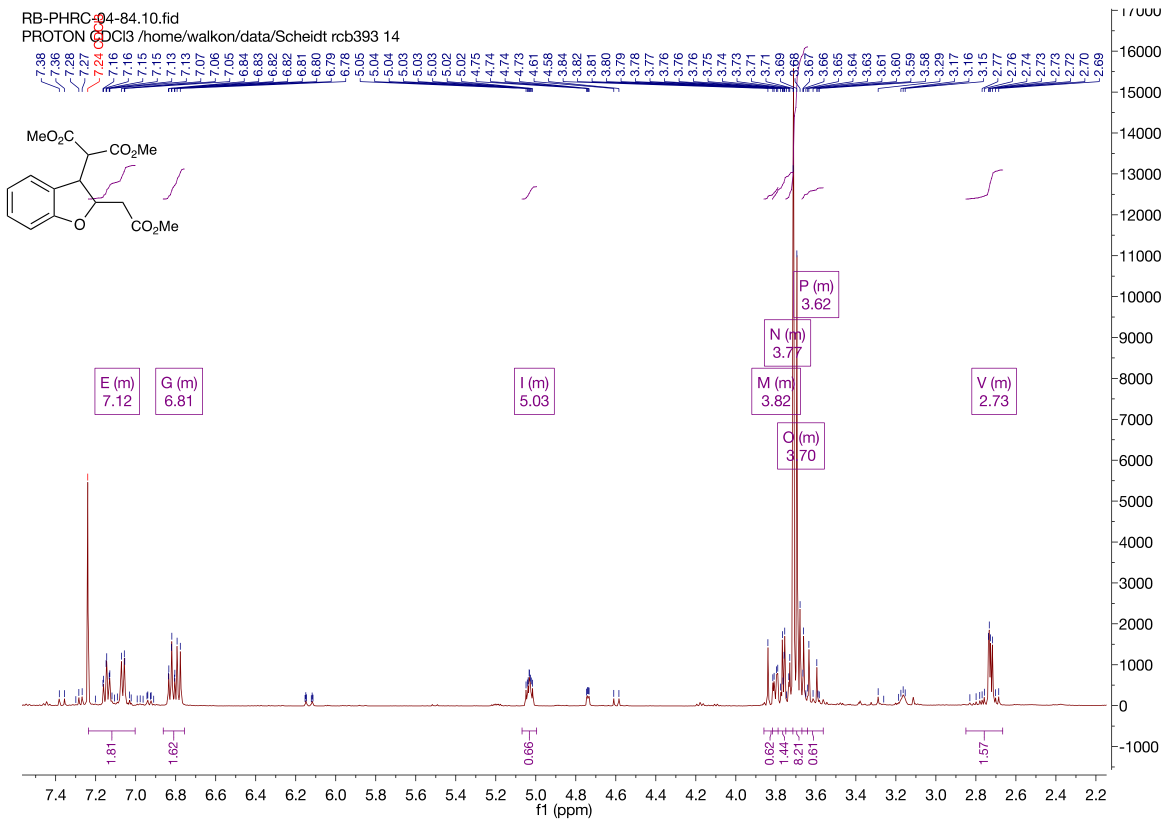
RB-PHRC-04-84.10.fid
PROTON CDCl3 /home/walkon/data/Scheidt rcb393 14
7.38 7.36 7.28 7.27 7.24 CDCl3 7.16 7.16 7.15 7.15 7.13 7.13 7.07 7.06 7.05 6.84 6.83 6.82 6.82 6.81 6.80 6.79 6.78 5.05 5.04 5.03 5.03 5.03 5.02 5.02 4.75 4.74 4.74 4.73 4.61 4.58 3.84 3.82 3.81 3.80 3.79 3.78 3.77 3.76 3.76 3.75 3.74 3.73 3.71 3.69 3.68 3.67 3.66 3.65 3.64 3.63 3.61 3.60 3.59 3.58 3.29 3.17 3.16 3.15 2.77 2.76 2.74 2.73 2.73 2.72 2.70 2.69
MeO2C
CO2Me
O
CO2Me
E (m) 7.12
G (m) 6.81
I (m) 5.03
M (m) 3.82
N (m) 3.77
O (m) 3.70
P (m) 3.62
V (m) 2.73
1.81
1.62
0.66
0.62
1.44
8.21
0.61
1.57
f1 (ppm)

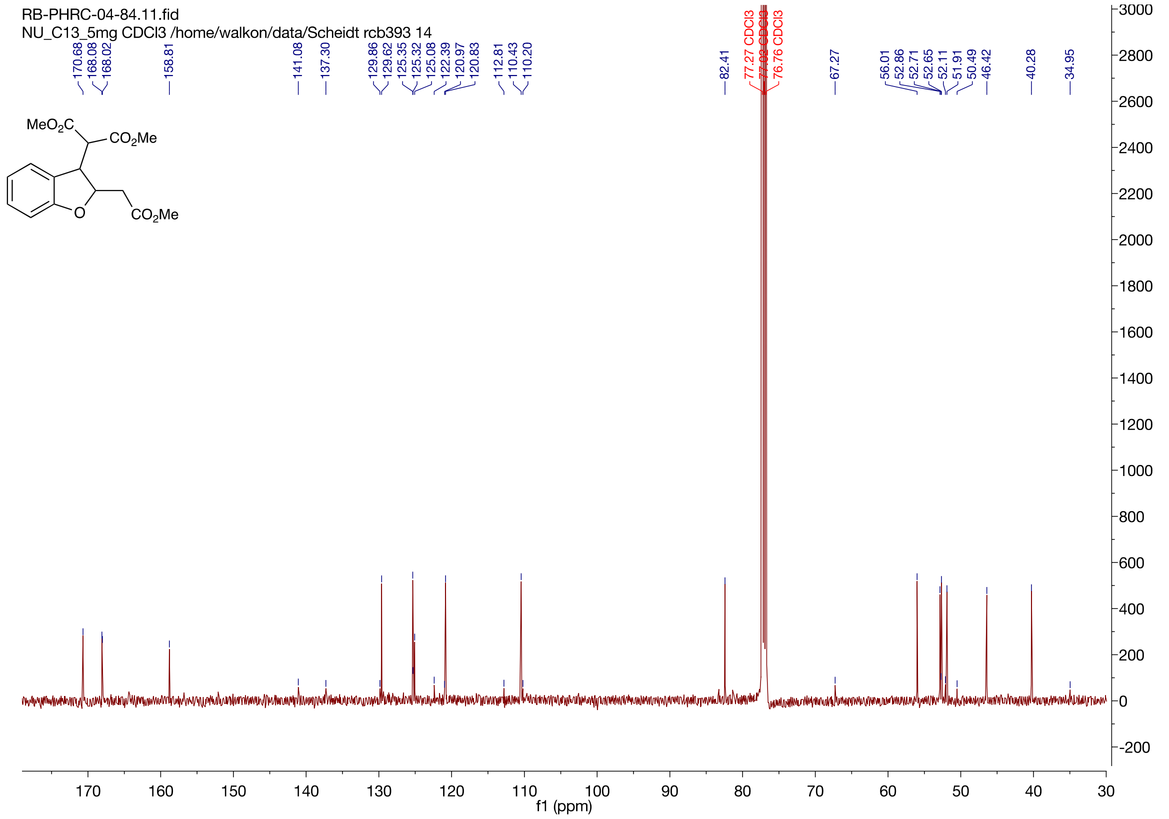
RB-PHRC-04-84.11.fid
NU_C13_5mg CDCl3 /home/walkon/data/Scheidt rcb393 14
170.68 168.08 168.02
158.81
141.08 137.30
129.86 129.62 125.35 125.32 125.08 122.39 120.97 120.83
112.81 110.43 110.20
82.41
77.27 CDCl3 77.02 CDCl3 76.76 CDCl3
67.27
56.01 52.86 52.71 52.65 52.11 51.91 50.49 46.42
40.28
34.95
MeO2C
CO2Me
O
CO2Me
f1 (ppm)

rb-phrc-05-21-crop1-col.10.fid
PROTON CDCl3 /home/walkon/data/Scheidt rcb393 2

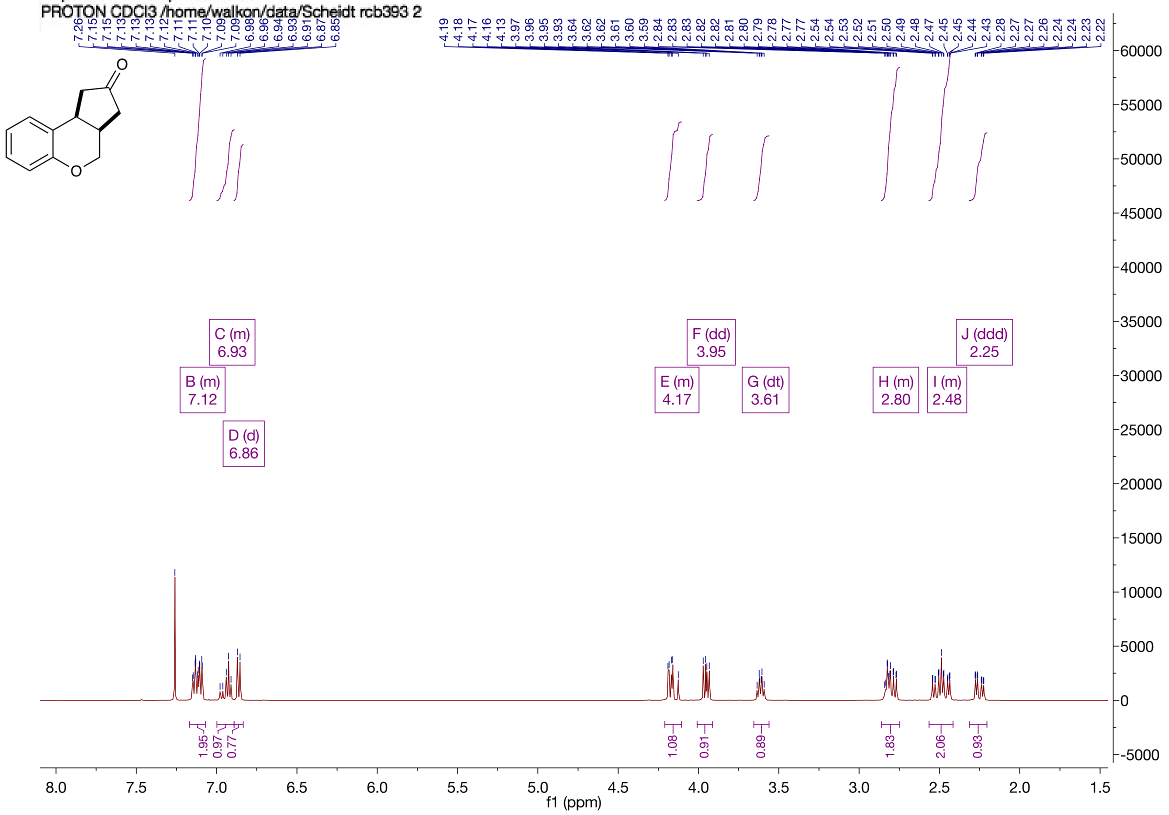

rb-phrc-05-21-crop1-col.11.fid
NU_C13_5mg CDCl3 /home/walkon/data/Scheidt rcb393 2

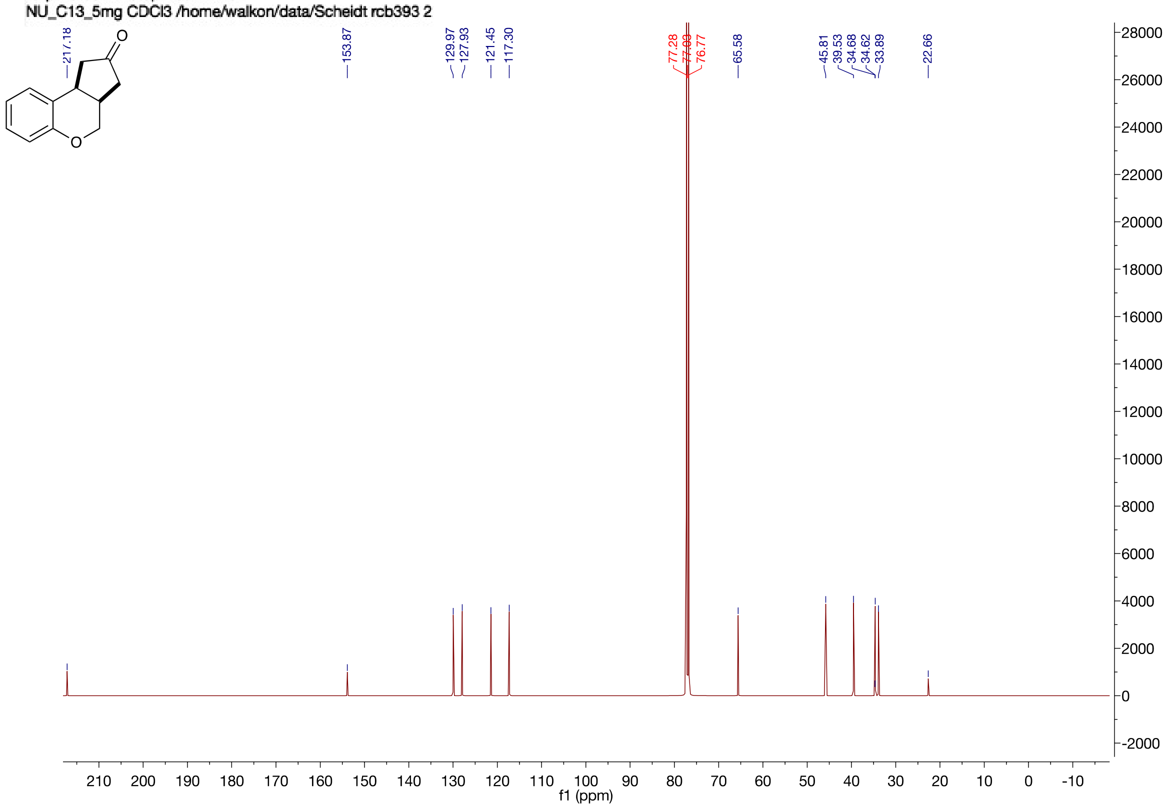

References:

1. Perrin, D. D.; Armarego, W. L. F., *Purification of Laboratory Chemicals*. 3rd ed.; Pergamon Press: Oxford, 1988.
2. Lin, K.; Wiles, R. J.; Kelly, C. B.; Davies, G. H. M.; Molander, G. A. *Acs Catal* **2017,** *7*, 5129
3. Cismesia, M. A.; Yoon, T. P. *Chem. Sci.* **2015,** *6*, 5426
